# Thin-Layer Electrochemistry: Visualizing the Diffusion Layer During Chronoamperometry and Cyclic Voltammetry

**DOI:** 10.1021/acs.jchemed.5c01710

**Published:** 2026-07-16

**Authors:** Mohammad Rafiee, Lida Khalafi, Keya Pandey, Mary Gipson

**Affiliations:** † Division of Energy, Matter and Systems, School of Science and Engineering, 12273 University of Missouri−Kansas City, Kansas City, Missouri 64110, United States; ‡ College of Arts and Sciences, 4191 Avila University, Kansas City, Missouri 64145, United States

**Keywords:** Hands-on Illustration, Hands-on Learning/manipulatives, First-Year Undergraduate/General, Upper-Division Undergraduate, Electrochemistry, Oxidation/Reduction, Redox Indicator, Diffusion Layer, Diffusion Coefficient, Chronoamperometry, Cyclic Voltammetry

## Abstract

The phenomena that occur in the diffusion layer play a central role in electrochemical processes but are often challenging for students to understand. Here, we present a demonstration and activity to directly observe and analyze the changes in diffusion layer during chronoamperometry and cyclic voltammetry. Methyl viologen (MV^2+^), a redox indicator with distinct color changes upon one-electron reduction, was used to generate an observable color change. The reaction was carried out in a homemade thin-layer cell, and the color changes were monitored and recorded using an optical microscope equipped with a digital camera. In chronoamperometry, students examined the time dependence of current decay and diffusion-layer growth, then calculated the diffusion coefficient of the methyl viologen radical cation (MV^+ •^). In cyclic voltammetry, students visually tracked the appearance and disappearance of the colored diffusion layer during forward and reverse potential scans. Linking these visual observations to the electrochemical responses enhanced students’ ability to comprehend the underlying processes. This activity offers a versatile and visually engaging tool for teaching electrochemistry in both introductory and advanced courses.

## Graphical abstract

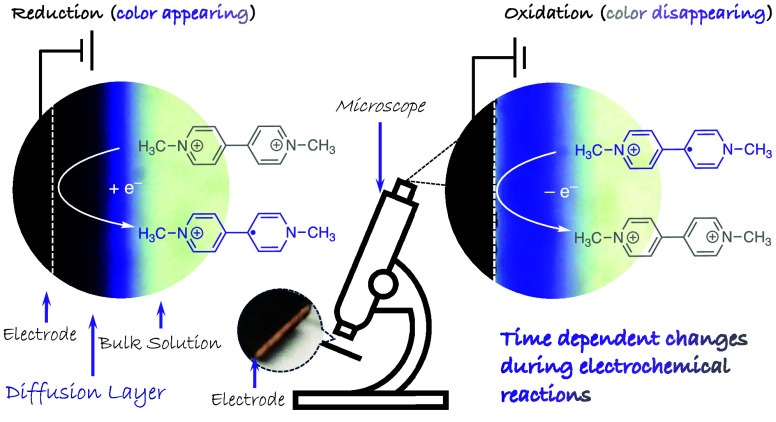

## Introduction

Diffusion is driven by concentration gradients, which are differences in the concentration of ions or molecules between two regions that cause species to move from areas of higher concentration to lower concentration.^1^ Electrode reactions provide one of the most controlled means for generating such gradients.^2^ The diffusion layer, a thin region adjacent to the electrode surface, forms through electron transfer at the electrode and mass transport to and from the bulk solution.^2,3^ Diffusion, in turn, governs the evolution of the gradients and serves as the sole mass transport mechanism in techniques such as cyclic voltammetry (CV) and chronoamperometry (CA).^4,5^ The thickness of the diffusion layer (denoted here as Δ), and the distance a molecule travels by diffusion, are determined by the diffusion coefficient and time. Understanding the time dependence of diffusion-layer growth and the resulting current response in electroanalytical techniques is therefore fundamental to understanding electrochemistry.

Visualization plays an important role in chemistry education by helping students connect observable phenomena with underlying processes. Prior work has shown that visual approaches can enhance understanding by linking measurable signals to dynamic chemical processes.^6–10^ Such approaches support the development of mental models by connecting macroscopic observations with abstract concepts. In this work, real-time color changes provide a direct visual link to electrochemical processes, facilitating conceptual understanding. We recently developed an educational experiment using a simple optical microscope with a digital eyepiece camera to observe and record the formation and growth of the diffusion layer.^11^ In that experiment, water reduction generated hydroxide ions (OH^–^), which turned the phenolphthalein indicator pink. This setup allowed students to track the time-dependent expansion of the colored diffusion layer and calculate the diffusion coefficient (*D*) of the electrochemically generated OH^–^ ions. It also provided a hands-on opportunity to discuss concepts such as the diffusion layer, diffusion coefficient, and the concentration profile of OH^–^ near the electrode. However, because water served as the solvent, a typical chronoamperometric current–time response was not obtained. Moreover, the observed color change was not a direct product of the electrode reaction but an indirect response of the pH indicator to the electrochemically generated base (Figure 1a).

**Figure 1:**
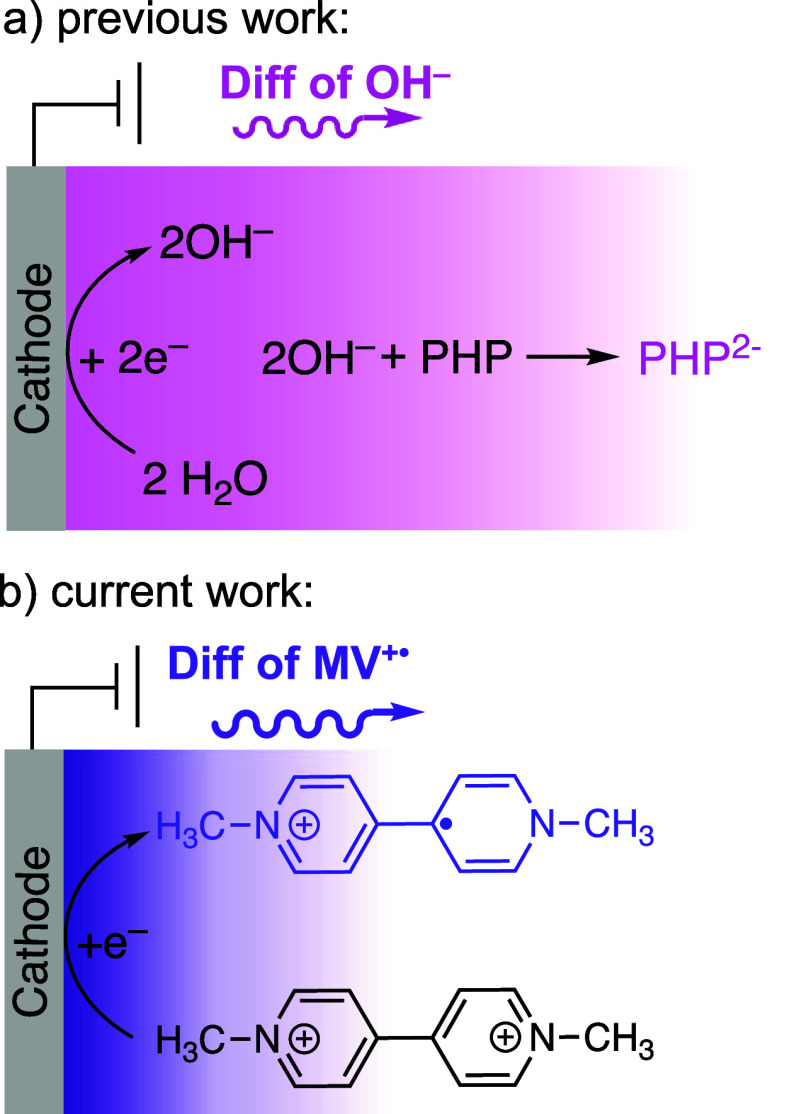
Formation of the colored diffusion layer by (a) electrochemical generation of OH^–^ in the presence of phenolphthalein (PHP) and (b) reduction of methyl viologen (MV^2+^).

In the present work, we used the same experimental setup and advanced this approach by making the electrochemical process directly observable. Using methyl viologen (MV^2+^) as a redox indicator enabled direct visualization of electroactive species undergoing electrode reactions (Figure 1b). The guiding concept of this experiment is “Electrode reaction = color change”. This direct coupling between the redox process and visible color formation provides a more realistic representation of the diffusion layer and allows students to correlate its evolution with chronoamperometric current–time data. Furthermore, the reversible redox chemistry of MV^2+^ enables students to observe both the appearance and disappearance of color during the forward and reverse scans of CV, clearly illustrating the dynamic relationship between potential, current, and concentration in this widely used electrochemical technique.

## SCIENTIFIC BACKGROUND AND OVERVIEW

### Indicator

*N*,*N*′-Dimethyl-4,4′-bipyridinium dichloride, known as methyl viologen (MV^2+^), is widely used as a redox indicator and electrochromic material, due to its distinct color change during redox reactions.^12–16^ It is an excellent candidate for visualizing the diffusion layer through direct electron transfer. The oxidized form, MV^2+^, is colorless in solution. A one-electron reduction of MV^2+^ produces the radical cation (MV^+•^), which has a deep violet-blue color. Further reduction of MV^+ •^ to the neutral species (MV) yields a colorless or pale-yellow product. However, this step is uncommon in electrochemical applications.^17^ The redox reaction of MV^2+/^MV^+ •^ (Figure 2) is the focus of this activity.

**Figure 2:**
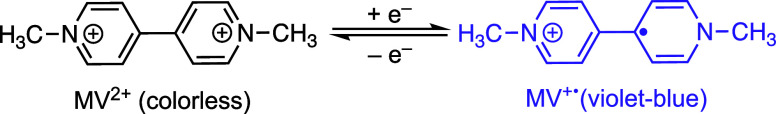
Redox reaction of methyl viologen to its cation radical.

### Chronoamperometry

Chronoamperometry (CA) is a standard technique for investigating electrochemical reactions.^18–22^ In this method, the electrode potential is stepped from a value at which no electron transfer occurs to one at which a faradaic process takes place, and the resulting current is recorded as a function of time. The resulting current–time profile is called a chronoamperogram (Figure 3). The measured current includes both faradaic contributions from electron transfer reactions and nonfaradaic (charging) current associated with double-layer charging. The charging current is typically significant only during the first 1–2 s of the experiment.^18^ When a sufficiently large overpotential is applied, the current is determined by the flux of redox-active species to the electrode surface. In a quiescent solution, mass transport occurs predominantly via diffusion, which is described by the Cottrell equation (eq 1):^23^

1 $$I=nFA\pi -\frac{1}{2}{C_{A}D}_{A}^{1/2}t^{-1/2}$$

**Figure 3:**
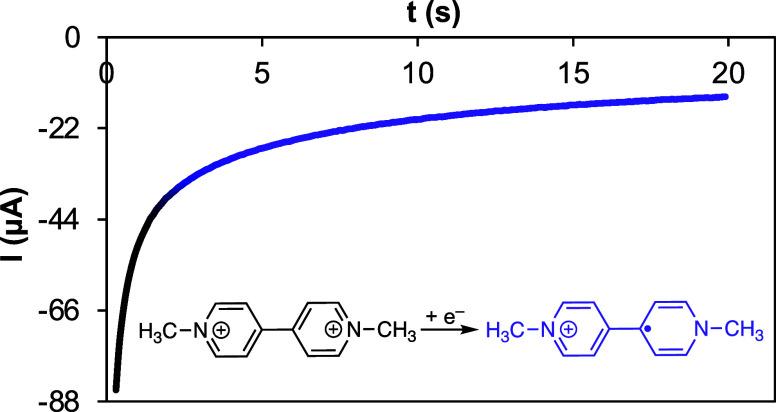
Chronoamperogram of reduction of methyl viologen, reaction conditions: 5 mM methyl viologen in aqueous solution; supporting electrolyte, 0.05 M NaCl; applied potential, −0.7 V.

In this equation, *I* is the current (A), *n* is the number of electrons transferred, *F* is the Faraday constant (96,485 C mol^–1^), *A* is the electrode area (cm^2^), *C*_*A*_ is the bulk concentration (M) of the electroactive species (mol cm^–3^), *D*_*A*_ is the diffusion coefficient (cm^2^ s^–1^), and *t* is time (s). The time dependence of current and its relationship to diffusion-layer growth are the key concepts explored in this activity.

### Cyclic Voltammetry

CV is one of the most widely employed electrochemical techniques for investigating electrode processes and determining the redox properties of electroactive species.^24,25^ In this method, the potential is swept linearly with time, starting from an initial value (*E*_i_), passing through the formal redox potential (*E*°′), to a switching potential (*E*_s_). At *E*_s_, the scan direction is reversed, and the potential is swept back to a final value (*E*_f_), typically equal to *E*_i_. For a solution containing the oxidized form (i.e., MV^2+^), reduction begins as the potential is scanned to values near *E*°′, generating a cathodic current. At the electrode surface, the relative concentrations of the oxidized and reduced species are described by the Nernst equation and vary with the applied potential. As the potential becomes more negative than E°’, the surface concentration of MV^2+^ decreases, leading to the maximum peak current. Beyond this peak potential, the surface concentration of MV^2+^ approaches zero, and the expansion of the diffusion layer causes a decrease in the concentration gradient and a subsequent decay in current. On the reverse scan, as the potential is swept back to more positive values, the electrochemically generated radical cation (MV^+ •^) is oxidized back to MV^2+^, producing an anodic peak with a similar shape to the cathodic peak, but in the opposite direction. The cyclic voltammogram of MV^2+^, together with the associated reactions, is illustrated in Figure 4. The reversible redox couple MV^2+^/MV^+ •^, with its observable color change at the electrode surface, is used to visualize the temporal formation and decay of the diffusion layer during the voltammetric experiment.

**Figure 4:**
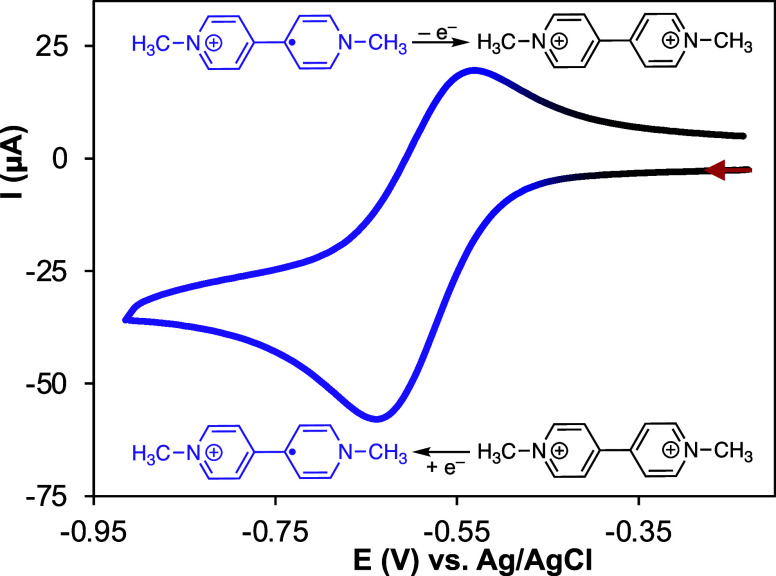
Cyclic voltammogram of reduction of methyl viologen, reaction conditions: 5 mM methyl viologen in aqueous solution, supporting electrolyte 0.05 M NaCl, scan rate 50 mV/s. (The arrow shows where the scan begins and the scan direction.)

### Experimental Procedure

The thin-layer electrochemical cell described in our previous work,^11^ was adapted to a three-electrode configuration (working, counter, and reference electrodes) for this study. Detailed fabrication procedures and step-by-step experimental protocols are provided (see the ). Voltammetric and amperometric measurements were conducted to monitor the reduction and oxidation of MV^2+^/MV^+ •^. Real-time color changes at the electrode surface were recorded using a microscope equipped with a digital camera, and diffusion-layer thicknesses were determined from the recorded images as a function of time.

### Hazards

Goggles and gloves should always be worn during this experiment. Methyl Viologen (MV^2+^) is a commonly used herbicide that is toxic through ingestion, inhalation, and dermal contact. Rinse eyes and skin if exposed, collect spillage, and avoid release to the environment.^26^ Remove personal protective equipment after handling this product. Proper waste disposal should be implemented after every use of this chemical during the experiment. No unexpected or unusually high safety hazards were encountered.

## Results and Discussion

The following results represent the evolution of the diffusion layer observed during CA and CV experiments, together with the corresponding discussion.

### Chronoamperometry

The CA experiment was performed at −0.70 V for 20 s while monitoring the solution using a microscope equipped with a digital camera. At this potential, the colorless MV^2+^ species is fully reduced to the violet-blue MV^+ •^ species, producing visually distinct color changes that expand over time (Figure 5a–d).

**Figure 5:**
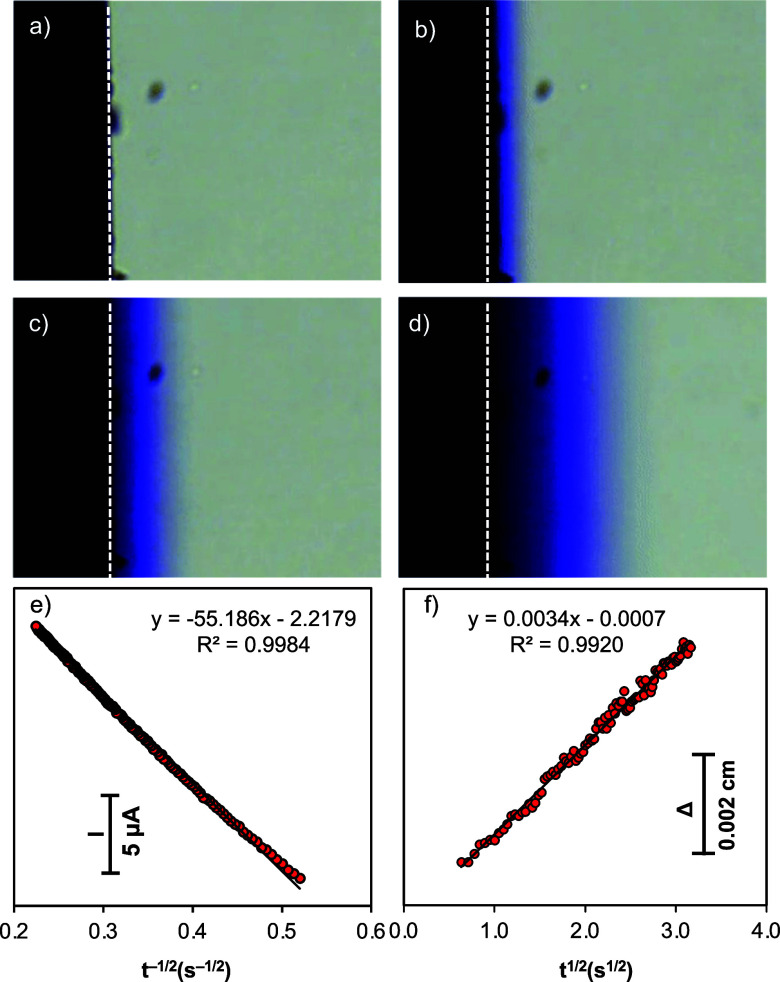
(a–d) Time-dependent progression of the diffusion layer during the chronoamperometric reduction of MV^2+^ to violet-blue MV^+ •^ at indicated times. (e) Plot of current versus *t* ^–1/2^. (f) Plot of thickness of the diffusion layer (Δ) versus *t* ^1/2^. All data correspond to the chronoamperometric experiment shown in Figure 3.

The resulting chronoamperogram (current–time plot), shown in Figure 3, exhibits the decay behavior described by the Cottrell equation (eq 1). Consequently, when current is plotted versus *t* ^–1/2^, a linear relationship is observed, as illustrated in Figure 5e. To relate the decay in current to changes in the diffusion layer, the time-dependent growth of the diffusion layer was also examined. The Δ (in cm) can be estimated using eq 2:^27^

2 $$\Delta ={\left(2D\right)}^{1/2}t^{1/2}$$

where *D* is the diffusion coefficient (cm^2^ s^–1^) and *t* is time (s). Students used time frames extracted from the recorded video (see the ) to plot Δ versus *t* ^1/2^. The Δ value was determined by measuring the distance from the electrode surface to the border between the faintly colored edge and the colorless region, corresponding to the area where the concentration closely matches that of the bulk solution. This boundary was identified visually. A template Excel file (see the ) along with an example completed by a student (see the ), is provided. The resulting plots showed a linear trend, as illustrated in Figure 5f, and the slope of this plot corresponds to (2*D*)^1/2^, from which the diffusion coefficient (*D*) can be calculated. For example, the data in Figure 5 yielded a slope of 0.0034, corresponding to a diffusion coefficient of (5.28 ± 0.42) × 10^–6^ cm^2^ s^–1^, which agrees well with reported literature values for methyl viologen.^28,29^ Statistical analysis, including the use of *t* test, could be applied to compare results across classrooms and with values reported in the literature; however, this is not discussed in the present paper. Although these values correspond to MV^+ •^, the diffusion coefficient of MV^2+^ is reported to be similar. More importantly, as a key learning point, the plots in Figure 5e and 5f illustrate that, while the diffusion layer grows proportionally to *t* ^1/2^, the current simultaneously decays proportionally to *t* ^–1/2^. The diffusion coefficient can also be estimated from the slope of *I* vs *t* ^–1/2^, using the Cottrell equation. For the homemade copper electrode, the calculated *D* value deviated by a factor of 2–3 from the literature, likely due to electrode surface roughness and solution access near the heat-shrink tubing. However, using a commercial glassy carbon disk electrode produced a *D* value consistent with both literature values and the diffusion-layer analysis. This demonstration highlights the direct relationship between diffusion-layer expansion and the time-dependent current in CA, reinforcing the connection between theoretical predictions and observable experimental behavior.

### Comparing the Diffusion Coefficient of MV^+ •^ with OH^–^

By determining the diffusion coefficient of MV^+ •^ in this activity, we took the opportunity to compare it with that of OH^–^, reported previously using a similar color-change method at the electrode surface. While both values are consistent with literature reports, the diffusion coefficient for OH^–^ (2.41 × 10^–5^ cm^2^ s^–1^) was about four to five times larger than that of MV^+ •^. This notable difference reflects the unique nature of hydroxide transport in water. The higher *D* value and faster diffusion of OH^–^, compared with other molecular or ionic species, result from a structural diffusion process known as the Grotthuss mechanism.^30^ In this process, OH^–^ transport does not rely solely on the physical motion of individual ions through the solvent. Instead, OH^–^ moves by a relay-like exchange across the hydrogen-bond network of water molecules, effectively hopping from one molecule to the next. This difference in how OH^–^ moves, compared with the slower diffusion of MV^+ •^, gives students a clear example of how molecular size and transport mechanisms affect diffusion rates.

### Cyclic Voltammetry

The CV experiment was also implemented in this activity to visualize the formation and disappearance of the colored diffusion layer associated with the MV^2+^/MV^+ •^ redox couple. During the forward scan, MV^2+^ is reduced to the violet-blue MV^+ •^ radical cation, and on the reverse scan MV^+ •^ is oxidized back to the colorless MV^2+^. The continuous potential sweep introduces additional complexity compared to CA, making it difficult to quantify the color changes or directly correlate them with current response. Nevertheless, the visual observations provide an opportunity to connect distinct features of the voltammogram to observable color changes.

Figure 6 presents the current–time response of the CV experiment, with selected points highlighted and their corresponding time frames shown.

**Figure 6:**
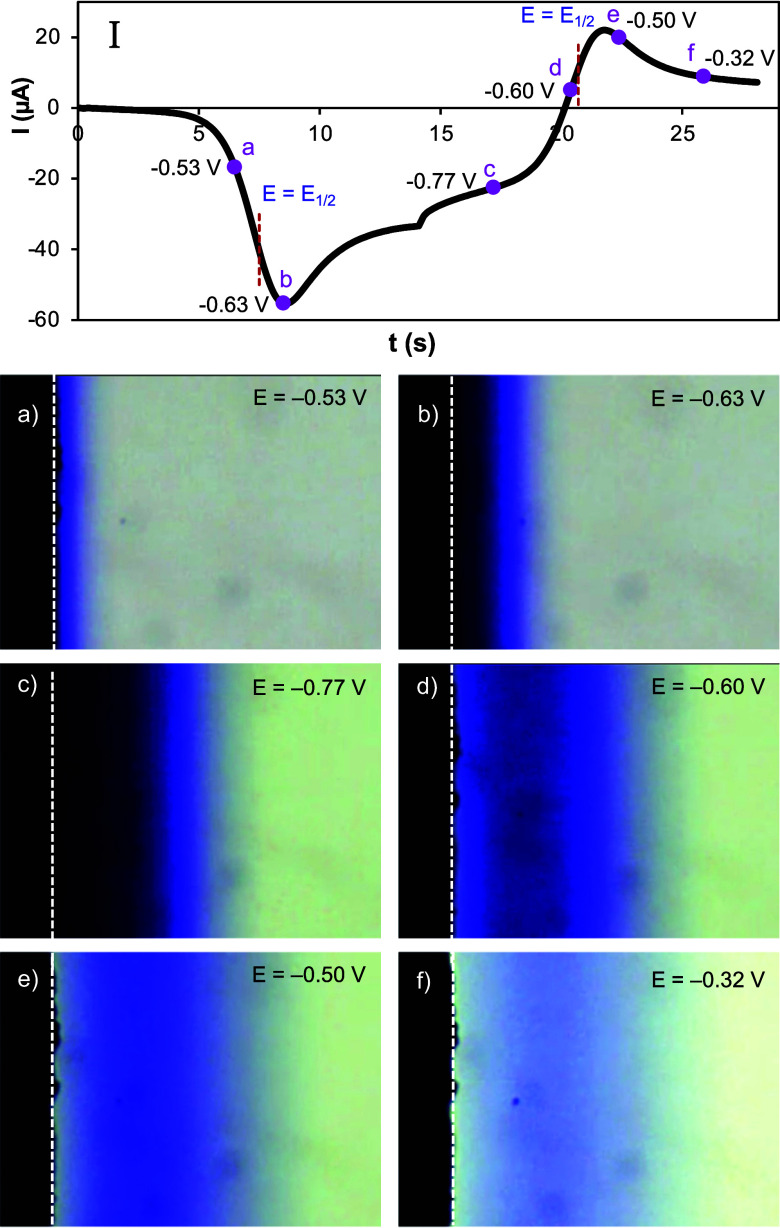
(I) Current–time response corresponding to the cyclic voltammogram presented in Figure 3, with selected points (a–f) highlighted by red circles and their corresponding potentials. Panels (a–f) show the respective time frames for the highlighted points in (I).

The electrode processes and visual changes can be summarized as follows:

- The reduction of MV^2+^ to MV^+ •^ begins at potentials slightly more positive than *E*_1/2_ (point “a” in Figure 6). Because the initial concentration of MV^+ •^ is zero, electron transfer proceeds to establish equilibrium at the electrode surface as described by the Nernst equation.
- At the peak potential (point “b” in Figure 6), which occurs at potentials more negative than *E*_1/2_, the current reaches its maximum as most MV^2+^ near the electrode surface is converted to MV^+ •^. At this point, the surface concentration of MV^2+^ is nearly depleted, and the diffusion layer has not yet extended much, resulting in the maximum concentration gradient.
- At potentials more negative than the peak potential (point “c” in Figure 6), the concentration of MV^2+^ at the electrode surface approaches zero, while the concentration of MV^+ •^ reaches its maximum. The overall color intensity does not increase noticeably beyond the reduction peak, but the diffusion layer continues to expand with time, causing the current to decay after the peak. After the scan reversal and before the potential approaches *E*_1/2_ on the positive-going sweep, the main process is still reduction, with the dominant effect being further expansion of the diffusion layer.
- As the potential reaches and passes *E*_1/2_ in the positive direction, the electrochemically generated MV^+ •^ is oxidized back to MV^2+^ (points “d” and “e” in Figure 6). A clearly observable feature is the rapid fading and disappearance of the blue color near the electrode, which makes the reverse reaction visually obvious, accompanied by the appearance of anodic current.
- At more positive potentials, MV^+ •^ species farther from the electrode diffuse back and are oxidized to MV^2+^. This causes the colored diffusion layer to fade progressively, until the region near the electrode becomes almost completely colorless (point “f” in Figure 6).

Overall, it is important to note that all the observed changes occur within a very thin layer, less than 0.02 cm thick. For a typical voltammetric experiment utilizing a disk electrode with a diameter of 0.3 cm (0.07 cm^2^ surface area), the total solution volume affected by the electrode reaction is only about 0.0014 mL. This is negligible compared to the bulk solution (usually 10–25 mL), which explains why electroanalytical experiments like CV have almost no effect on the overall concentration. If the solution is stirred, the system effectively returns to its initial state, allowing the experiment to be repeated multiple times with the same solution.

## IMPLEMENTATION AND PEDAGOGICAL CONTEXT

This experiment was initially developed with the help of two undergraduate research students and was later repeated by three additional students who became involved in electrochemistry research projects. It is performed as a hands-on experiment for students interested in undergraduate electrochemistry research. For classroom implementation, the activity was conducted in an Analytical Chemistry course primarily enrolling third-year chemistry, biology, premedical, and predental students. The class size typically ranged from 40 to 48 students; in the present semester, 44 students participated. Laboratory sessions were divided into two groups of approximately 20–25 students each. The total time required for the presentation, demonstration, and surveys was approximately 1 h. The activity was implemented with students who had not been formally introduced to CV or CA in their courses. Although students had previously used voltammetry in a vitamin C experiment,^31^ it was applied primarily as a technique to measure peak current as a function of ascorbic acid concentration, without emphasis on the underlying electrochemical principles. Therefore, a 30 min presentation was first delivered to compare chemical redox reactions with electrochemical reactions and to introduce the fundamental concepts of CV and CA (see the ). Basic thermodynamic and kinetic concepts relevant to CV, such as the relationship between redox potentials and molecular orbital energetics, as well as the influence of follow-up chemical steps on peak shapes, were also briefly reviewed to provide context for the activity,^19,32^ PowerPoint slides from the presentations are provided in the ). Following this introduction, the preactivity survey was administered to assess baseline understanding (see the ). The electrochemical setup, along with methyl viologen and its redox reaction, was then introduced, and students observed the demonstration and real-time recordings of the experiments. A post-activity survey was subsequently conducted to evaluate learning outcomes (see the ). Finally, students were assigned a take-home exercise in which they analyzed the time-dependent growth of the diffusion layer and determined the diffusion coefficient using the provided video recordings and PDF files (see the ).

Survey results were evaluated descriptively by comparing the number of students selecting correct responses in the pre- and post-activity surveys. Comparison of the pre- and post-activity surveys suggests improved conceptual understanding of electrochemical processes among students. For the question related to diffusion-controlled behavior in CA, the number of students selecting the correct responses increased from 26 in the pre-survey to 37 in the post-survey. Similarly, for the question addressing the role of forward and reverse scans in CV, correct responses increased from 28 to 41 students. Students’ ability to interpret electrochemical plots also showed a positive trend, with the number reporting that they could interpret current–time and current–potential relationships “very well” or “somewhat well”, increasing from 23 before the activity to 37 after the demonstration. In addition, responses indicated a greater ability to connect the observed color changes of methyl viologen to concentration gradients and diffusion-layer growth near the electrode. Responses to the open-ended question further supported these findings: students reported that the activity helped them visualize processes at the electrode surface (5 responses), understand the role of mass transfer in electrochemistry (8 responses), recognize how electricity can cause chemical change (4 responses), and improve their overall understanding of electrochemical reactions (7 responses). Student performance on the take-home exercise was also evaluated. Among 44 students who completed the assignment, the majority were able to successfully determine the diffusion-layer thickness and calculate the diffusion coefficient. Specifically, 37 students obtained values within an acceptable range consistent with expected results. Four students produced linear plots but reported diffusion coefficient values that differed by more than a factor of 3 from expected values, while three students made significant errors (e.g., unit conversion or calibration issues in PDF-based measurements), leading to incorrect results. For the 37 students with acceptable results, the calculated diffusion coefficients ranged from 4.54 to 5.92, with an average of 5.21 and a standard deviation of 0.68. Students received written feedback on their diffusion-layer analysis following submission. These results indicate that most students were able to successfully complete the analysis, with observed errors primarily related to data handling rather than conceptual understanding. Overall, the activity, survey results, and student responses suggest enhanced conceptual understanding and strong student interest in the visual–electrochemical learning approach.

The student surveys used in this study were reviewed by the University of Missouri–Kansas City Institutional Review Board and determined to qualify for exempt review under 45 CFR 46.104­(d)(2)­(i) (IRB Project No. 2134892).

## Conclusion

This activity provides a powerful way to visualize electrochemical processes by combining redox indicator reactions with real-time microscopic observation. Compared with our previous approach using water reduction, this method offers a more direct and quantitative connection between diffusion-layer growth and chronoamperometric behavior. Using a redox indicator at a limited concentration, rather than water as a solvent, allows the chronoamperometric response to be described quantitatively and directly correlated with the observed color changes at the electrode surface. The activity enables students to explore the time dependence of both current decay and diffusion-layer growth, and to calculate the diffusion coefficient of MV^+ •^. This transforms the abstract theoretical concepts of mass transfer in electrochemistry into observable phenomena. By visually tracking the formation and decay of the colored diffusion layer during voltammetry, students can gain an intuitive understanding of the reactions occurring during the forward and backward potential scans. This visualization can reinforce theoretical concepts and provide a conceptual framework for teaching and learning this widely used electrochemical technique. The activity can function as a standalone experiment or complement existing electrochemistry curricula, and it is well-suited for introductory chemistry courses as well as more advanced analytical, physical, or instrumental analysis courses, offering a versatile and visually compelling tool for teaching both the theory and practice of electrochemistry.

## Associated Content

Two recorded videos of the expansion of the diffusion layer were accessed: one during CA and one during cyclic voltammetry. These videos are available on YouTube.^33,34^

## Supplementary Material



















## Notes

Analysis of the diffusion layer thickness during chronoamperometry experiment, showing an example of the plot and the obtained diffusion coefficient (XLSX)
Blank Microsoft Excel file to enter the values of diffusion layer thickness for deriving the diffusion coefficient (XLSX)
Chem vs EChem Redox Reactions.pptx: PowerPoint presentation including chemical and electrochemical redox reactions and the fundamental concepts of cyclic voltammetry (CV) and chronoamperometry (CA) (PDF)
Experimental and Instruction including the experimental details and the procedure for analysis of the recorded time frames using Adobe Acrobat Reader (DOCX) and its PDF version (PDF)
10 FPS file derived from the video recording of the chronoamperometric experiment of methyl viologen (GIF)
Images extracted from the video recording of the chronoamperometric experiment of methyl viologen at 0.1 s intervals (PDF)
Post-Activity Survey, containing the student surveys administered after the demonstration (PDF)
Pre-activity survey, containing the student surveys administered before the demonstration (PDF)
