# Supplementary material for: Thin-Layer Electrochemistry: Visualizing the Diffusion Layer During Chronoamperometry and Cyclic Voltammetry
Source: J Chem Educ. 2026 Jul 16;103(8):4704–10. doi: 10.1021/acs.jchemed.5c01710 (PMC13472089; doi:10.1021/acs.jchemed.5c01710)
Supplement: Supplementary file 1 [file ed5c01710_si_003.pdf]

Supporting Information

Thin-Layer Electrochemistry: Visualizing the Diffusion Layer During Chronoamperometry and Cyclic Voltammetry

Mohammad Rafiee[a*], Lida Khalafi[b], Keya Pandey[a], Mary Gipson[a]

[a] Division of Energy, Matter and Systems, School of Science and Engineering, University of Missouri−Kansas City, Kansas City, Missouri 64110, United States

[b] College of Arts and Sciences, Avila University, Kansas City, Missouri 64145, United States

* Mohammad Rafiee: mrafiee@umkc.edu

Note: These slides were developed for use alongside guided discussion and chalkboard instruction and are not intended to serve as a comprehensive or standalone treatment of electrochemical reactions. They are provided as Supporting Information to supply illustrative material that may assist instructors in presenting electrochemical concepts in undergraduate chemistry courses.

Redox Reaction between Iodate and Iodide:

$$IO^{-}_{3(aq)} + 8I^{-}_{(aq)} + 6H^{+}_{(aq)} \rightarrow 3I^{-}_{3(aq)} + 3H_2O_{(l)}$$

Redox Reaction between Thiosulfate and Iodine

$$I^{-}_{3(aq)} + 2S_2O^{2-}_{3(aq)} \rightarrow 3I^{-}_{(aq)} + S_4O^{2-}_{6(aq)}$$

Redox Reaction between ascorbic acid and Iodine

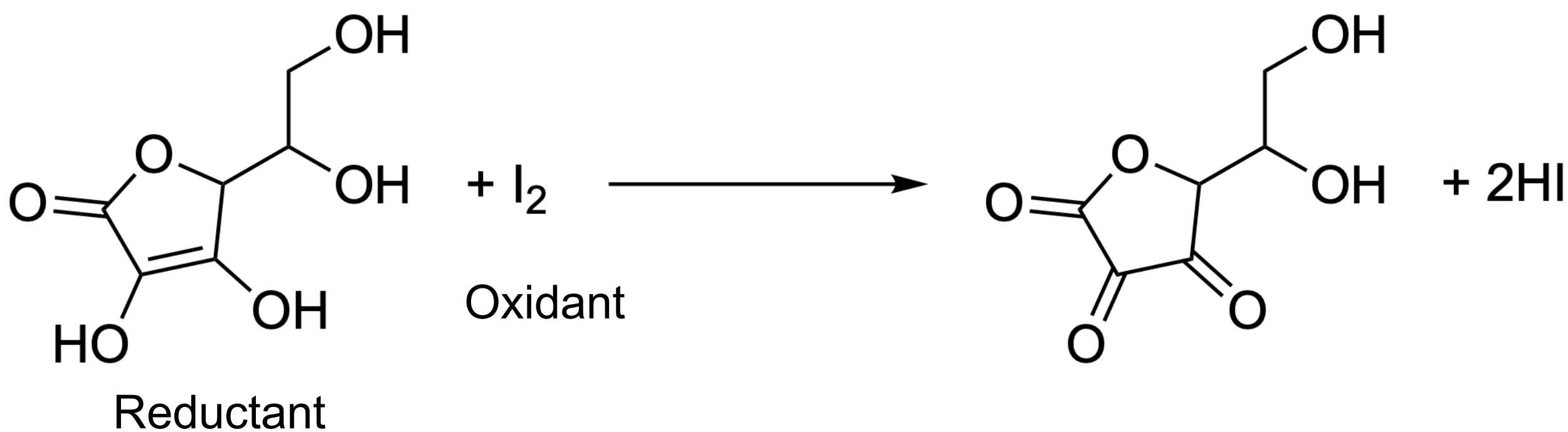

Or: $$I^{-}_{3(aq)} + H_2A_{(aq)} \rightarrow 3I^{-}_{(aq)} + D_{(aq)} + 2H^{+}_{(aq)}$$

Redox Reactions

Redox Reaction between Iodate and Iodide:

$$IO^-_{3(aq)} + 8I^-_{(aq)} + 6H^+_{(aq)} \rightarrow 3I^-_{3(aq)} + 3H_2O_{(l)}$$

Redox Reaction between Thiosulfate and Iodine

$$I^-_{3(aq)} + 2S_2O^{2-}_{3(aq)} \rightarrow 3I^-_{(aq)} + S_4O^{2-}_{6(aq)}$$

Redox Reaction between ascorbic acid and Iodine

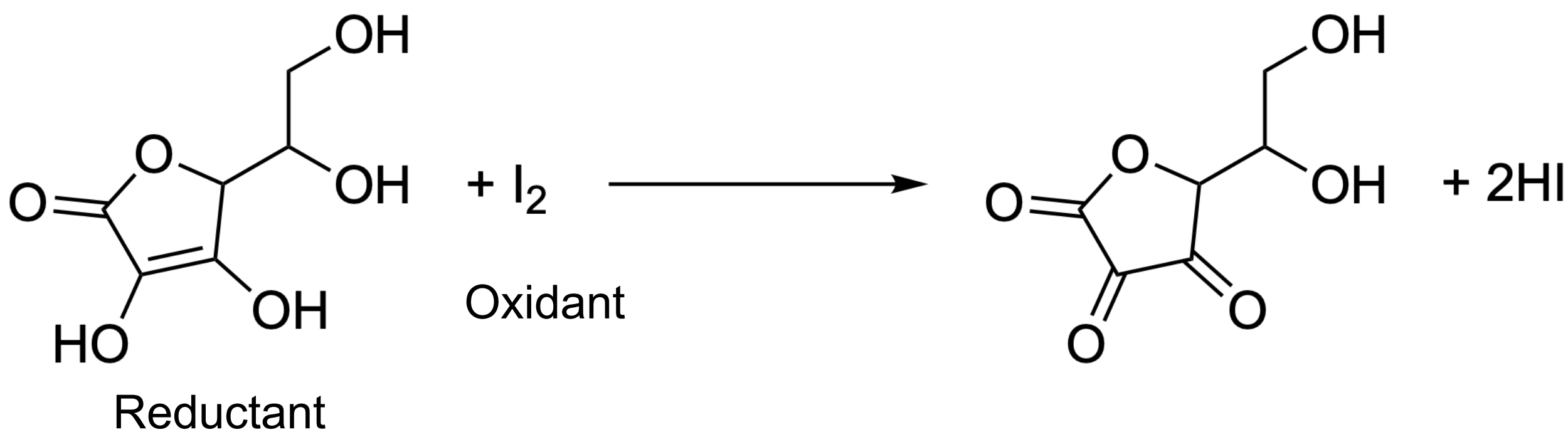

Or: $$I^-_{3(aq)} + H_2A_{(aq)} \rightarrow 3I^-_{(aq)} + D_{(aq)} + 2H^+_{(aq)}$$

Chemical vs Electrochemical Reactions

Redox Reaction

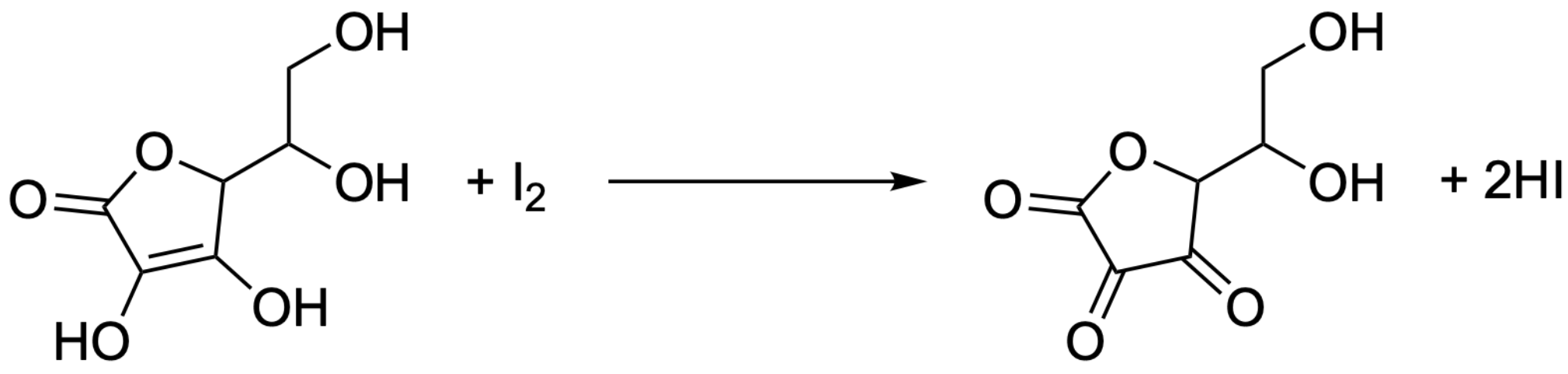

Half Reactions:

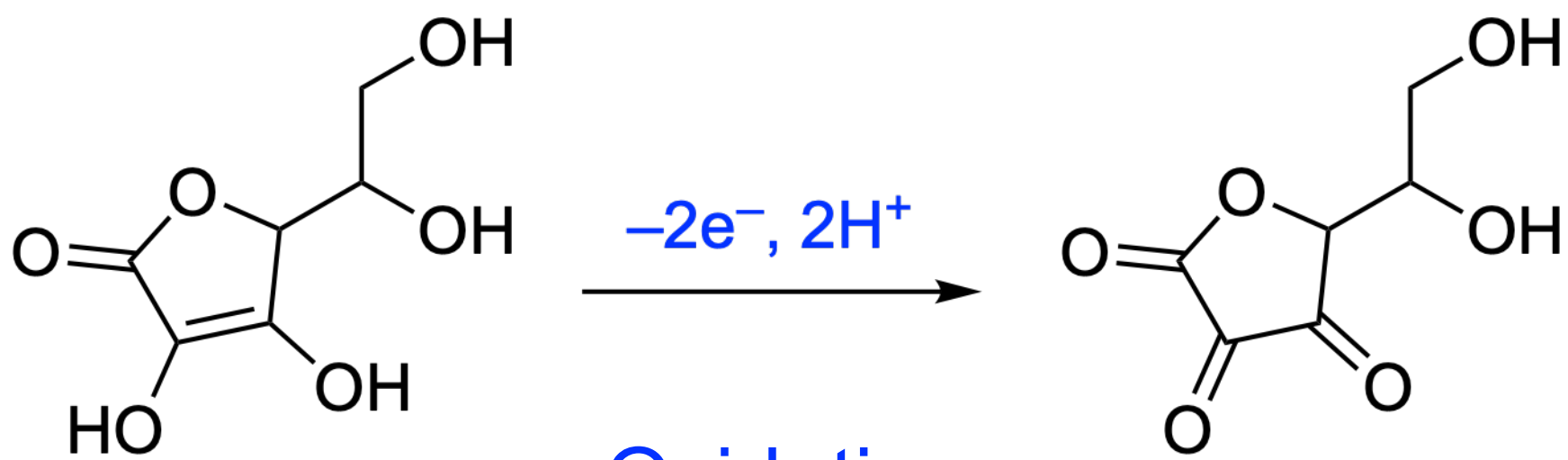

Oxidation

$$I_2 \xrightarrow{+2e^-,\ 2H^+} 2HI$$

Reduction

Electrochemical Reactions

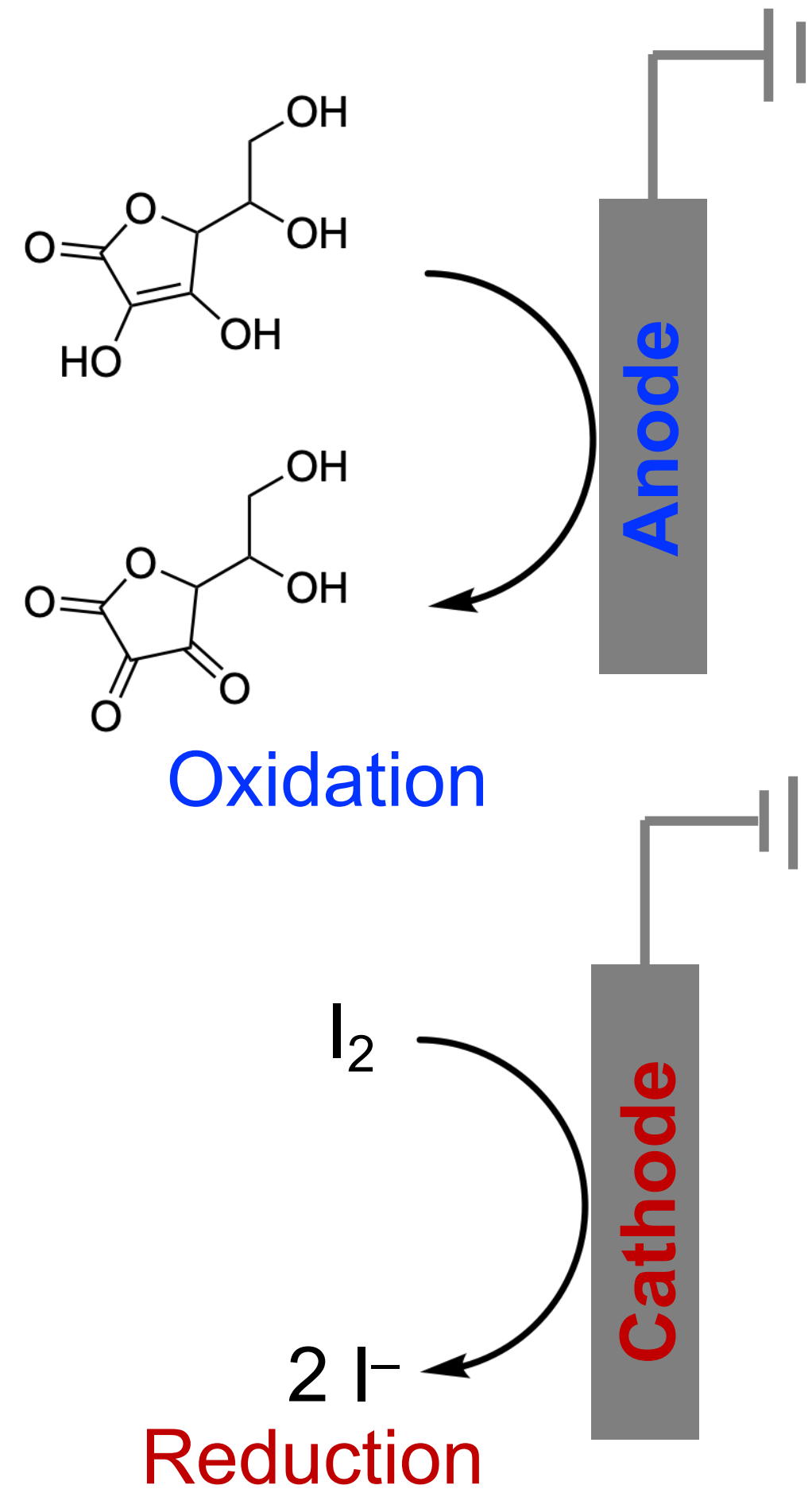

Advantages of Electrochemical Reactions

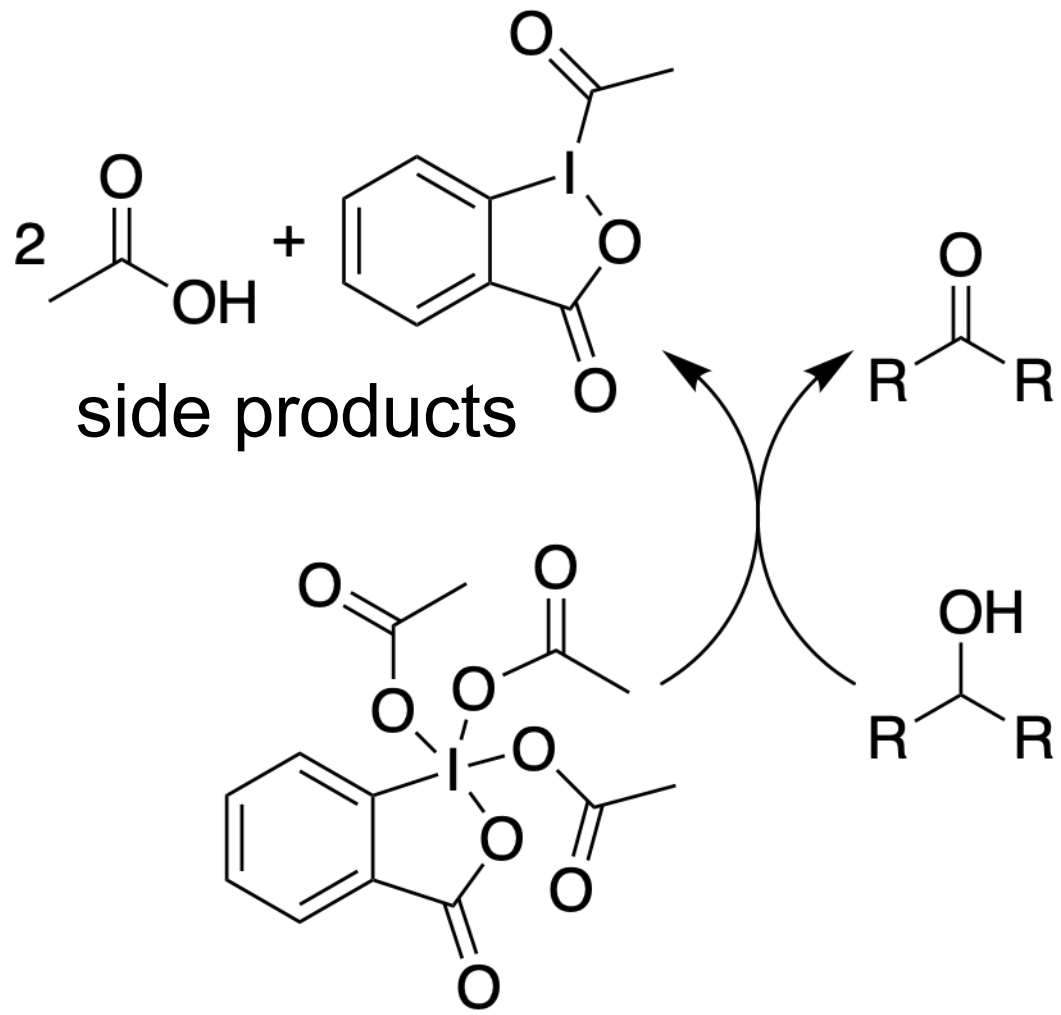

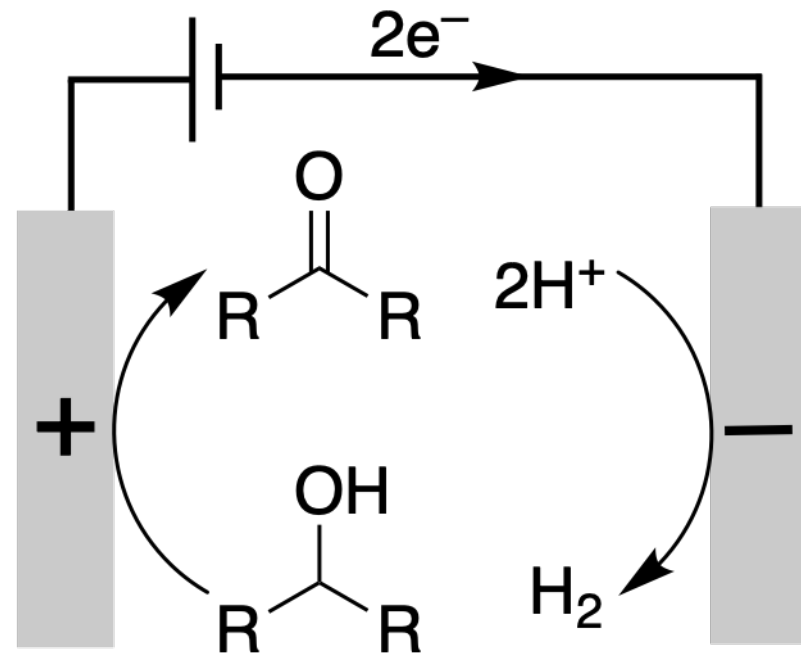

Dess, D. B.; Martin, J. C. *J. Org. Chem.* **1983**, *48*, 4155.

Kaulen, J.; Schäfer, H.-J. Tetrahedron, 38, 1982, 3299.

Advantages of electrochemistry over chemical redox reagents:

1. Avoid potentially hazardous waste from some chemical oxidants/reductants

2. Tunable
   - Chemist (not redox reagent) sets the reaction potential
   - Control rate of generation of reactive species

Cyclic Voltammetry

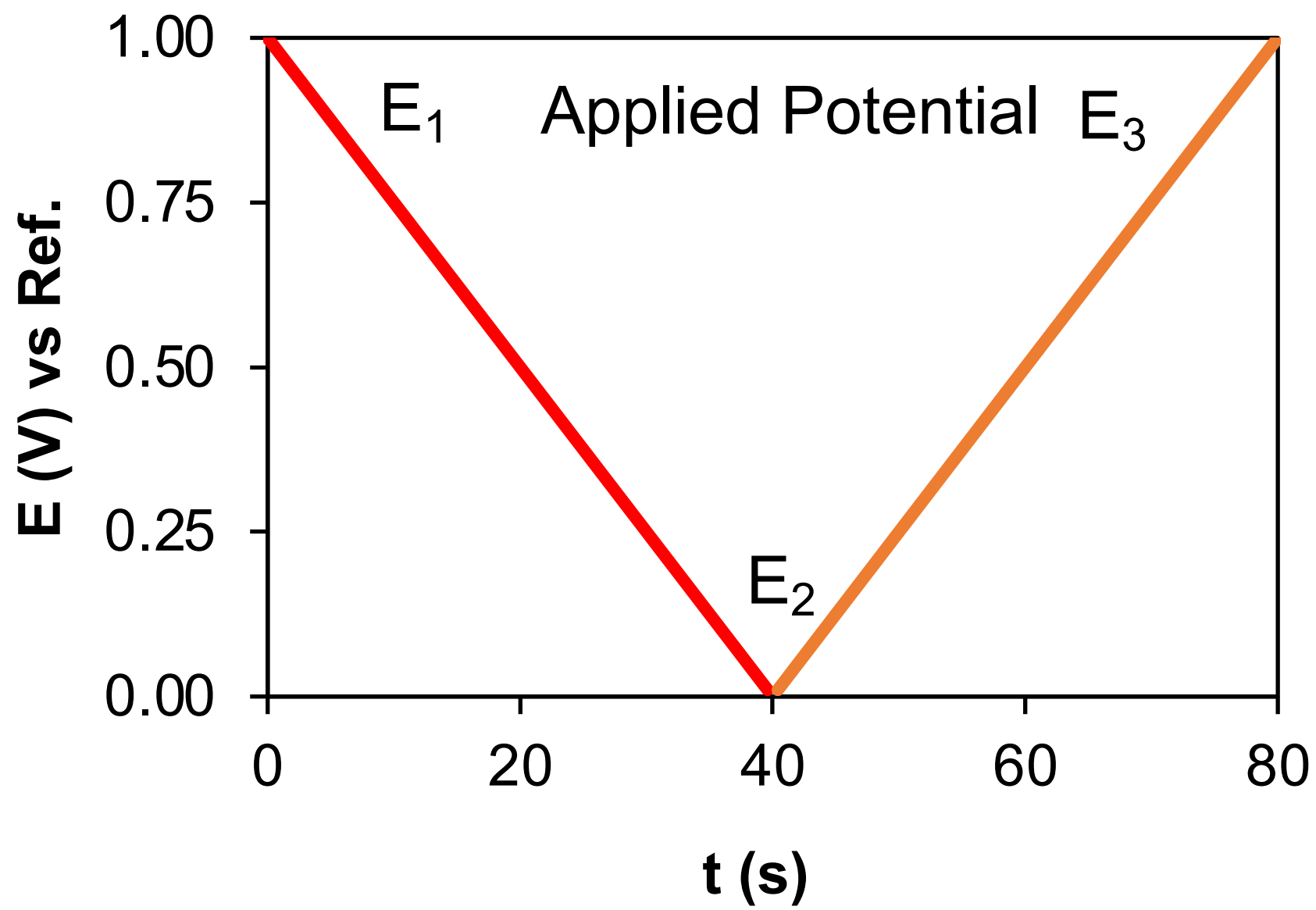

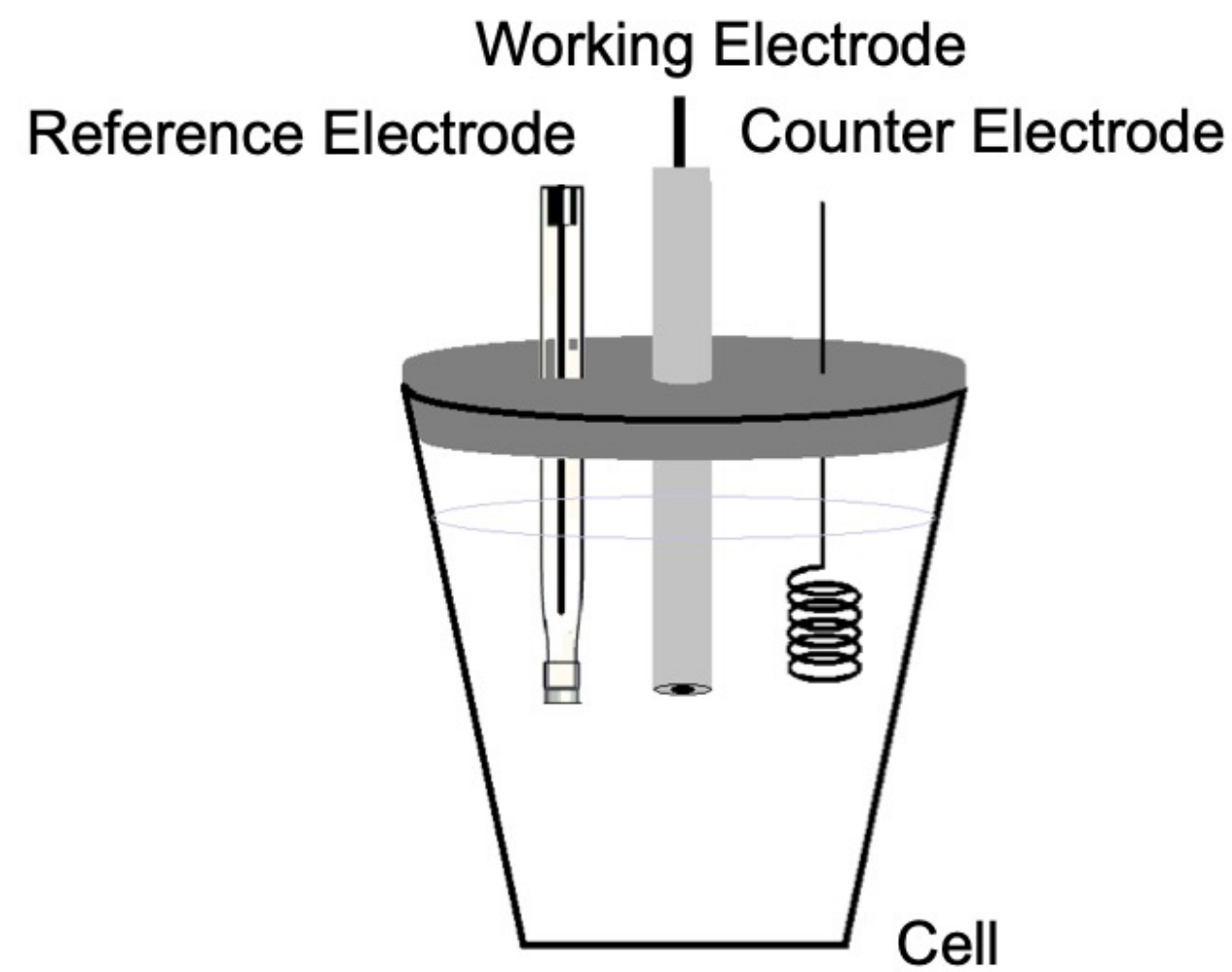

The working electrode is the electrode of interest.

The reference electrode provides a well-defined potential against which the working electrode potential is measured or scanned.

The counter electrode along with working electrode provides circuit over which current flows between the working electrode and counter electrode.

Cyclic Voltammetry

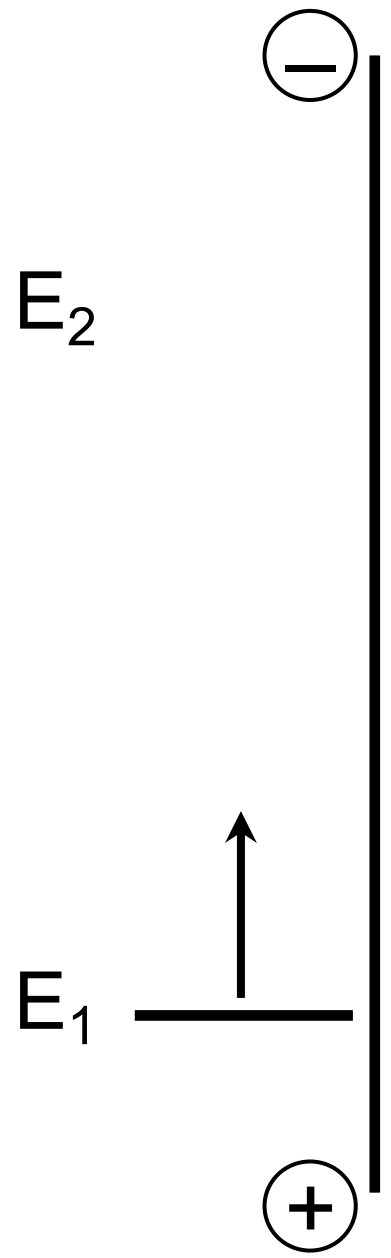

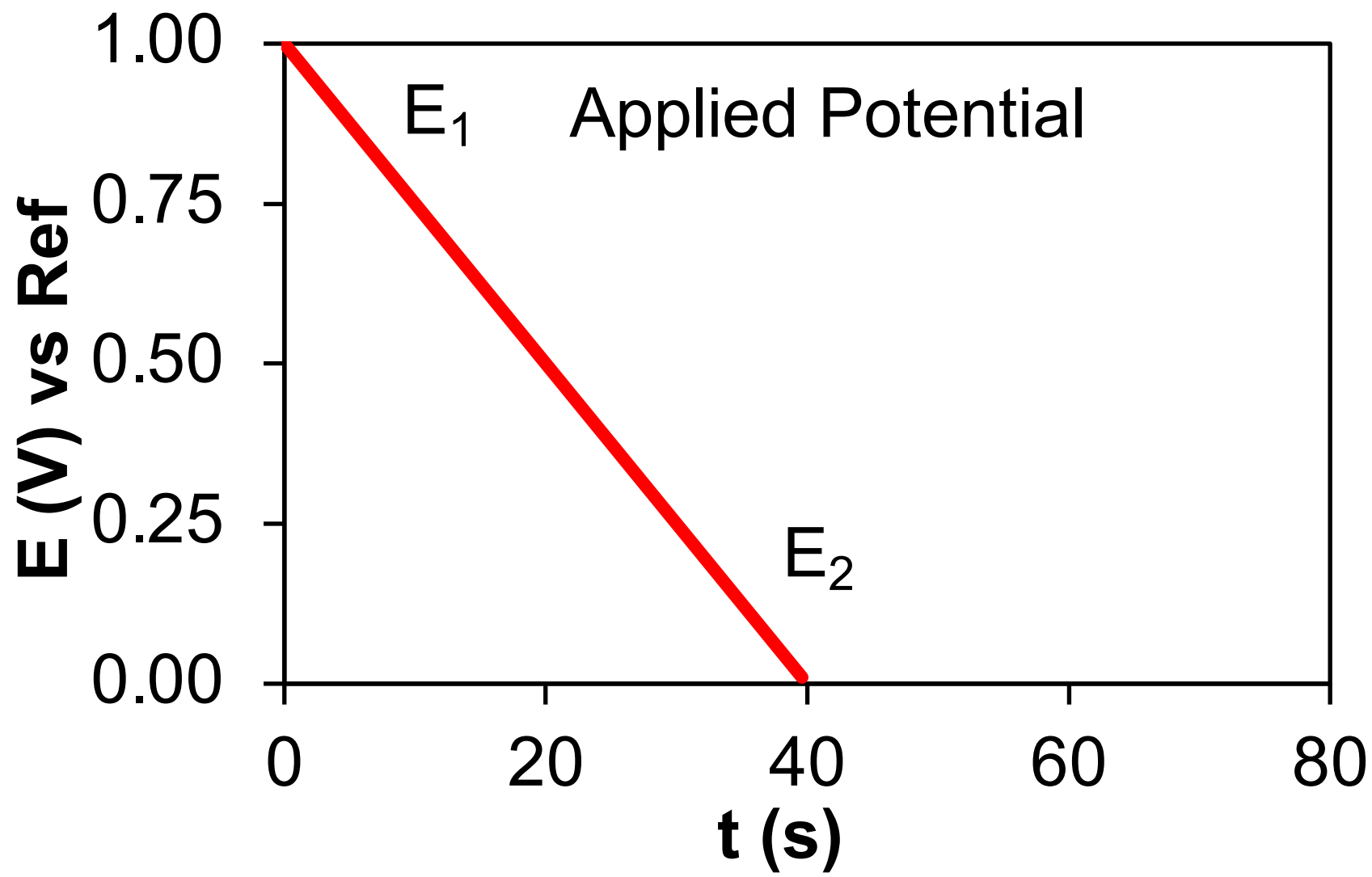

Cyclic Voltammetry

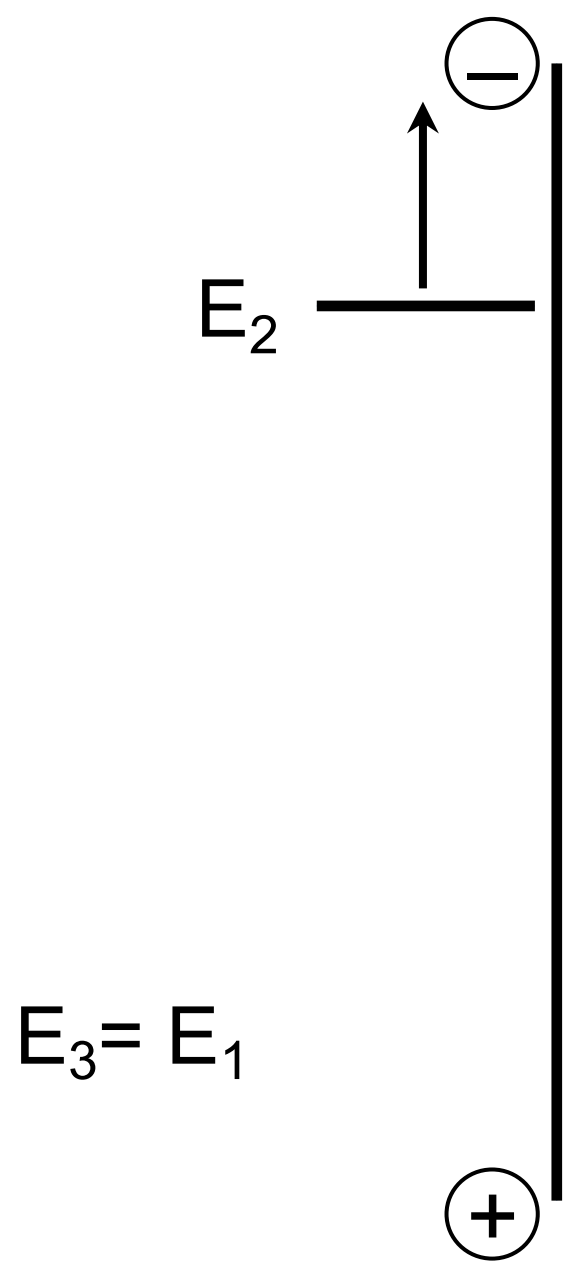

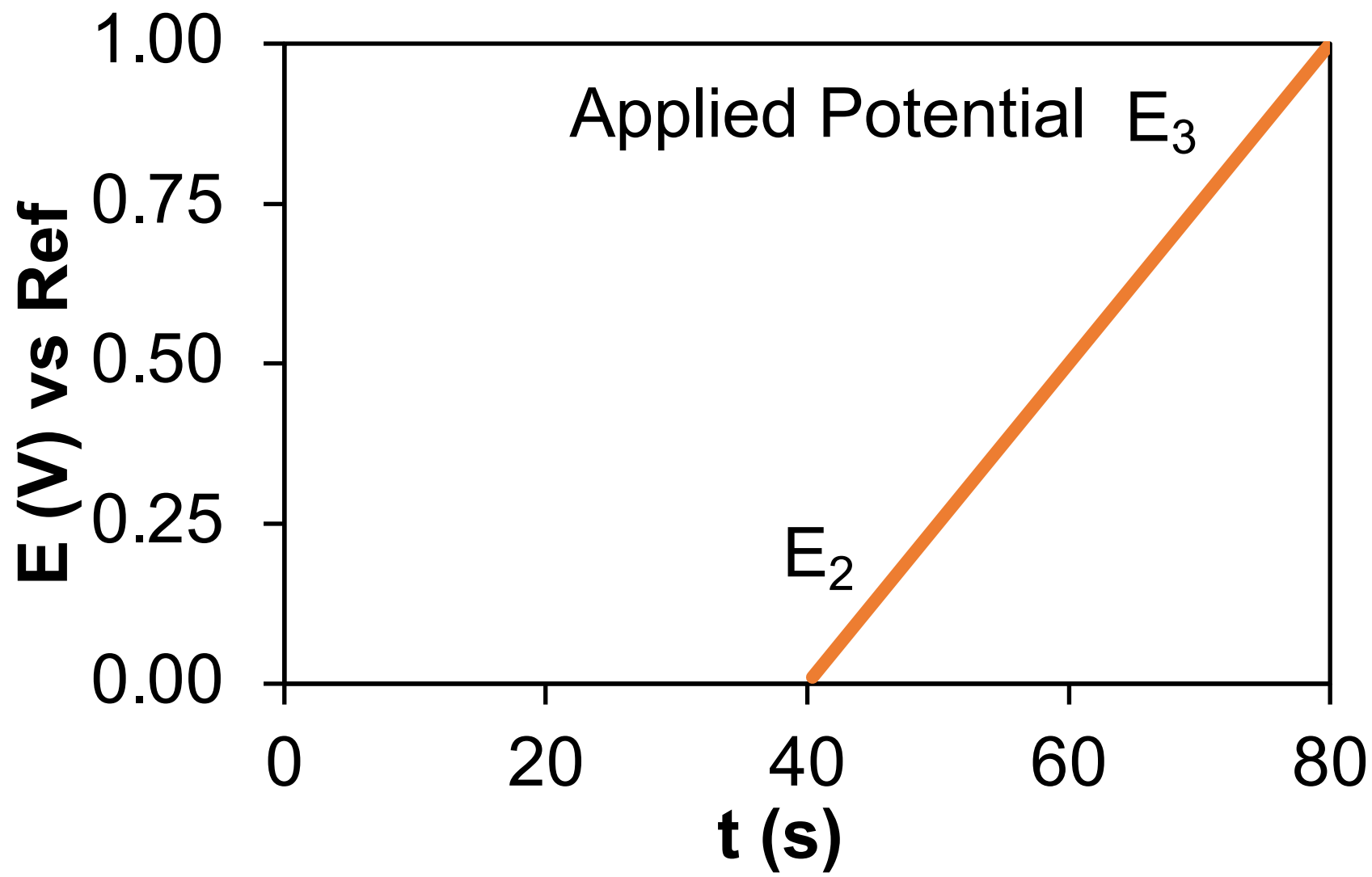

Cyclic Voltammetry

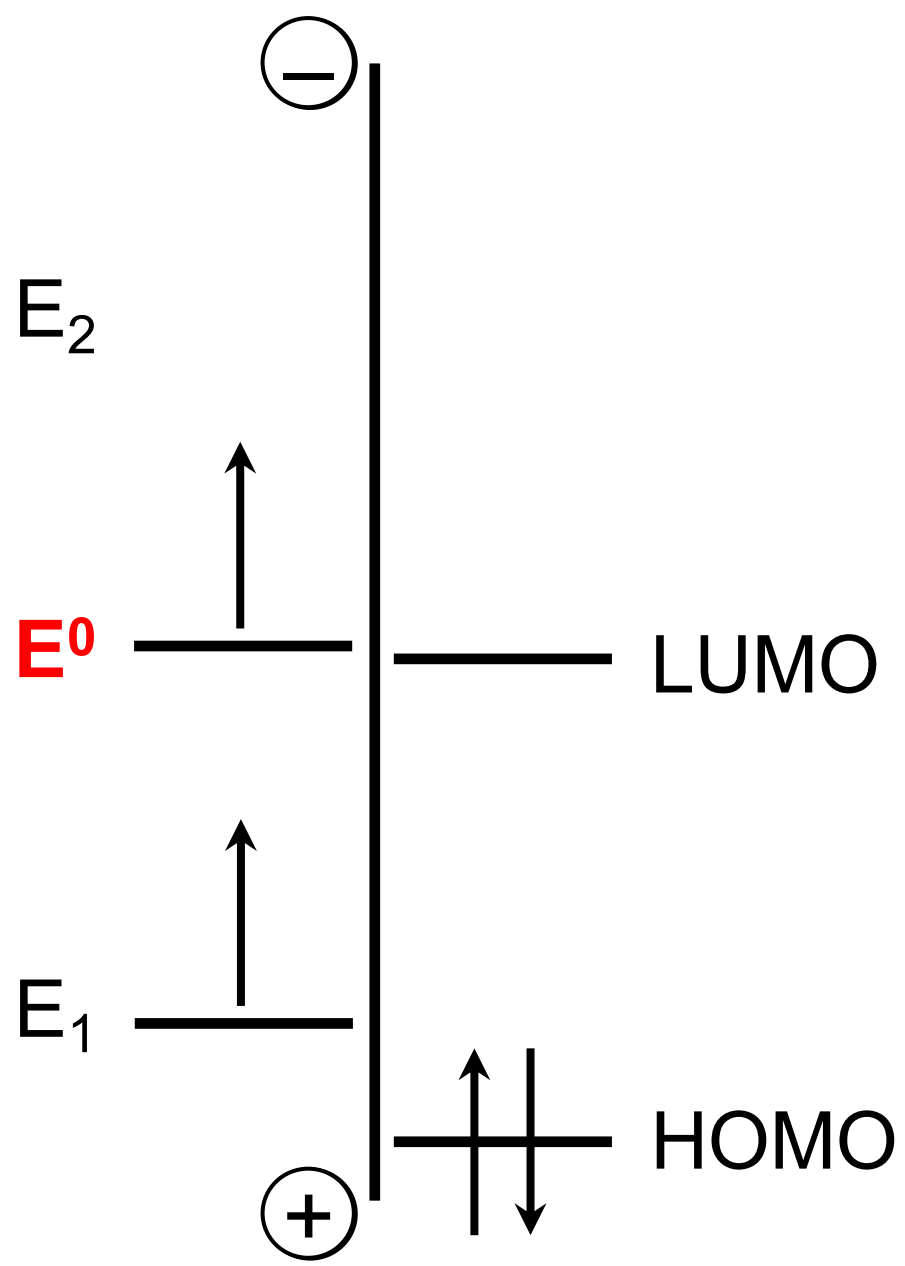

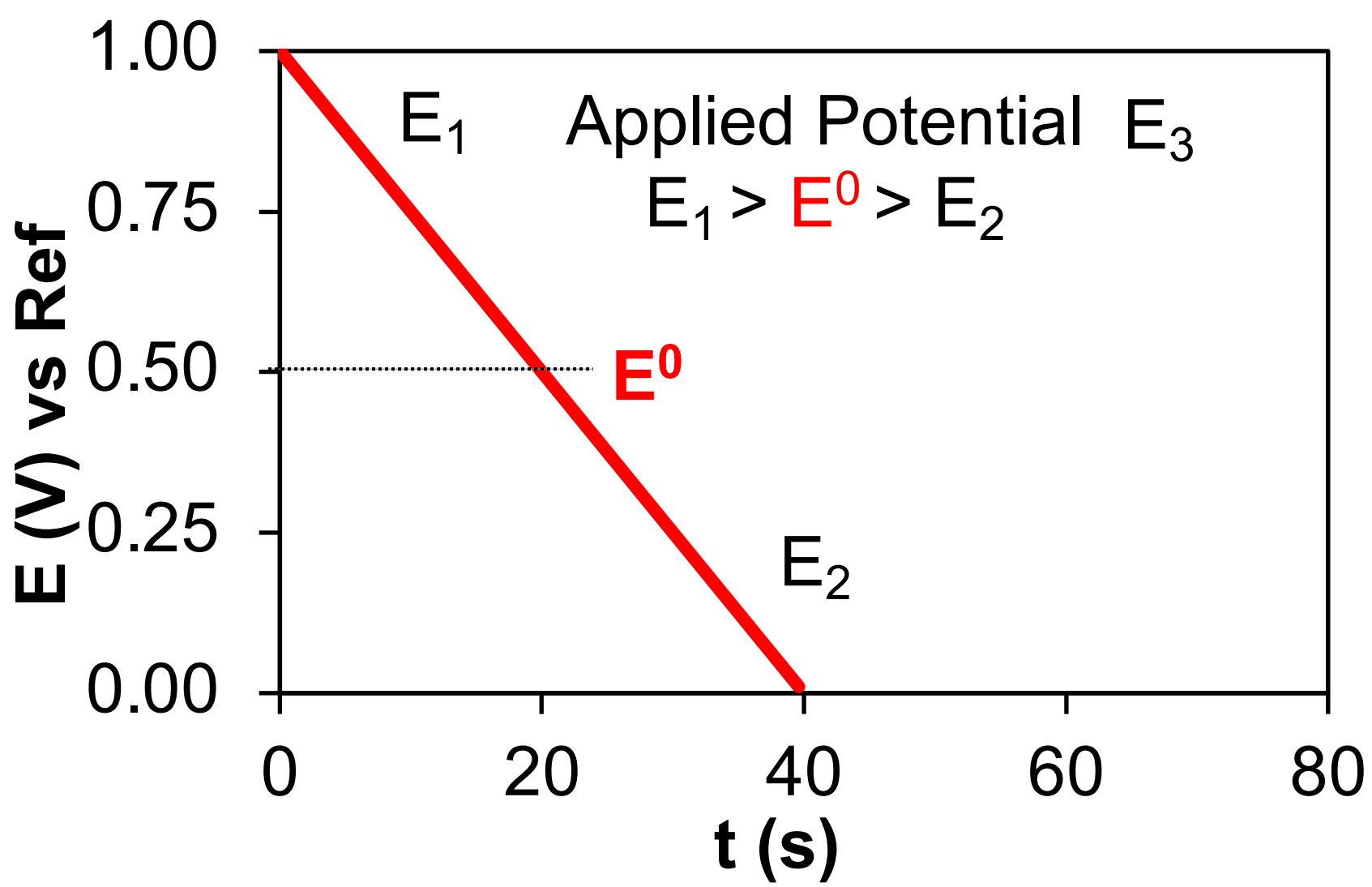

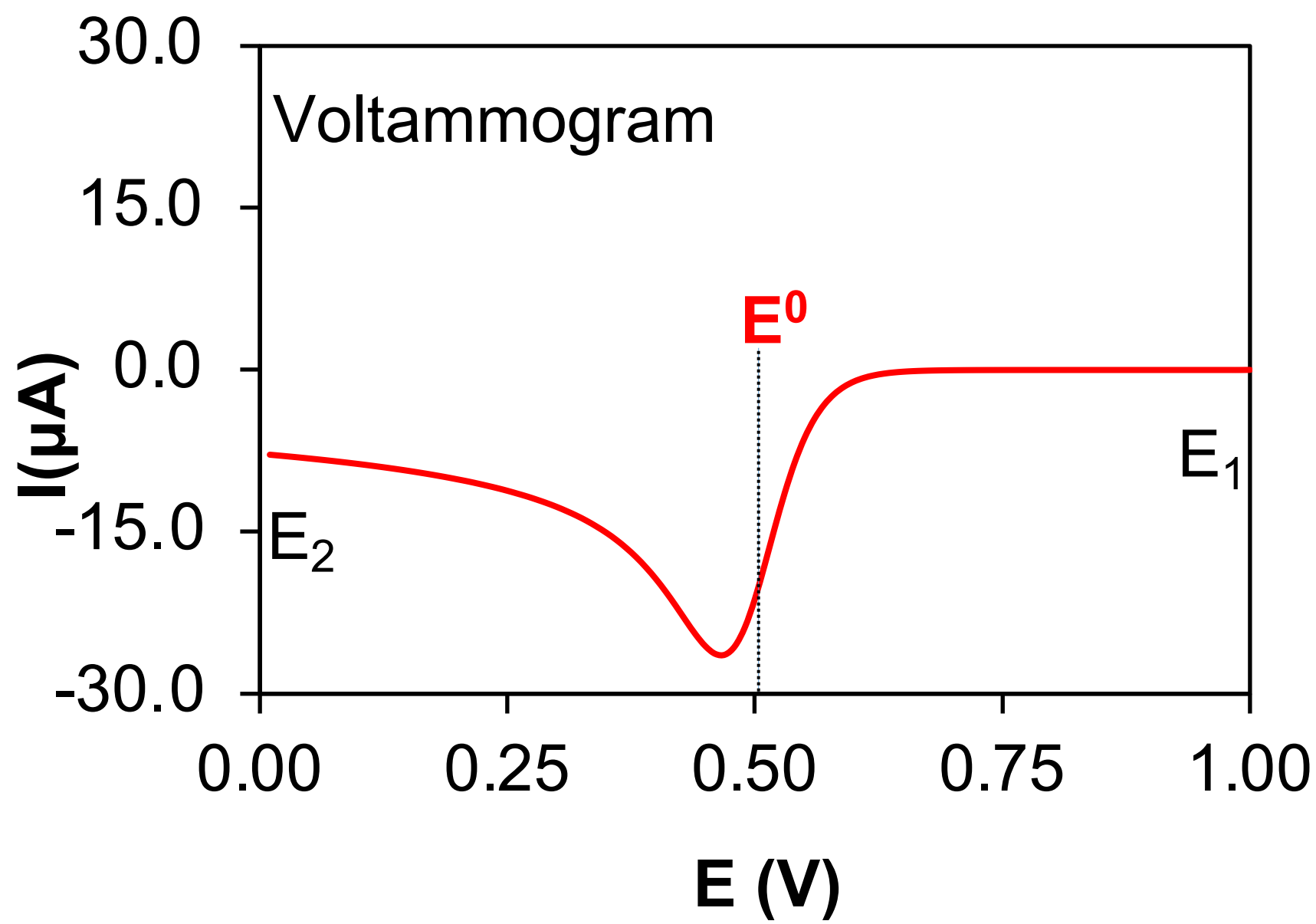

Cyclic Voltammetry

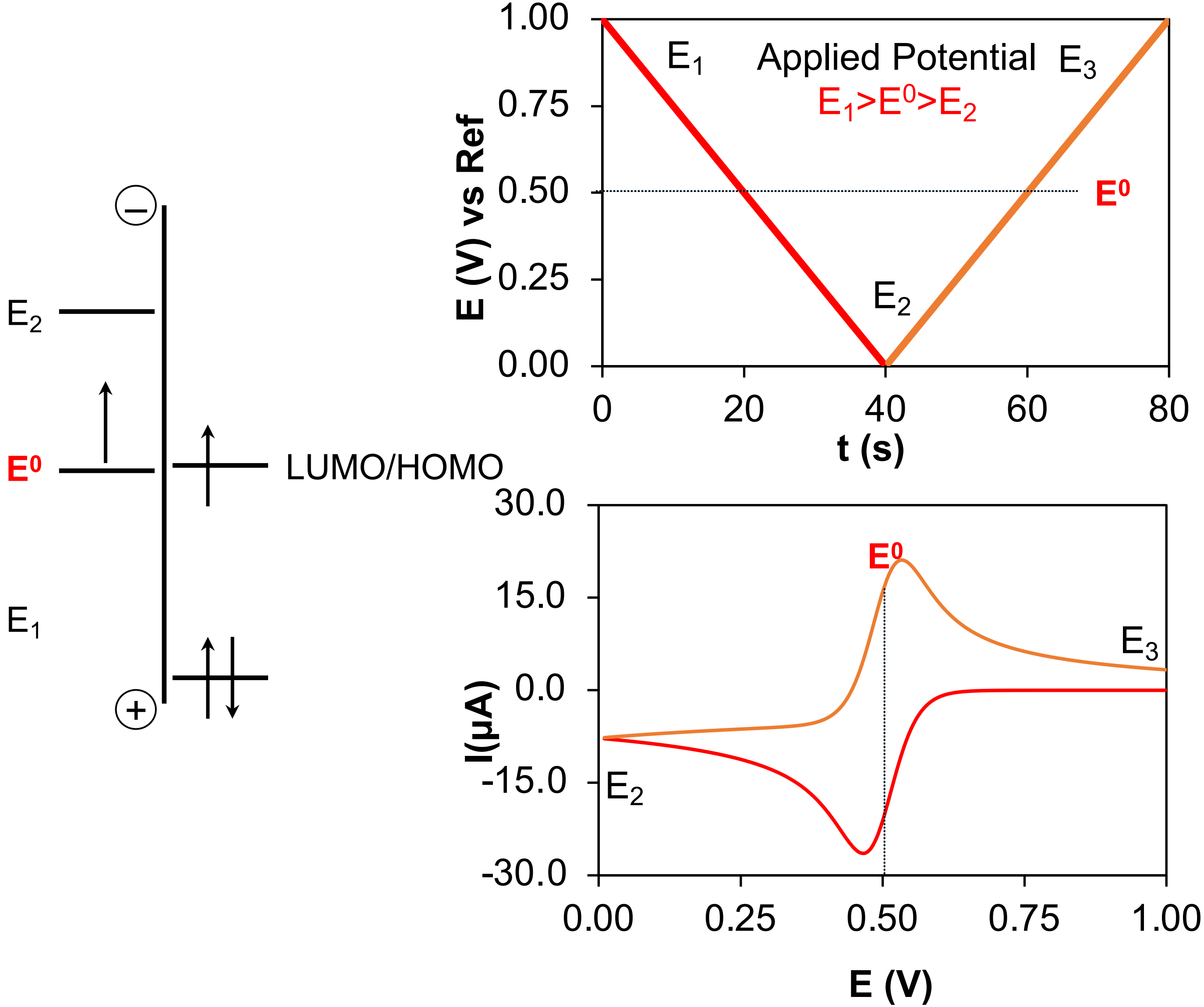

Cyclic Voltammetry

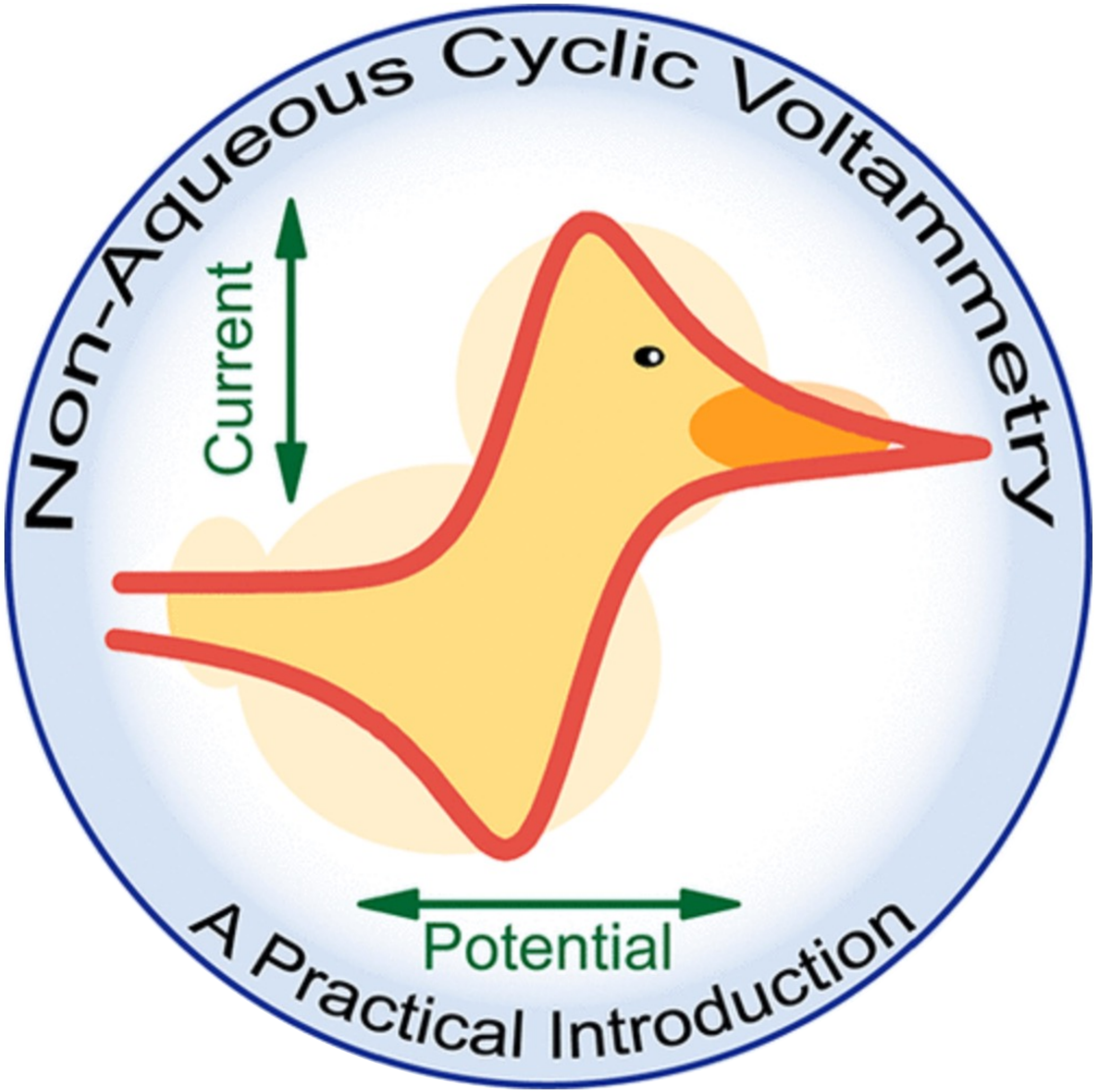

Cyclic Voltammetry

$$I_p = 269An^{3/2}CD^{1/2}v^{1/2}$$

Peak current (I_p) provides a quantitative measure of the concentration, diffusion coefficient (D), and electron transfer number (n) of a redox-active species in solution.

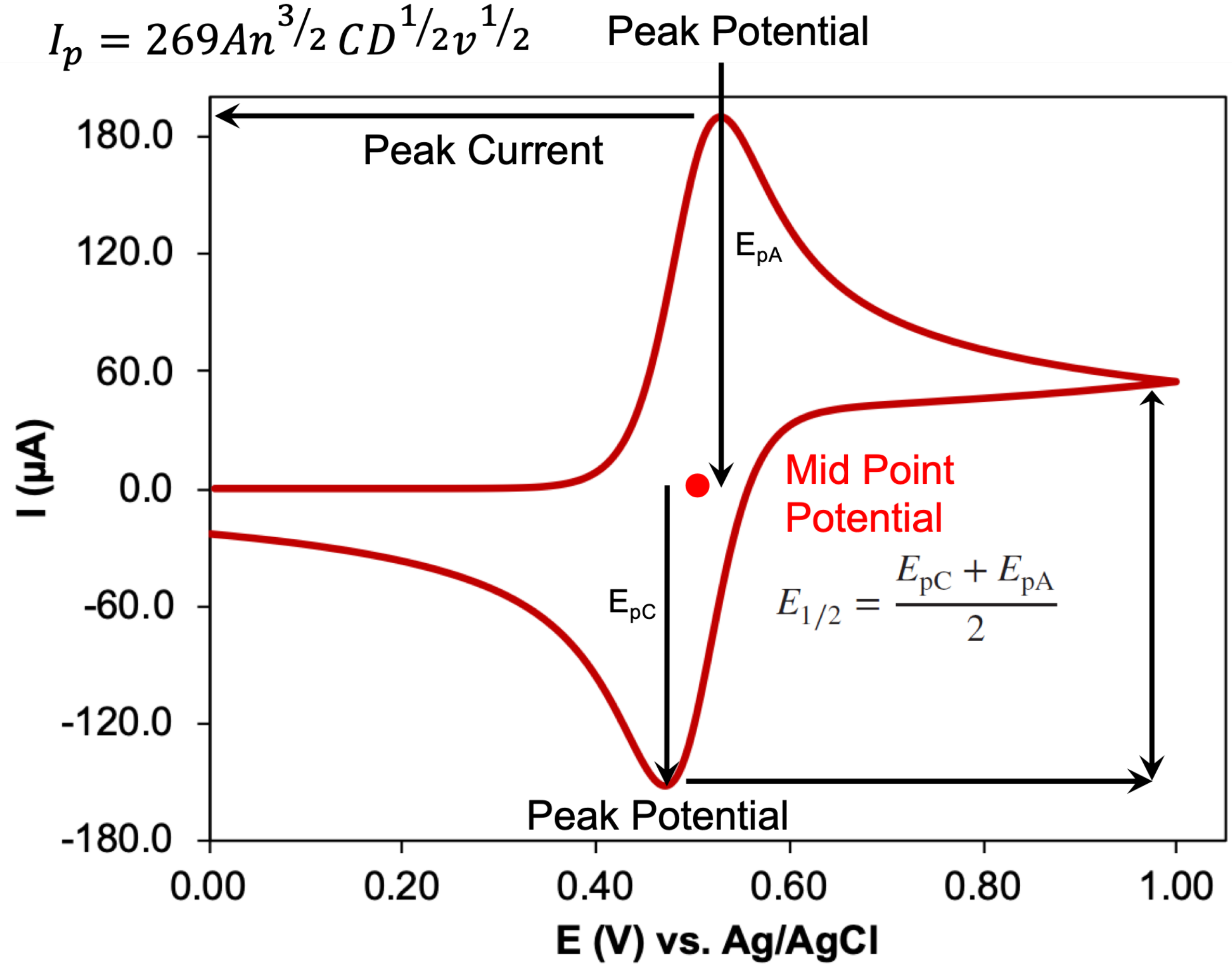

$E_{1/2}$ is a representative of E^0 and quantitative measure of a substance’s tendency to gain or lose electrons in a redox (reduction–oxidation) reaction.

Effect of Chemical Reactions

A CV with: forward oxidation reaction

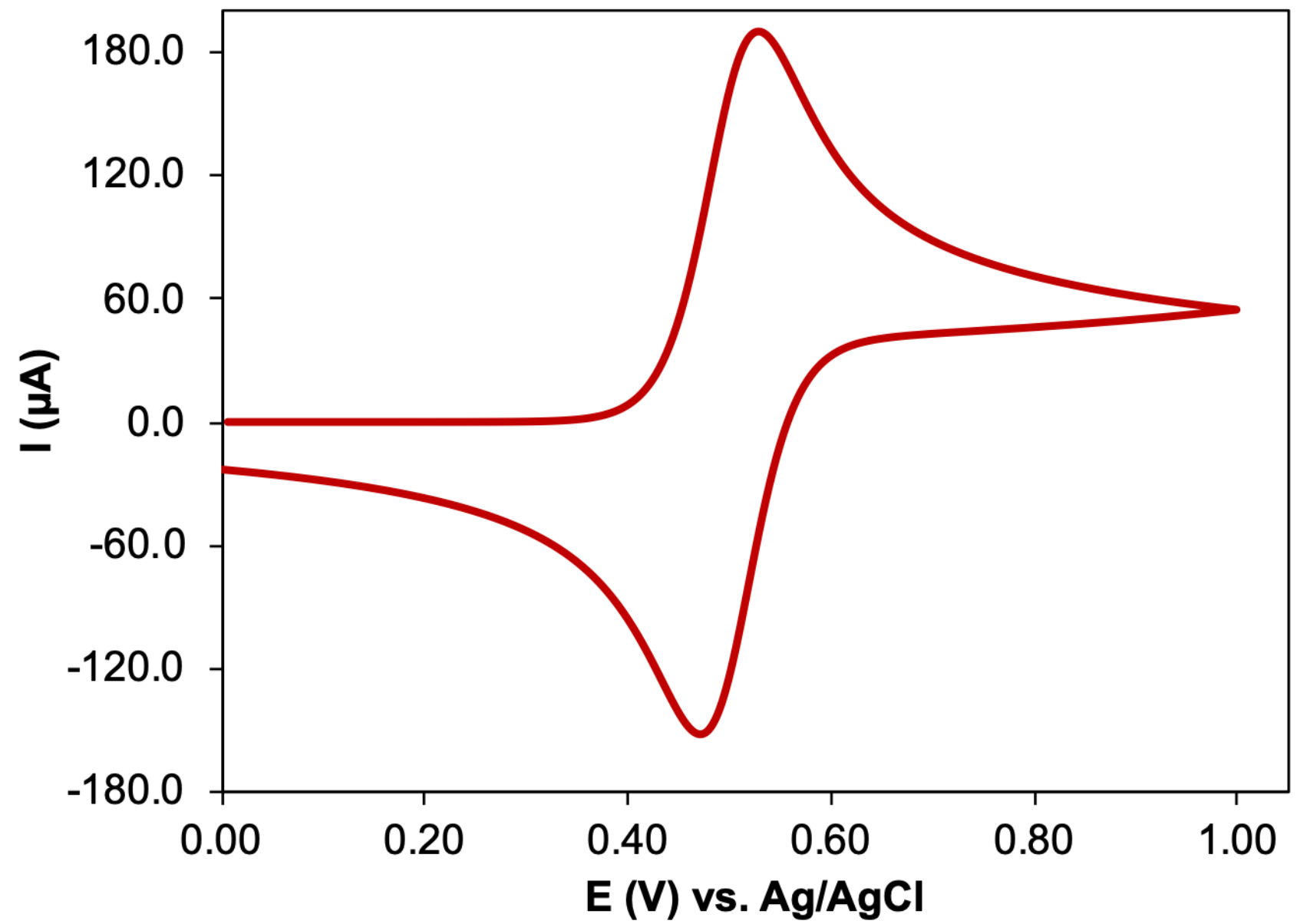

and backward reduction reaction

CV of ascorbic acid oxidation.

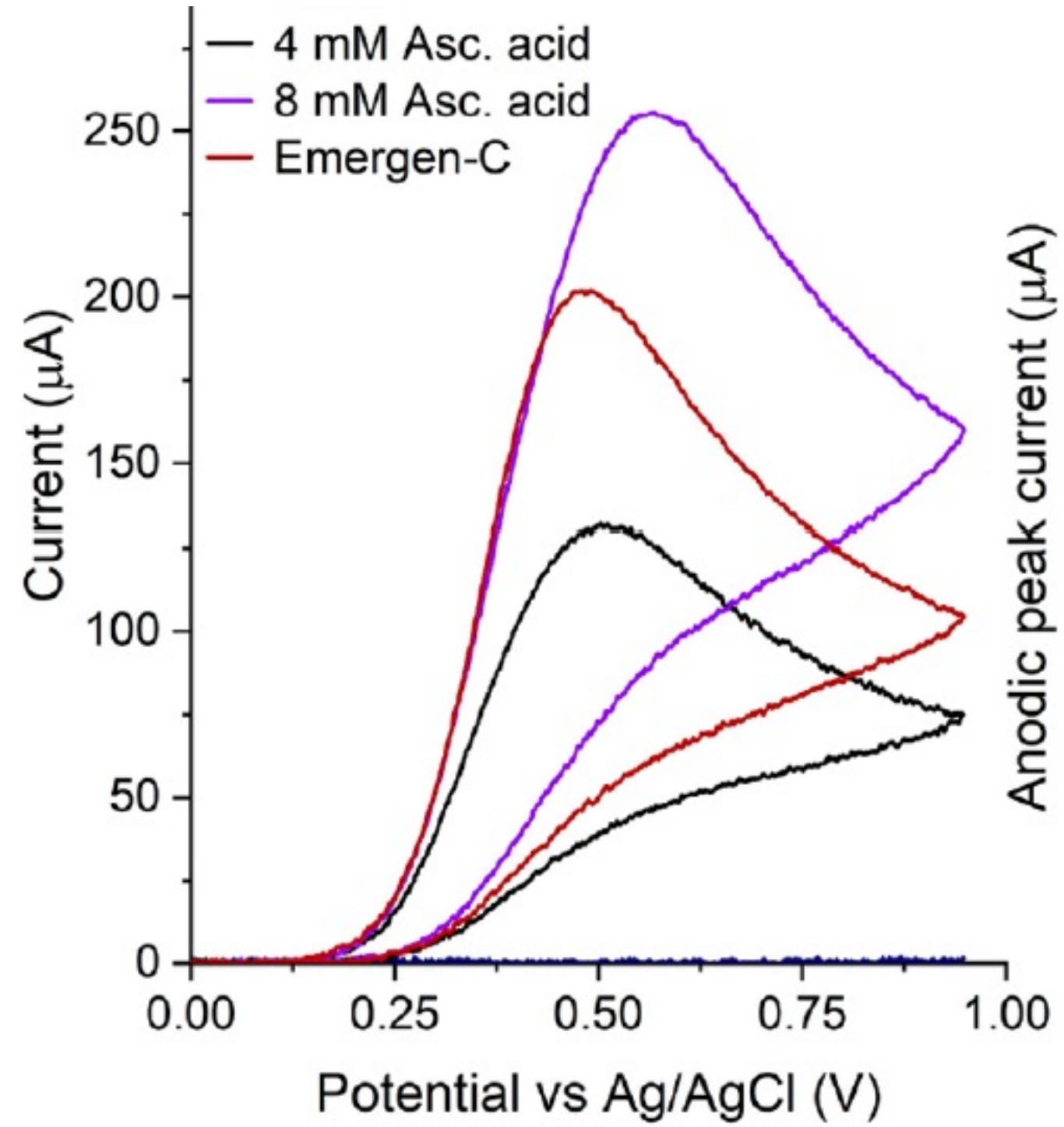

No backward reaction.

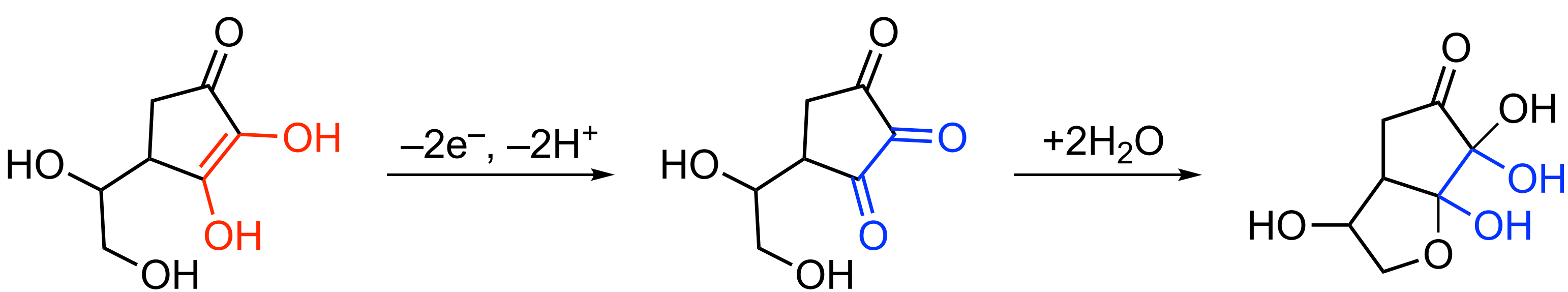

Chronoamperometry

Chronoamperometry applies a constant potential and measures current over time; the potential is sufficient to consume the target redox-active species at the electrode surface.

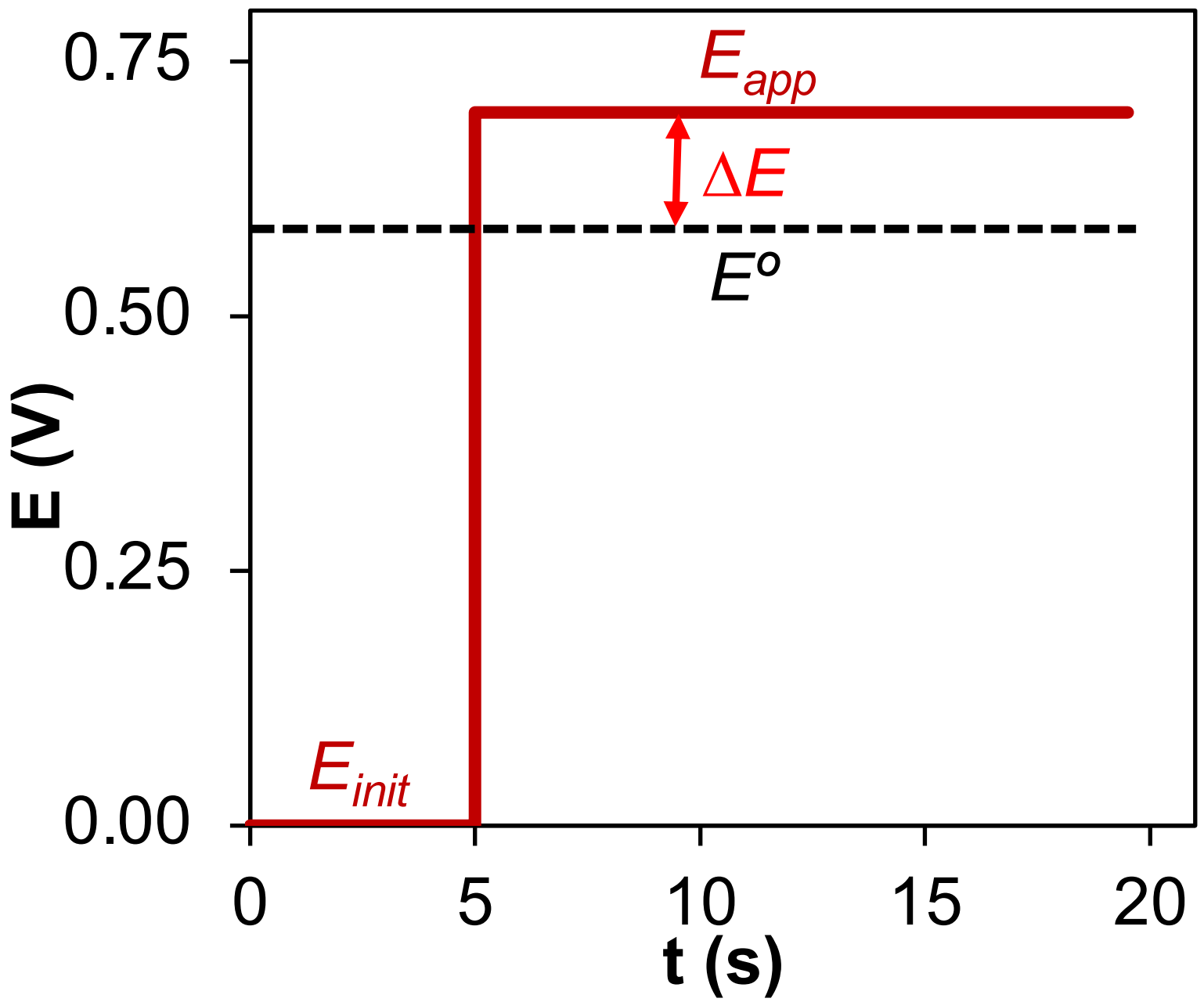

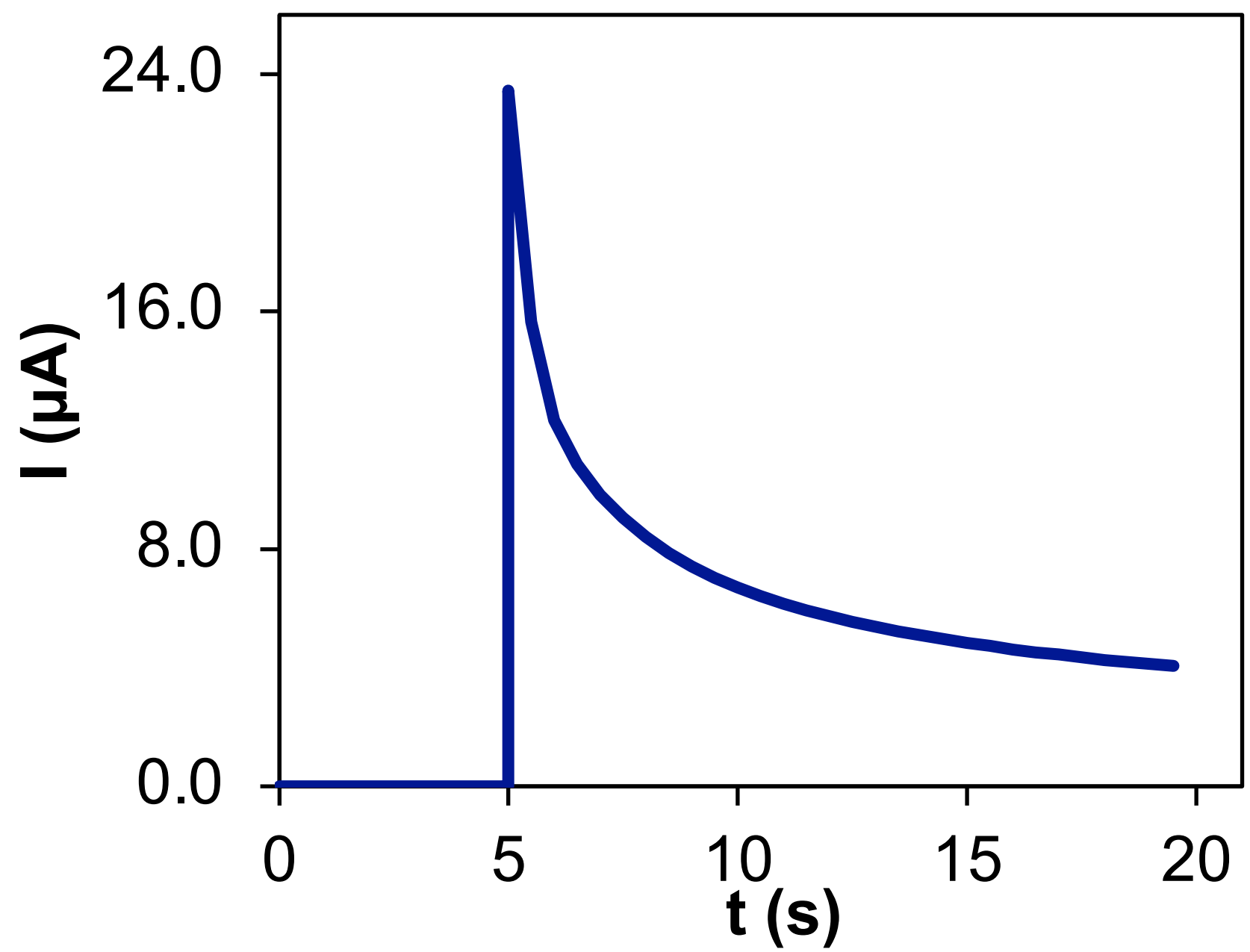

providing insight into mass transport, the concentration of electroactive species, and the consumed charge and number of electrons involved in the reaction.
