# Supplementary material for: Thin-Layer Electrochemistry: Visualizing the Diffusion Layer During Chronoamperometry and Cyclic Voltammetry
Source: J Chem Educ. 2026 Jul 16;103(8):4704–10. doi: 10.1021/acs.jchemed.5c01710 (PMC13472089; doi:10.1021/acs.jchemed.5c01710)
Supplement: Supplementary file 2 [file ed5c01710_si_004.pdf]

Supporting Information

Thin-Layer Electrochemistry: Visualizing the Diffusion Layer During Chronoamperometry and Cyclic Voltammetry

Mohammad Rafiee[a]*, Lida Khalafi[b], Keya Pandey[a], Mary Gipson[a]

[a] Division of Energy, Matter and Systems, School of Science and Engineering, University of Missouri–Kansas City, Kansas City, Missouri 64110, United States

[b] College of Arts and Sciences, Avila University, Kansas City, Missouri 64145, United States

* Mohammad Rafiee: mrafiee@umkc.edu

Table of Contents

1. **Chemical reagents** 2
2. **Instrumentation** 2
3. **Apparatus and labware** 2
4. **Experimental Procedure** 3
5. **Safety** 5
6. **Preparing the Solutions** 5
7. **File Conversions** 5
8. **Instruction for Measuring the Thickness of Diffusion Layer using pdf File** 7
9. **Example of Results:** 14

Supporting Information

1. Chemical reagents

- Methyl viologen dichloride hydrate, Thermo Scientific Chemicals Methyl Viologen hydrate, 98%, was purchased from Fisher Scientific. $LD_{50} \approx 57$ mg/kg
- Sodium Chloride, Fisher Bioreagents, was purchased from Fisher Scientific.
- Platinum wire 99.99%, 0.5 mm diameter, was purchased from goodfellow.
- Silver wire 99.99%, 0.5 mm diameter, was purchased from goodfellow.
- Multipurpose 110 copper sheet, 5mm thickness was purchased from McMaster.
- Miniature heat-shrink tubing clear, ID before shrinking 0.75 mm, was purchased from McMaster.
- Ultra-thin wall heat shrink tubing, ID before shrinking 5 mm was purchased from McMaster.
- Fine grit polishing pads (fine sandpapers), was purchased from BASi.
- Deionized water was used for making the solutions.

2. Instrumentation

- Pine Wavenow XV 100 Potentiostat
- Mettler Toledo Precision Balance (readability of 1 milligram)
- A digital microscope with LCD display that has all the components: 4.3 inch Premium 1080P HD Portable LCD Digital Color Microscope with Dual-LED Illumination, https://amscope.com/products/c-dm140?gclid=CjwKCAjw8ZKmBhArEiwAspcJ7p9_YrK90_YQeJhJhUWaPvZRWwpQzMRgEjhmFIwbP_ddToLZVJSpoRoCYTYQAvD_BwE. Accessed accessed 06/29/2023.

3. Apparatus and labware

- Alpha Nanotech High-Precision UV Quartz Cuvette (0.35 ml, 1.00 mm, 2-Sided); for the cell.
- Ace Glass, Inc. Heat gun, was purchased from Fisher Scientific.
- 100- and 50-mL volumetric flasks.

- Thermo Scientific™ Samco™ Fine Tip Transfer Pipettes, for transferring the solution to the cell.

4. Experimental Procedure

Fabrication of the cell and electrodes are described in our recent paper in details.[1] The difference of the electrochemical setup used for current study compared to previous one is that here the three electrolde system was used and instead of a DC adapter potentiostat was used for chronoamperometric or voltammetric experiments. Working and counter electrodes were the same as previous study. A homemade thin-layer electrochemical cell was combined with an optical microscope (100× magnification) to monitor color changes during the experiments. The cell was constructed from a UV–Vis cuvette with a 0.1 cm path length, which accommodated the working, counter, and reference electrodes and could be positioned under the microscope for observation. The working electrode was prepared from a flat copper strip (5.0 cm long, 0.5 cm wide, 0.05 cm thick) sealed in heat-shrink tubing. Approximately 1.0 cm at one end was left uncovered for connection to alligator clips attached to the potentiostat, while the opposite end was carefully sealed to expose only the cross-section of the strip to the solution. This exposed band defined the active electrode surface with dimensions equal to the strip width (5 mm) and thickness (0.5 mm). The counter electrode was a platinum wire (0.05 cm diameter, 5.0 cm length), sealed in heat-shrink tubing with both ends (about 10 mm) uncovered. One end was connected to the alligator clips, while the other end was immersed in solution to serve as the anode. The reference electrode was a silver wire (0.05 cm diameter), with its middle section insulated by heat-shrink tubing. One end of the silver wire was oxidized in NaCl solution to form AgCl on its surface, and the other end was connected to the potentiostat as the reference electrode lead. The electrolyte solution contained 0.05 M NaCl to provide a stable potential for the Ag/AgCl reference electrode.

Supporting Information

To visualize color changes, relatively high concentrations of MV^{2+} (5–10 mM) were used in an aqueous solution containing 0.05 M NaCl. The solution was purged with high-purity nitrogen gas (99.999%) before being transferred to the electrochemical cell. Electrochemical experiments were performed with a Pine WaveNow XV 100 Potentio/Galvano-Stat. All three electrodes were inserted into the cuvette and placed under the microscope, with the exposed tip of the copper working electrode centered in the field of view. The uncovered ends of the electrodes were connected to alligator clips and attached to the potentiostat (working, counter, and reference connections).

To perform the voltammetric experiment, a negative-going forward scan was applied from 0 to –0.9 V (vs. the reference electrode) to observe the reduction of MV^{2+}, followed by a positive-going backward scan from –0.9 to 0 V to oxidize the electrochemically generated $MV^{+\bullet}$ back to MV^{2+}. The midpoint potential of the voltammogram ($E_{1/2}$, representing E°') was determined by averaging the anodic and cathodic peak potentials, giving a value of –0.58 V. The amperometric experiment was performed by applying –0.70 V for 20 seconds. This potential is 120 mV more negative than E°', which is sufficiently negative to reduce the concentration of MV^{2+} to nearly zero, resulting in complete conversion of MV^{2+} to $MV^{+\bullet}$ at the electrode surface.[13] The microscope was equipped with a digital camera to record color changes at the working electrode surface in real time. The camera was calibrated with a scale bar to enable measurement of the thickness of the colored region. Video recordings were processed into GIF or PDF files with the rate of 10 frames per second. The thickness of diffusion layer was measured as a function of time (0.1 s increments) during chronoamperometric experiments.

5. Safety

Goggles and gloves should always be worn during this experiment. Methyl Viologen (MV^{2+}) is commonly used herbicide that is toxic through ingestion, inhalation, and dermal contact. Rinse eyes and skin if exposed, collect spillage, and avoid release to the environment. Remove personal protective equipment after handling this product. Proper waste disposal should be implemented after every use of this chemical during the experiment.

6. Preparing the Solutions

To prepare the solutions using solid substances, the following equation was used.

$$\text{Molarity} \times \text{Molar Mass} \times \text{Volume of Desired} = \text{Mass of substance needed}$$

To prepare 100 mL 0.05 M NaCl stock solution, 0.292 g of NaCl was dissolved in DI water in a 100 mL volumetric flask and diluted to mark.

$$0.050 \text{ mol/L} \times 58.44 \text{ g/mol} \times 0.100 \text{ L} = 0.292 \text{ g of NaCl}$$

To prepare 50 mL 5 mM (0.005 M) methyl viologen solution, A mass of 0.0688 g of methyl viologen dichloride monohydrate was weighed, transferred to a 50 mL volumetric flask, dissolved in the 0.05 M NaCl stock solution, and diluted to the mark with the same NaCl solution.

$$5.00 \times 10^{-3} \text{ mol/L} \times 275.18 \text{ g/mol} \times 0.05 \text{ L} = 0.0688 \text{ g of methyl viologen}$$

7. File Conversions.

The video recordings (mp4) obtained from the microscope's camera during amperometric and voltammetric experiments were converted to GIF format at 10 frames per second (10 FPS) using free online tools. An example website for converting mp4 or other video formats to GIF is https://ezgif.com/video-to-gif (accessed September 23, 2025). Each GIF frame represents 0.1 seconds of changes recorded at the electrode surface. The frame-by-frame sequence allows measurement of the diffusion layer thickness (colorful area) at each specific time point, determined by the FPS setting. For PDF conversion, frames were extracted individually from the GIF files. Example exported PDF and GIF files corresponding to the original mp4 recordings are provided in the Supporting Information.

Supporting Information

An example video of the expansion of the diffusion layer during chronoamperometry experiment is available at:

https://www.youtube.com/watch?v=Zok7E2R7uQQ, accessed September 25, 2025

The GIF and PDF files after the conversion of the mp4 video are available as additional Supprting Information files related to this paper.

An example video of the changes in diffusion layer during cyclic voltammetry experiment is available at:

https://www.youtube.com/watch?v=kYDCHia8CsQ, accessed September 25, 2025

8. Instruction for Measuring the Thickness of Diffusion Layer using pdf File

a) Open the pdf file in Adobe Acrobat Reader.

b) Click on the “Find text or Tools” tab (see Figure S1).

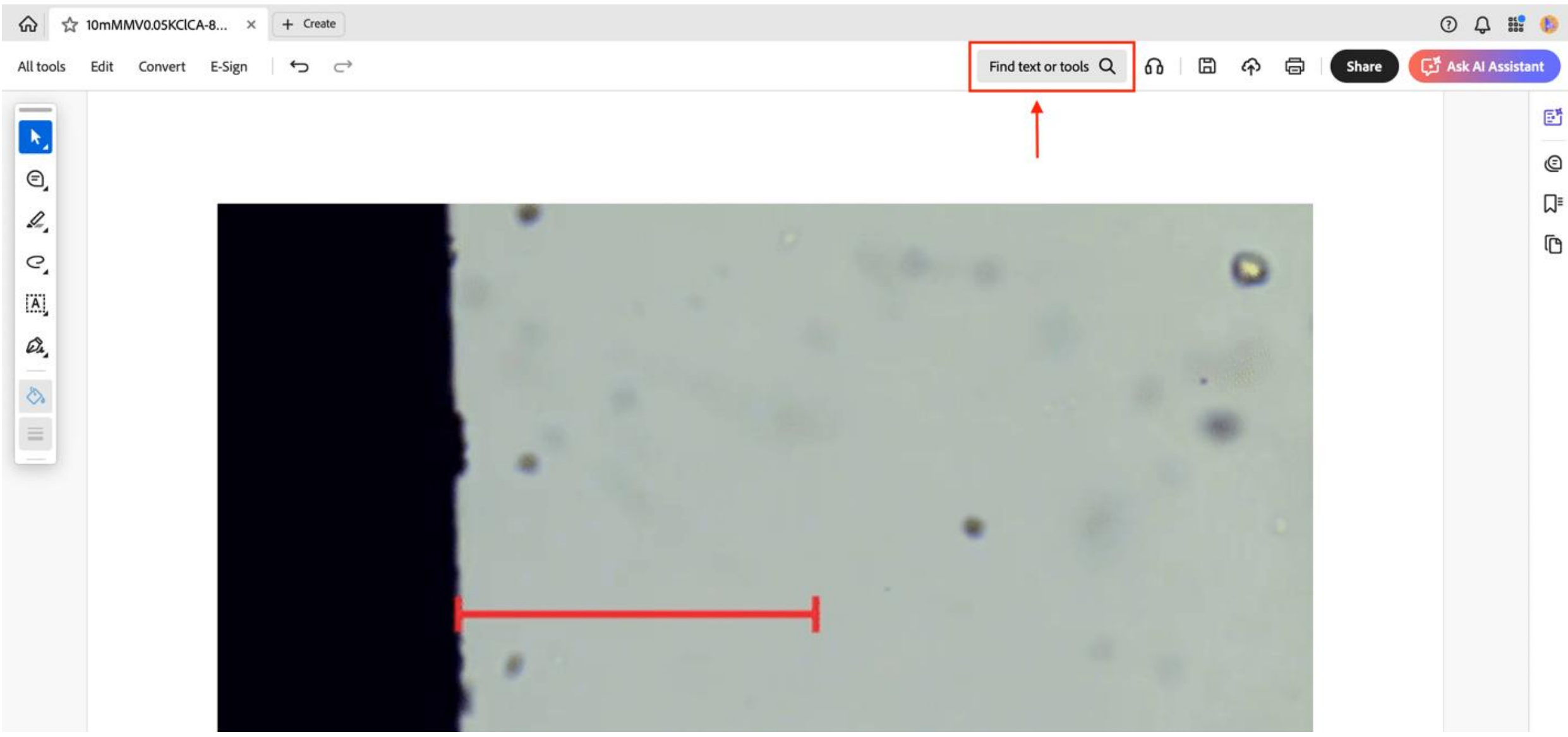

Figure S1. Demonstrating the position of “Find text or Tools” tab in Adobe Acrobat.

c) Open the “Measure” tool (see Figure S2)

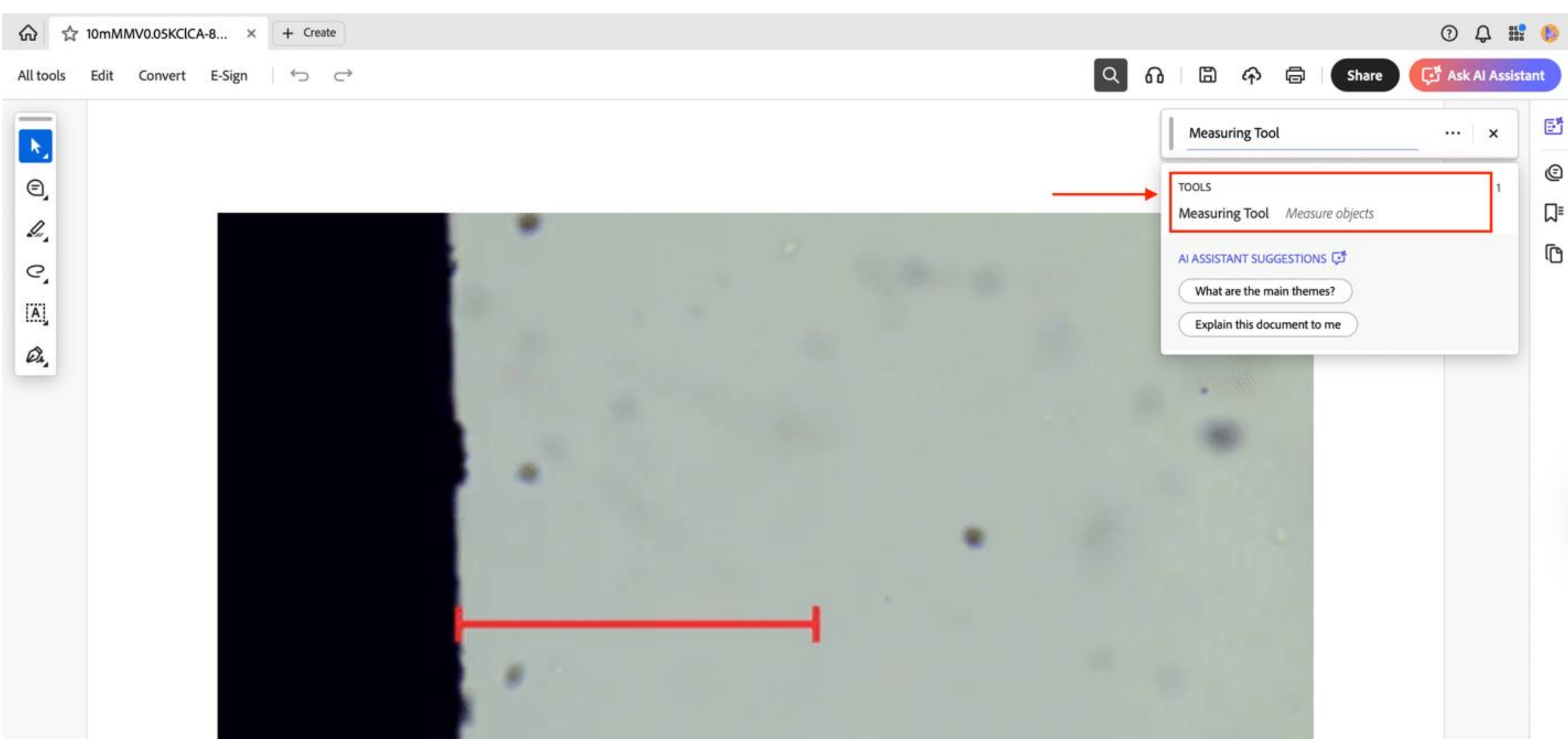

Figure S2. Demonstrating the position of “Measure” tab in Adobe Acrobat.

d) Activate the **"Measuring Tool."** as demonstrated in Figure S3.

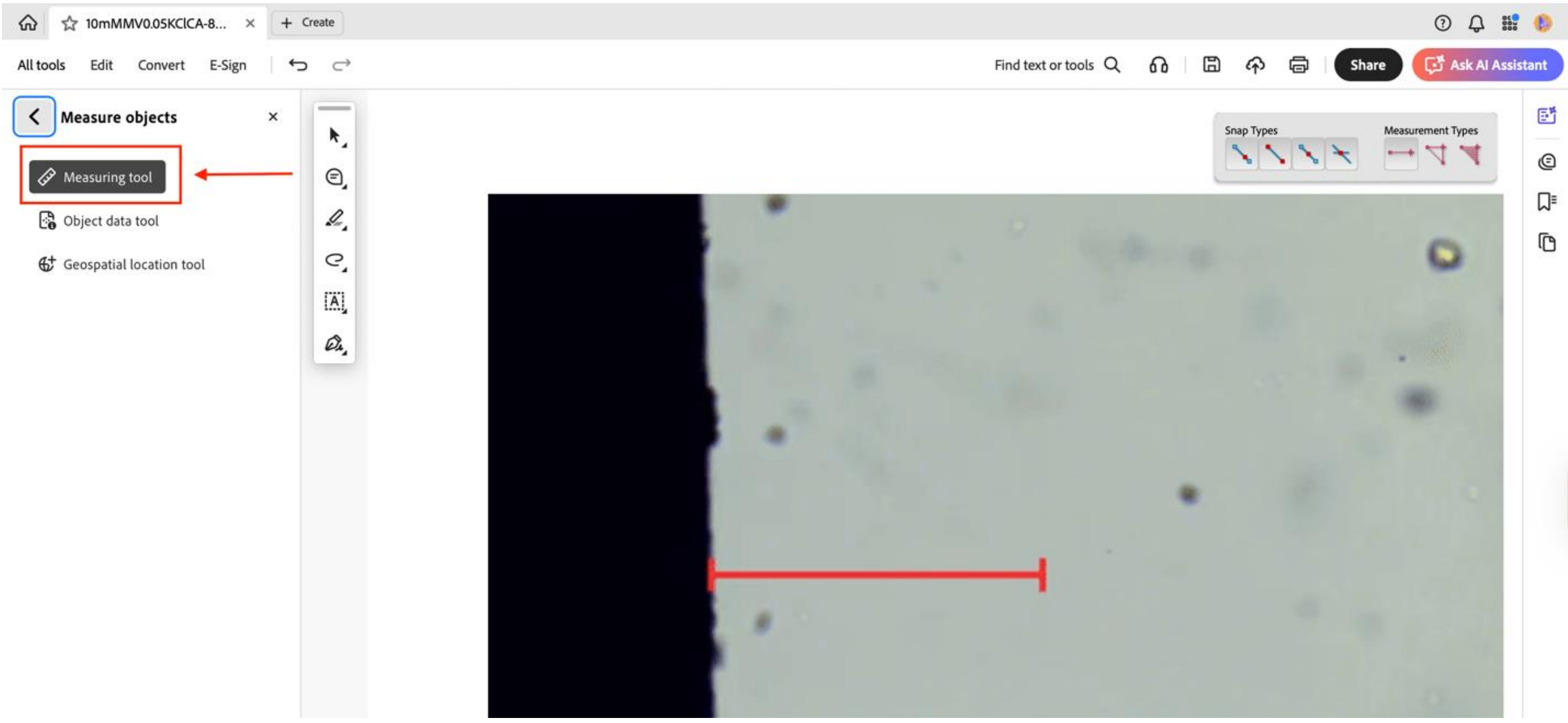

Figure S3. Demonstrating the position of "Masuring Tool" tab in Adobe Acrobat.

e) There are several measurement options. Select the **"Straight line"** as demonstrated in Figure S4**.**

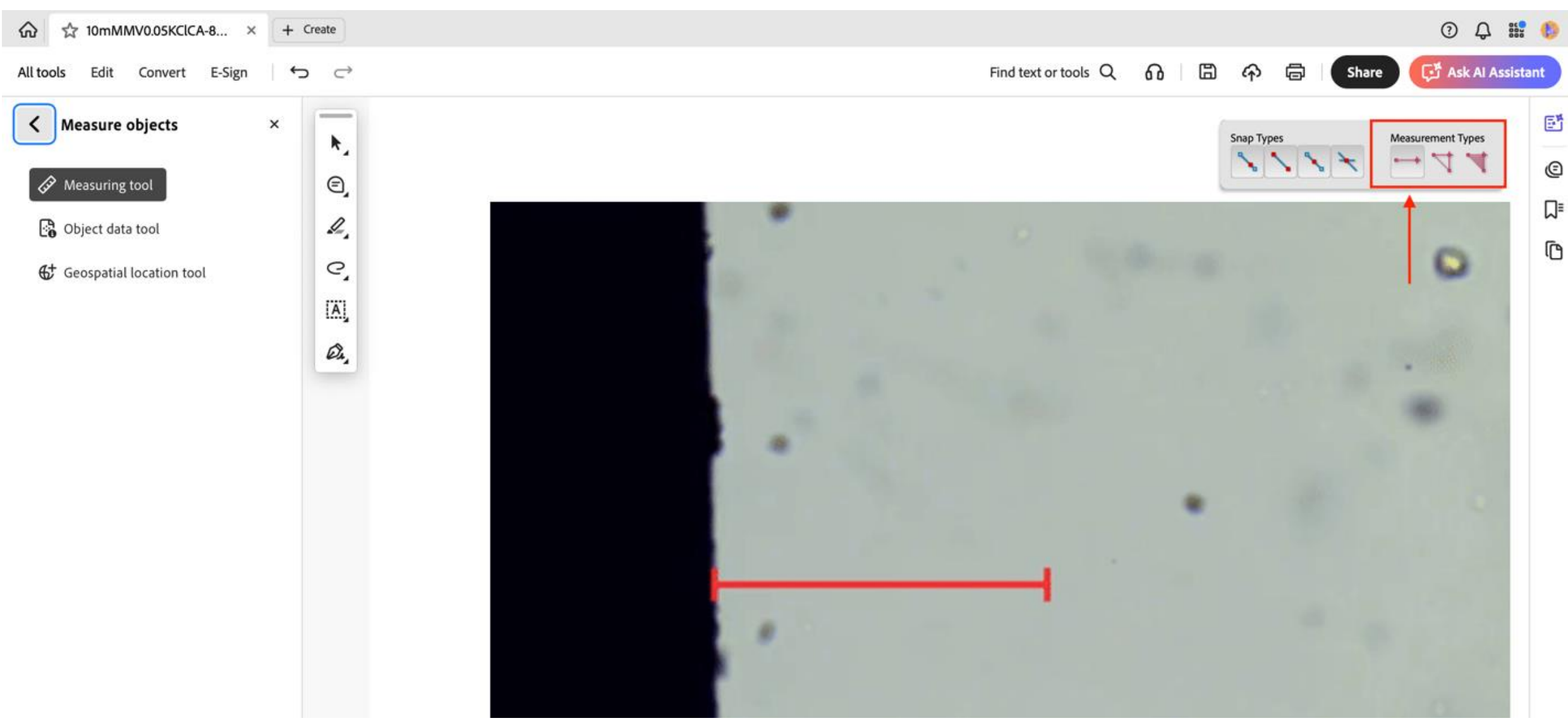

Figure S4. Demonstrating how to use "straight line" for length measurement in Adobe Acrobat.

f) To measure the horizontal distance, right-click and select **"Turn Ortho On"** to lock the orientation as demonstrated in Figure S5.

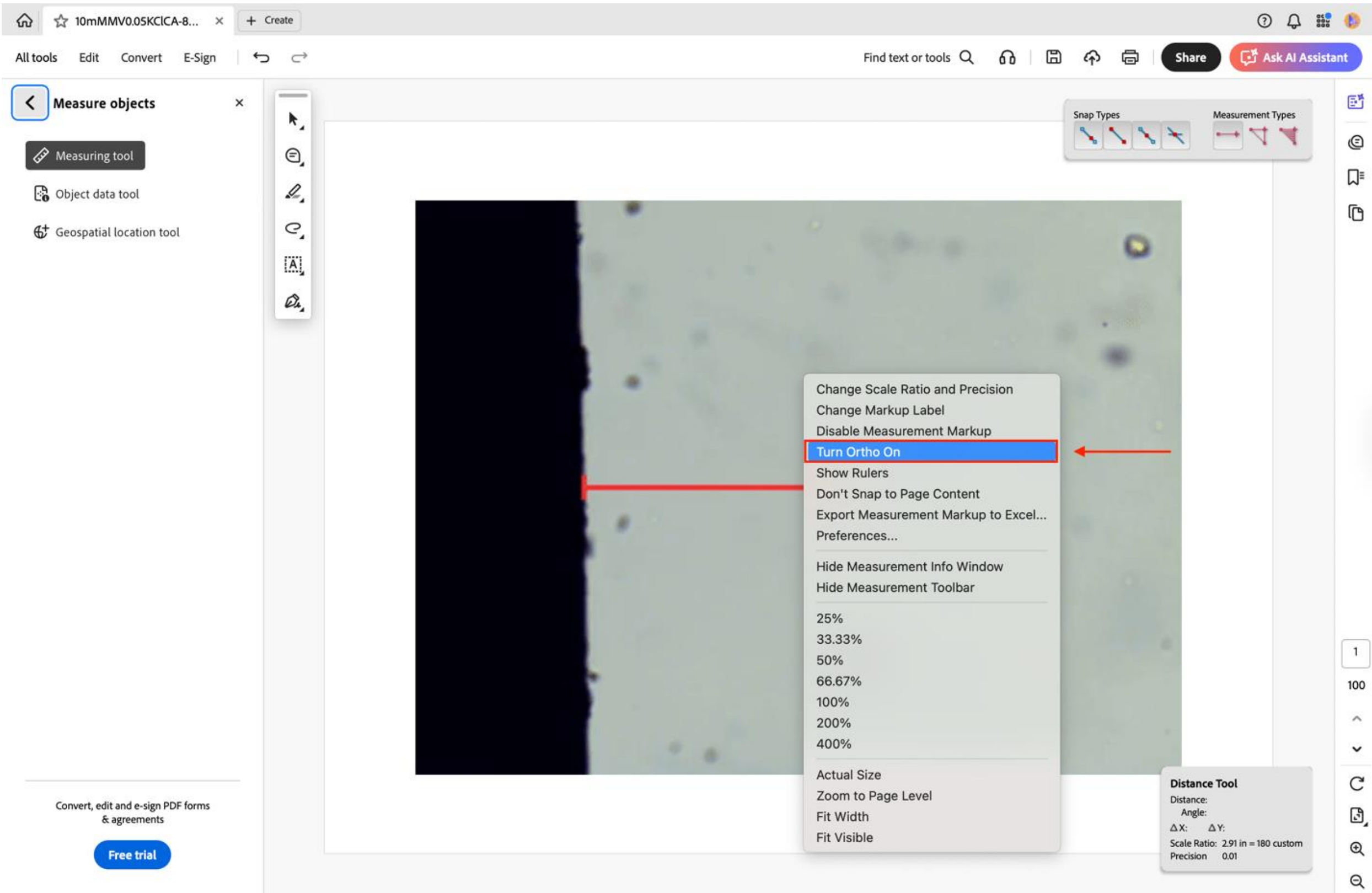

Figure S5. Demonstrating how to select "Turn Ortho On" to lock the measurement orientation in Adobe Acrobat.

g) As is shown in Figure S6, **the scale bar** is equal to **2.91 inches**. However, the scale bar's actual length differs from the measurement made by the calibrated microscope. The actual length is **0.018 cm** (According to the measurement made by the calibrated microscope). Therefore the scale needs to be **calibrated** for pdf files as demonstrated in Figure S6.

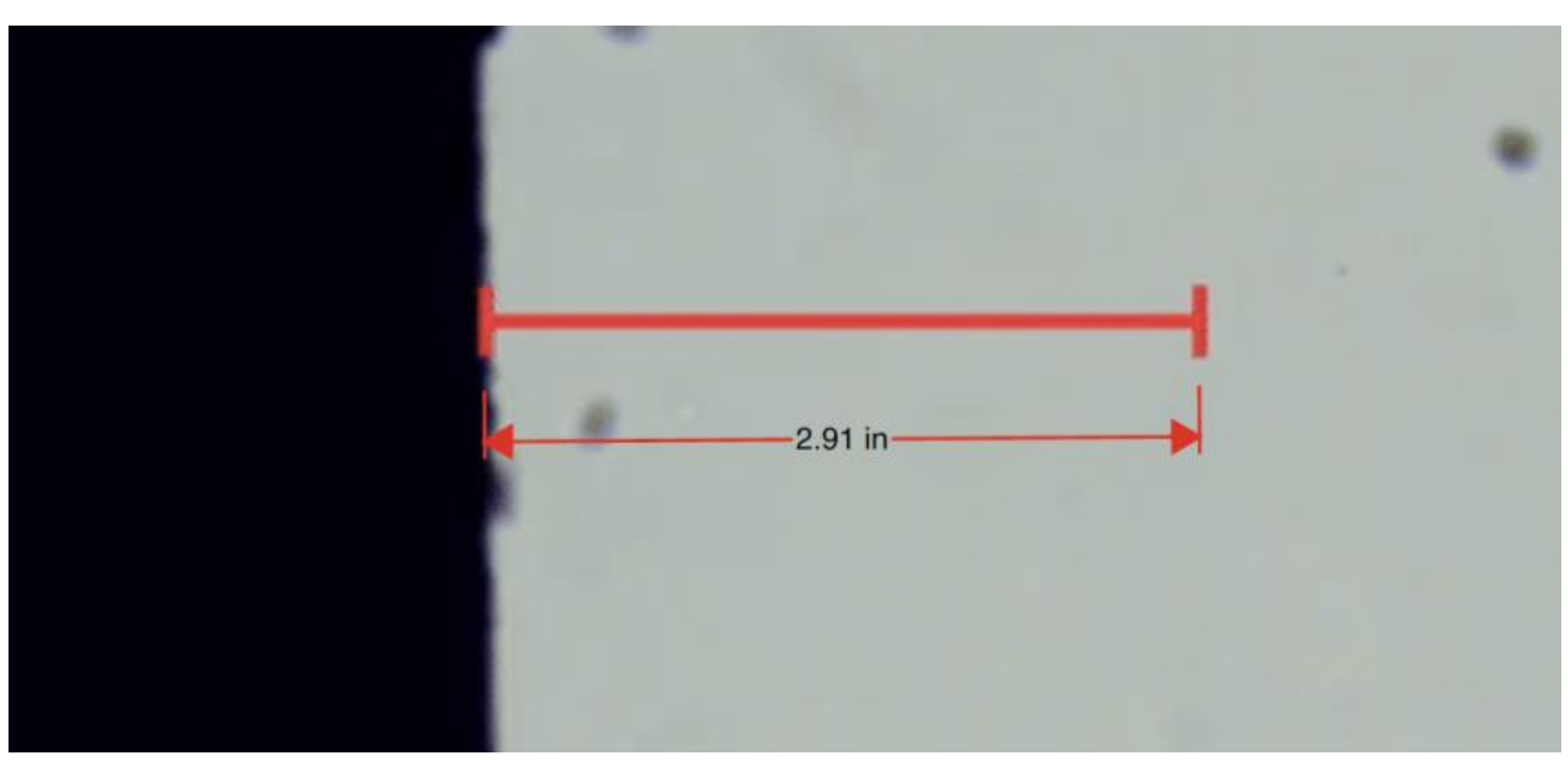

Figure S6. The scale-bar and its measured length in Adobe Acrobat.

h) To calibrate the scale, right-click and select **"Change Scale Ratio and Precision"** to customize the distance unit as demonstrated in Figure S7.

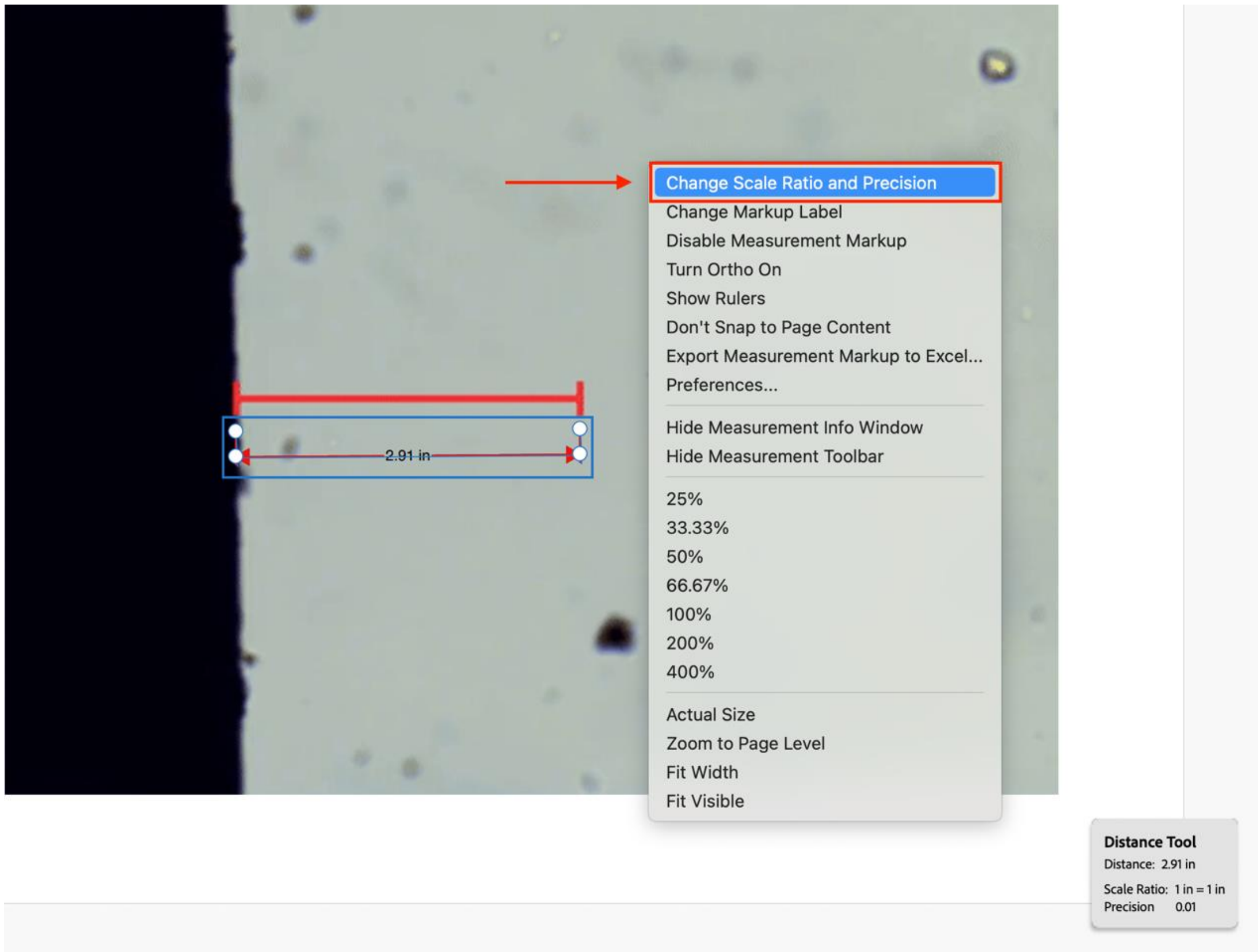

Figure S7. Demonstrating how to open the calibration tool in Adobe Acrobat.

i) We predetermined **2.91 inches to be equal to 180 custom (μm)** (Because this measurement tool can only measure distances up to two decimal places/the maximum precision of this tool is two decimal places). Then, in Excel, the distance unit must be changed from **μm to cm by multiplying by 10^{-4}** as demonstrated in Figure S8.

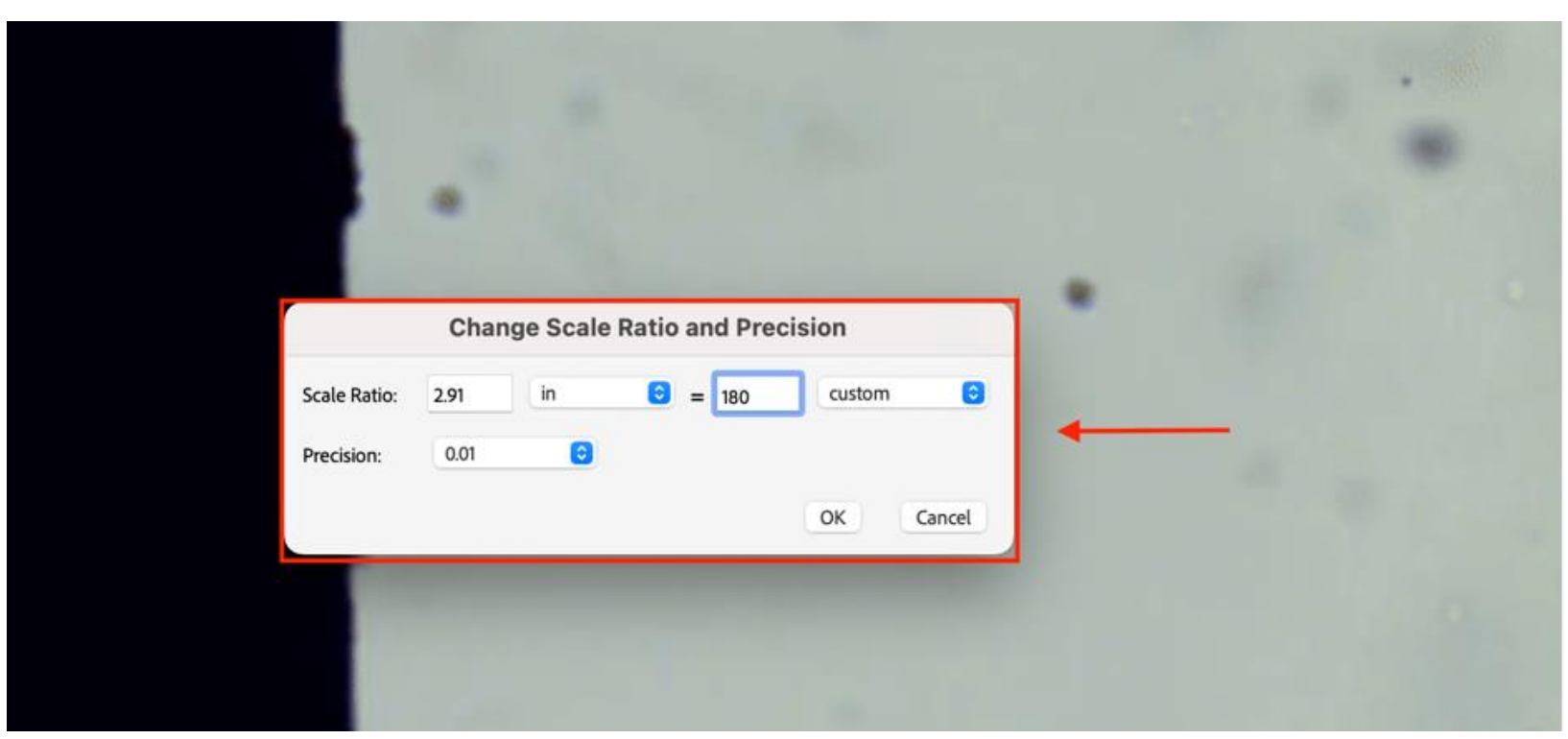

Figure S8. Demonstrating how to define the conversion factor in Adobe Acrobat.

j) Click once to select the starting point to measure the distance between two specific points. Then, click for the second time to locate where it ends. The measurement value should be included with the line after completing the measurement.

k) To change the default setting, right-click and go to the **"Preferences"** and **"Properties" tabs**. Then, you can click on **"Make Current Properties Default"** as demonstrated in Figure S9-S12.

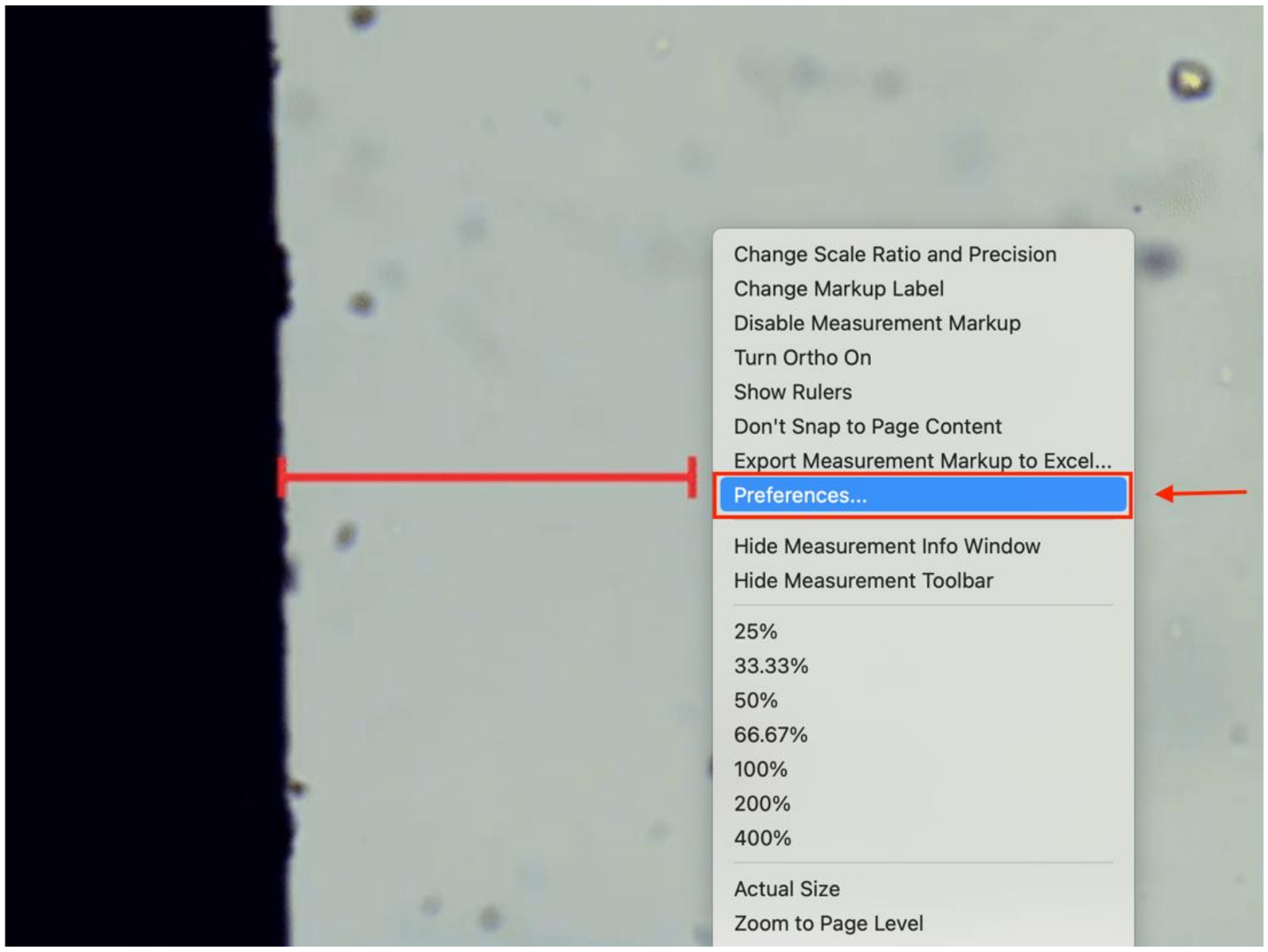

Figure S9. Demonstrating how to make the applied changes as default for Adobe Acrobat.

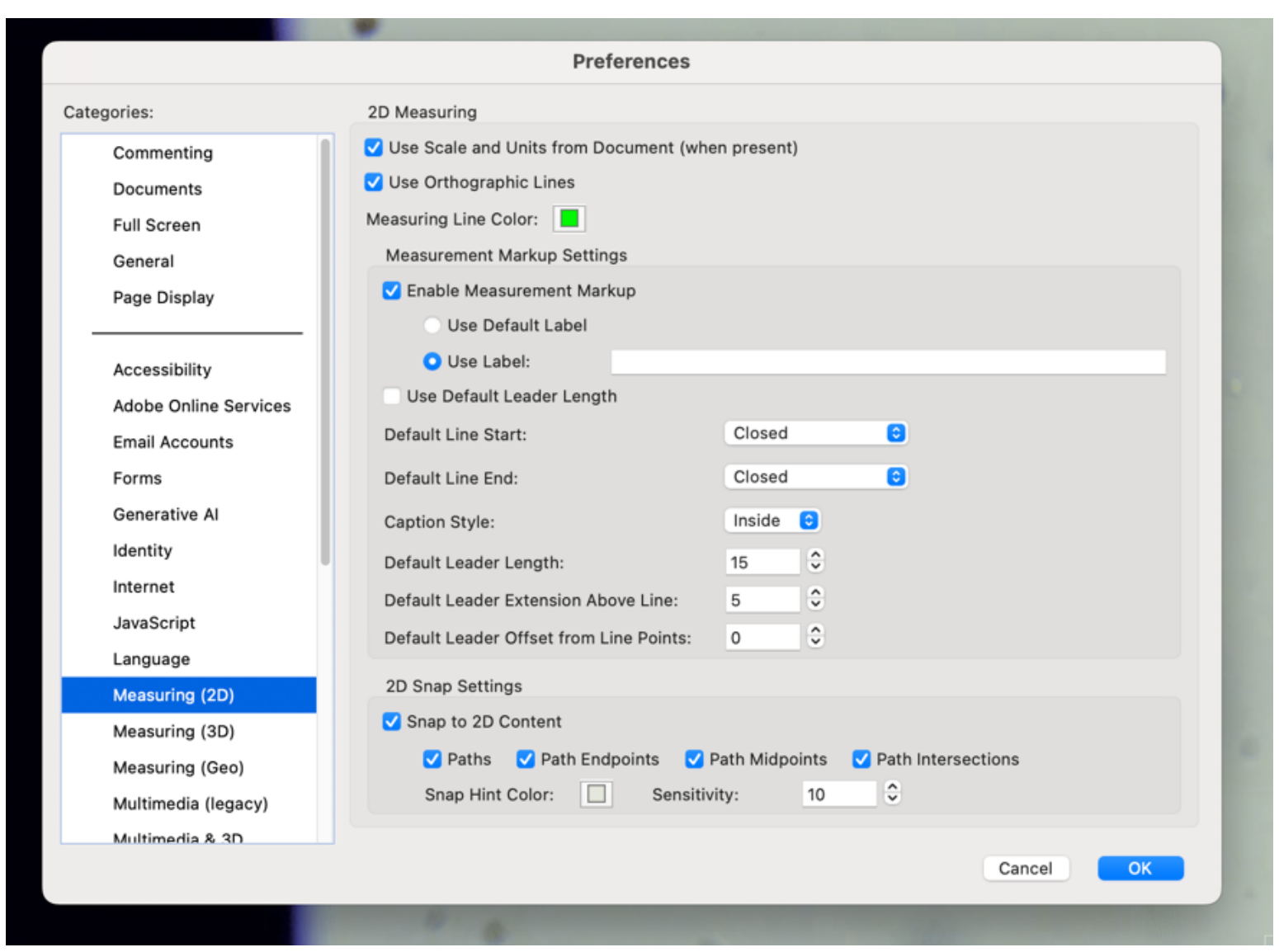

Figure S10. Demonstration of changing the line color for measuring tool in Adobe Acrobat.

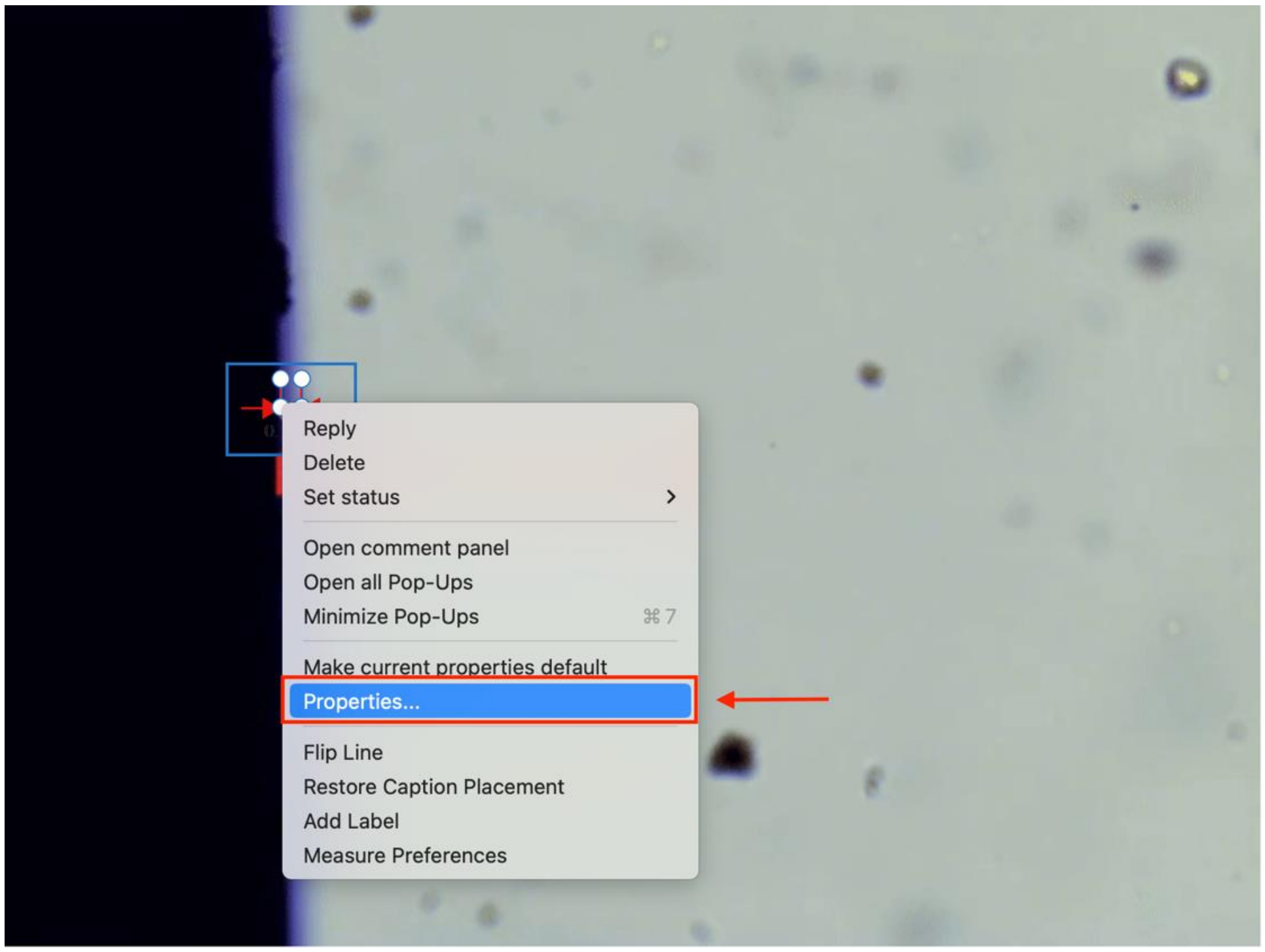

Figure S11. Demonstration of changing the line color for measuring tool in Adobe Acrobat.

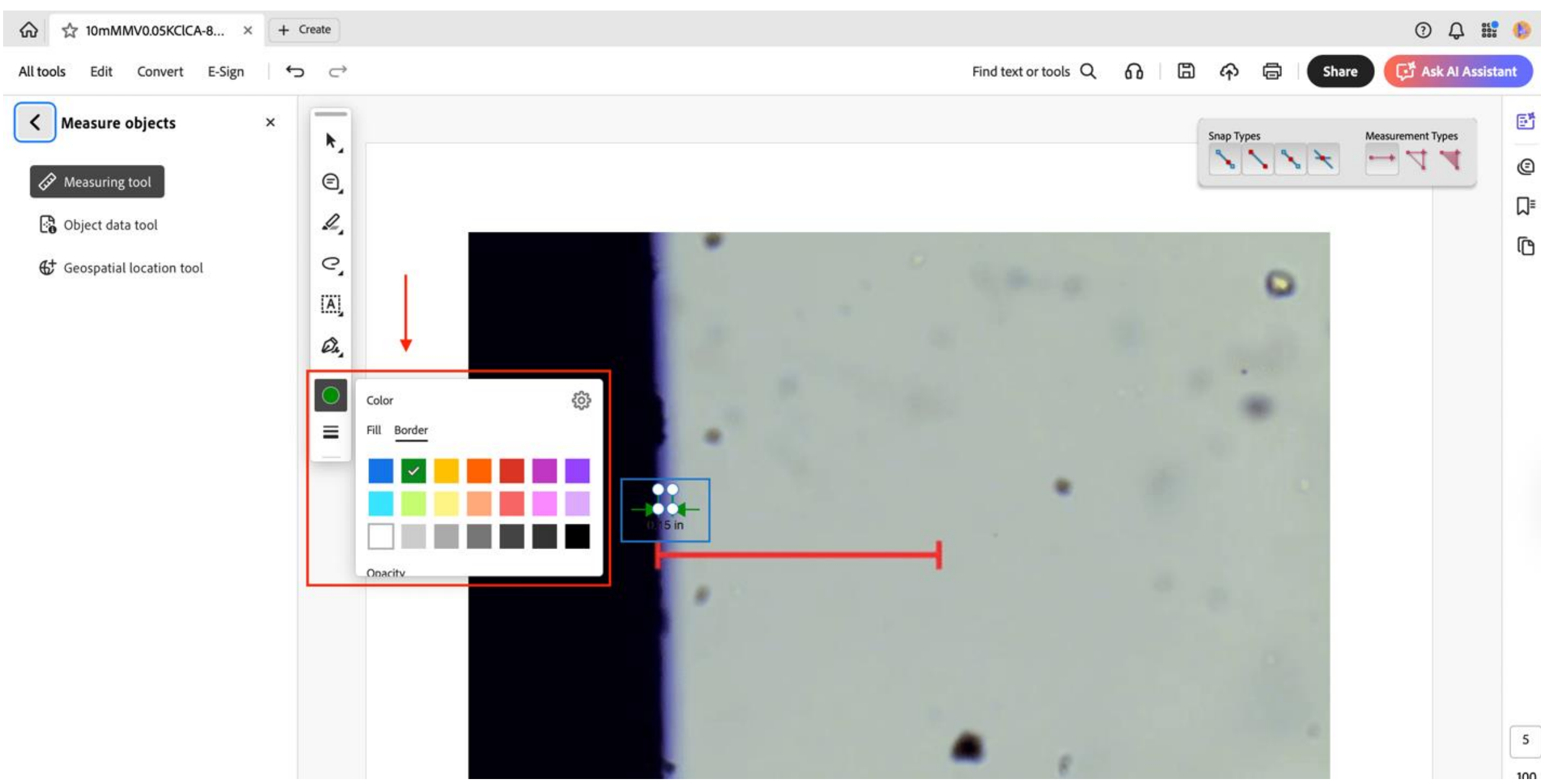

Figure S12. Demonstration of changing the line color for measuring tool in Adobe Acrobat.

You can either **add a label** to indicate each page's time or directly enter it into Excel as demonstrated in Figure S13.

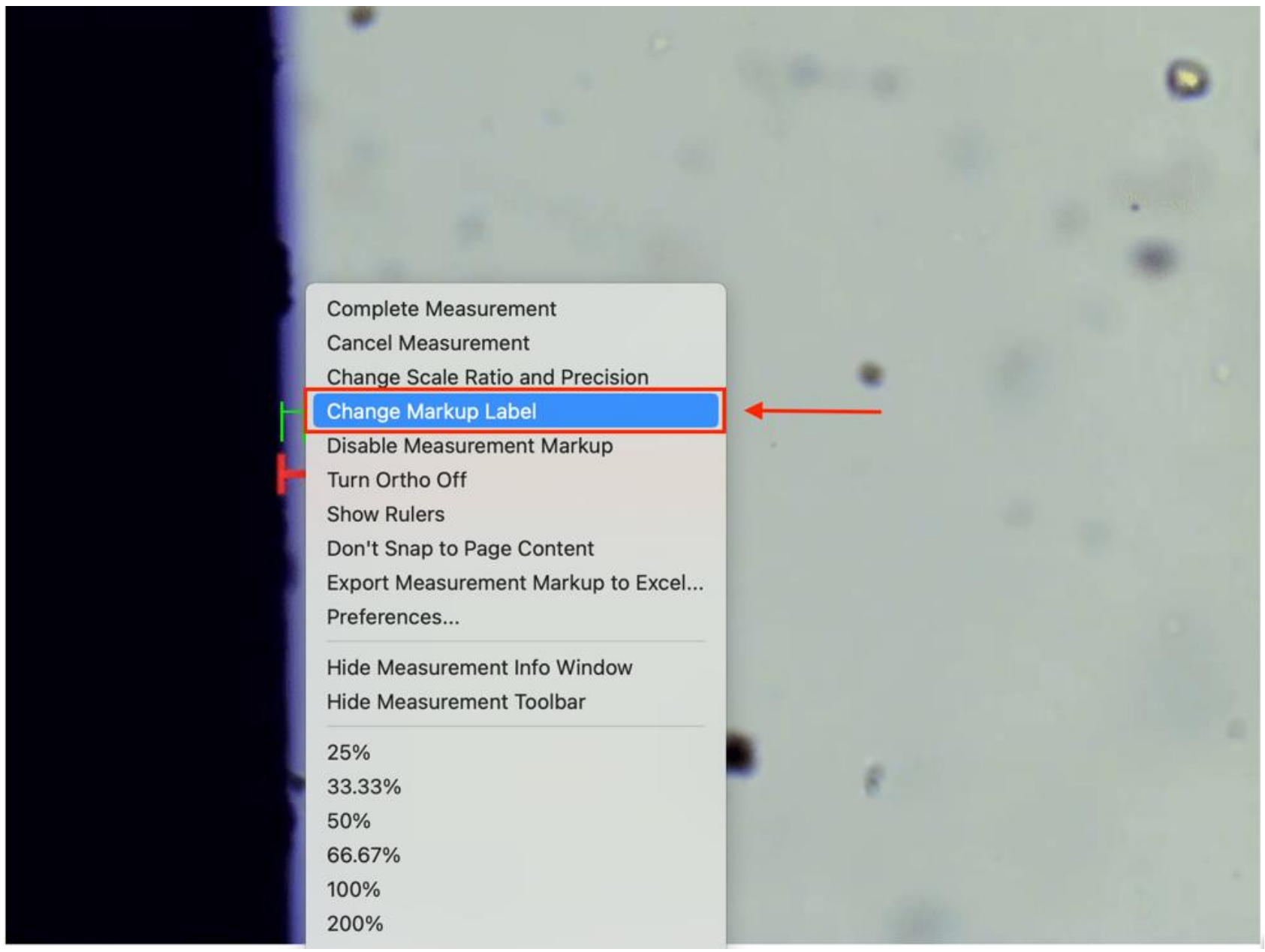

Figure S13. Demonstration of adding the measured value as a label in Adobe Acrobat.

l) When the measurement is complete, you can right-click and then **select "Export Measurement Markup to Excel"** to save and export the results as demonstrated in Figure S14 and S15.

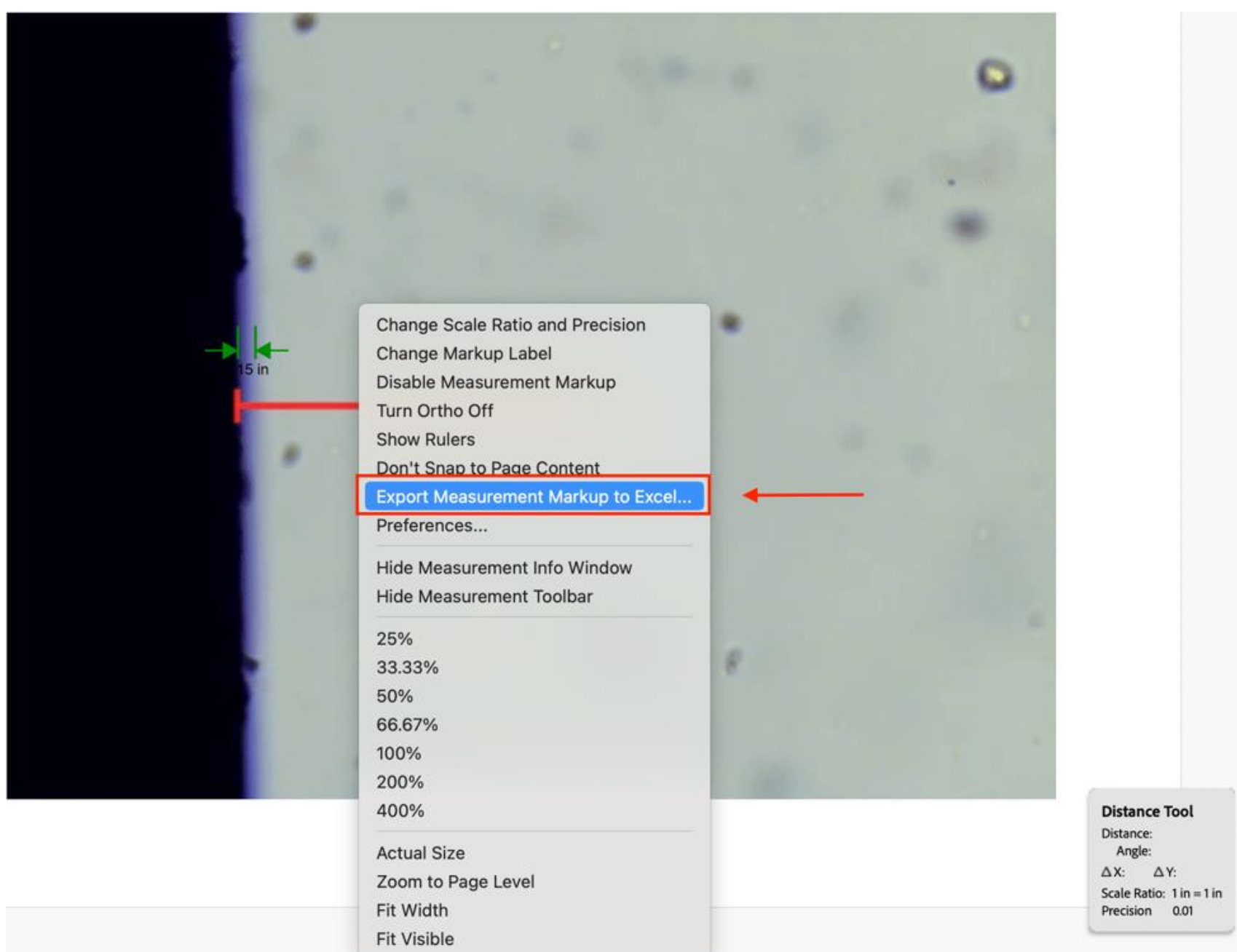

Figure S14. Demonstration of exporting the measured value using measuring tool in Adobe Acrobat.

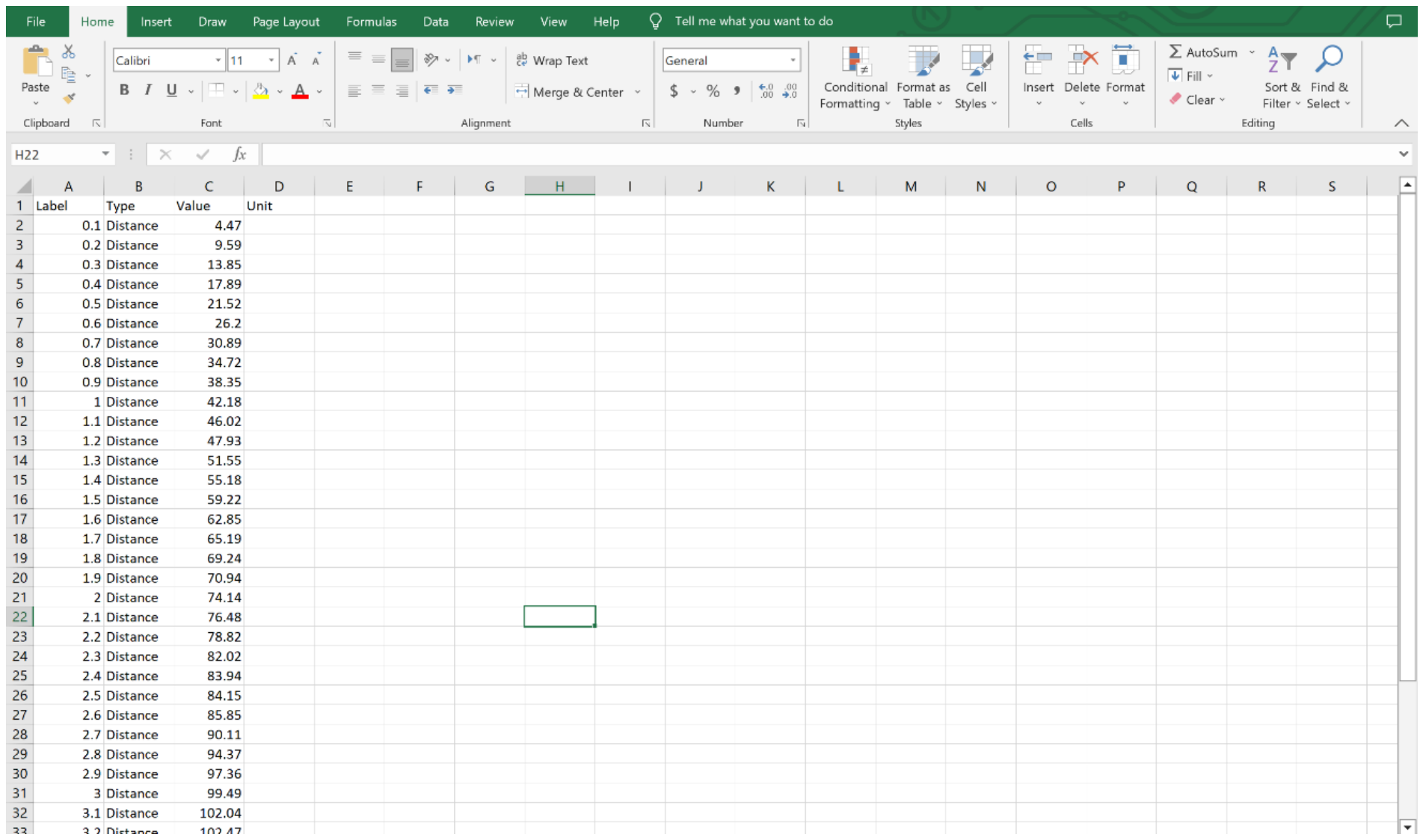

	Label	Type	Value	Unit
2	0.1	Distance	4.47	
3	0.2	Distance	9.59	
4	0.3	Distance	13.85	
5	0.4	Distance	17.89	
6	0.5	Distance	21.52	
7	0.6	Distance	26.2	
8	0.7	Distance	30.89	
9	0.8	Distance	34.72	
10	0.9	Distance	38.35	
11	1	Distance	42.18	
12	1.1	Distance	46.02	
13	1.2	Distance	47.93	
14	1.3	Distance	51.55	
15	1.4	Distance	55.18	
16	1.5	Distance	59.22	
17	1.6	Distance	62.85	
18	1.7	Distance	65.19	
19	1.8	Distance	69.24	
20	1.9	Distance	70.94	
21	2	Distance	74.14	
22	2.1	Distance	76.48	
23	2.2	Distance	78.82	
24	2.3	Distance	82.02	
25	2.4	Distance	83.94	
26	2.5	Distance	84.15	
27	2.6	Distance	85.85	
28	2.7	Distance	90.11	
29	2.8	Distance	94.37	
30	2.9	Distance	97.36	
31	3	Distance	99.49	
32	3.1	Distance	102.04	

Figure S15. Example of exported data (opened by Excel) after using the measuring tool in Adobe Acrobat.

m) After exporting the data, the thickness values (Column C) must be copied to the **Analysis Chart Blank** (the Excel file) and then replaced with Column L (**$\bar{\Delta}$ µm)** and then save the changes made.

9. Example of Results:

The results and plots collected during the experimental procedure are shown in Figures S16. The calculated slope and D value corresponding with the calibration plots (R^2) are presented in Table S1.

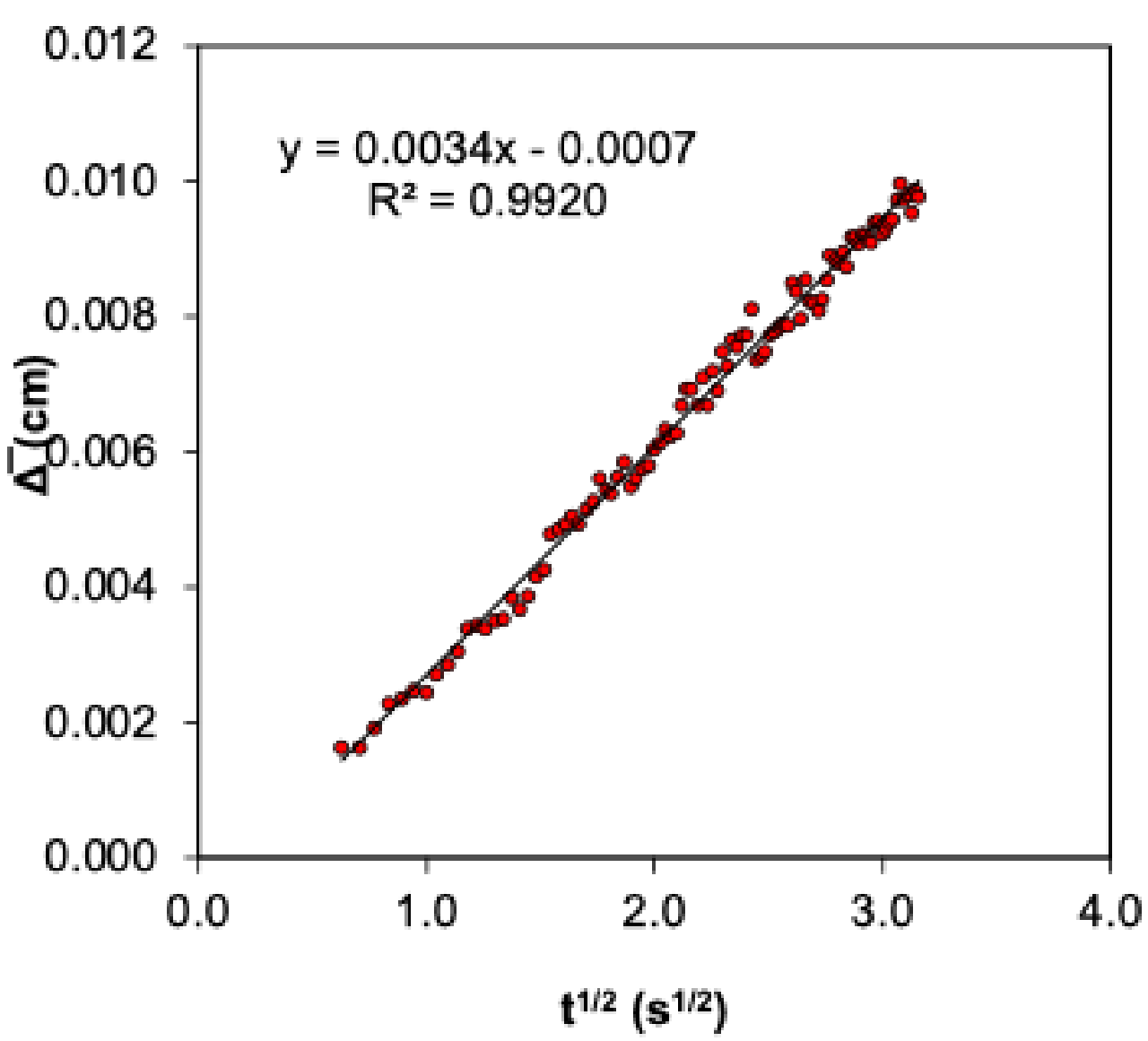

Figure S16. Example of derived curve with equation and R^2 value after plotting the thickness of diffusion layer vs square root of time, for 10 mM MV solution.

Table S1: Charactristics of the plots derived by thickness of diffusion layer vs square root of time for two independent solutions, 10mV MV solution.

**Test**	**Slope**	**R^2**	**Intercept**	**D (cm^2s^{-1})**
Figure S17	0.003390334	0.992032714	-0.00073642	5.7472E-06

---

1. Khalafi, L.; Nikzad, N.; Alhajeri, A.; Bacon, B.; Alvarado, K.; Rafiee, M. Electrochemistry under Microscope: Observing the Diffusion Layer and Measuring Diffusion Coefficient. *J. Chem. Educ.* **2023**, *100* (10), 4056–4061. https://doi.org/10.1021/acs.jchemed.3c00584.
