# Supplementary material for: Thin-Layer Electrochemistry: Visualizing the Diffusion Layer During Chronoamperometry and Cyclic Voltammetry
Source: J Chem Educ. 2026 Jul 16;103(8):4704–10. doi: 10.1021/acs.jchemed.5c01710 (PMC13472089; doi:10.1021/acs.jchemed.5c01710)

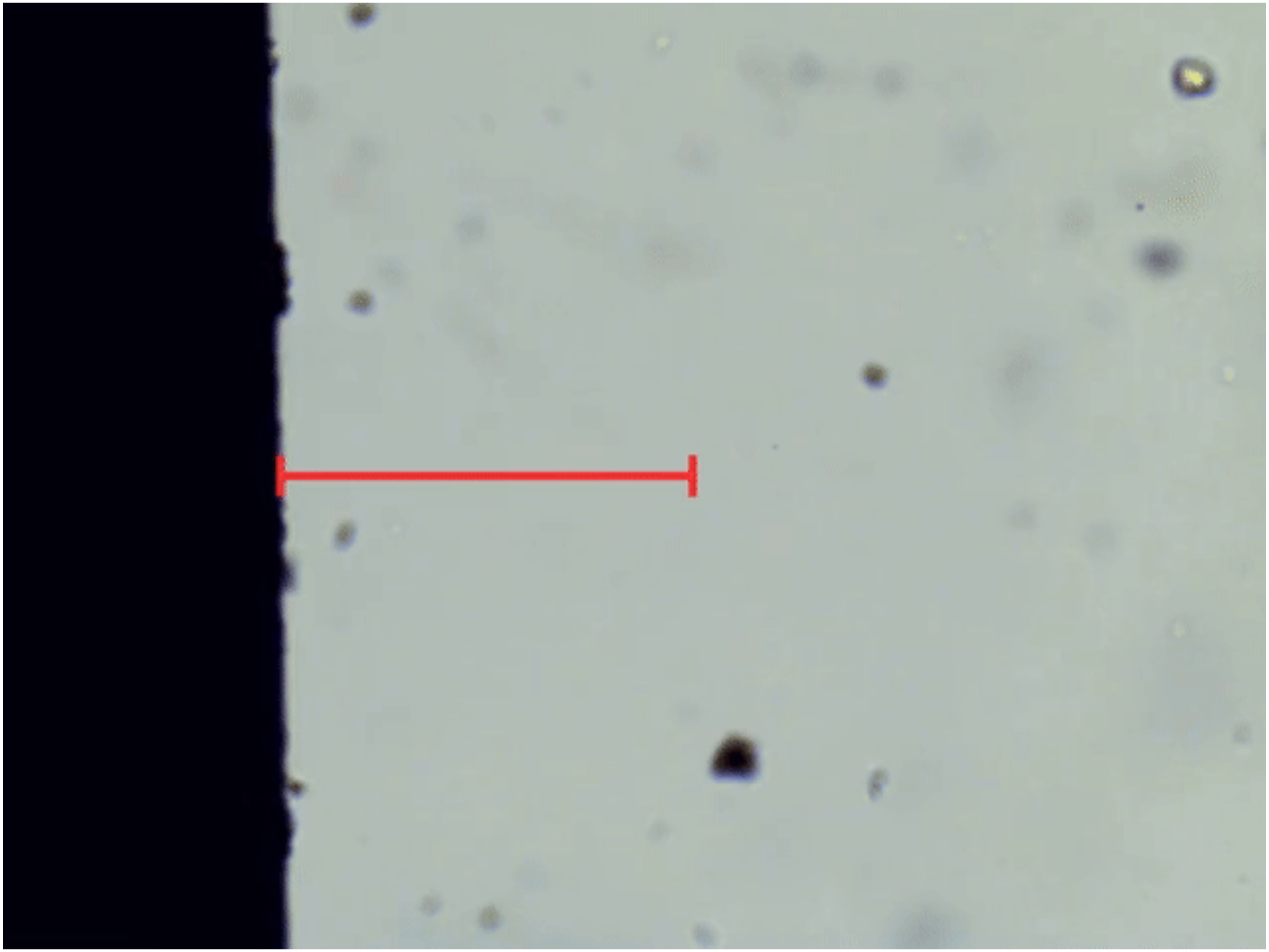

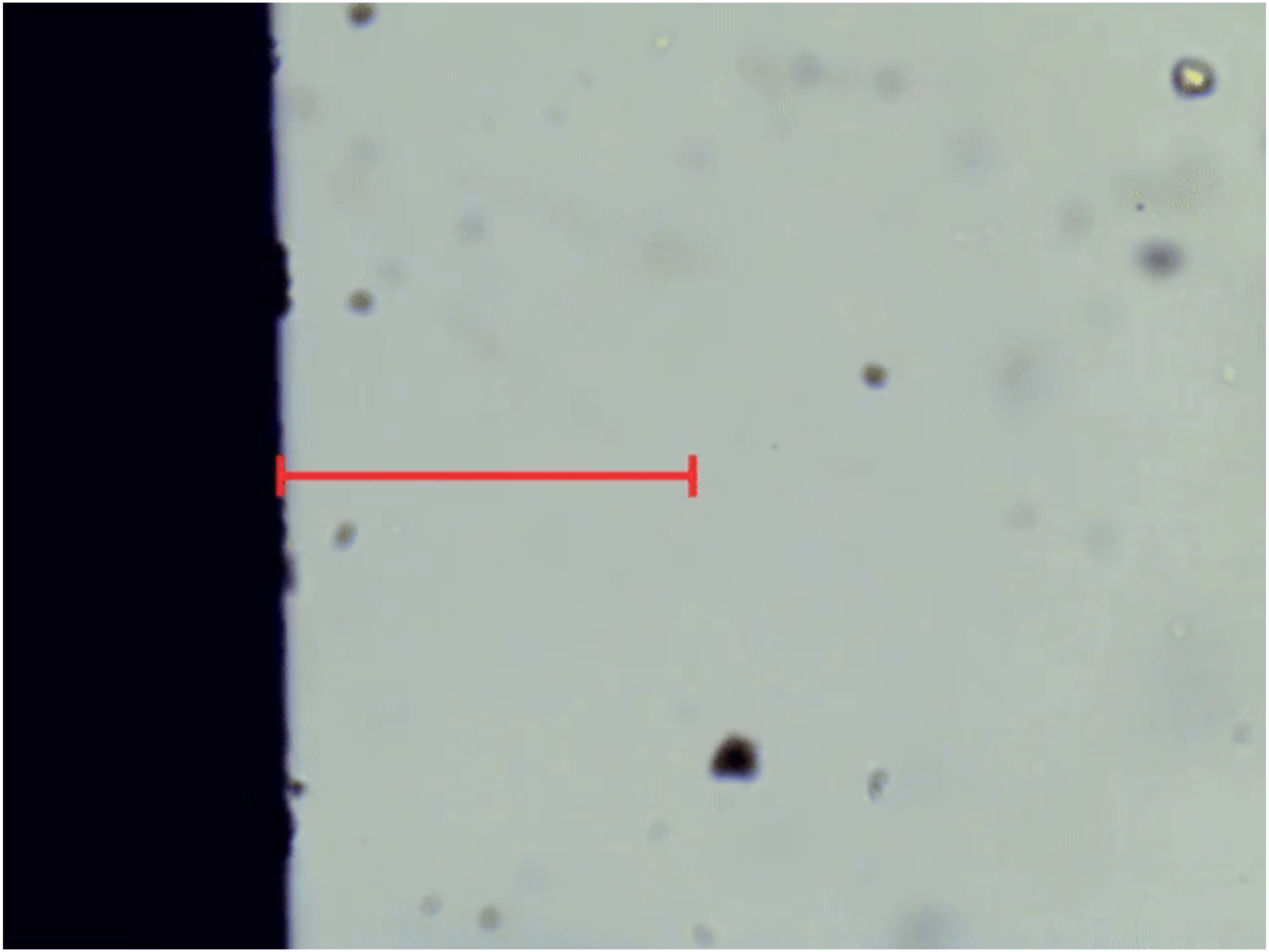

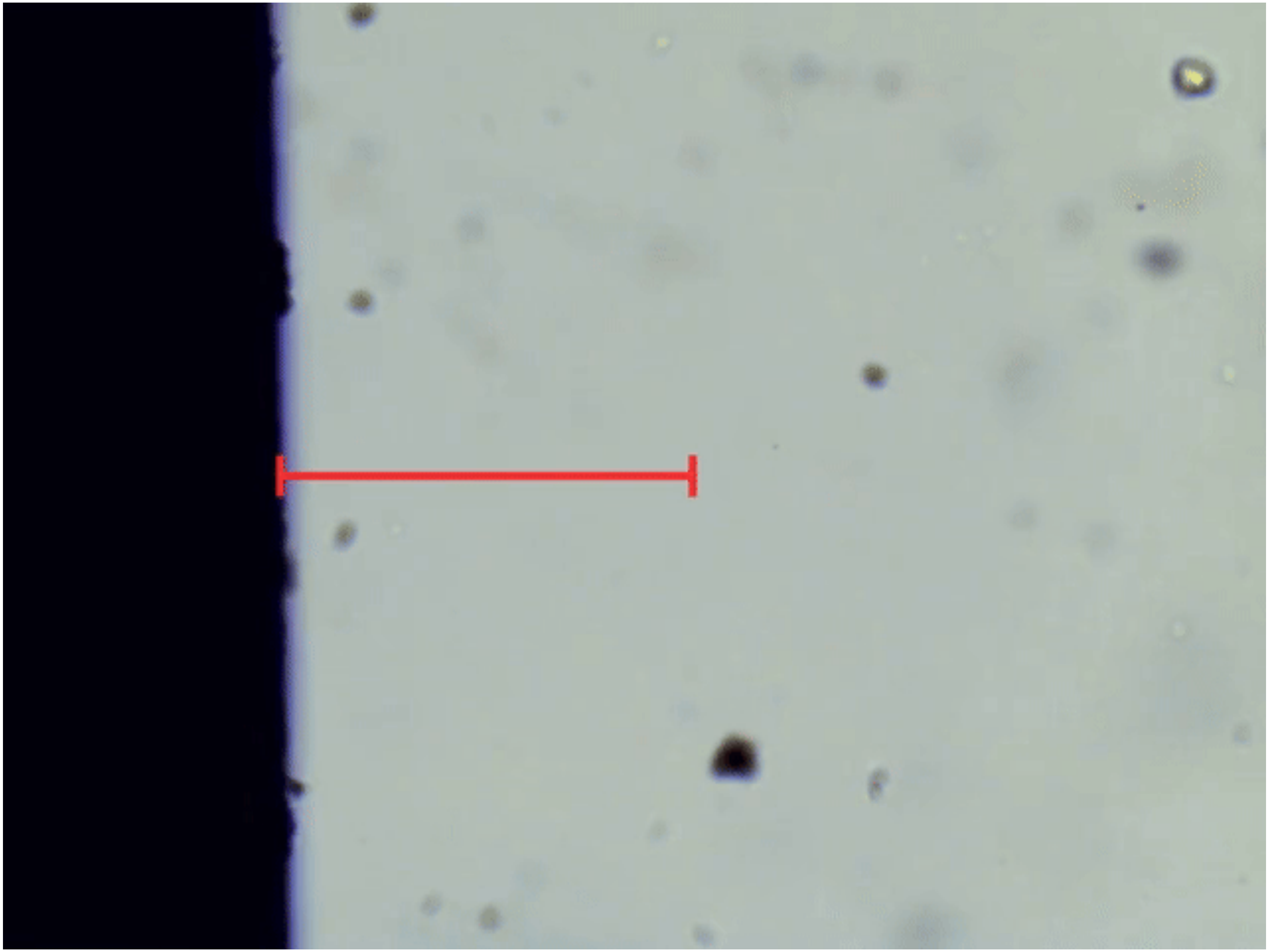

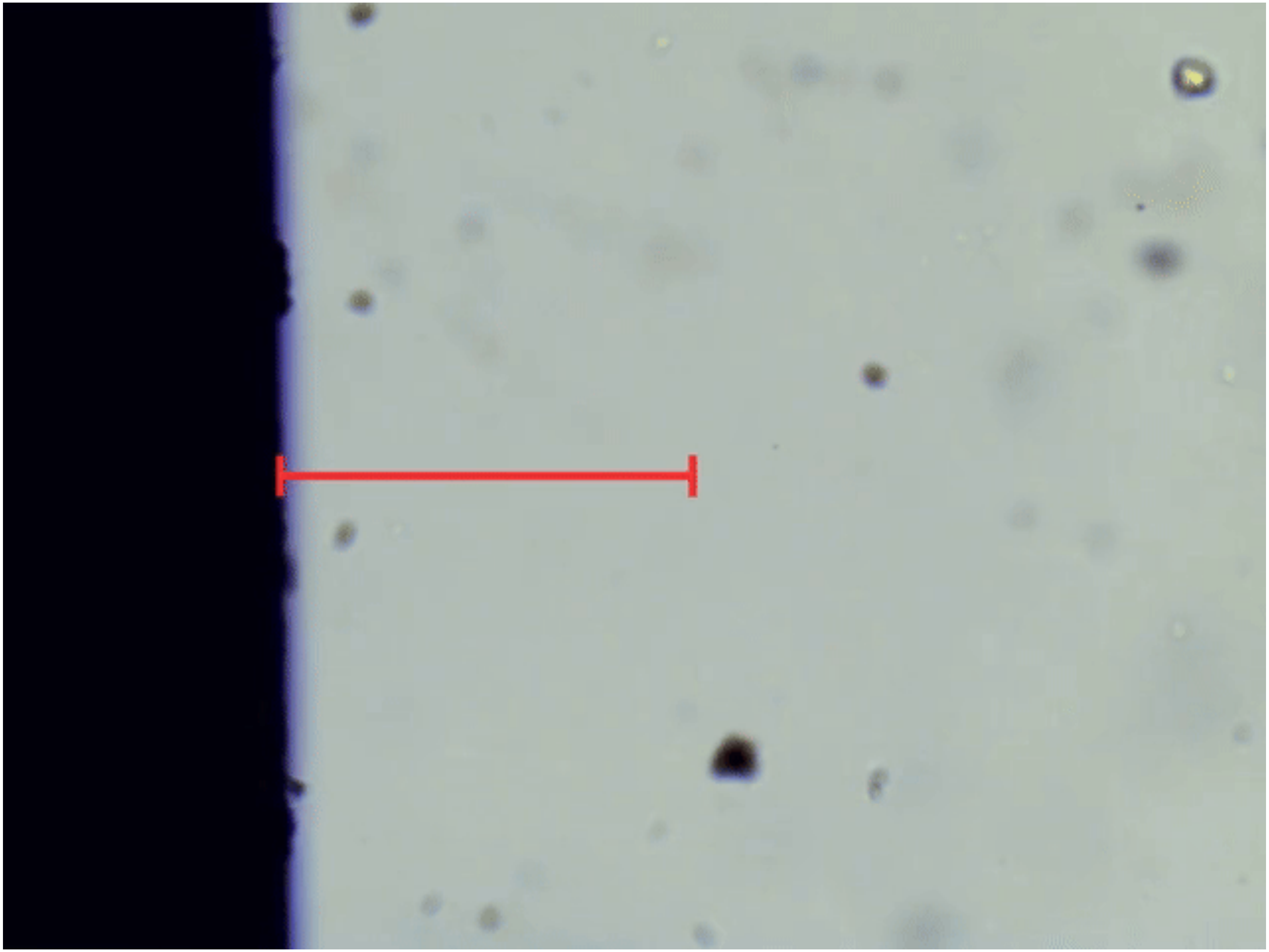

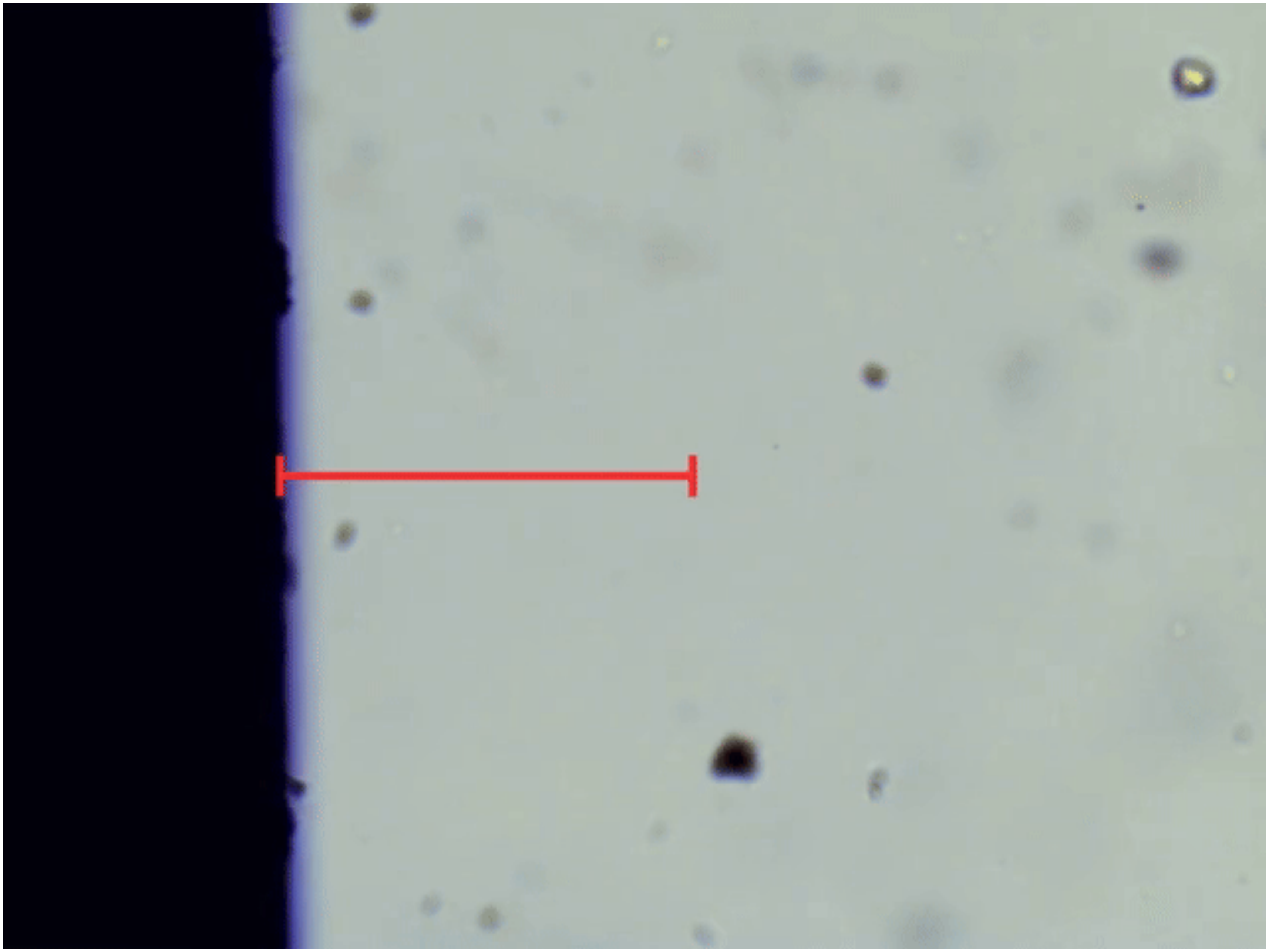

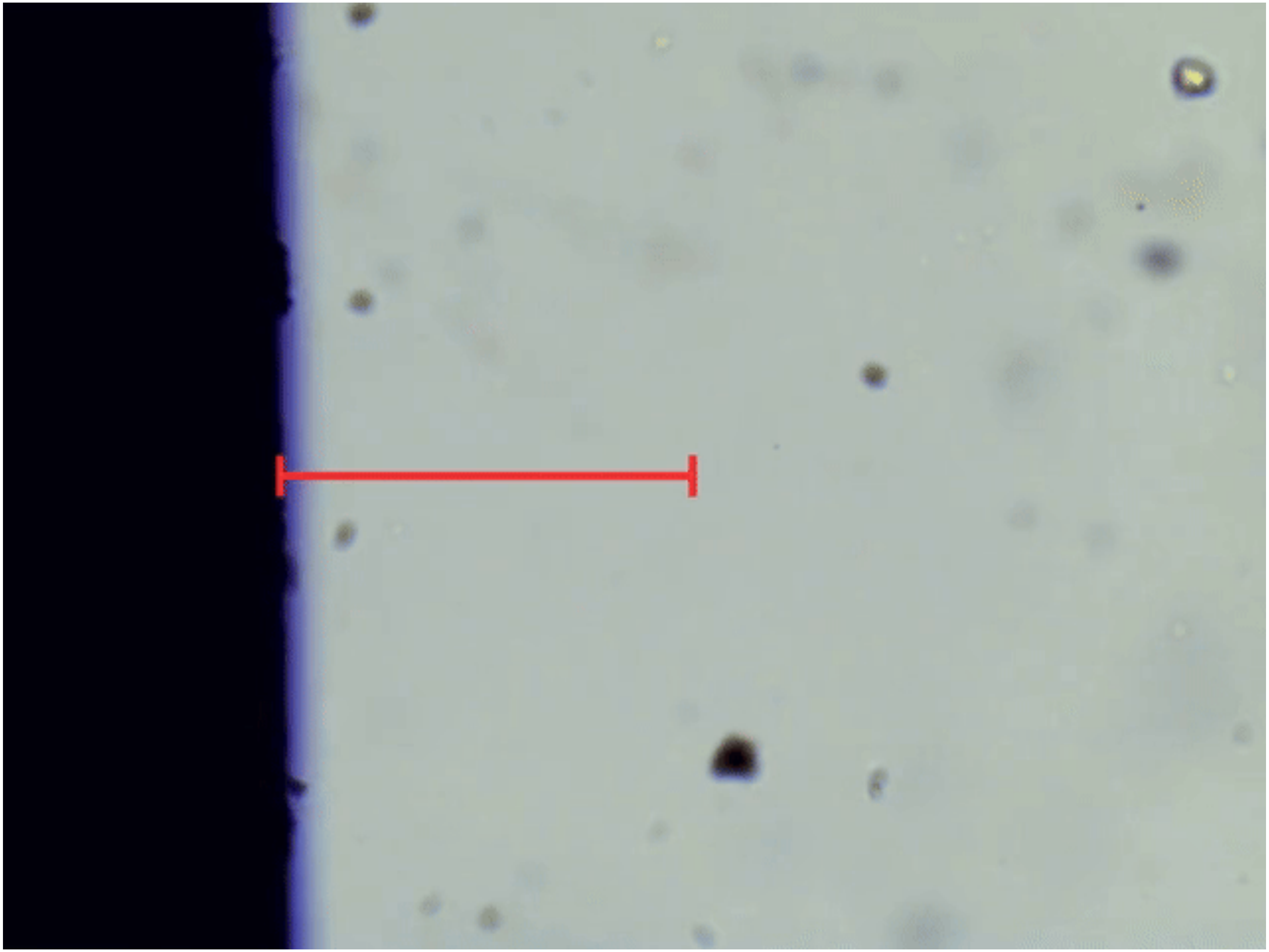

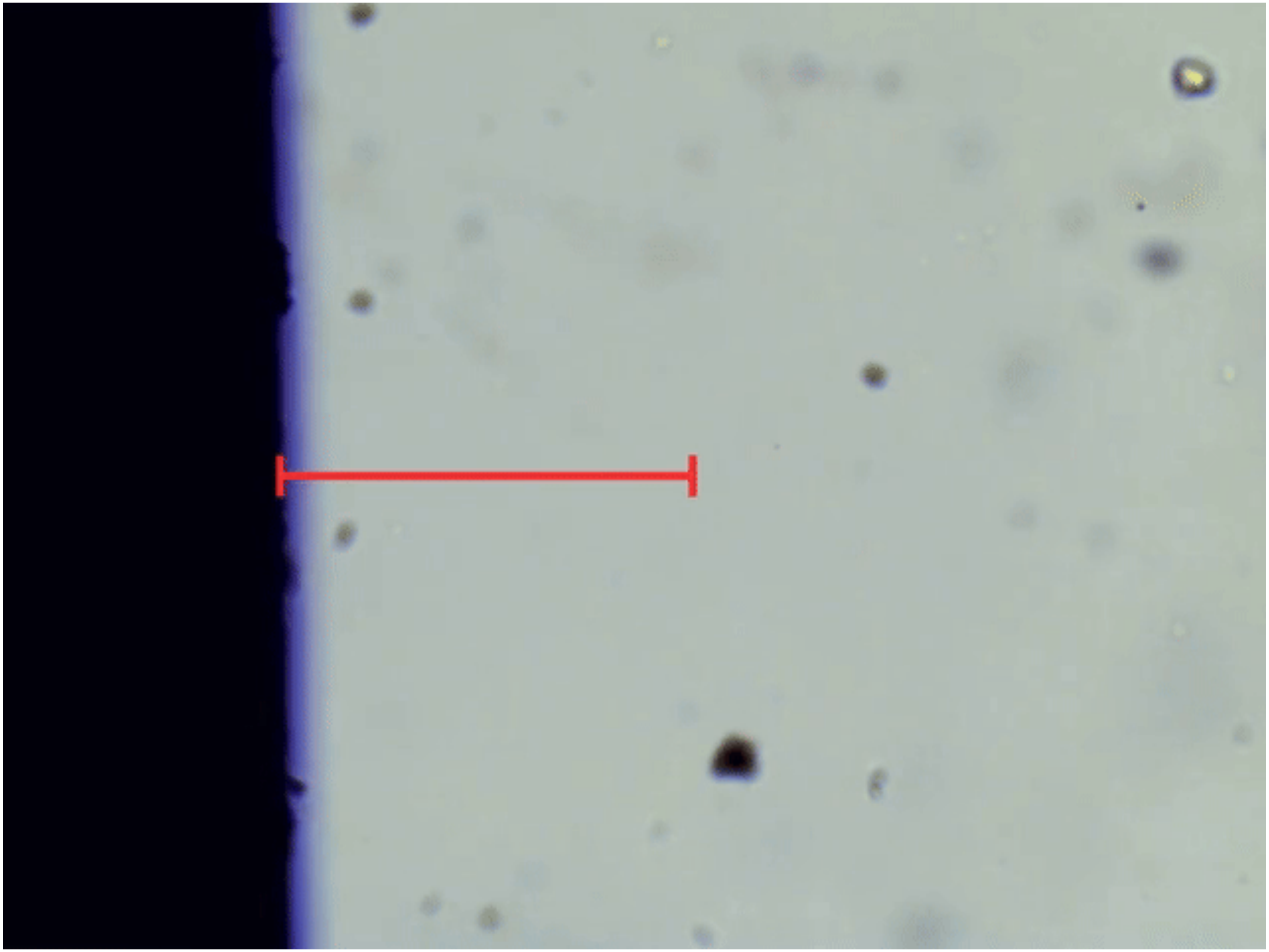

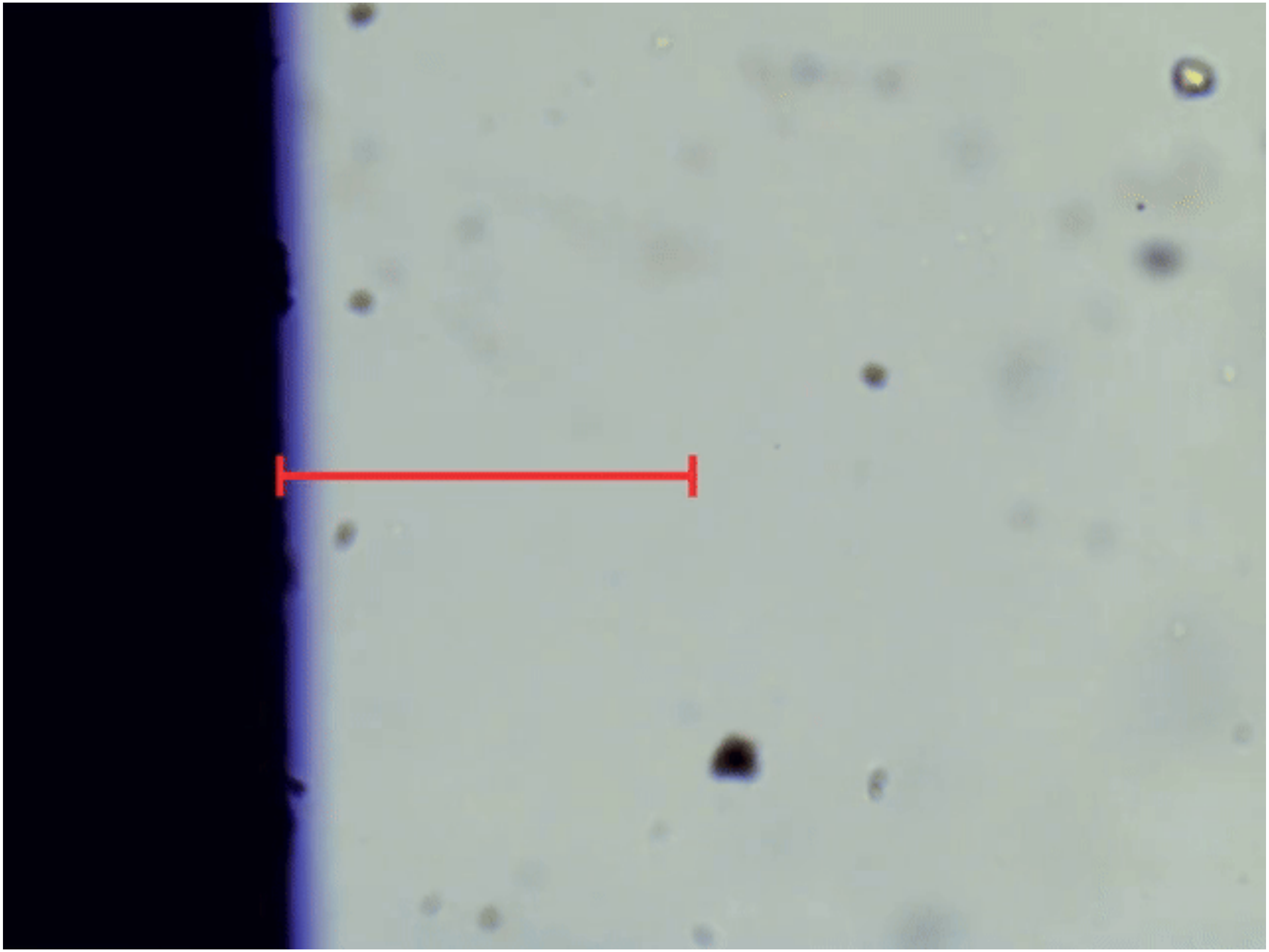

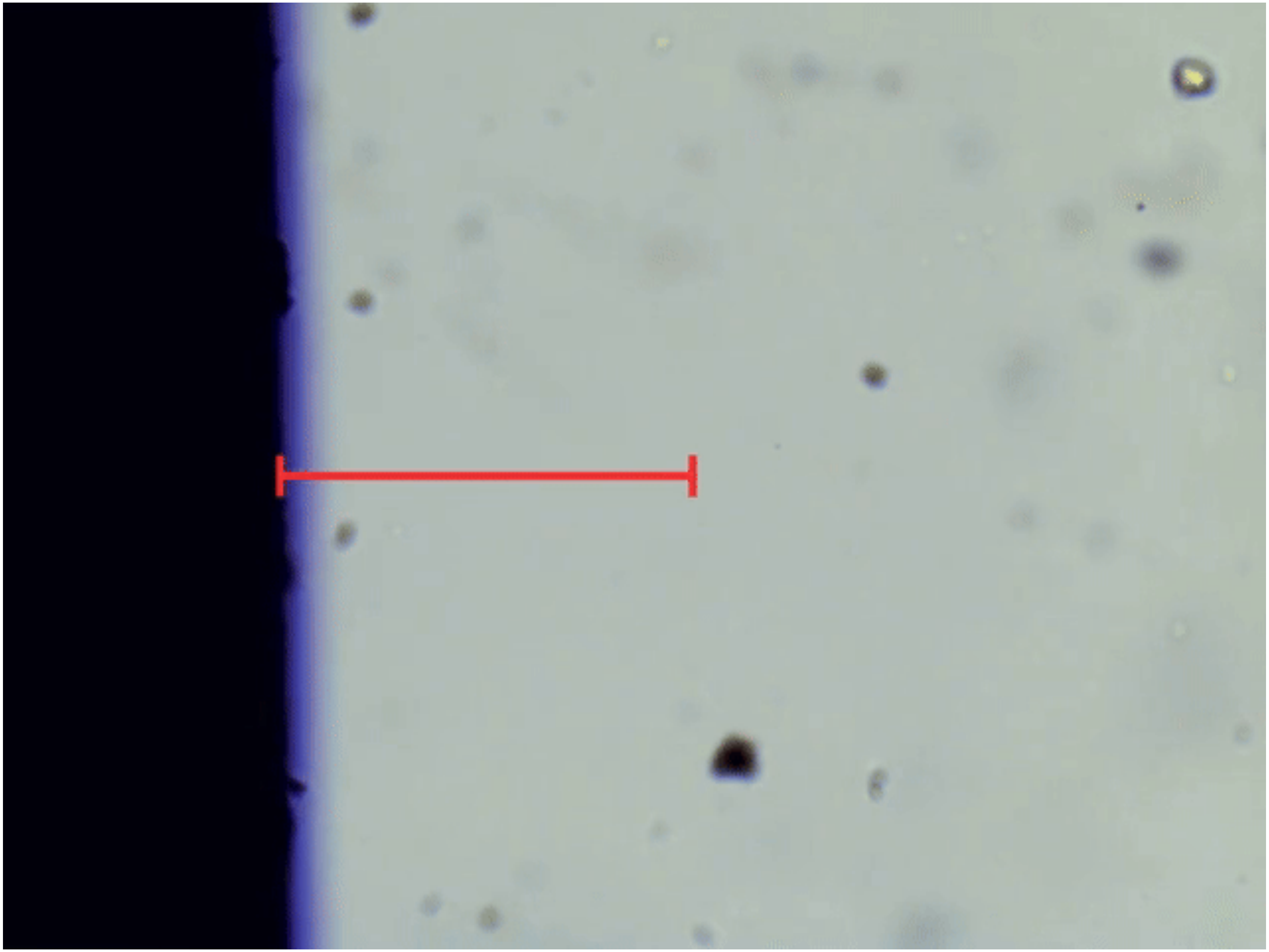

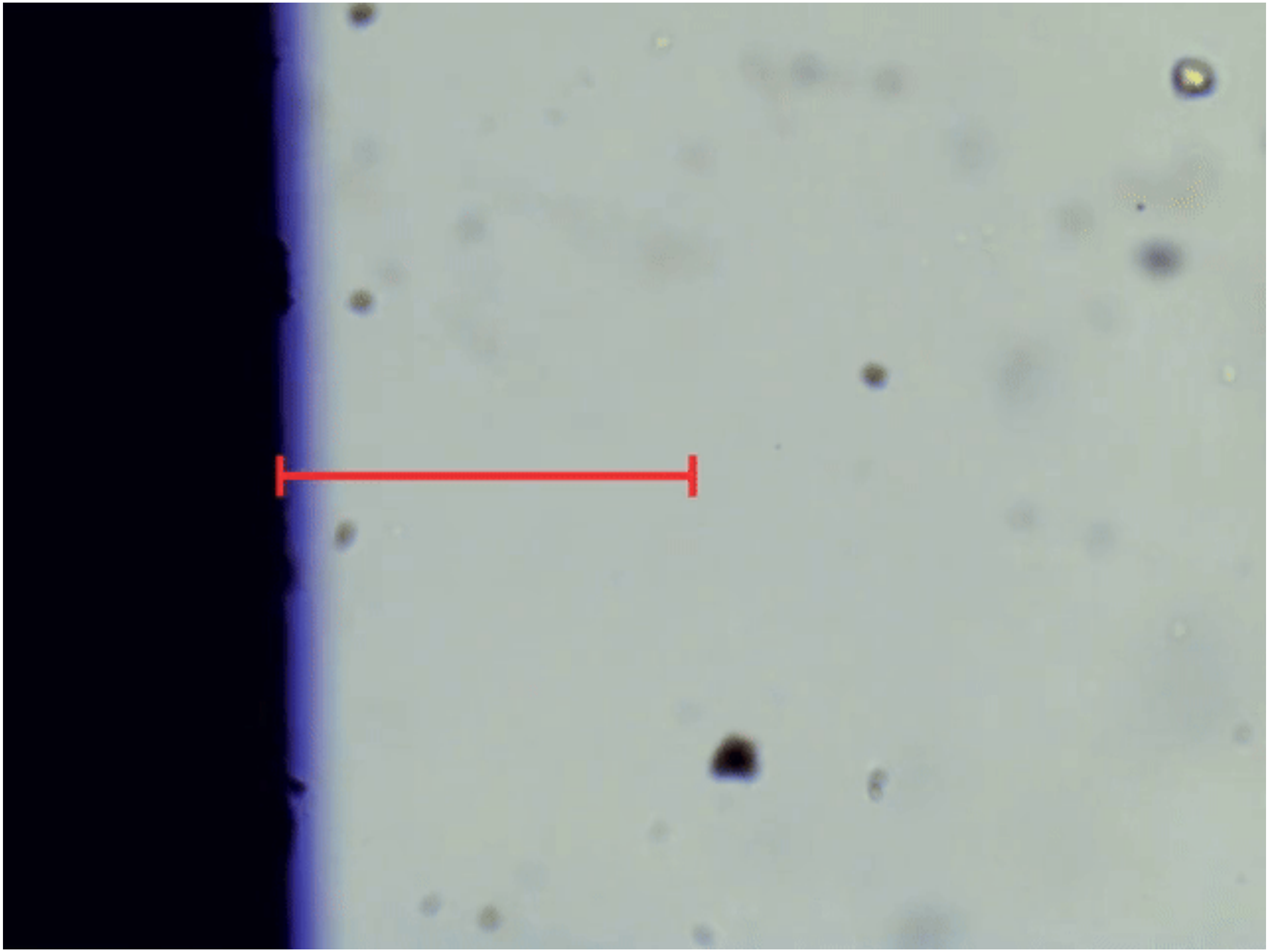

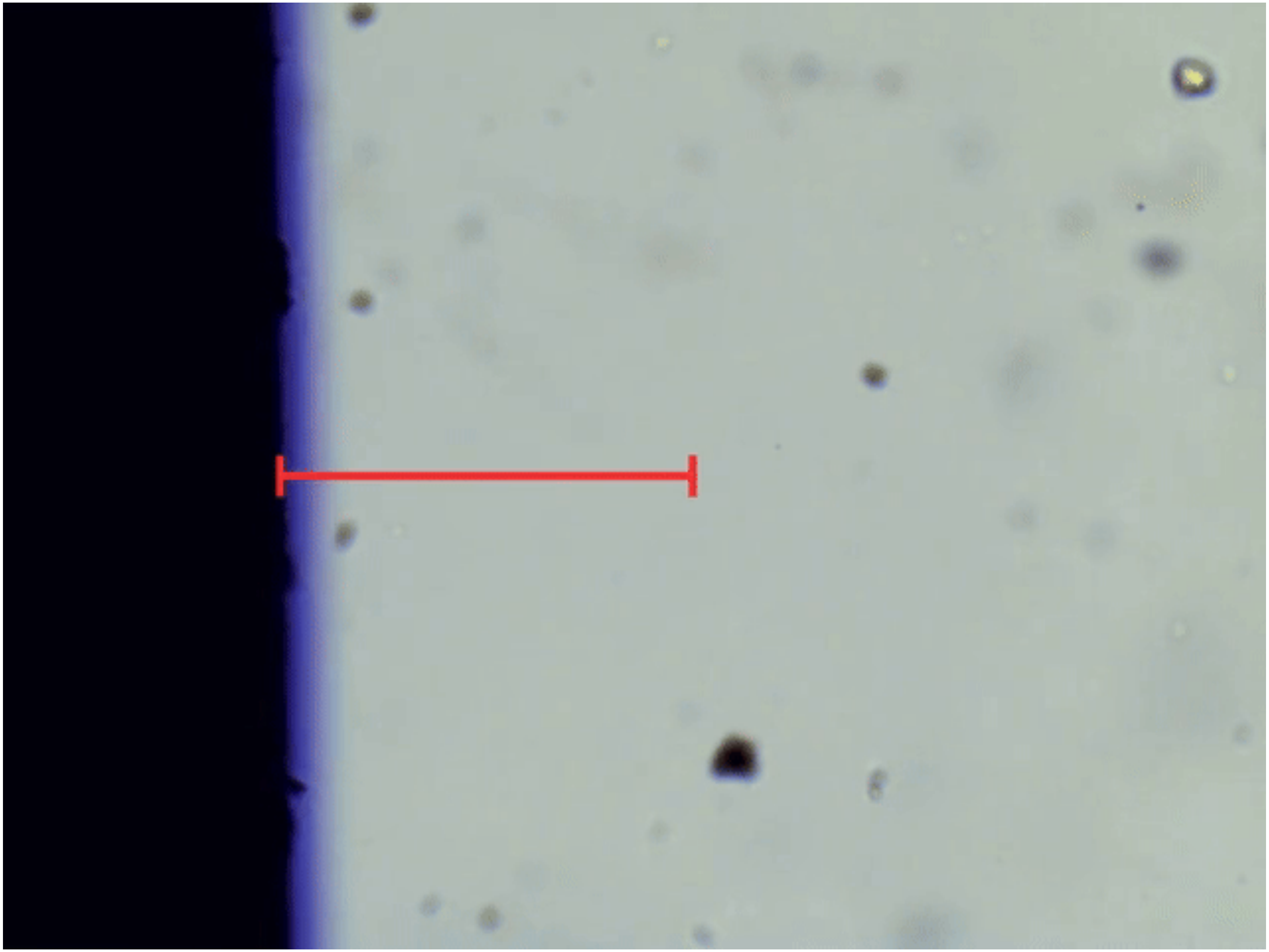

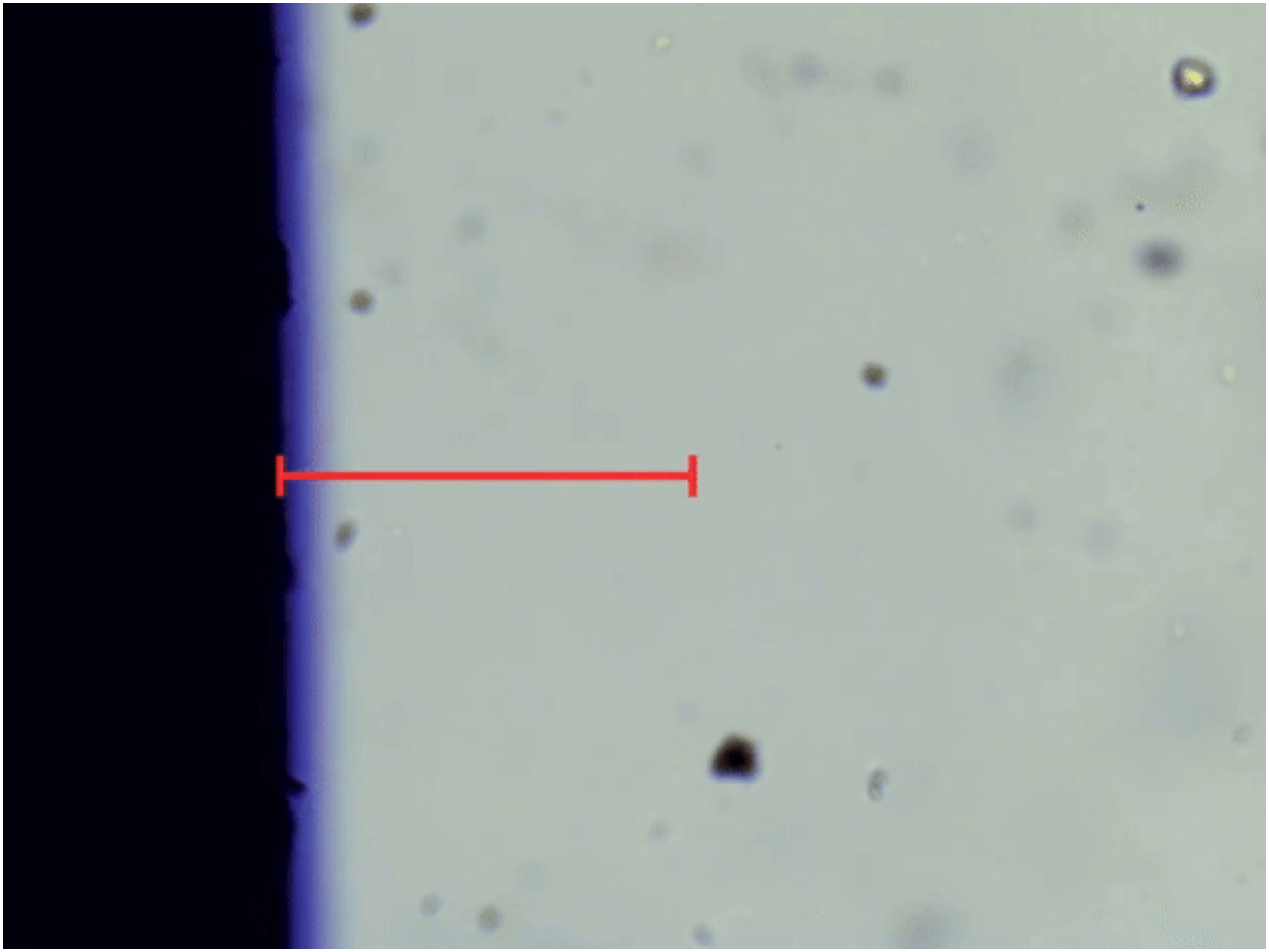

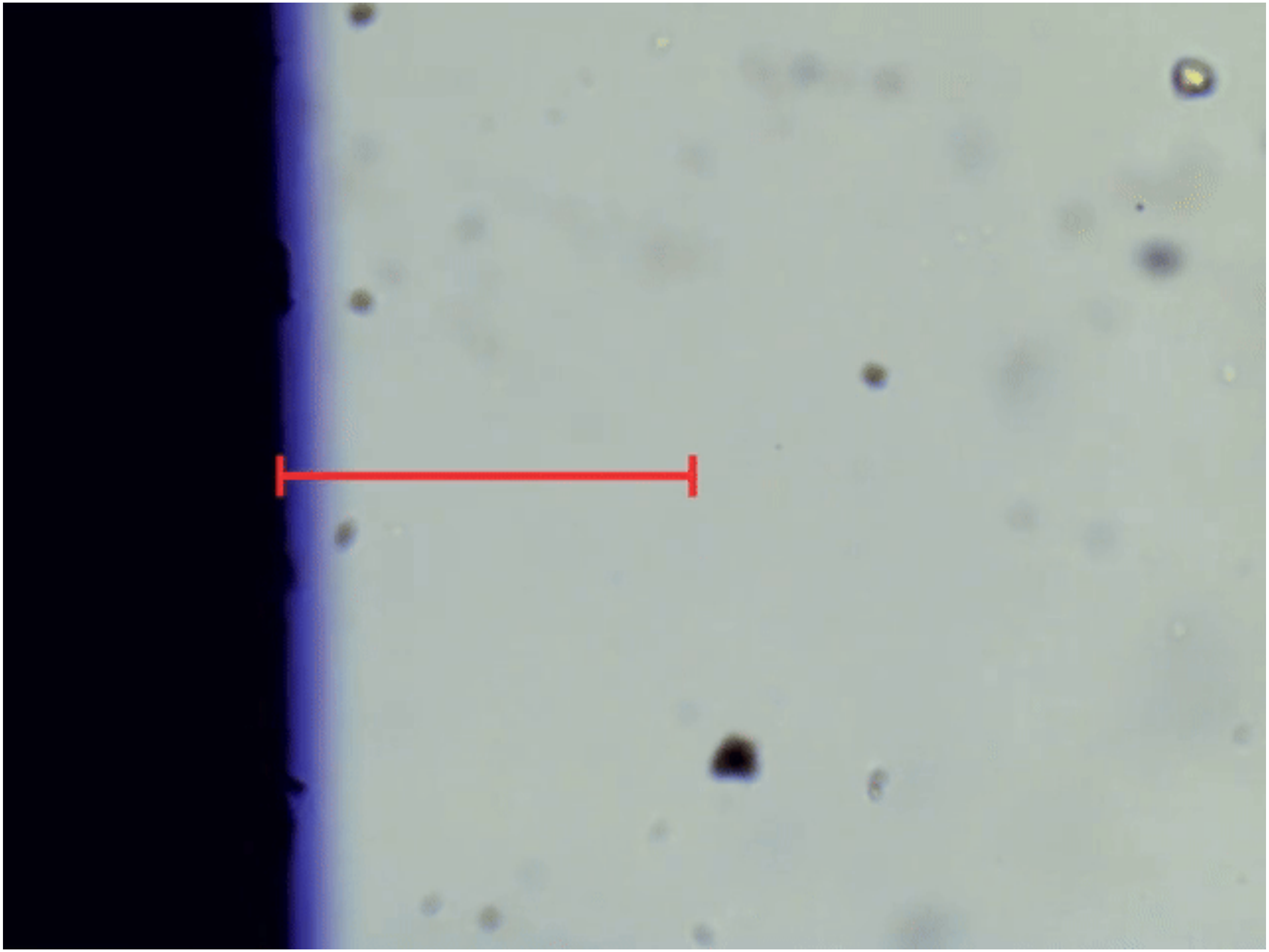

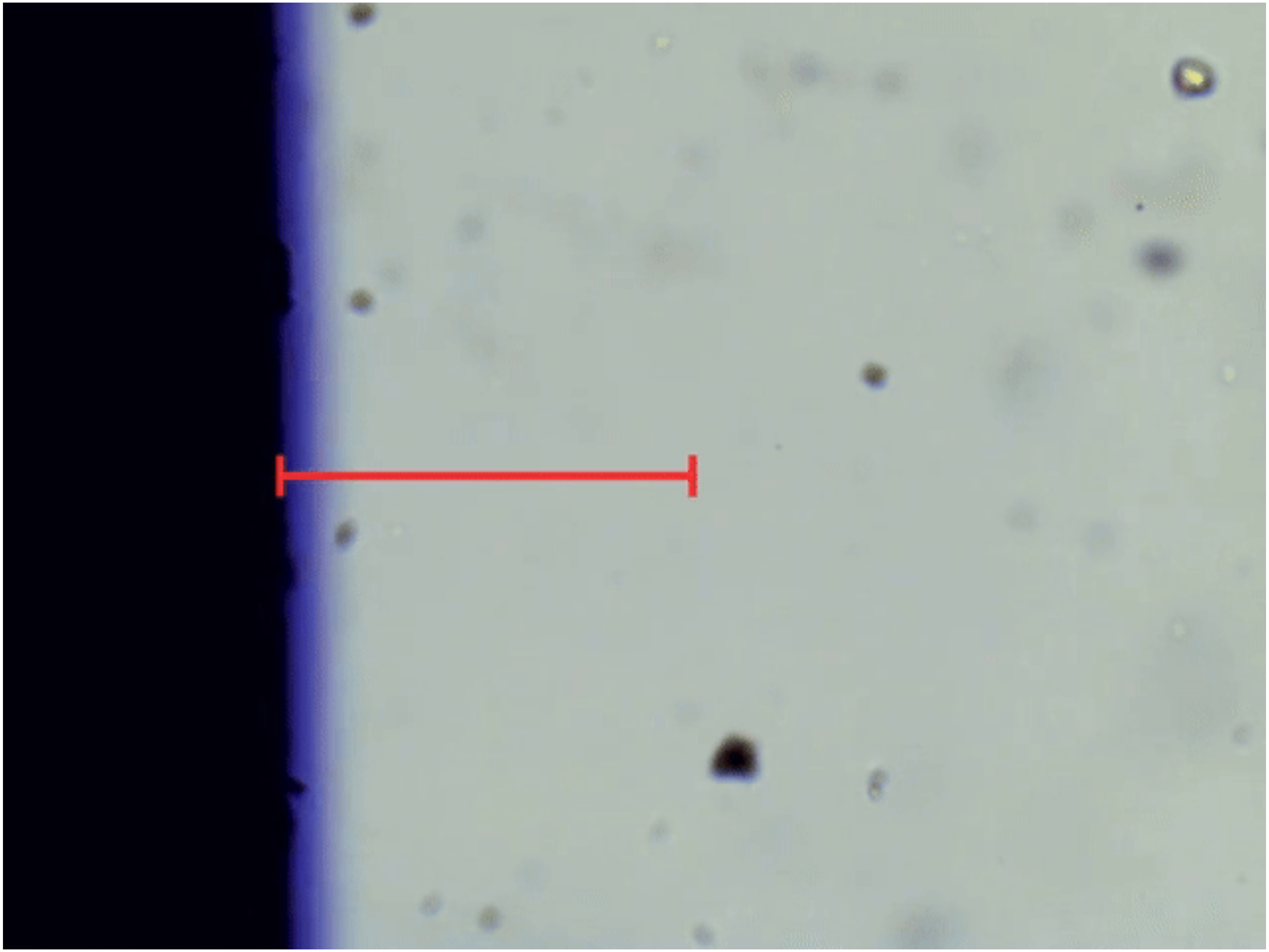

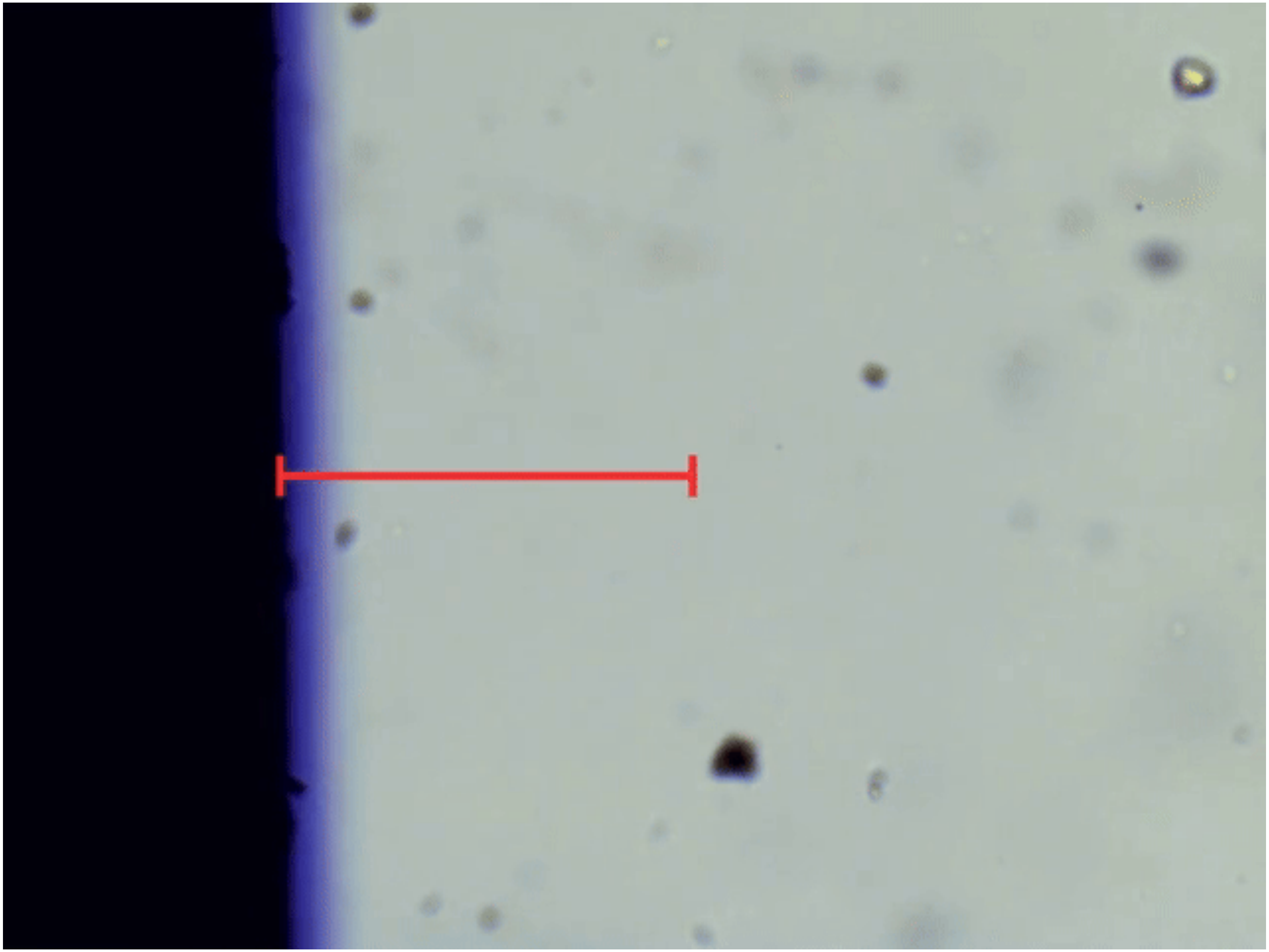

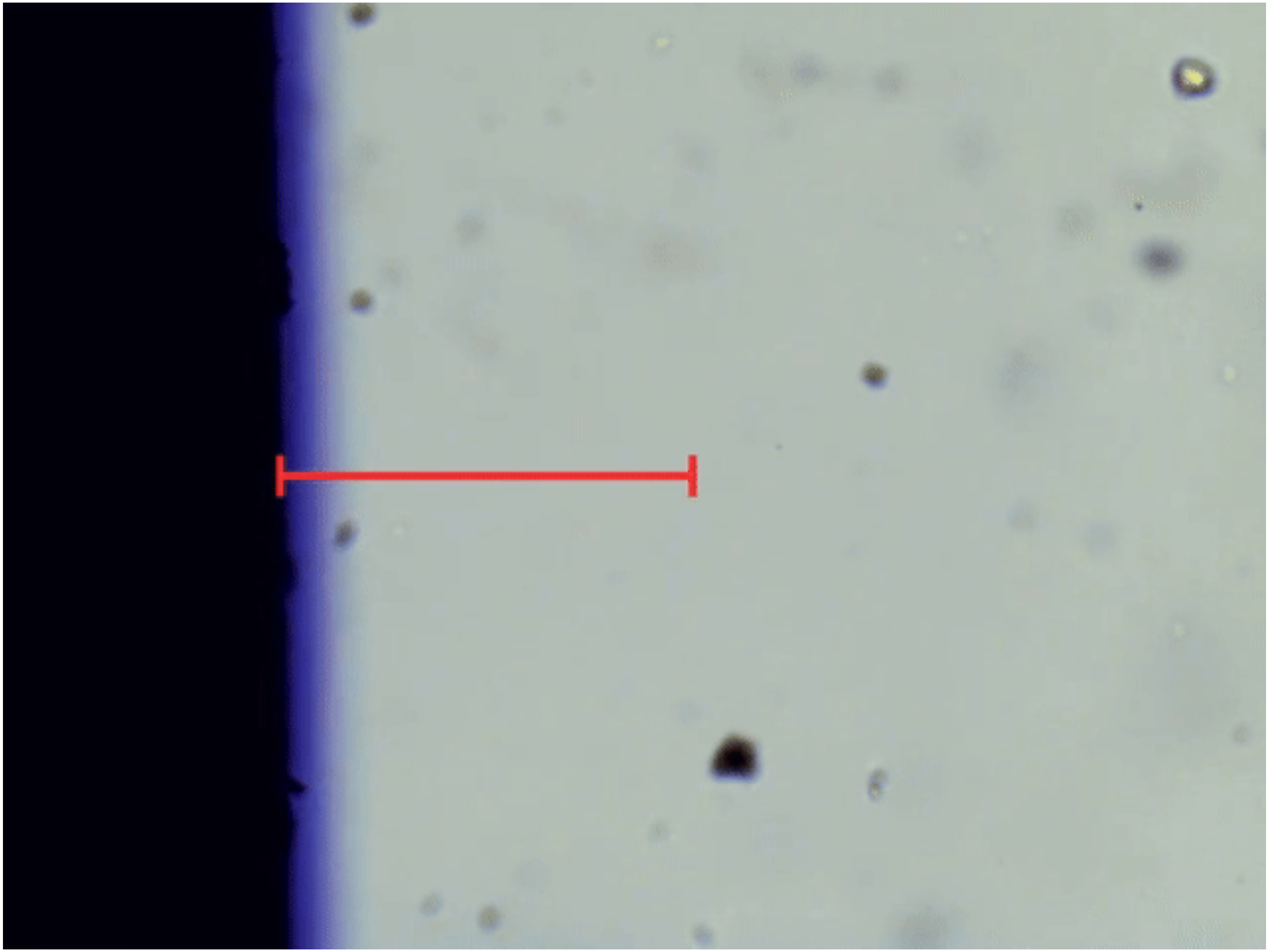

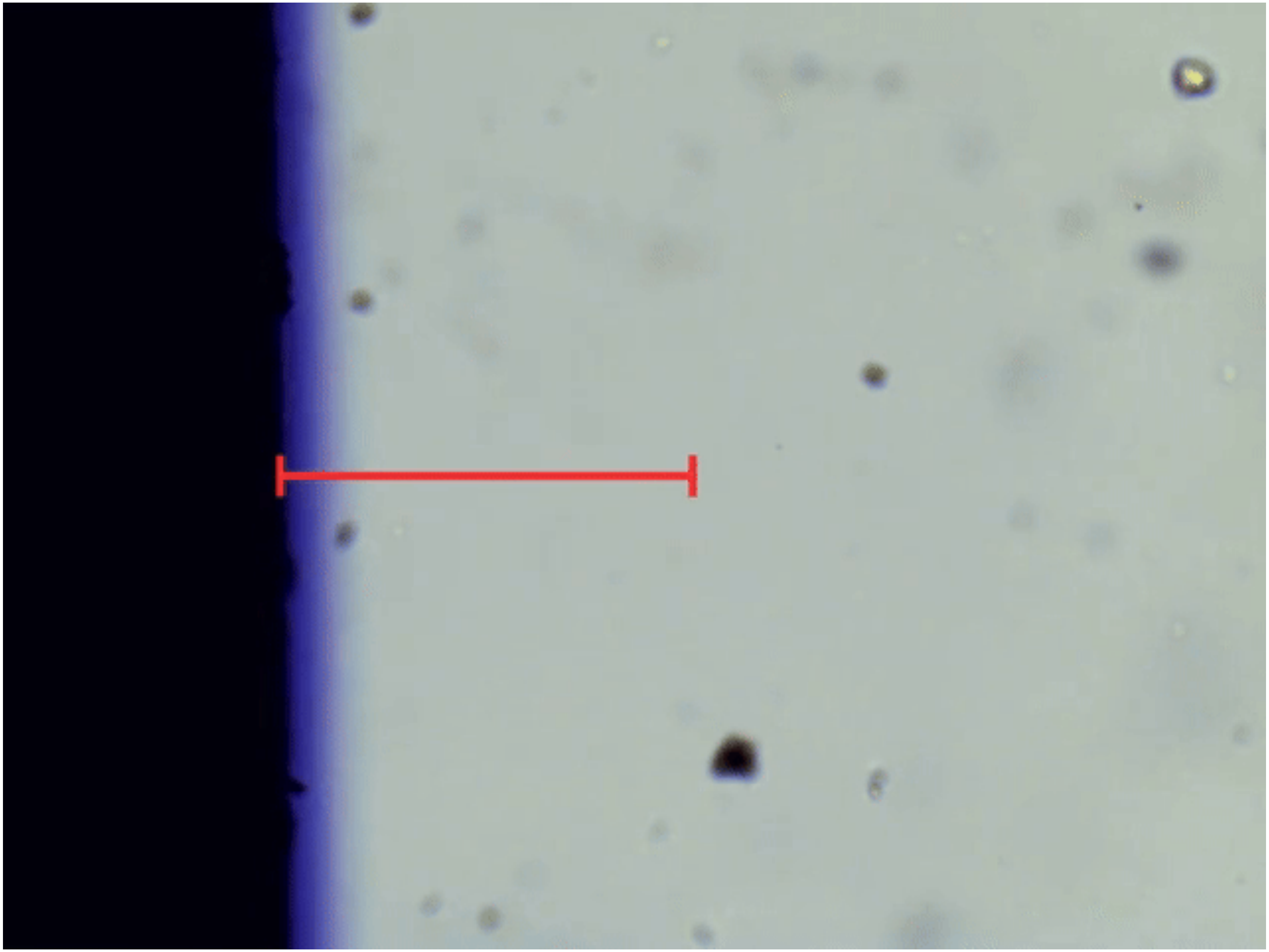

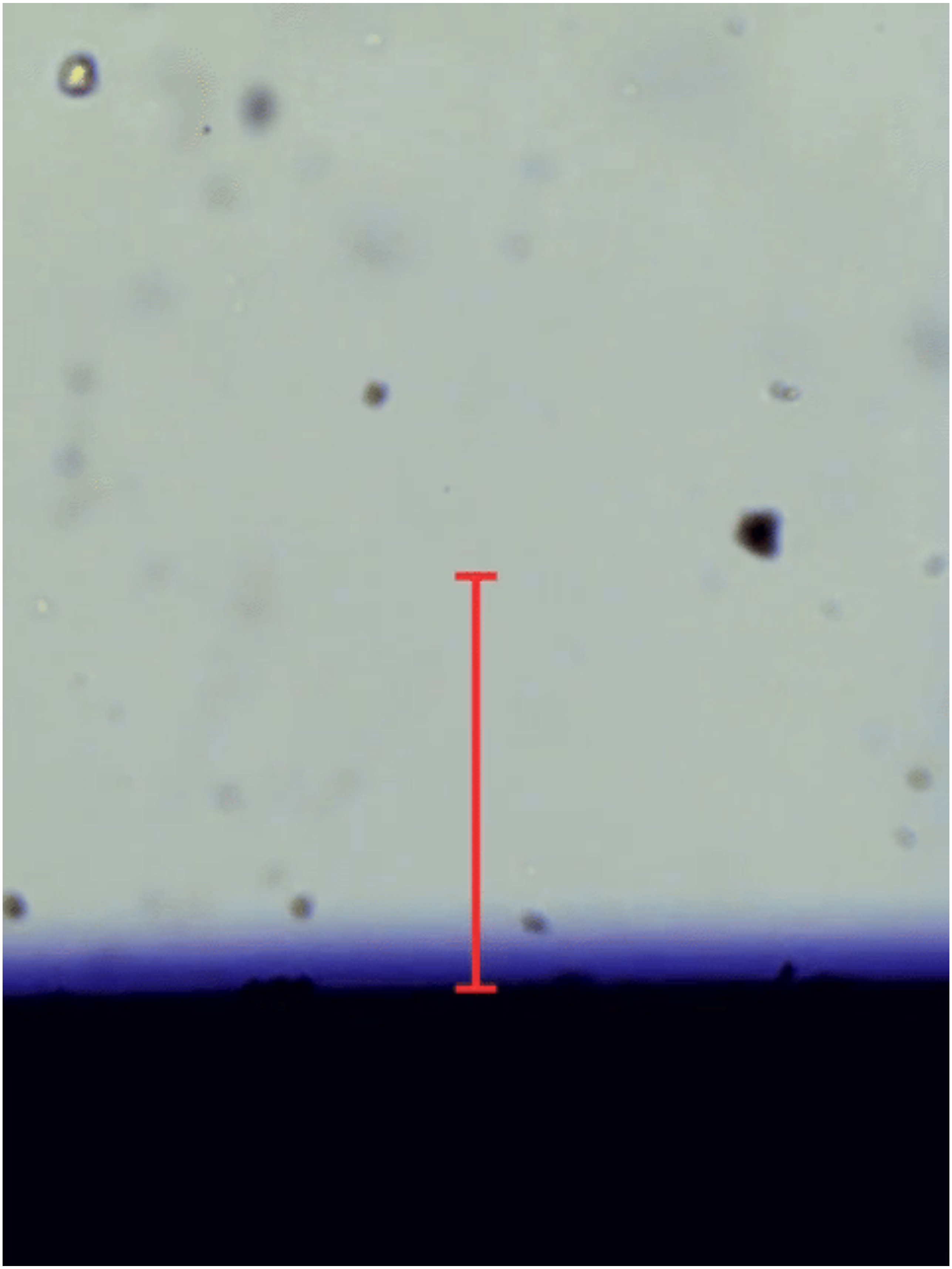

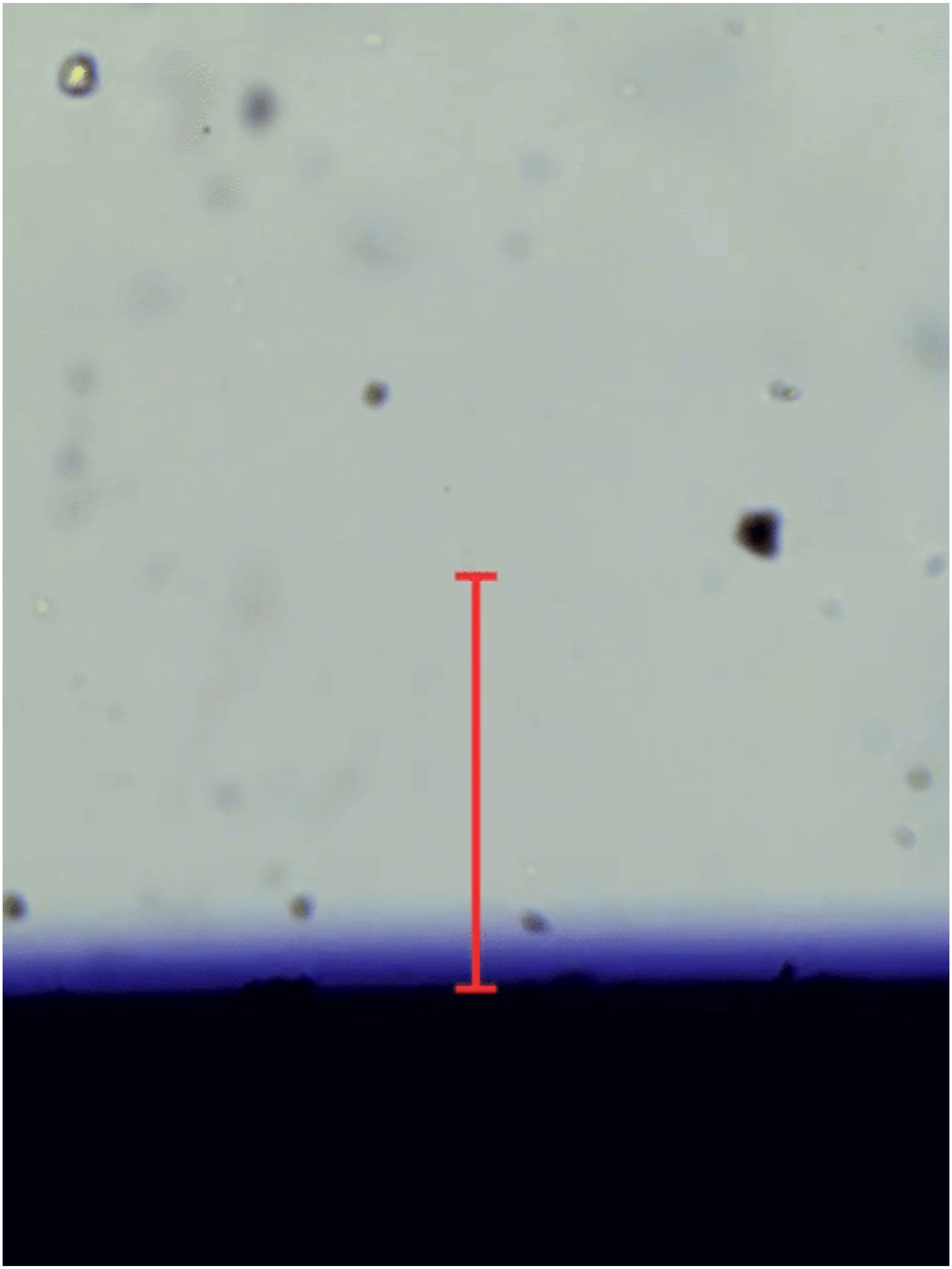

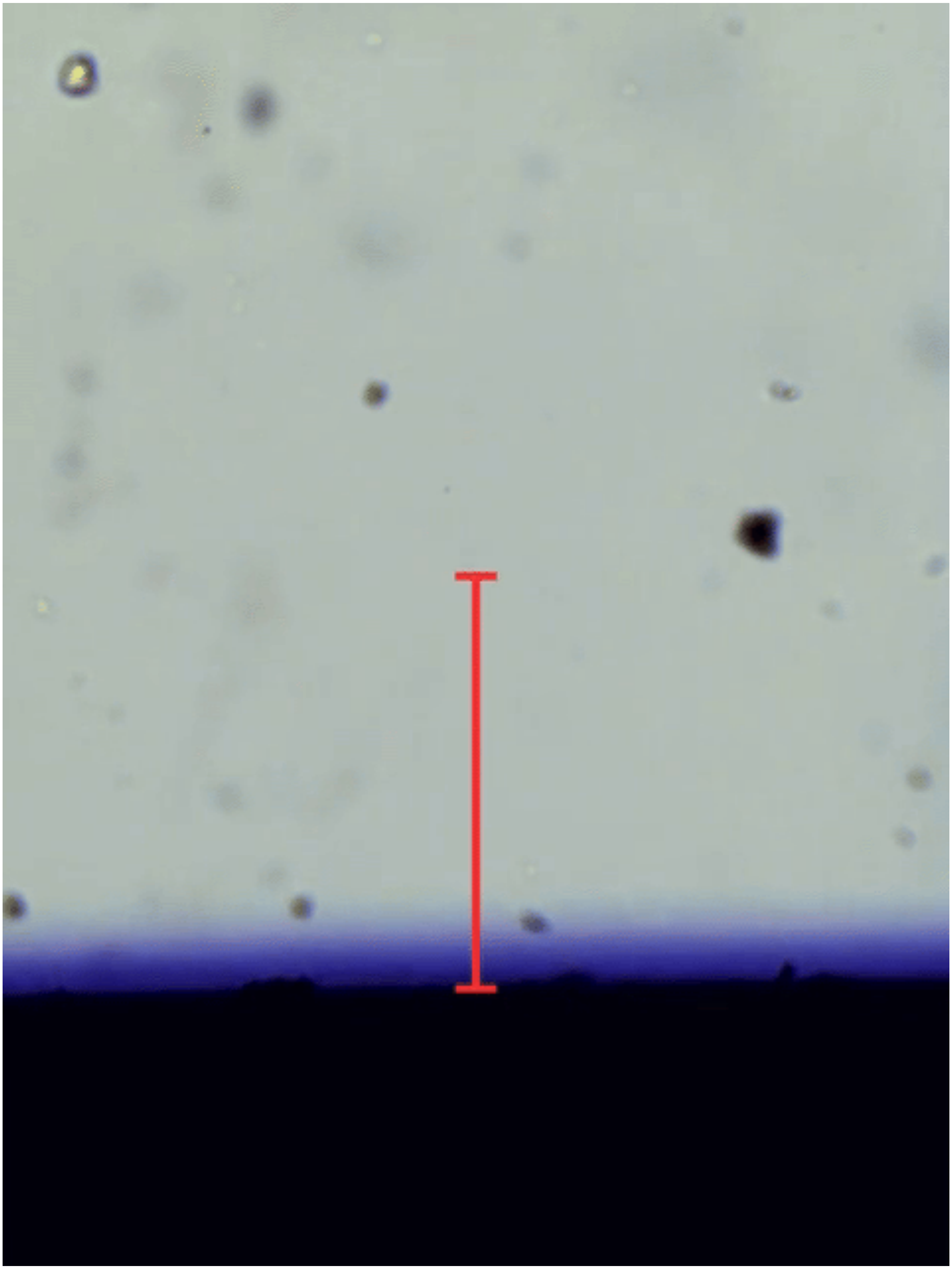

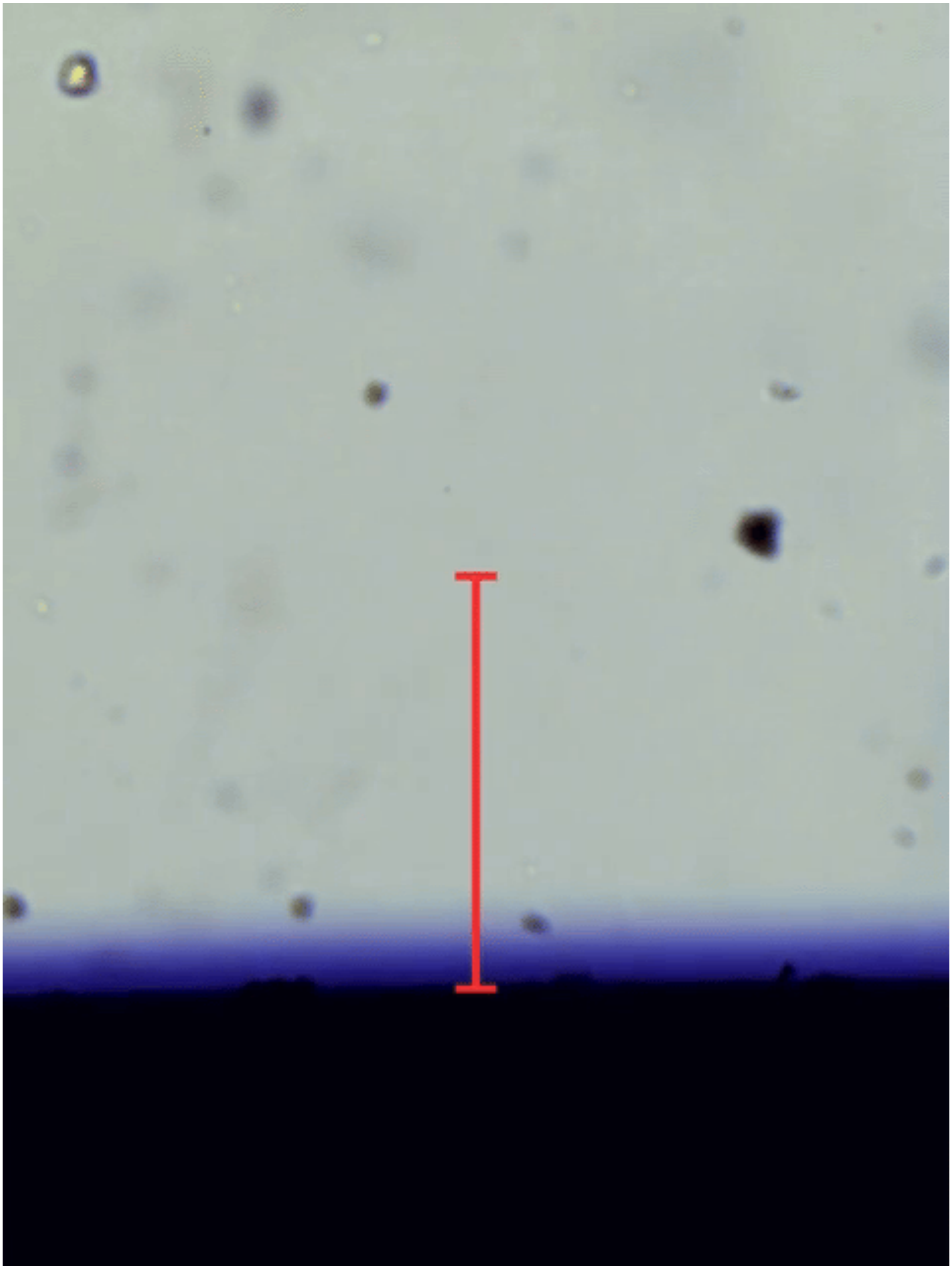

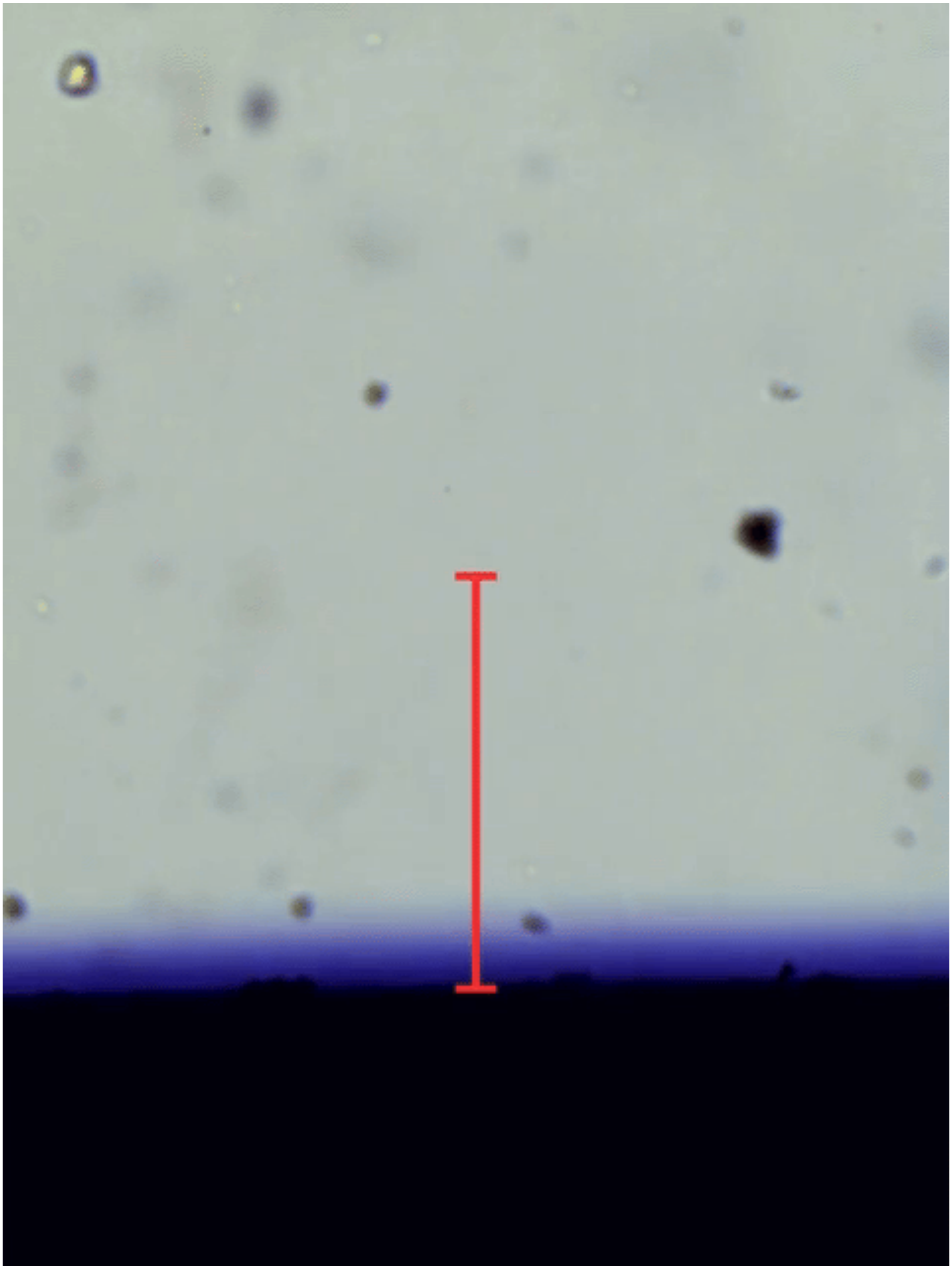

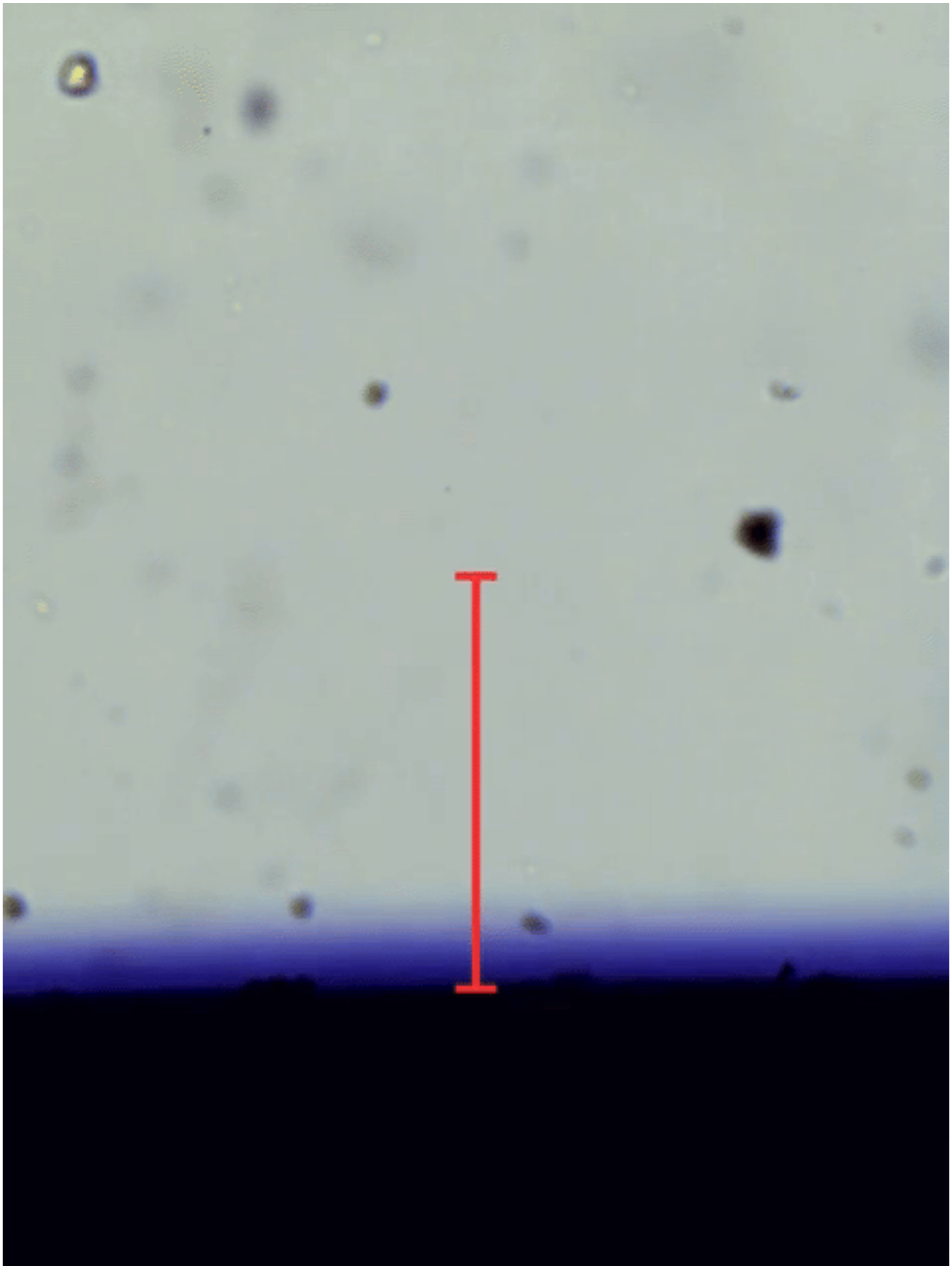

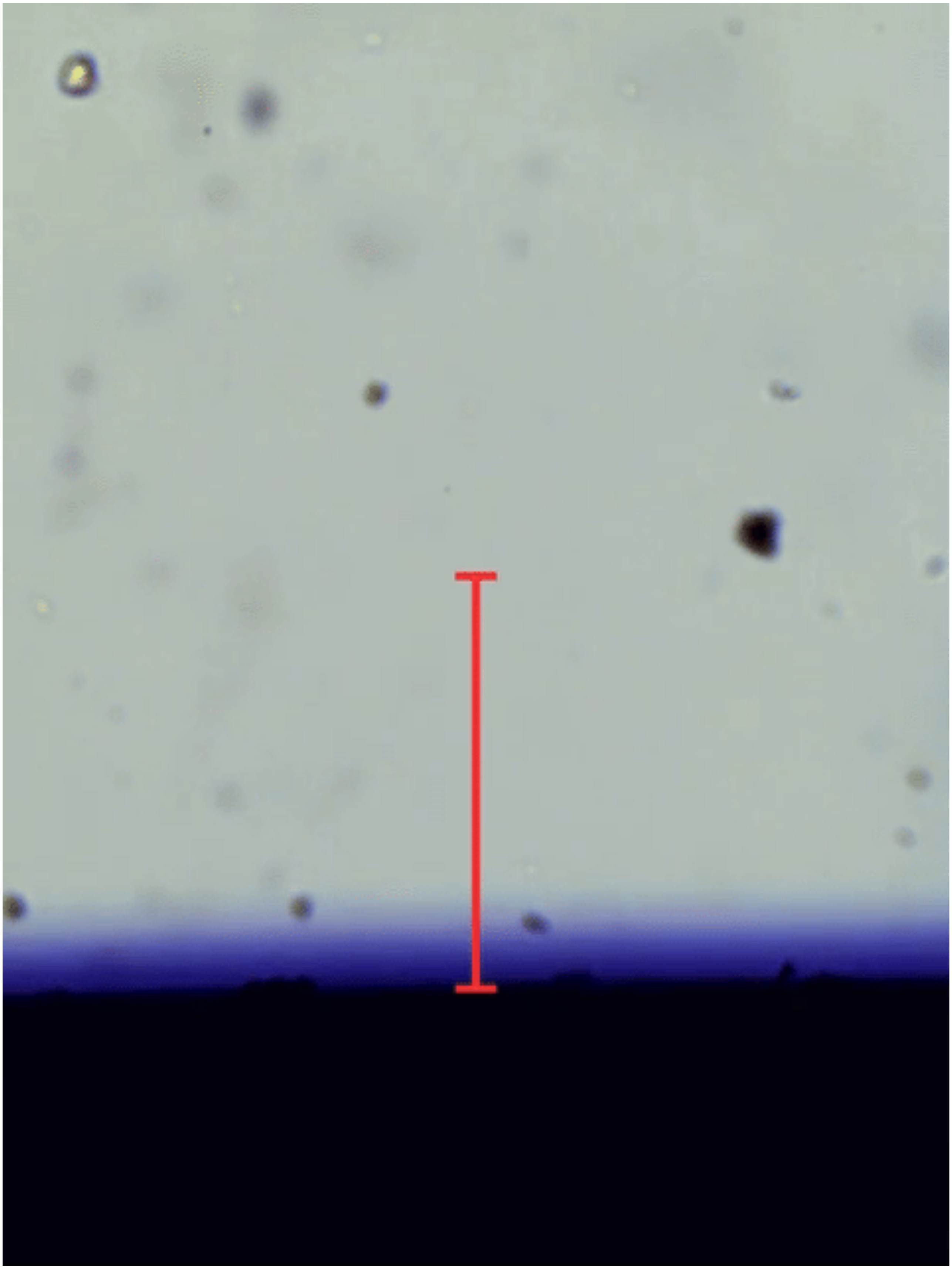

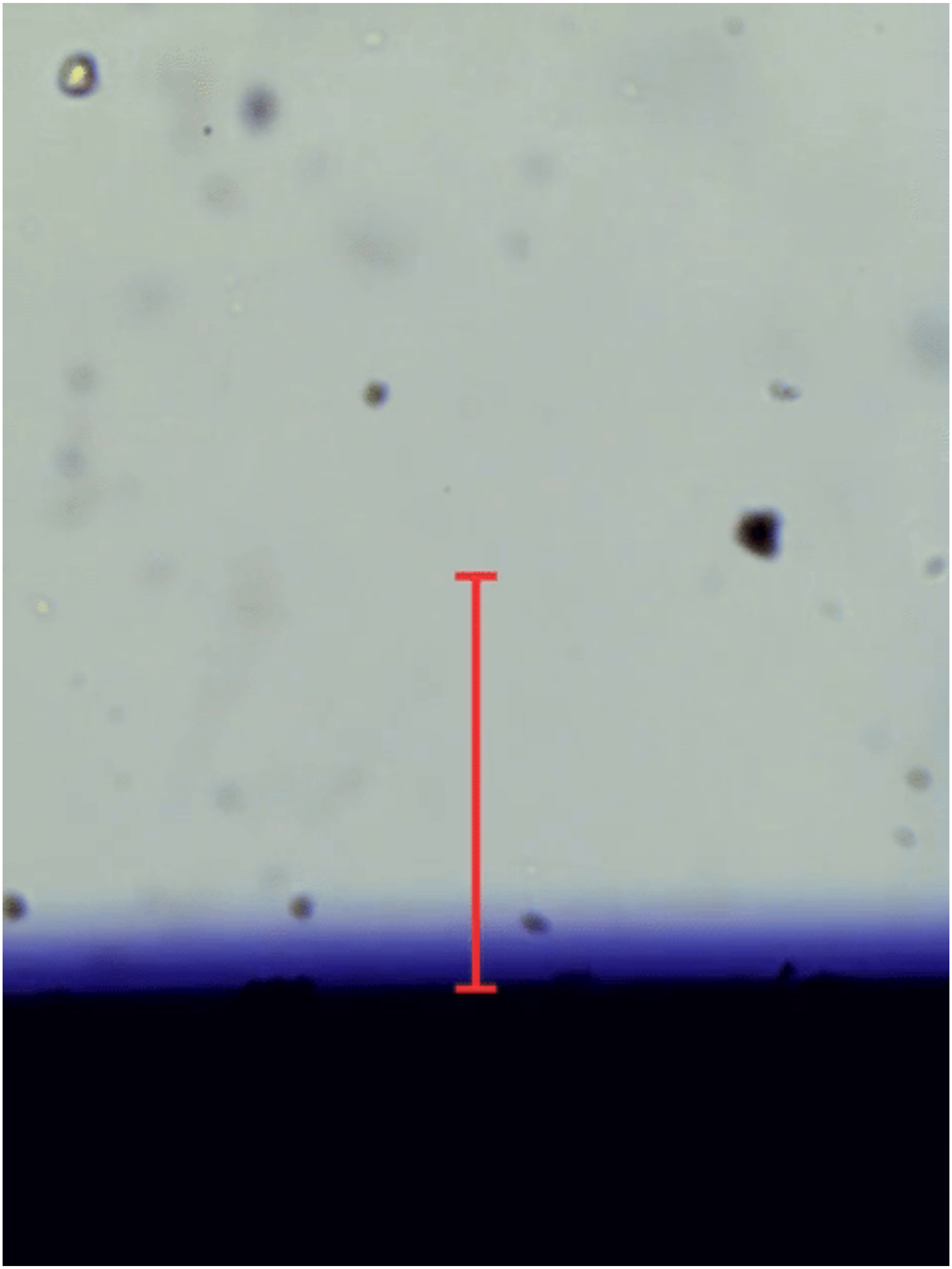

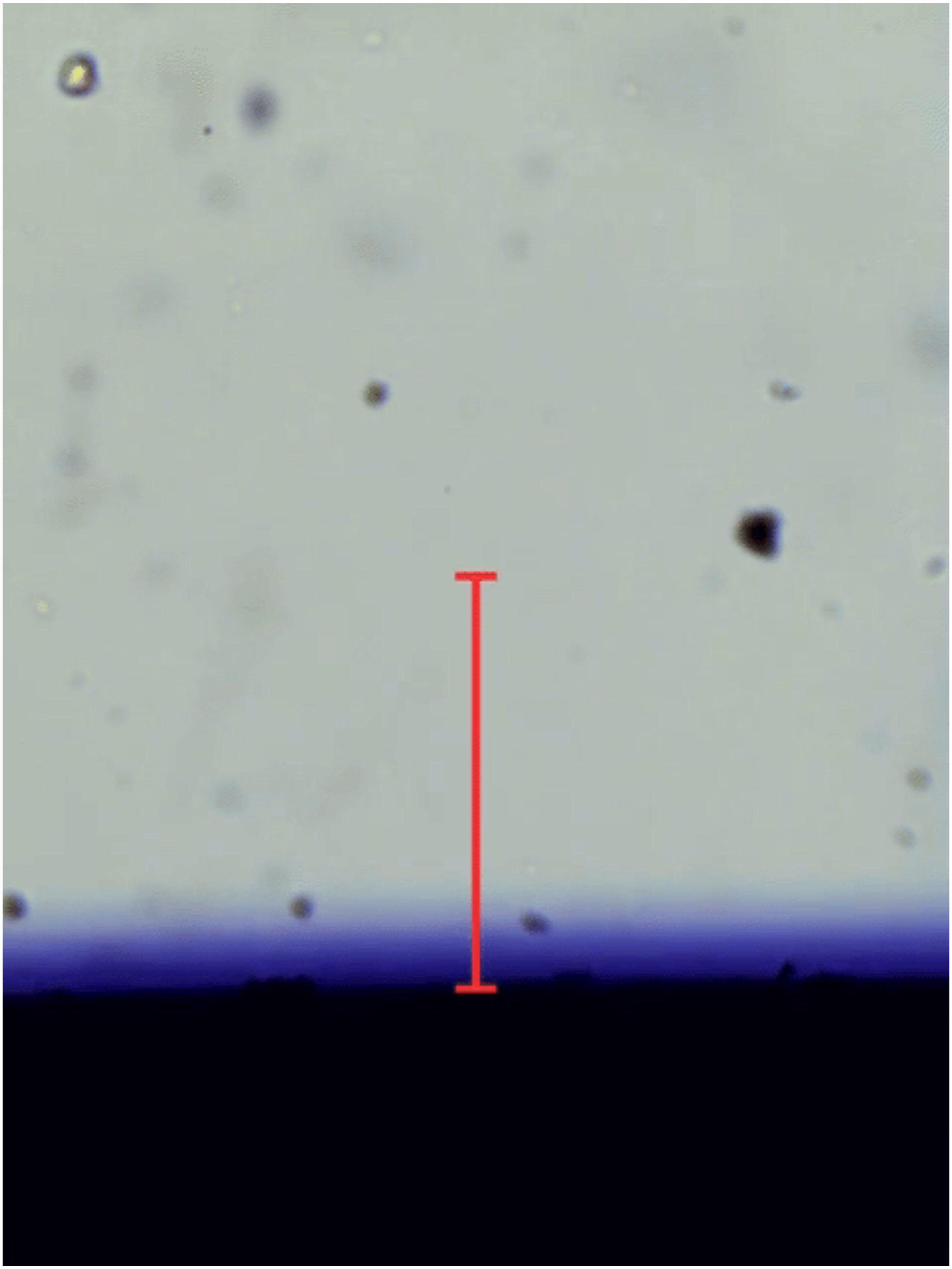

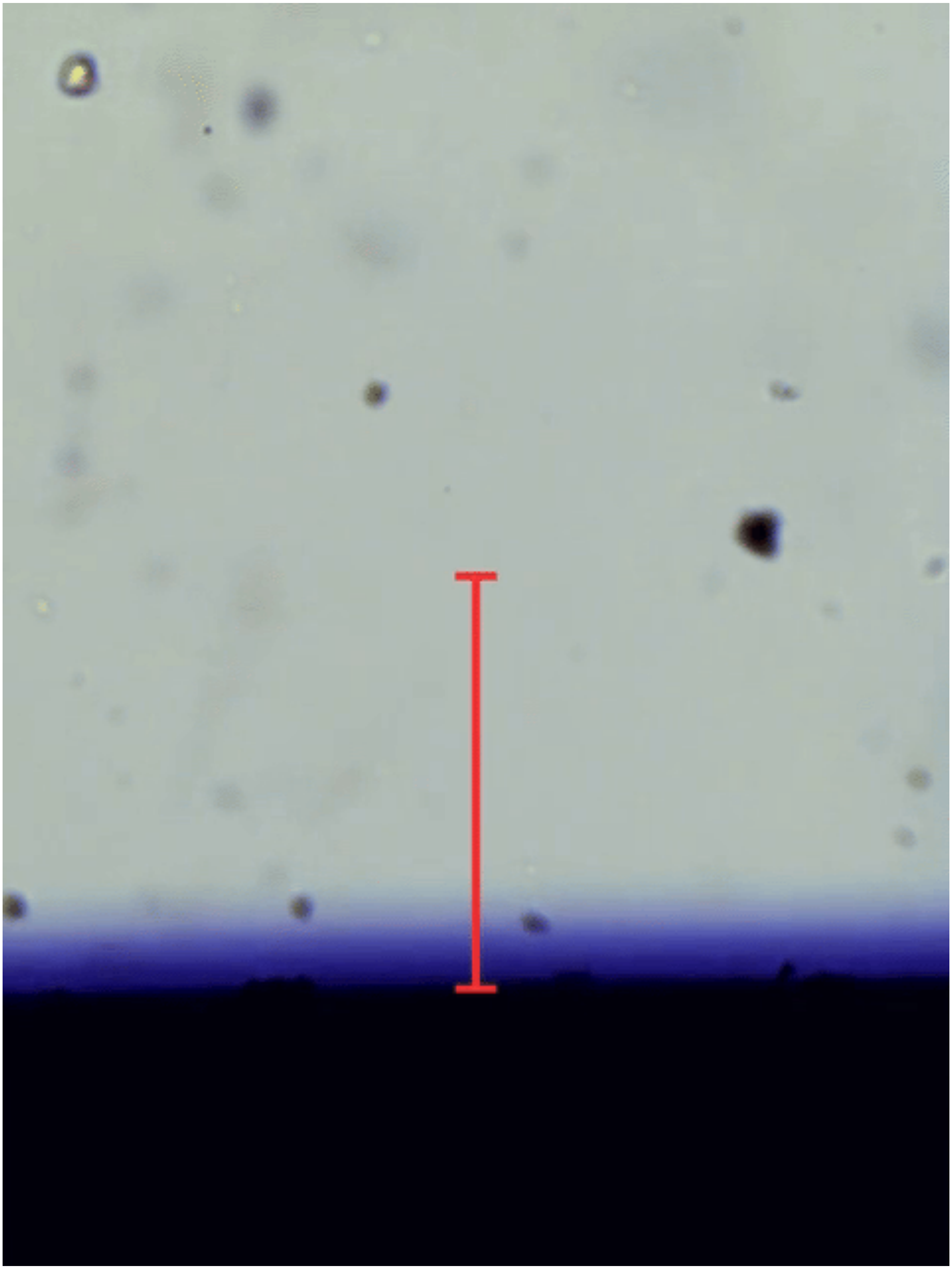

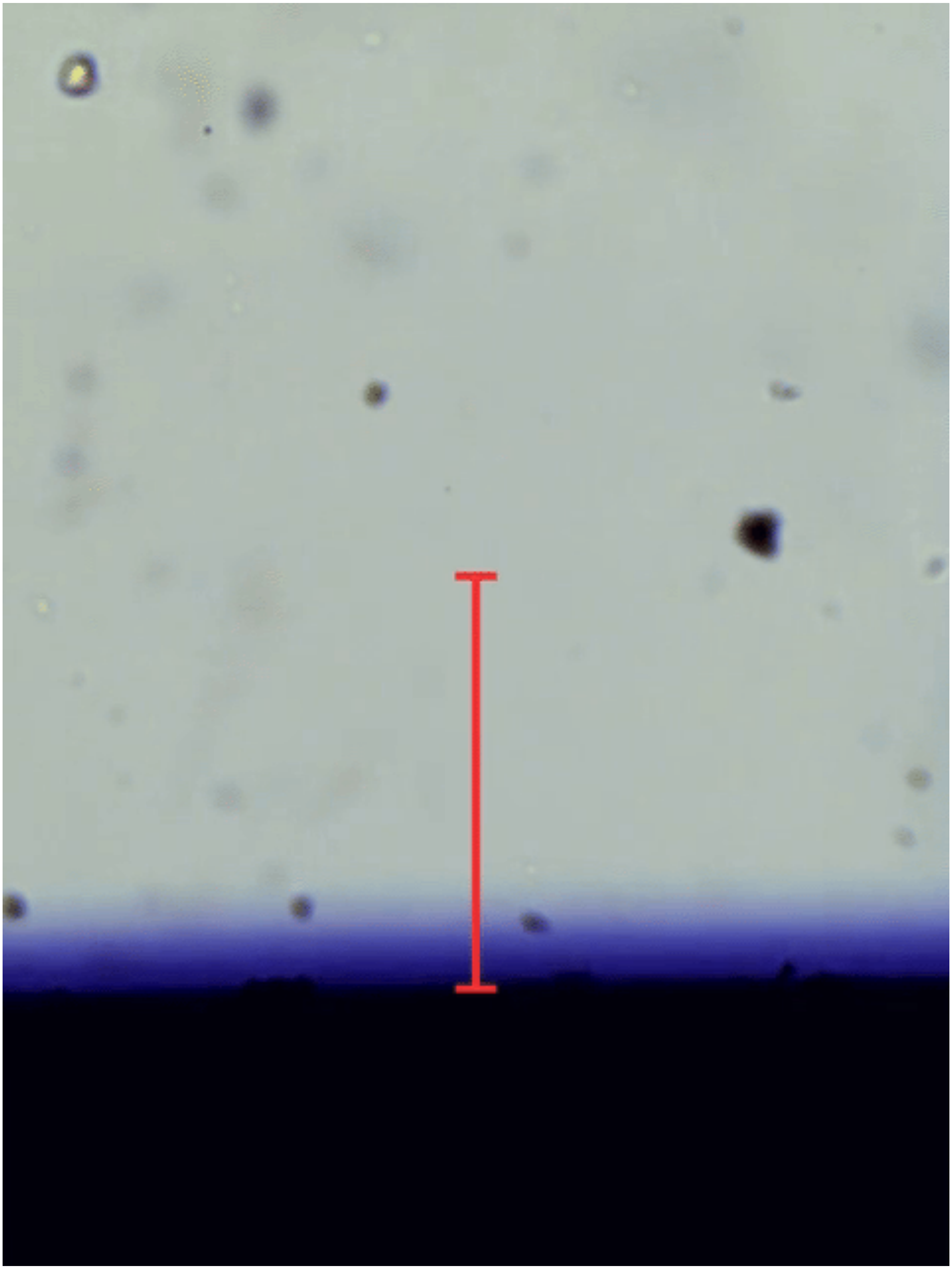

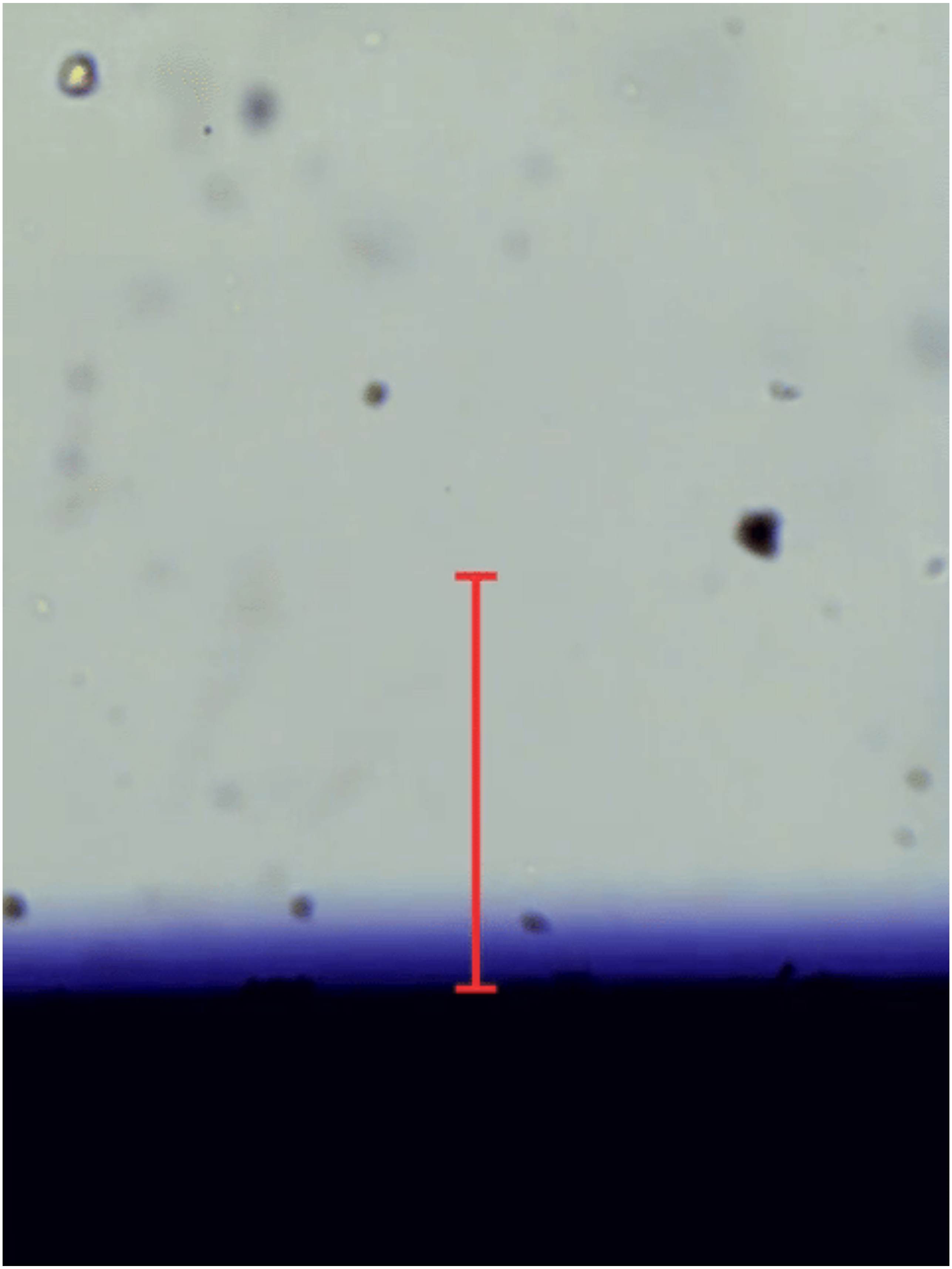

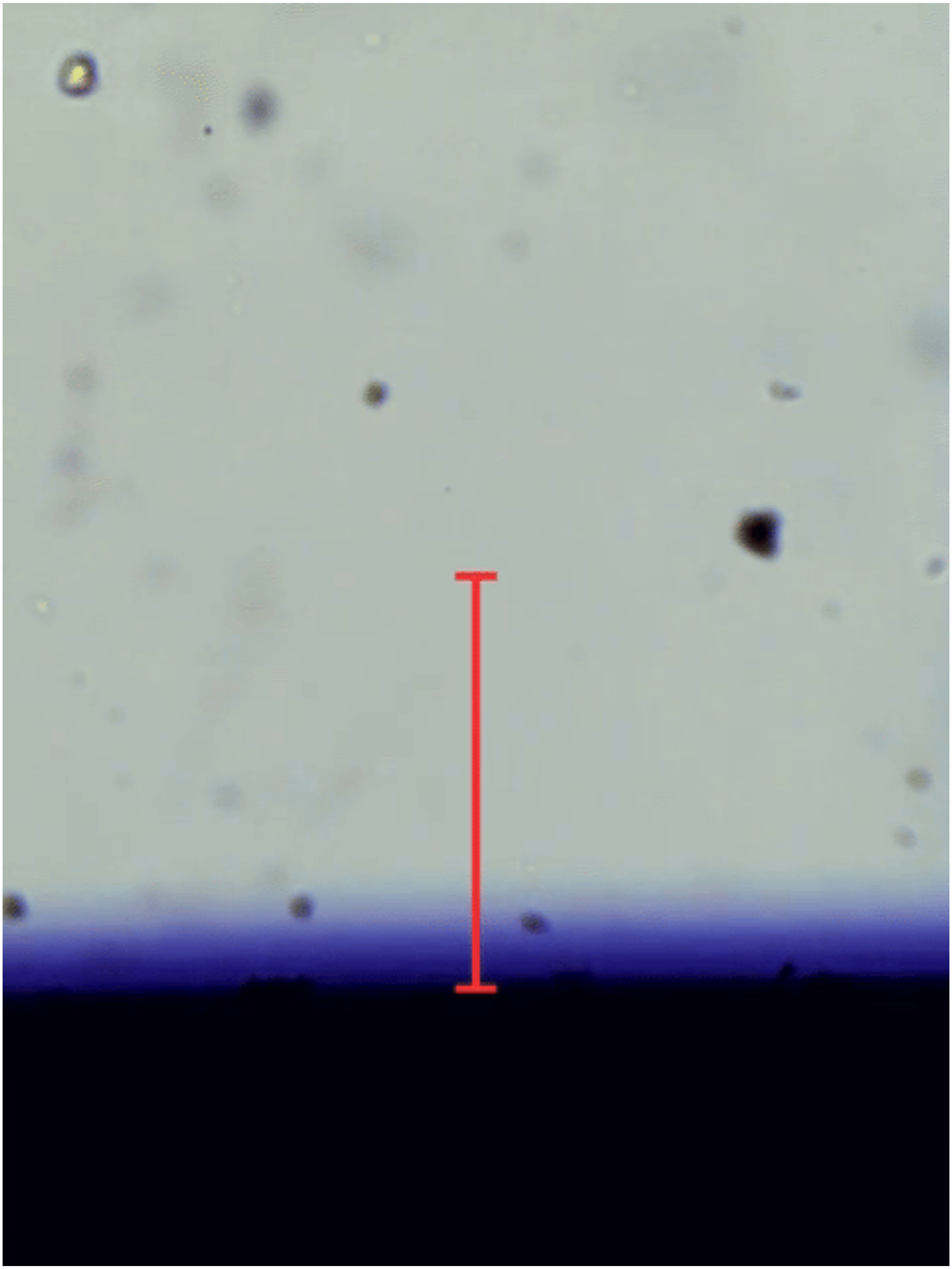

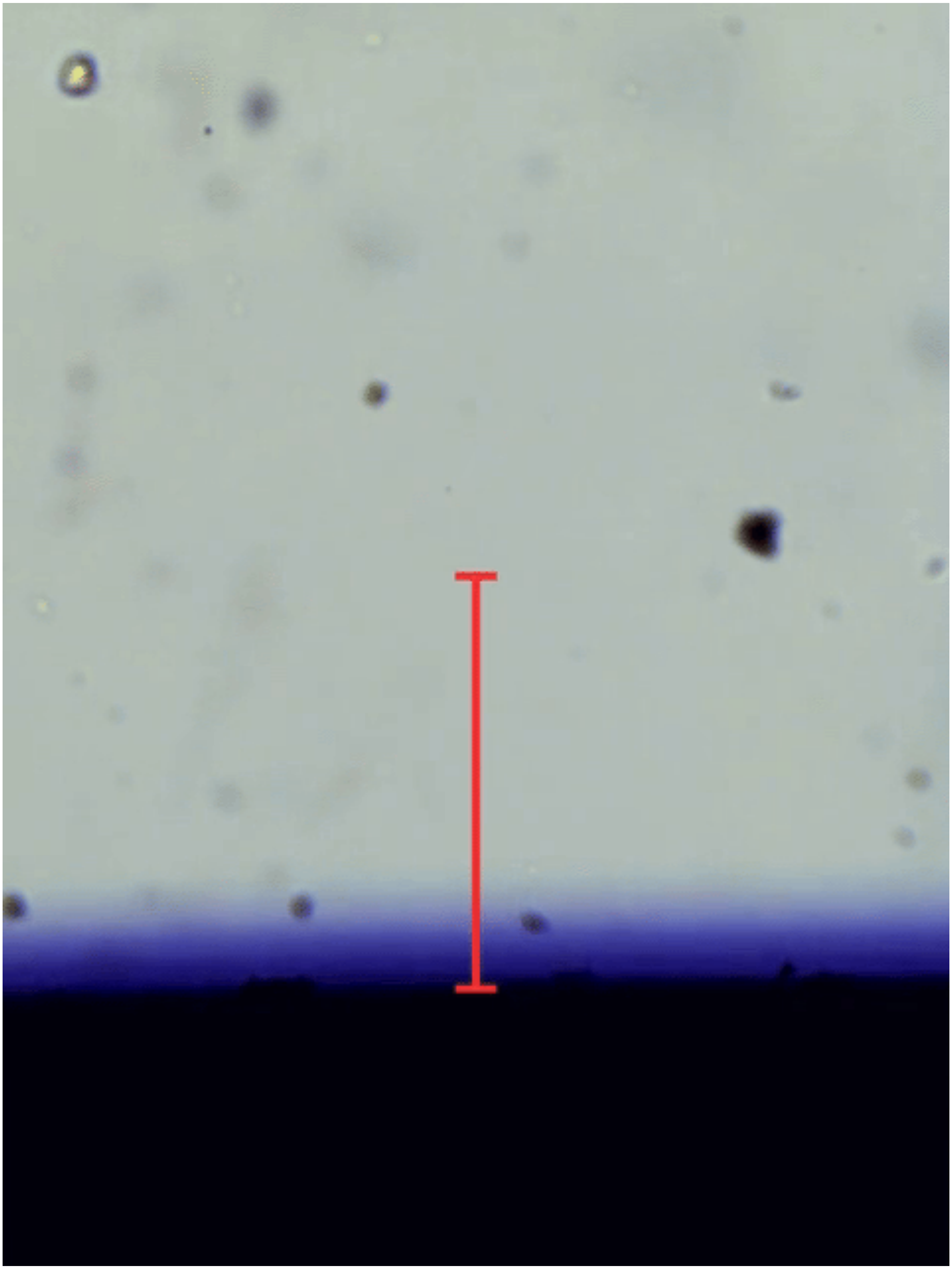

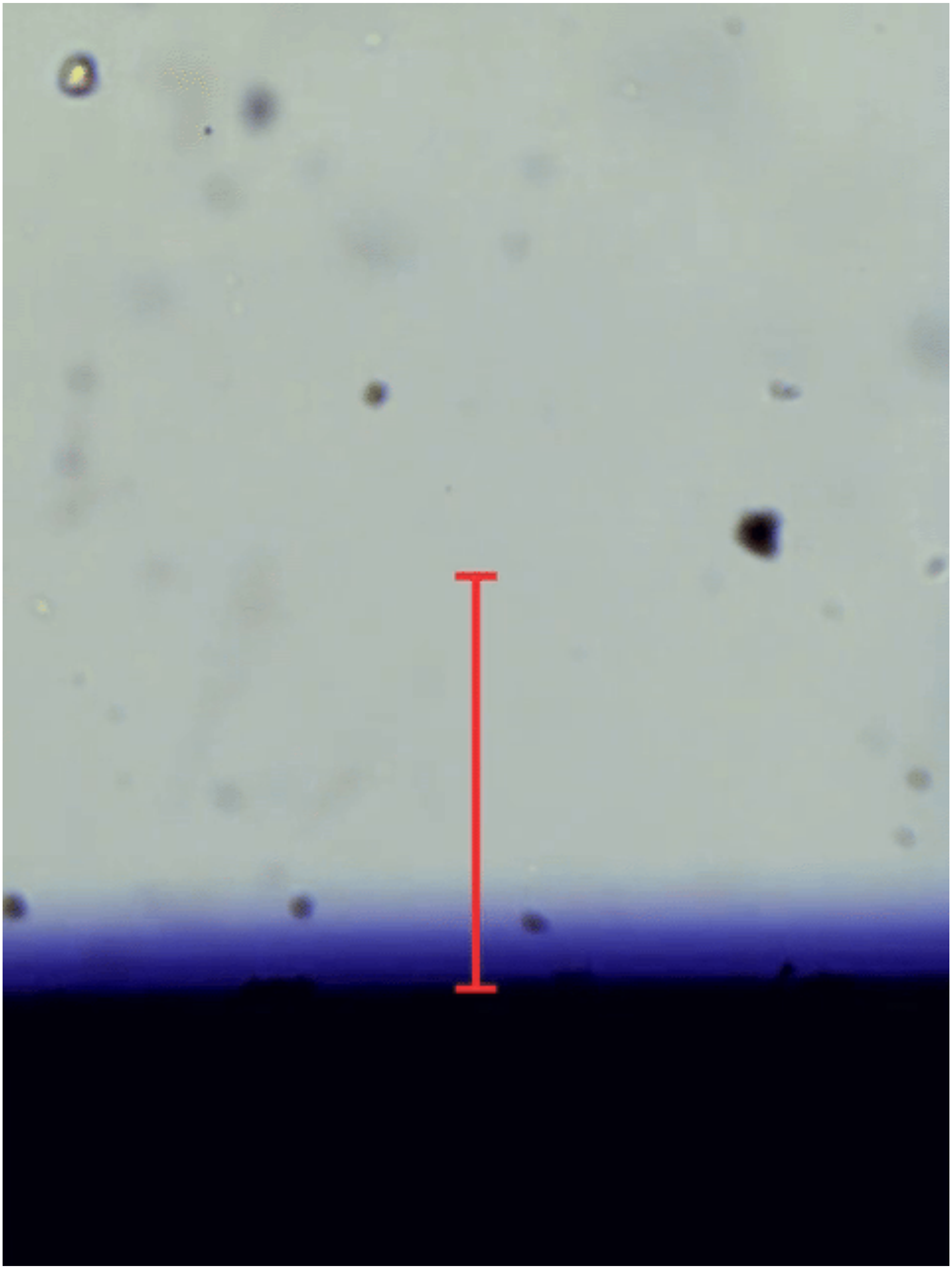

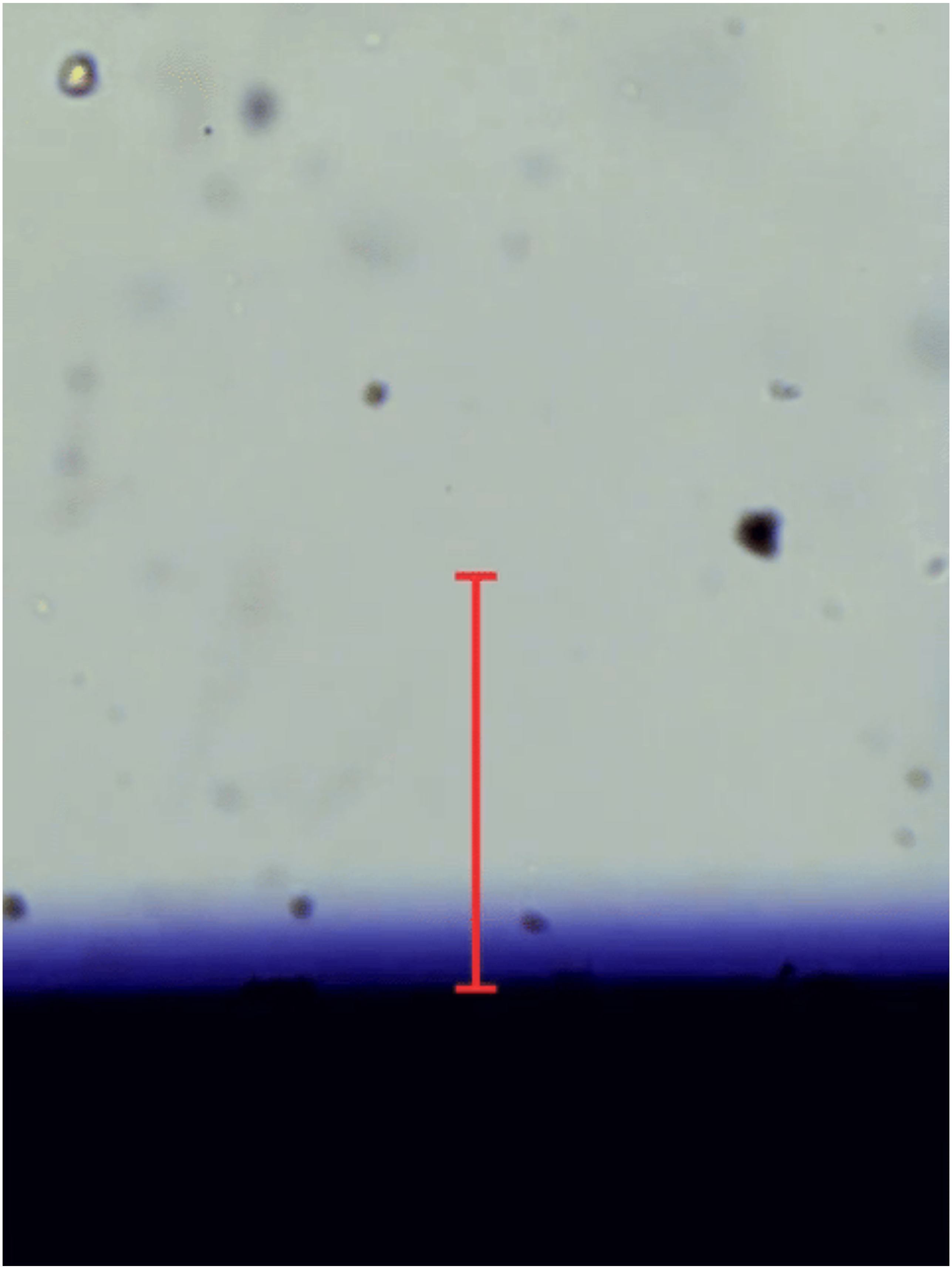

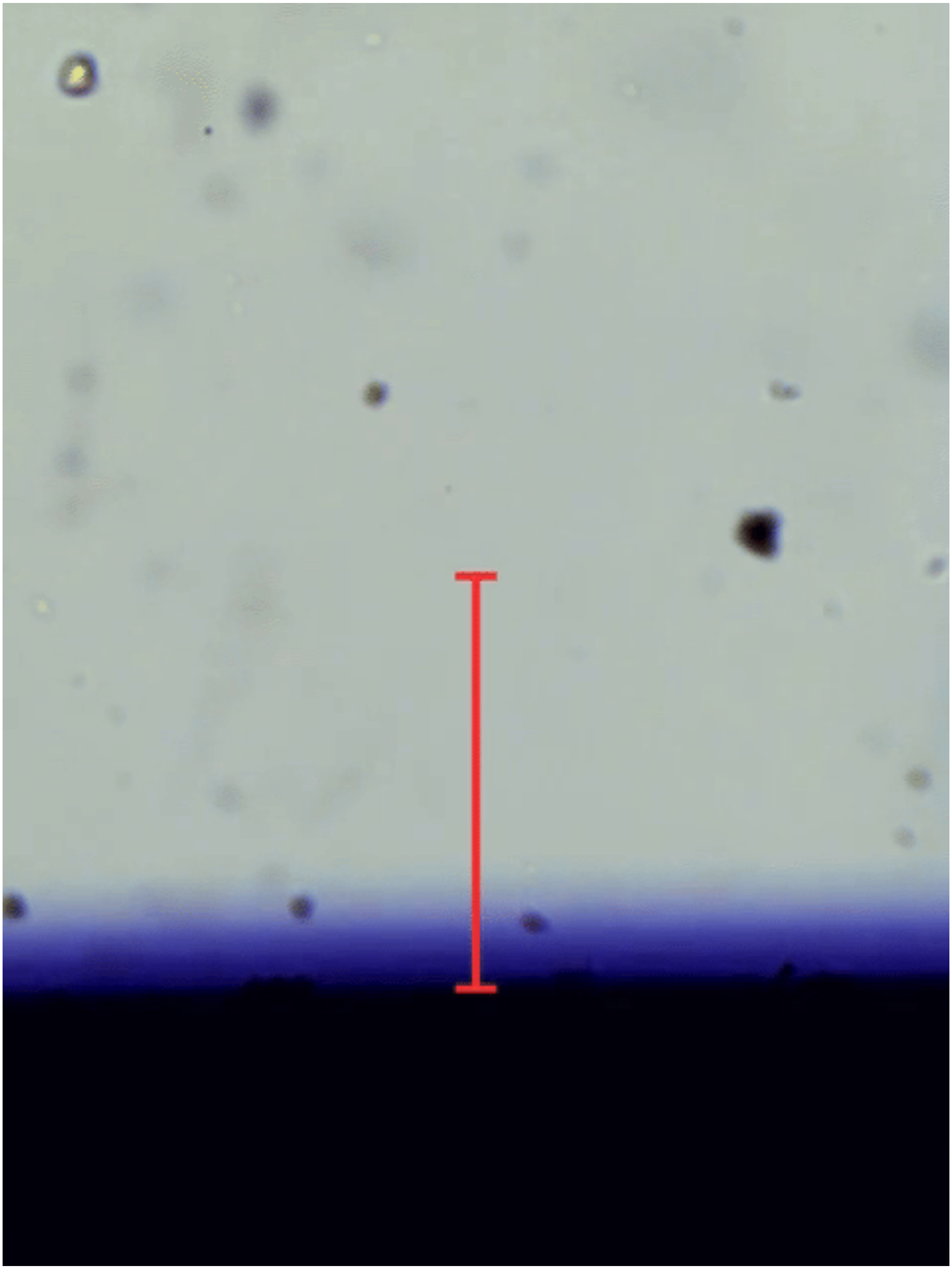

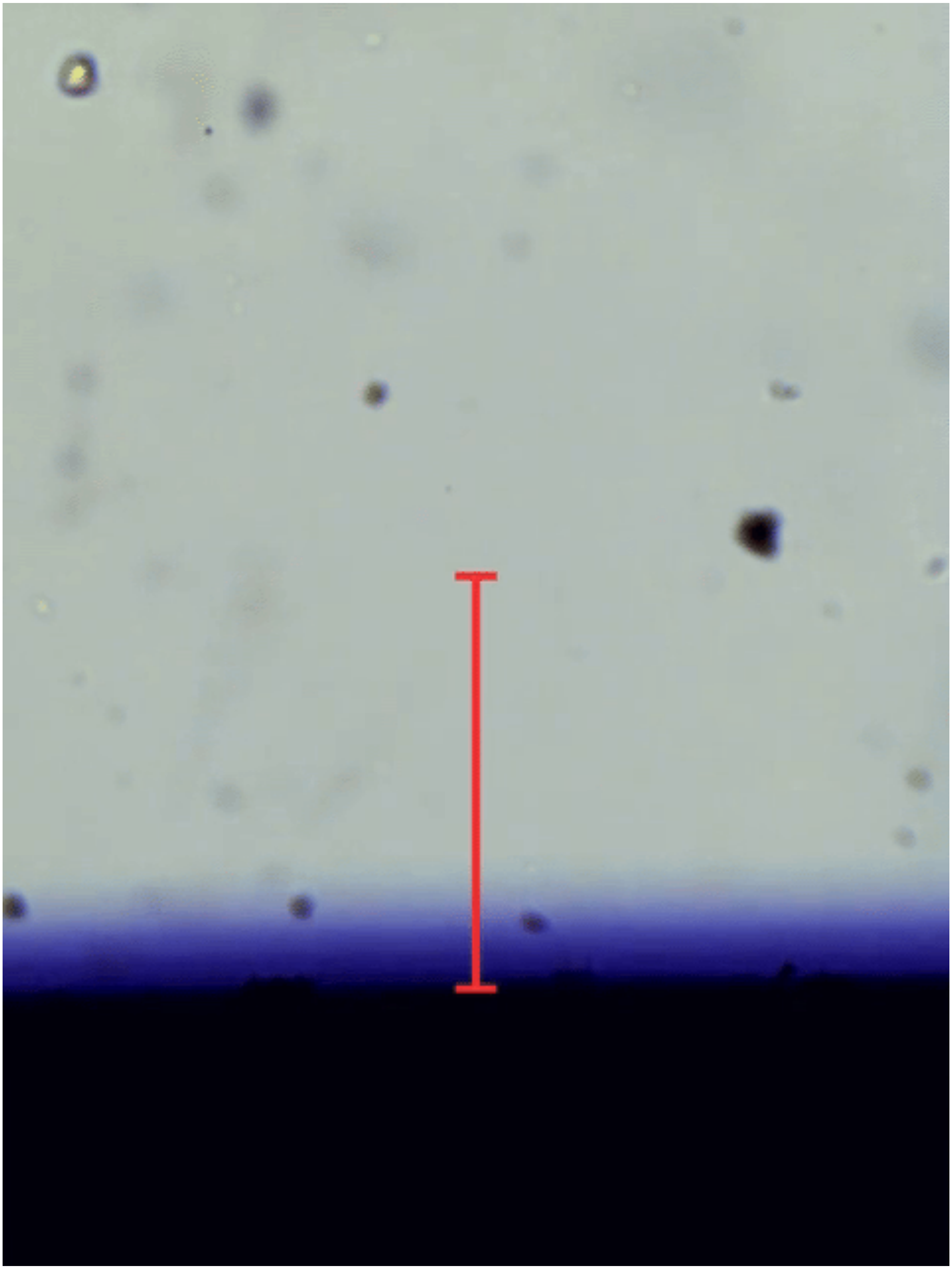

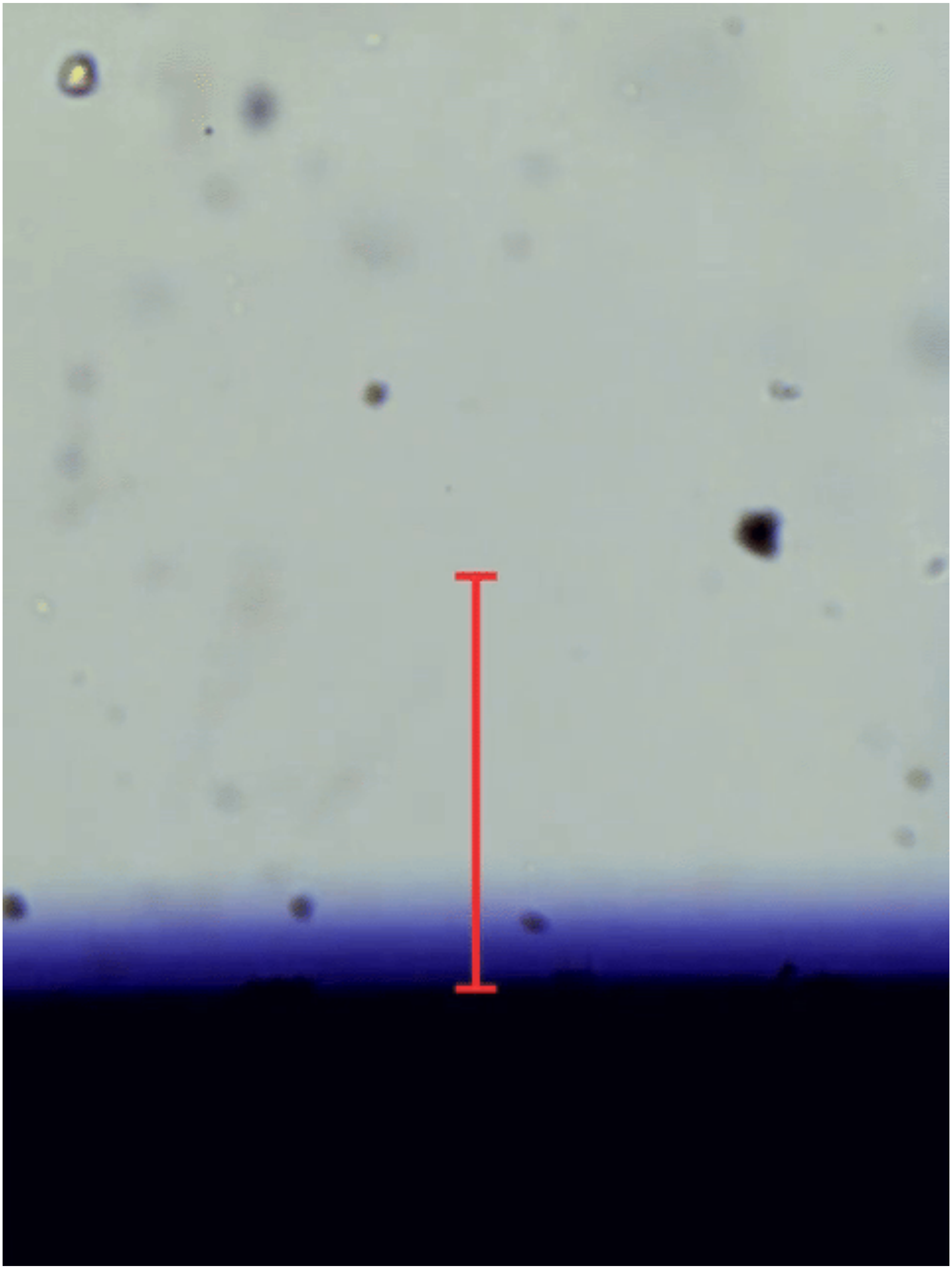

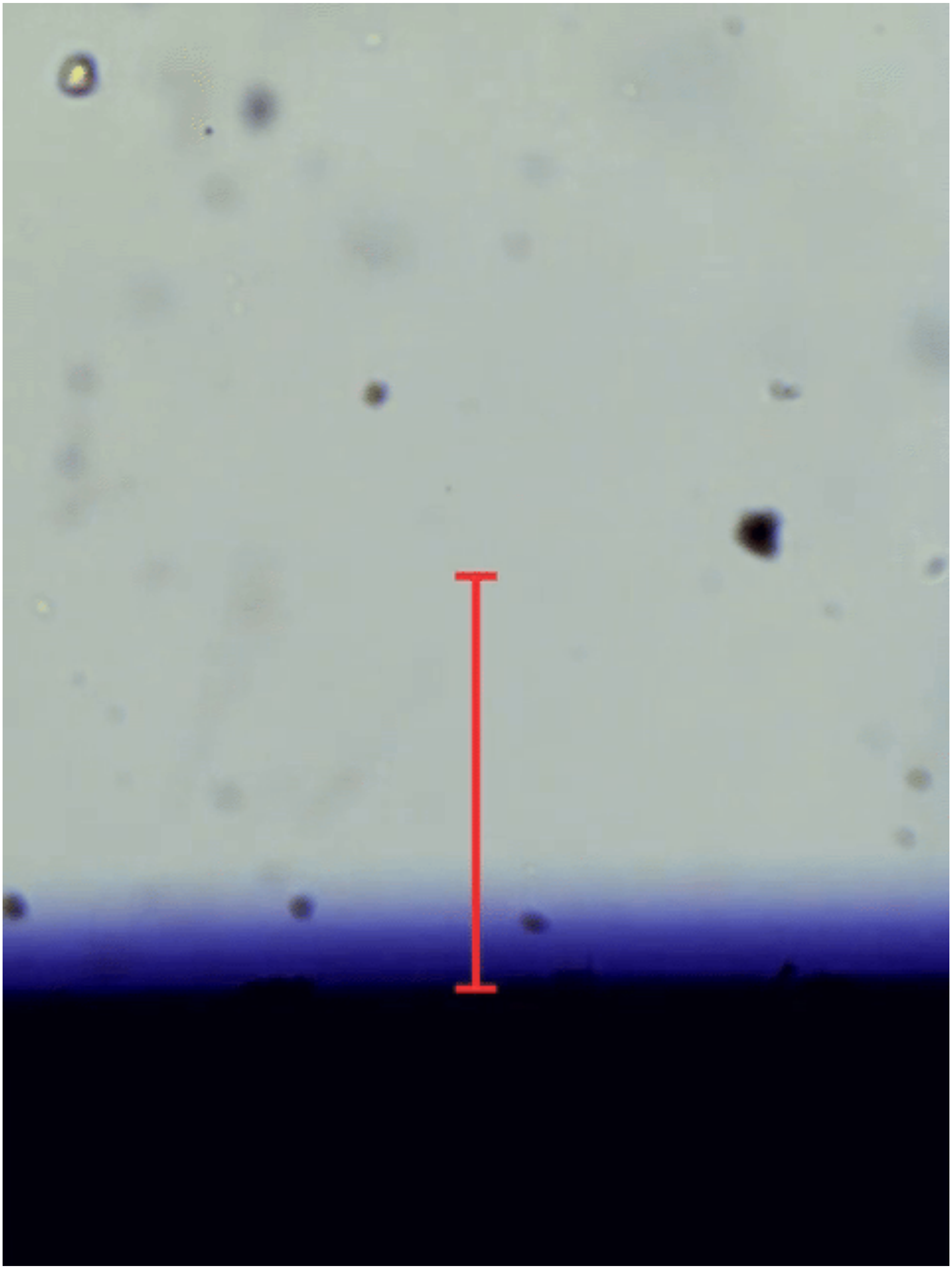

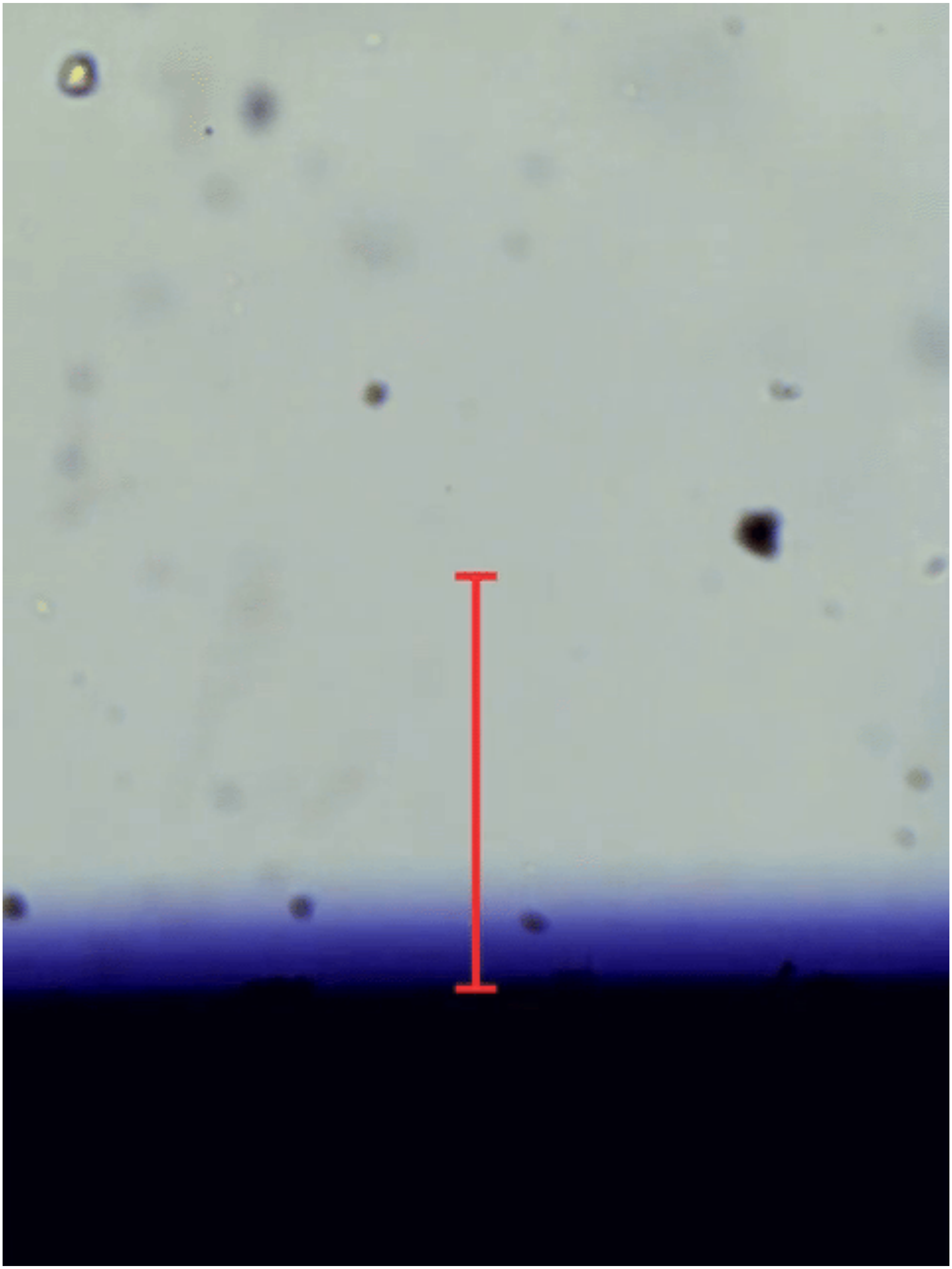

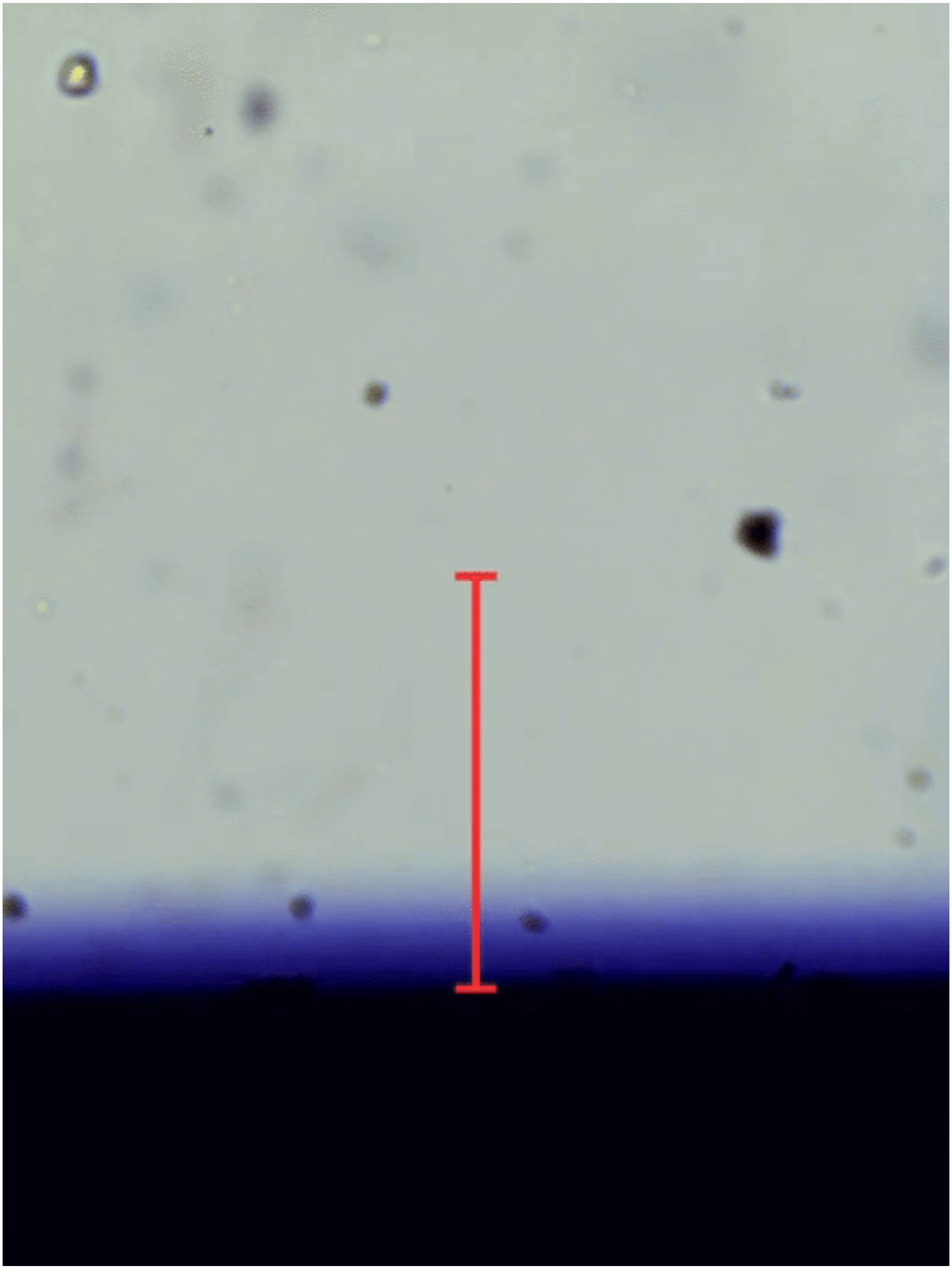

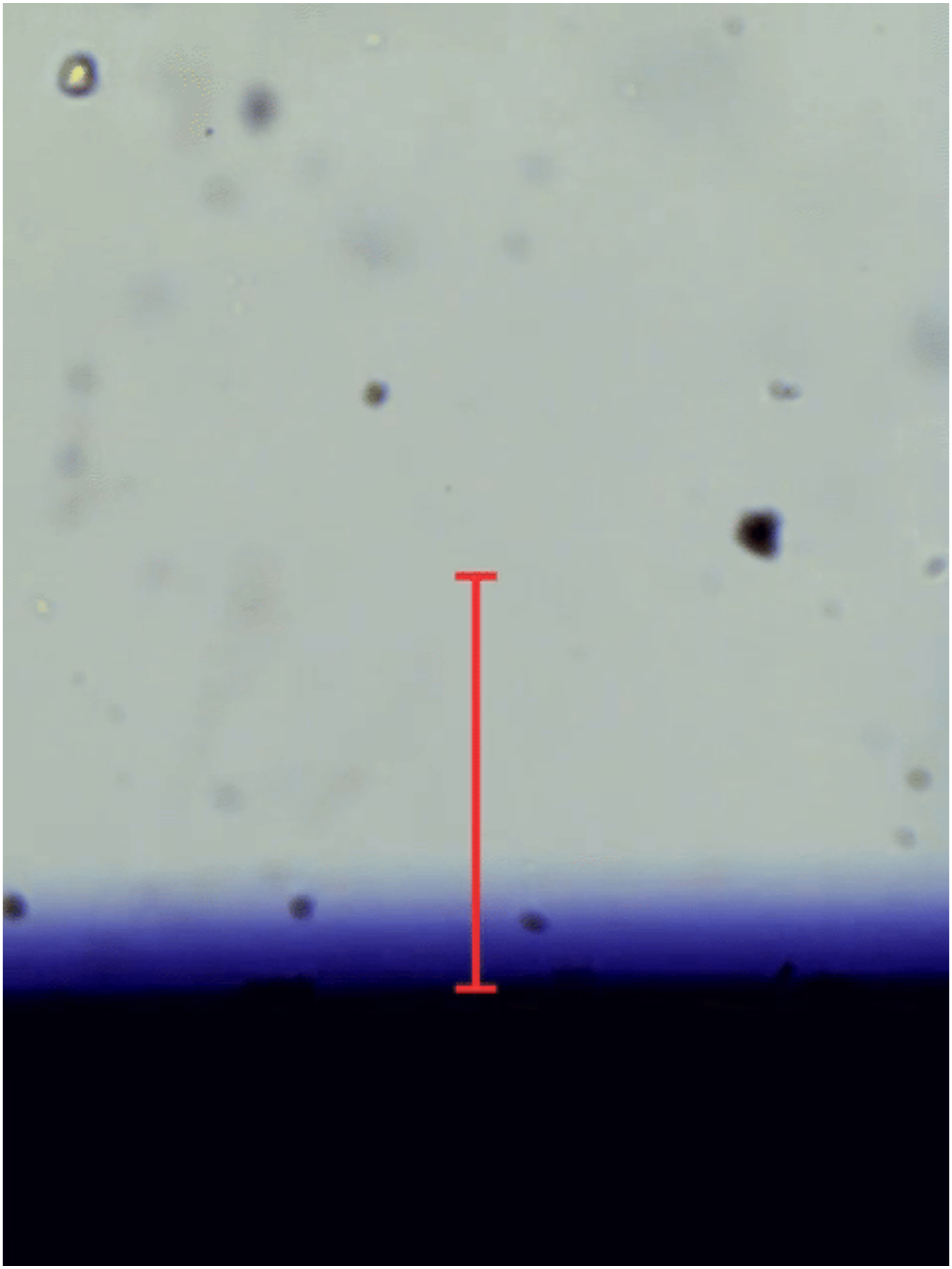

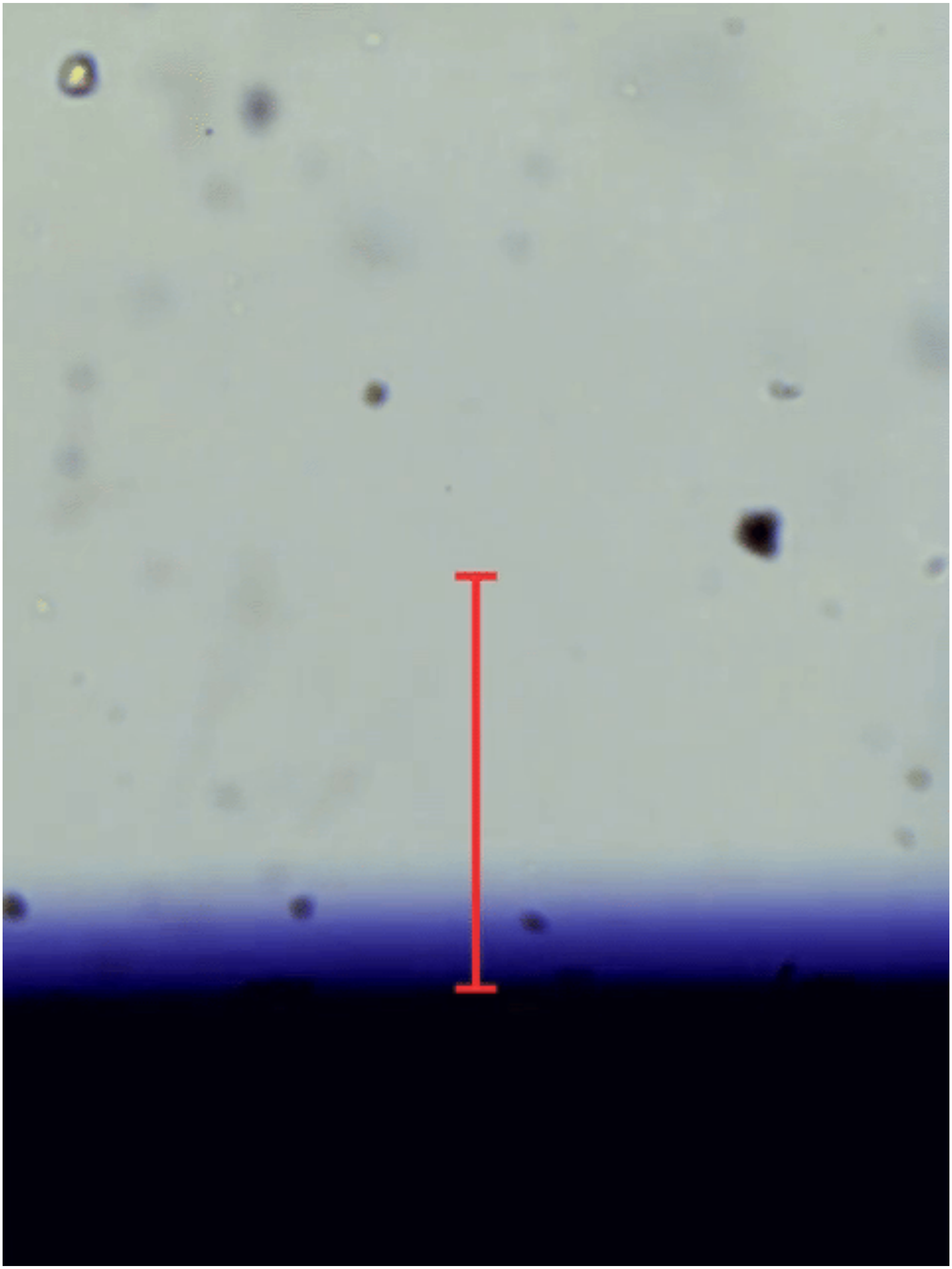

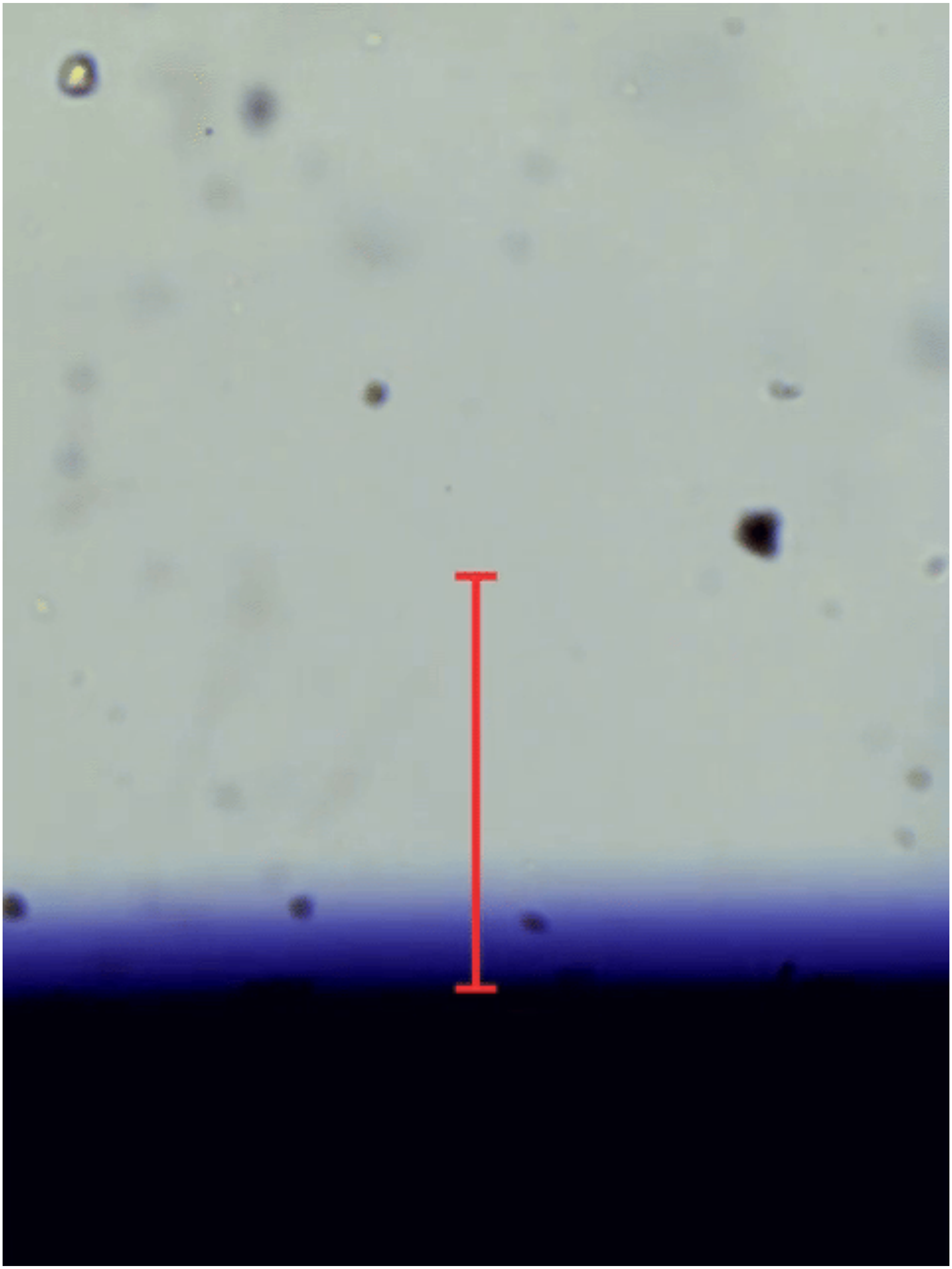

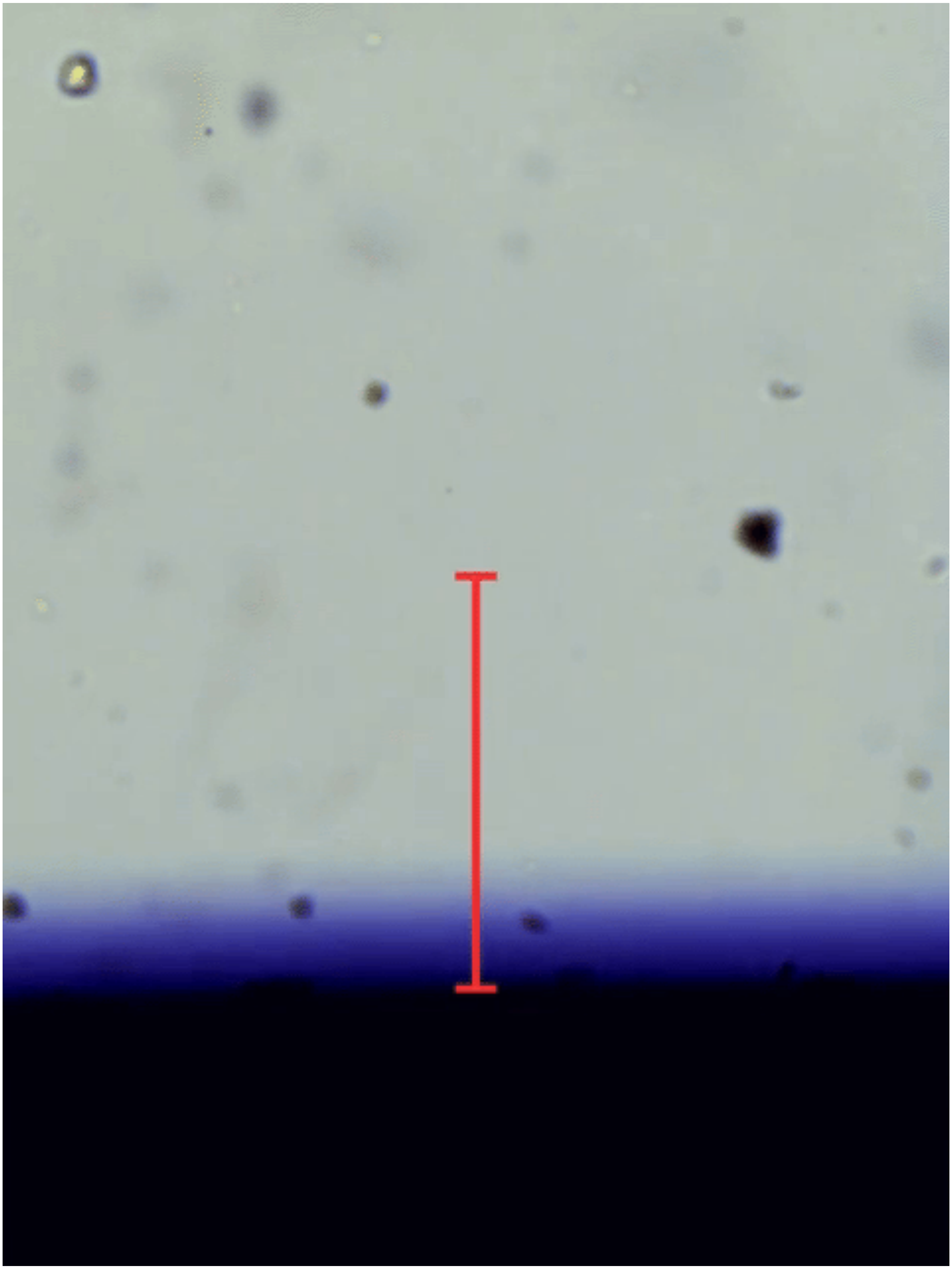

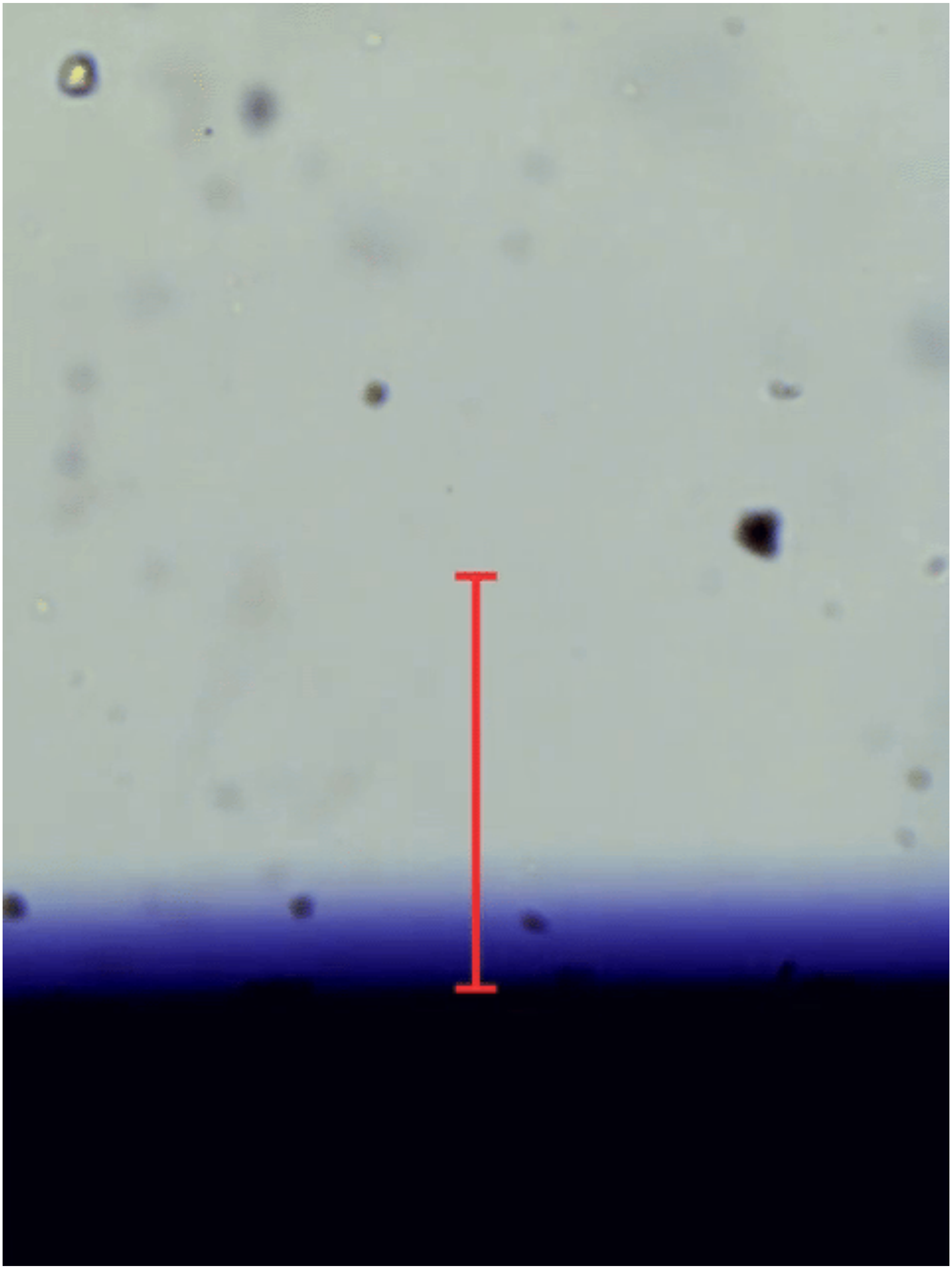

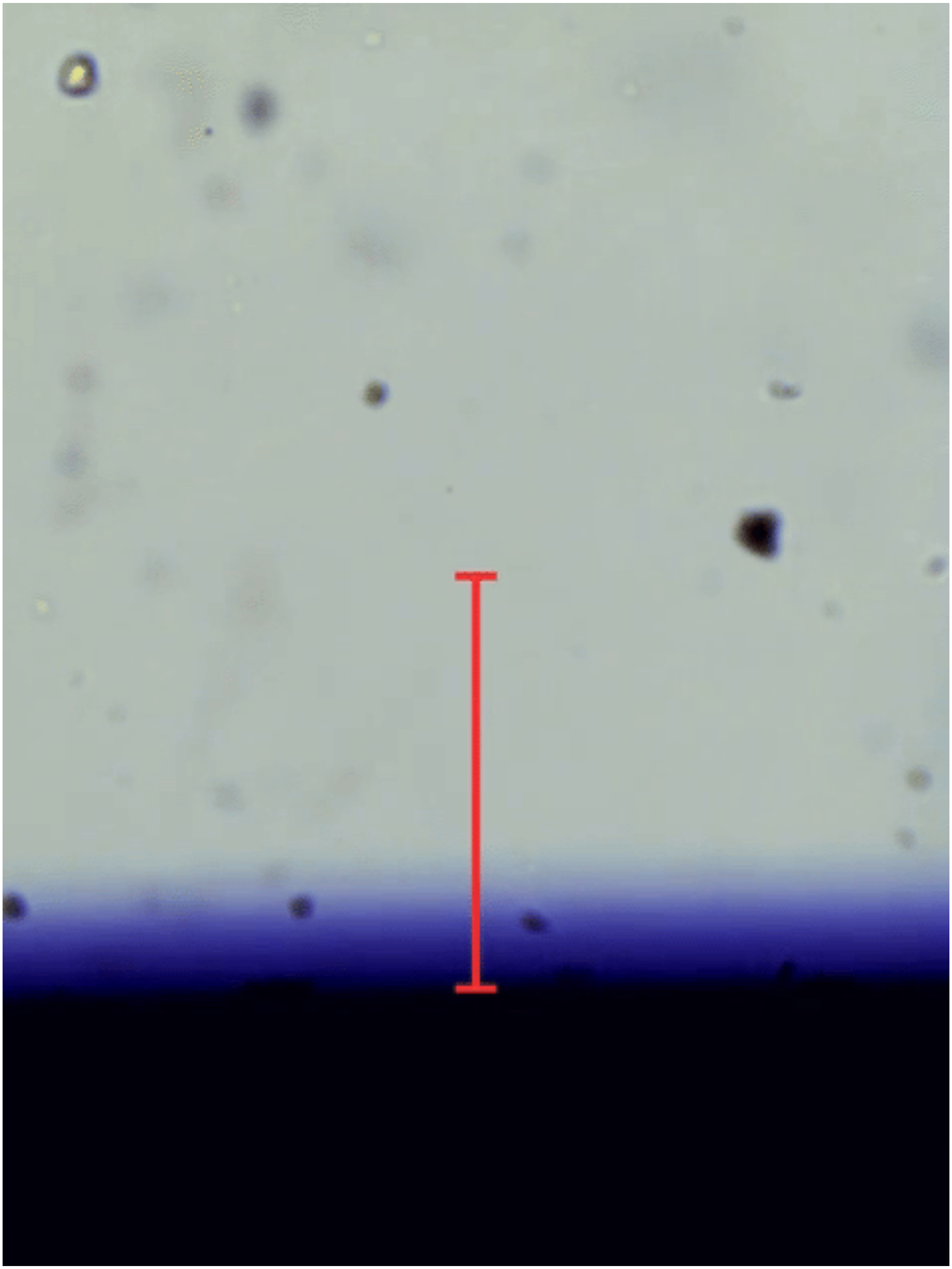

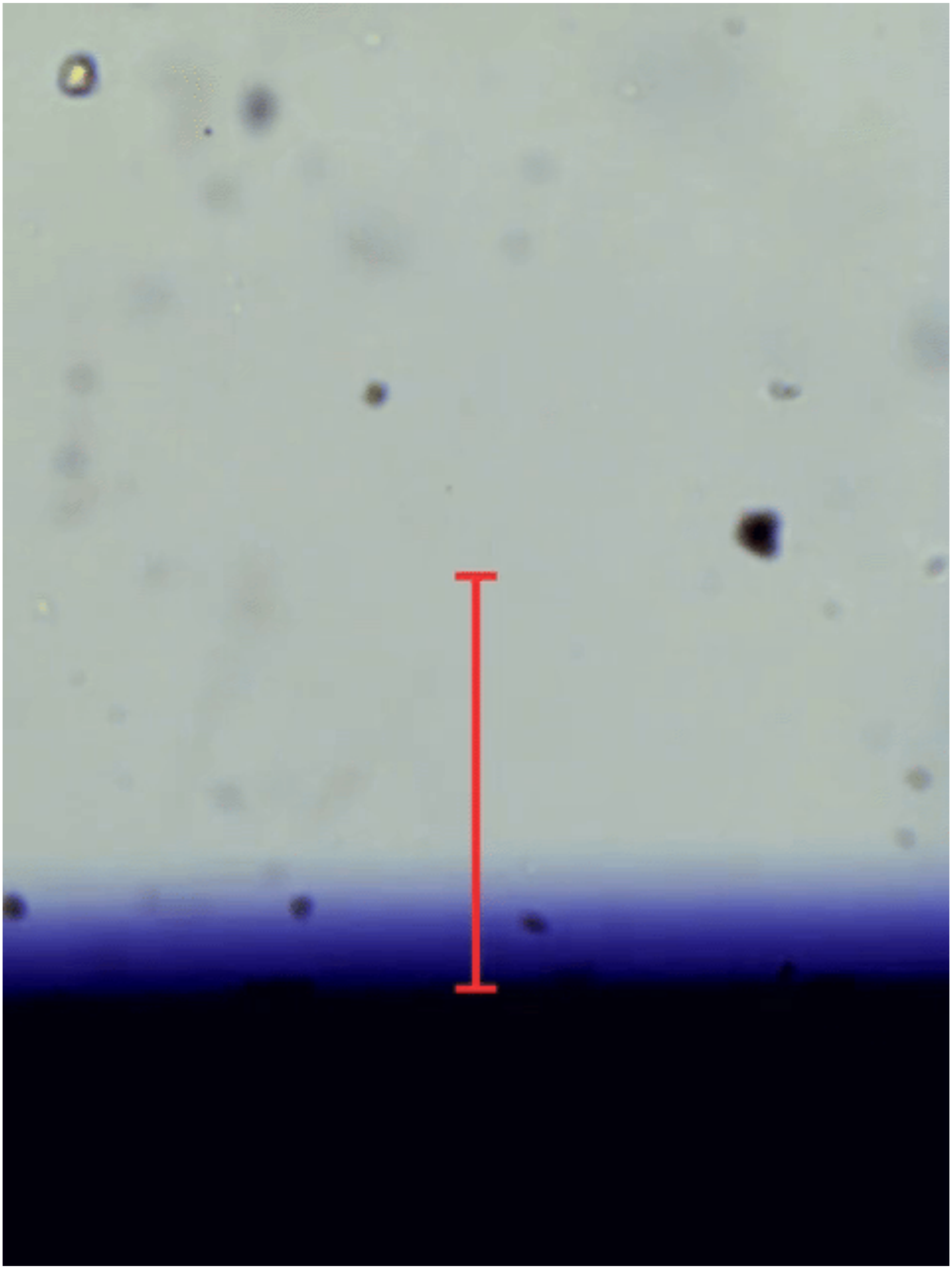

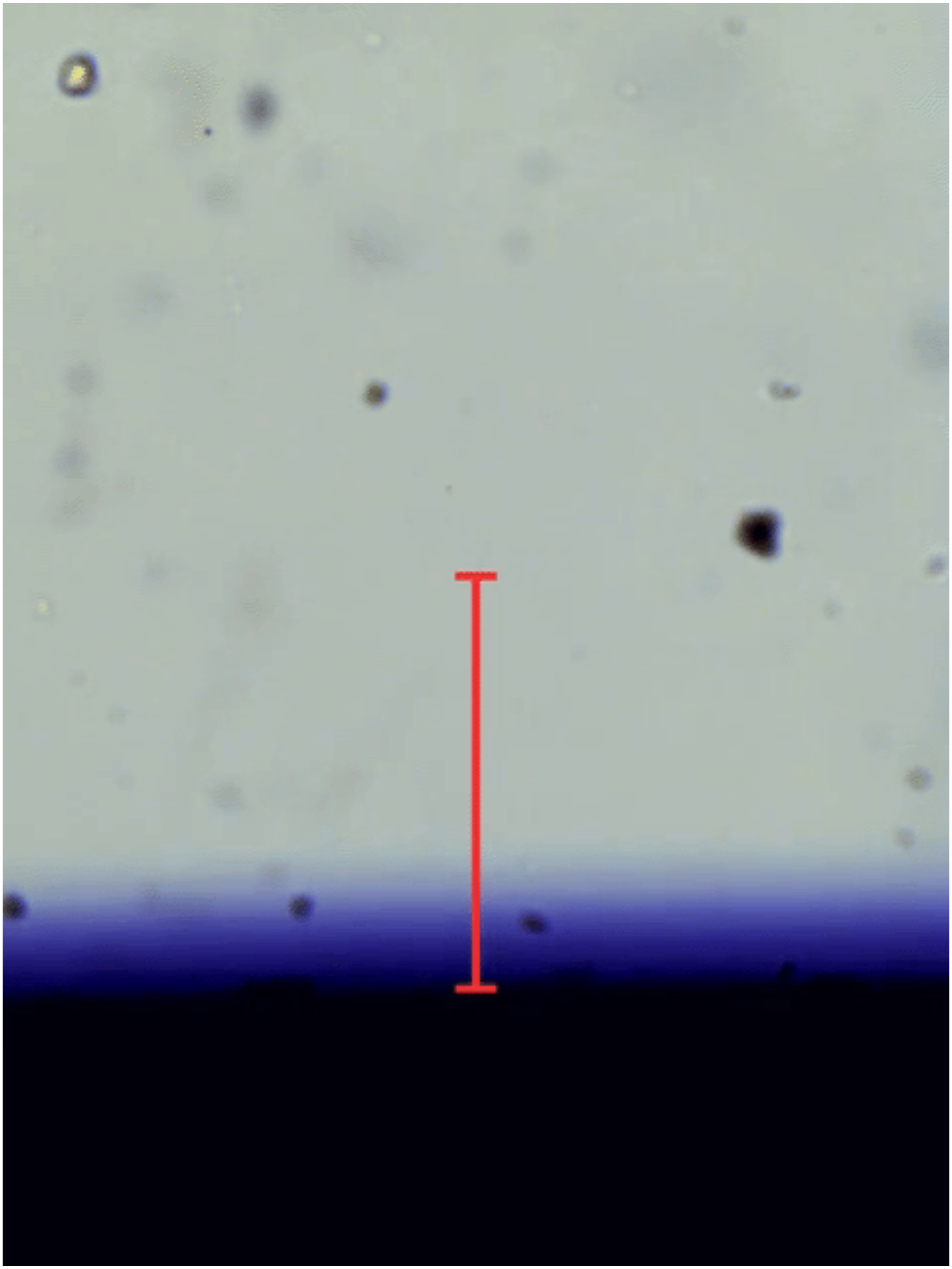

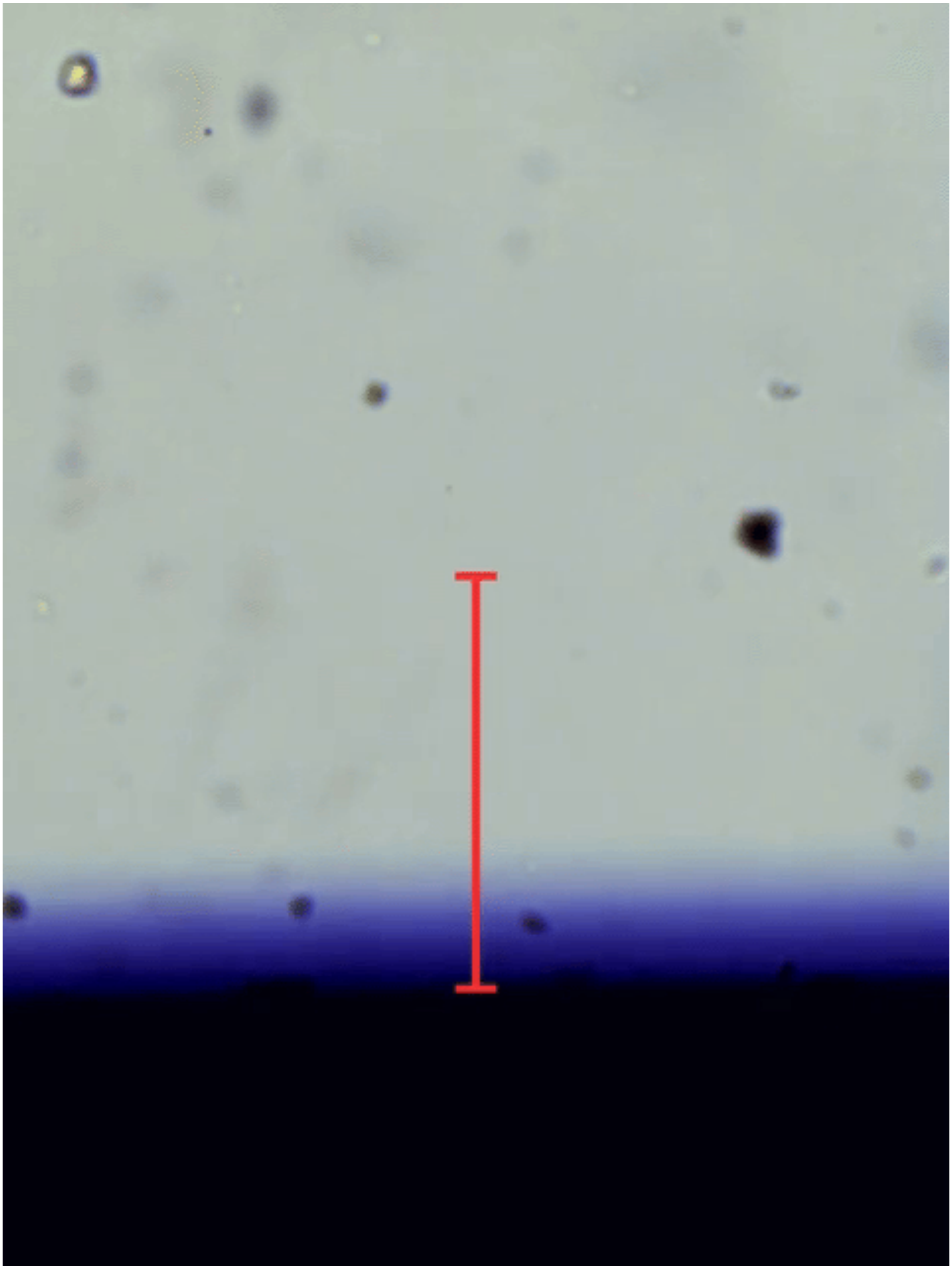

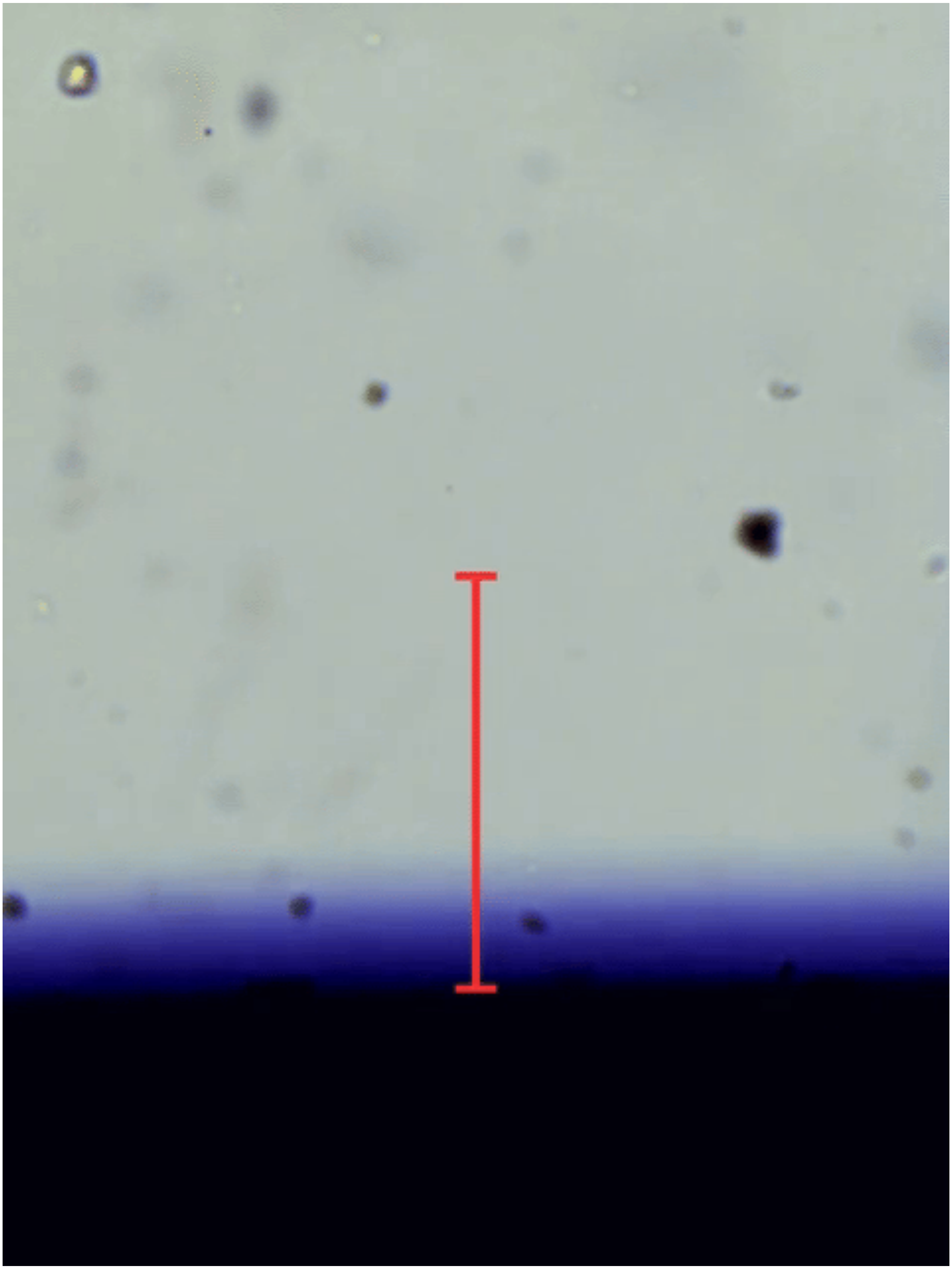

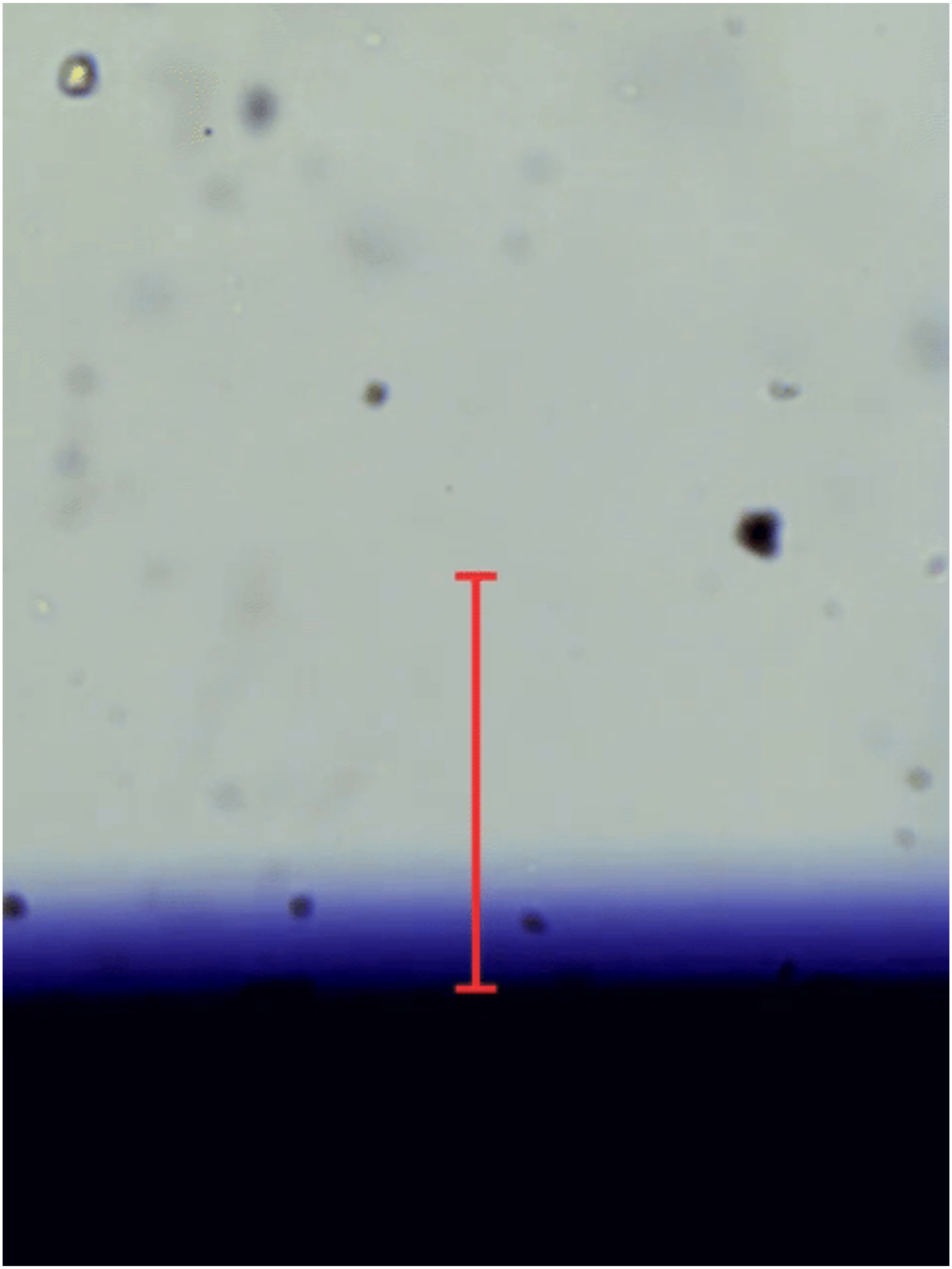

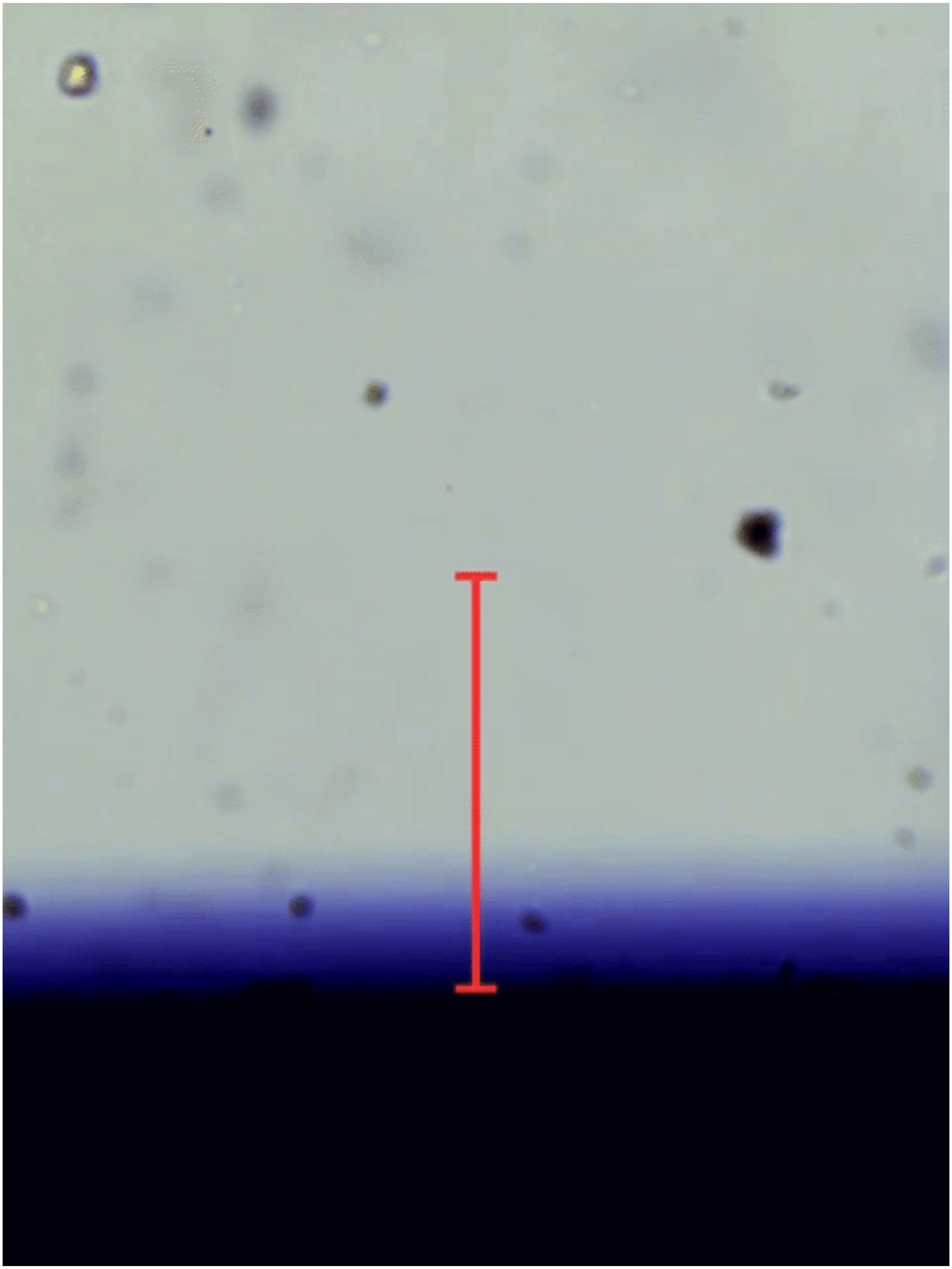

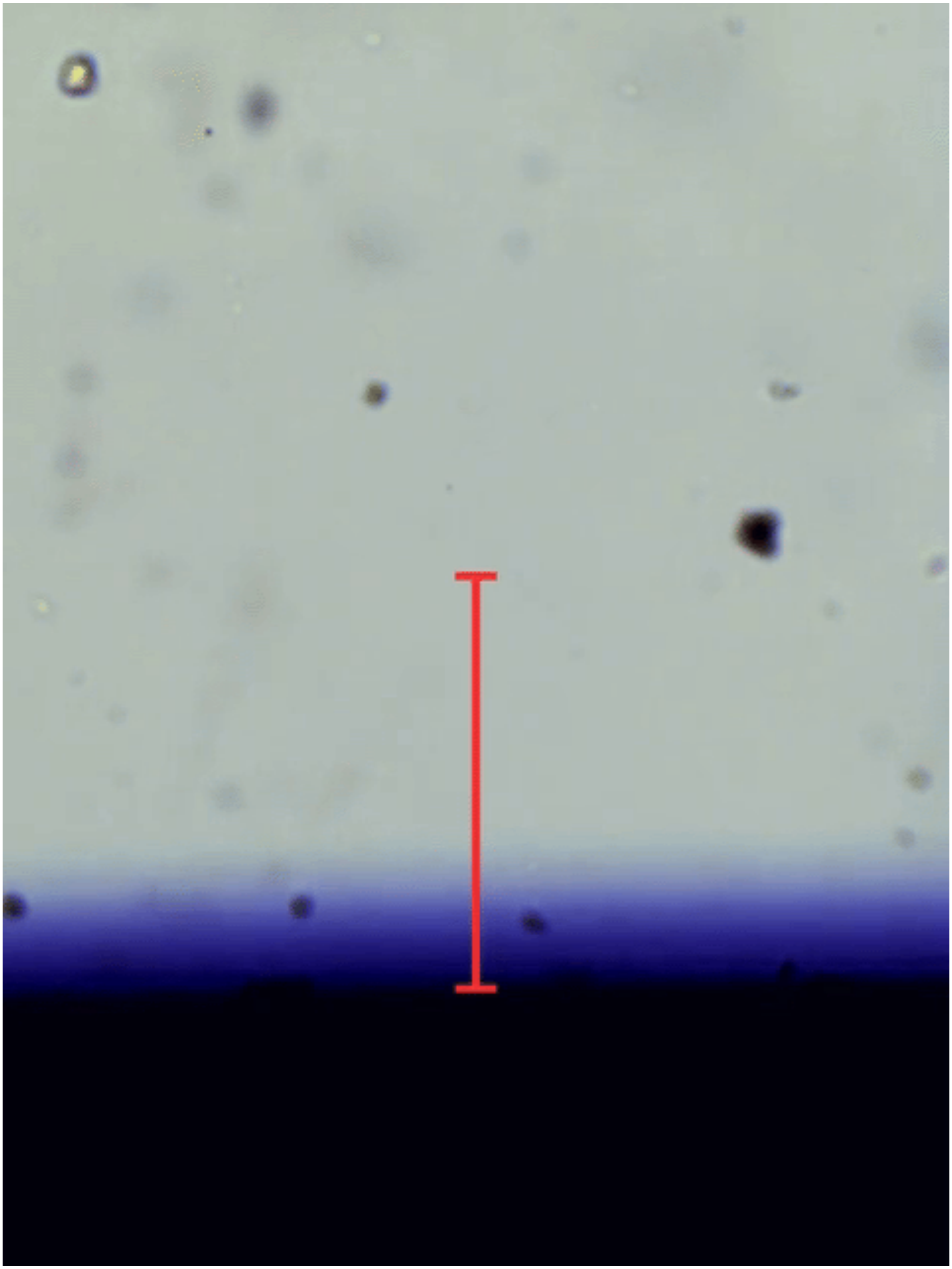

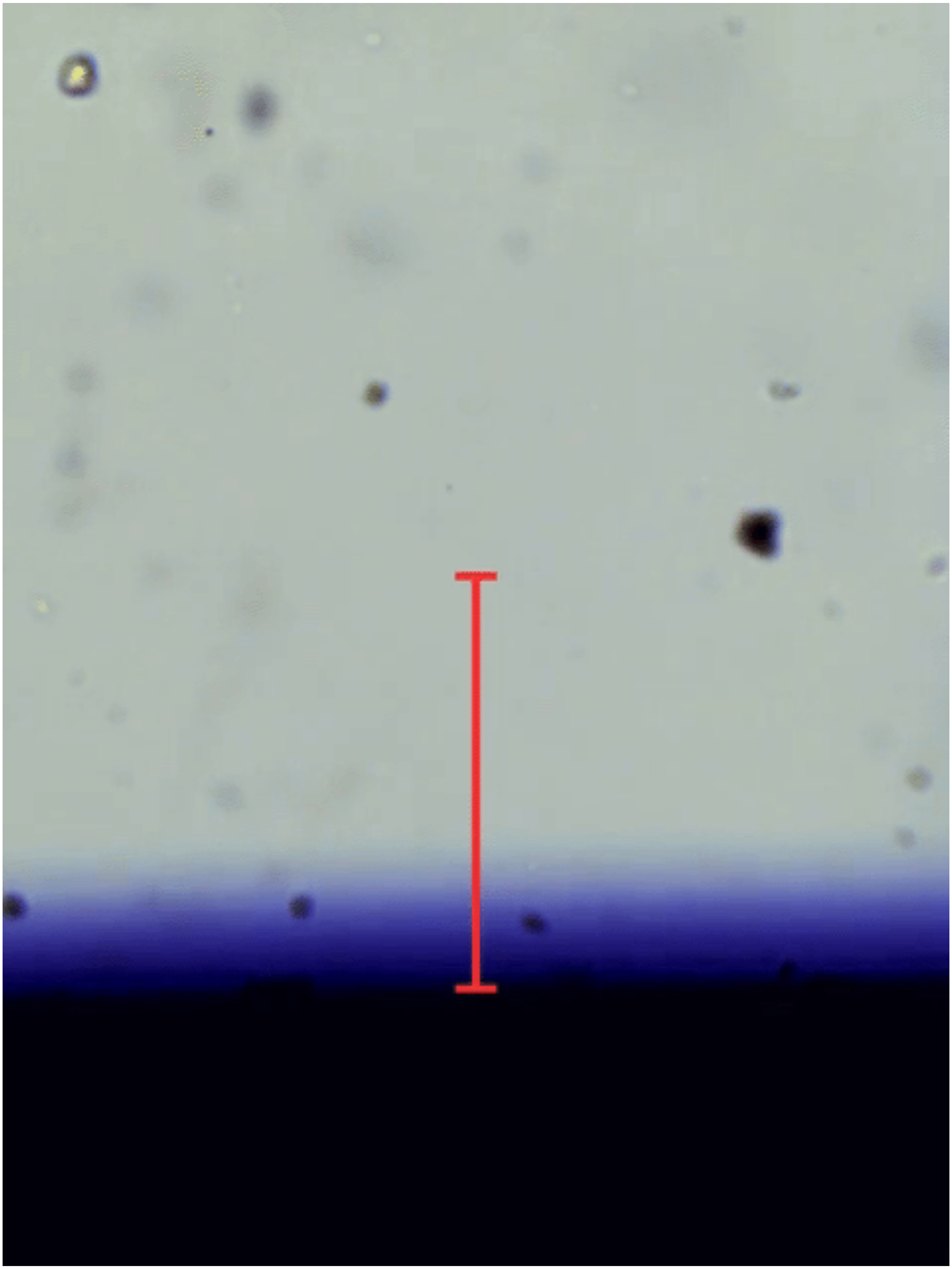

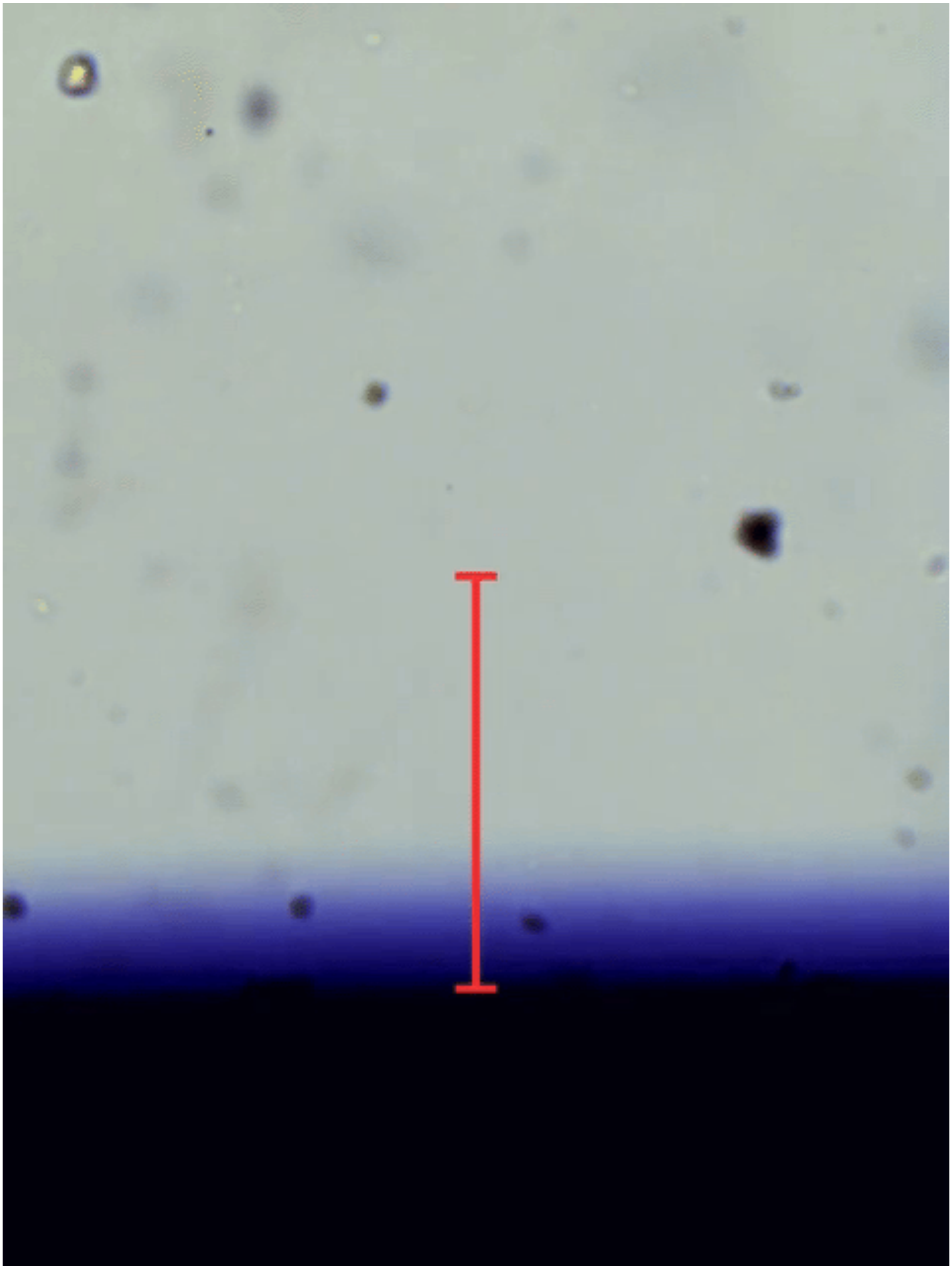

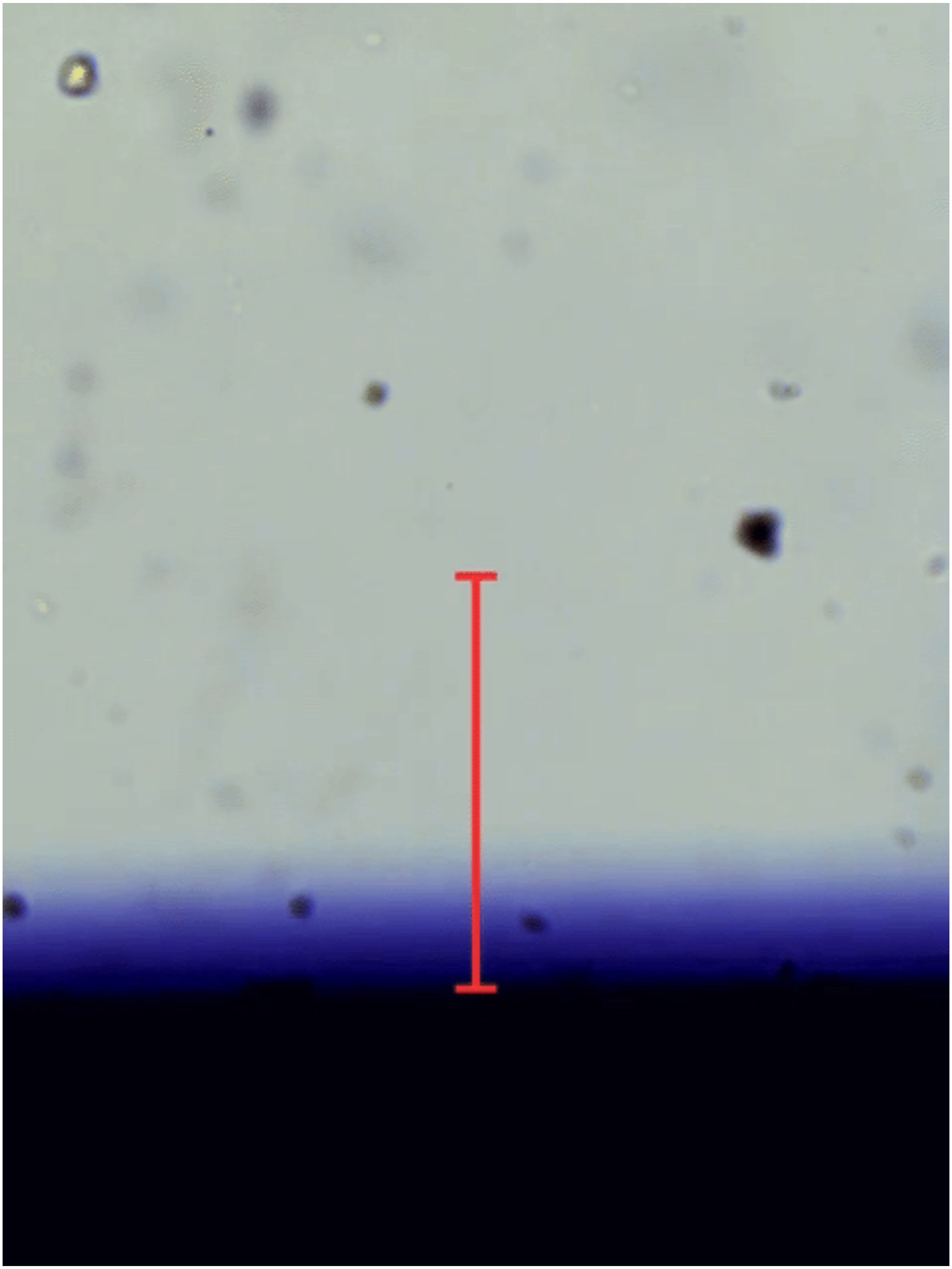

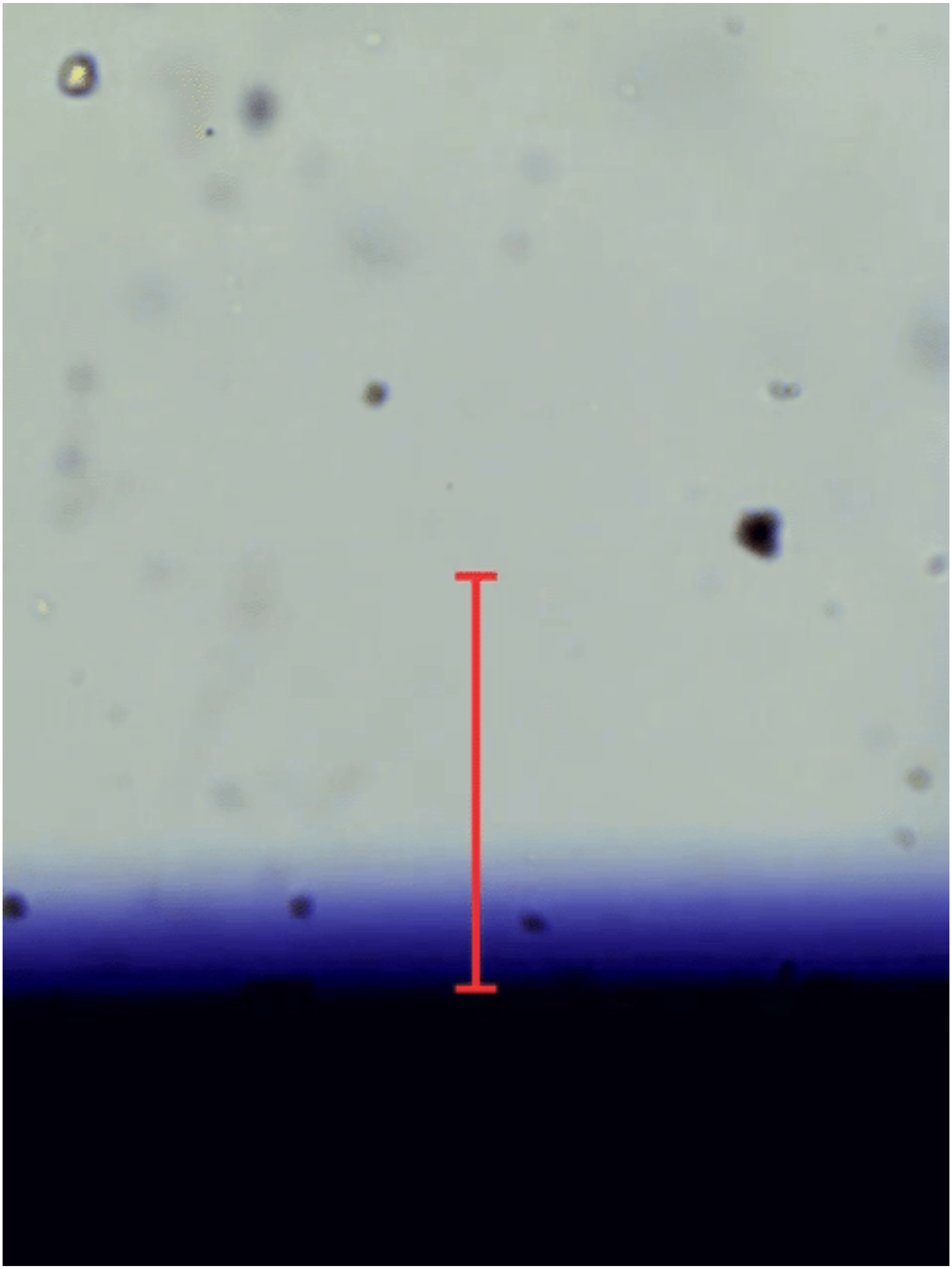

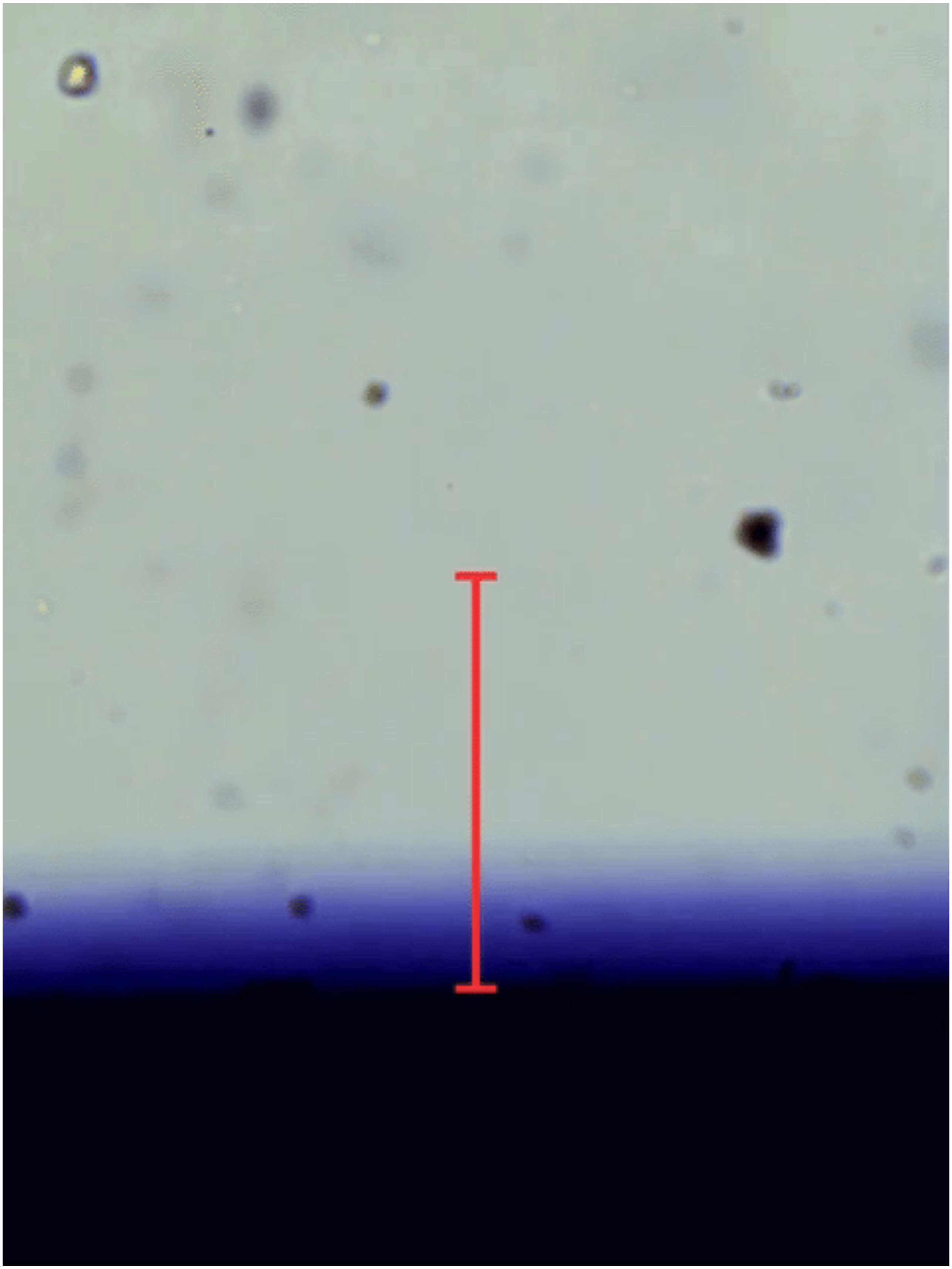

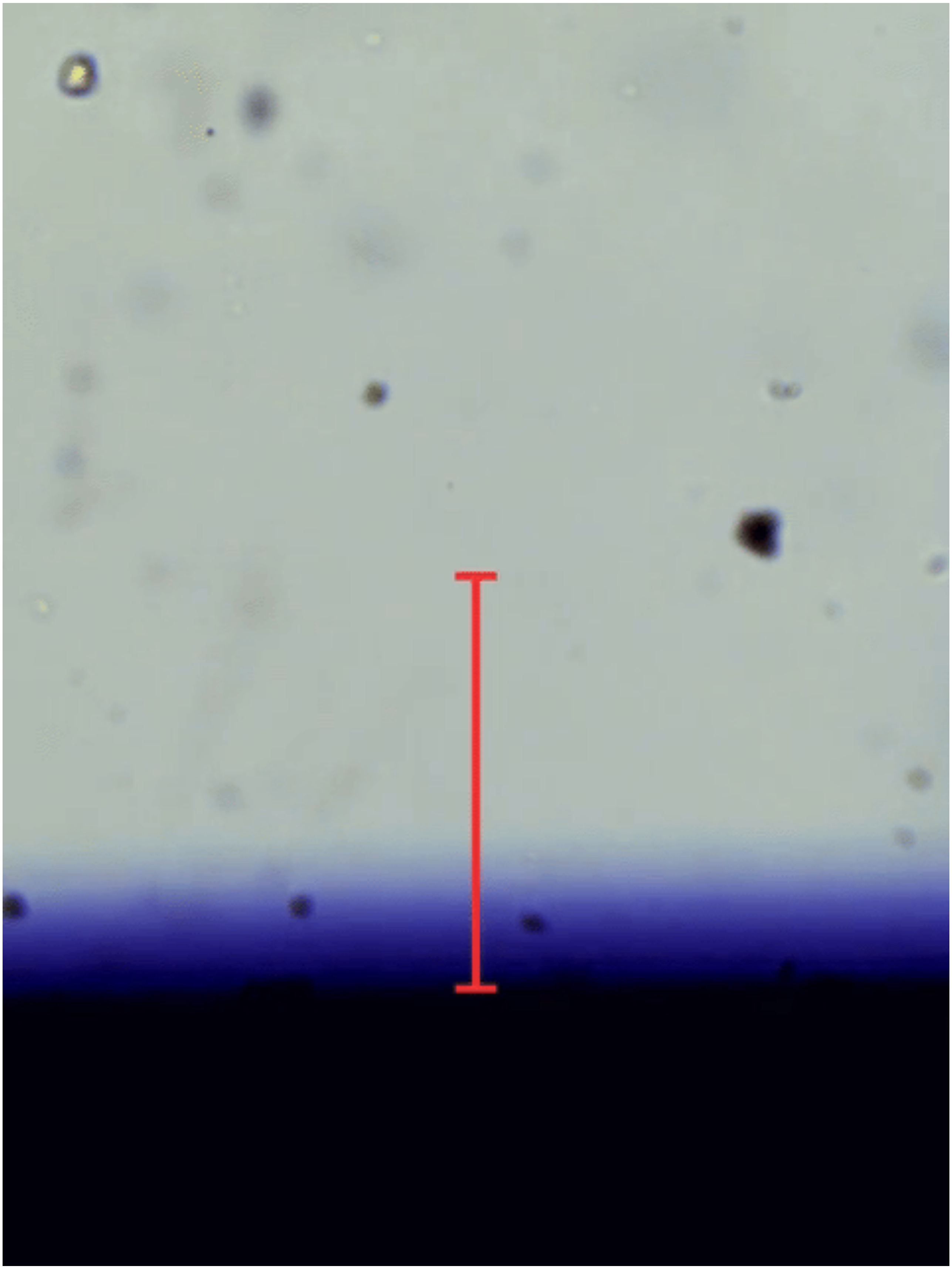

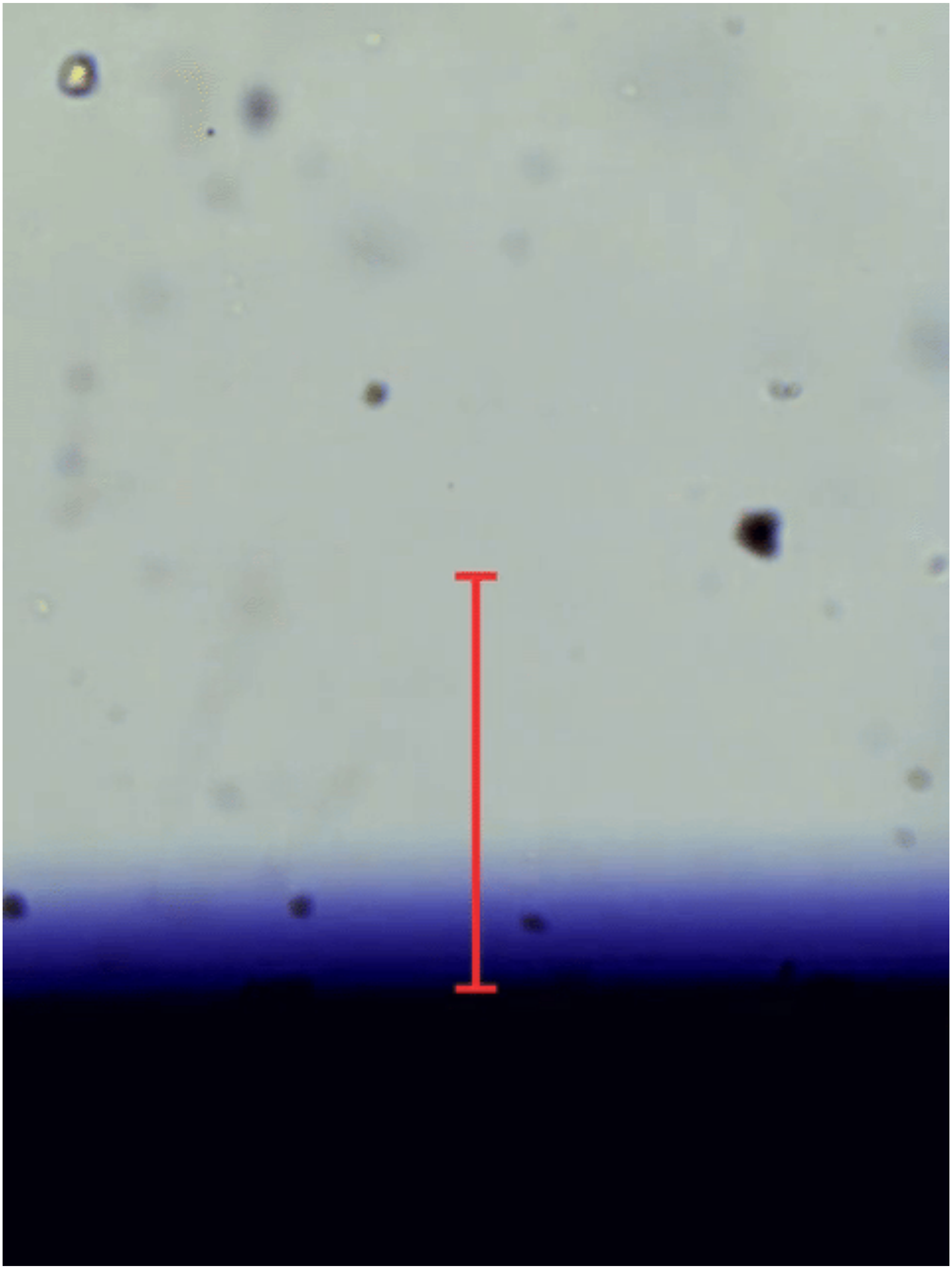

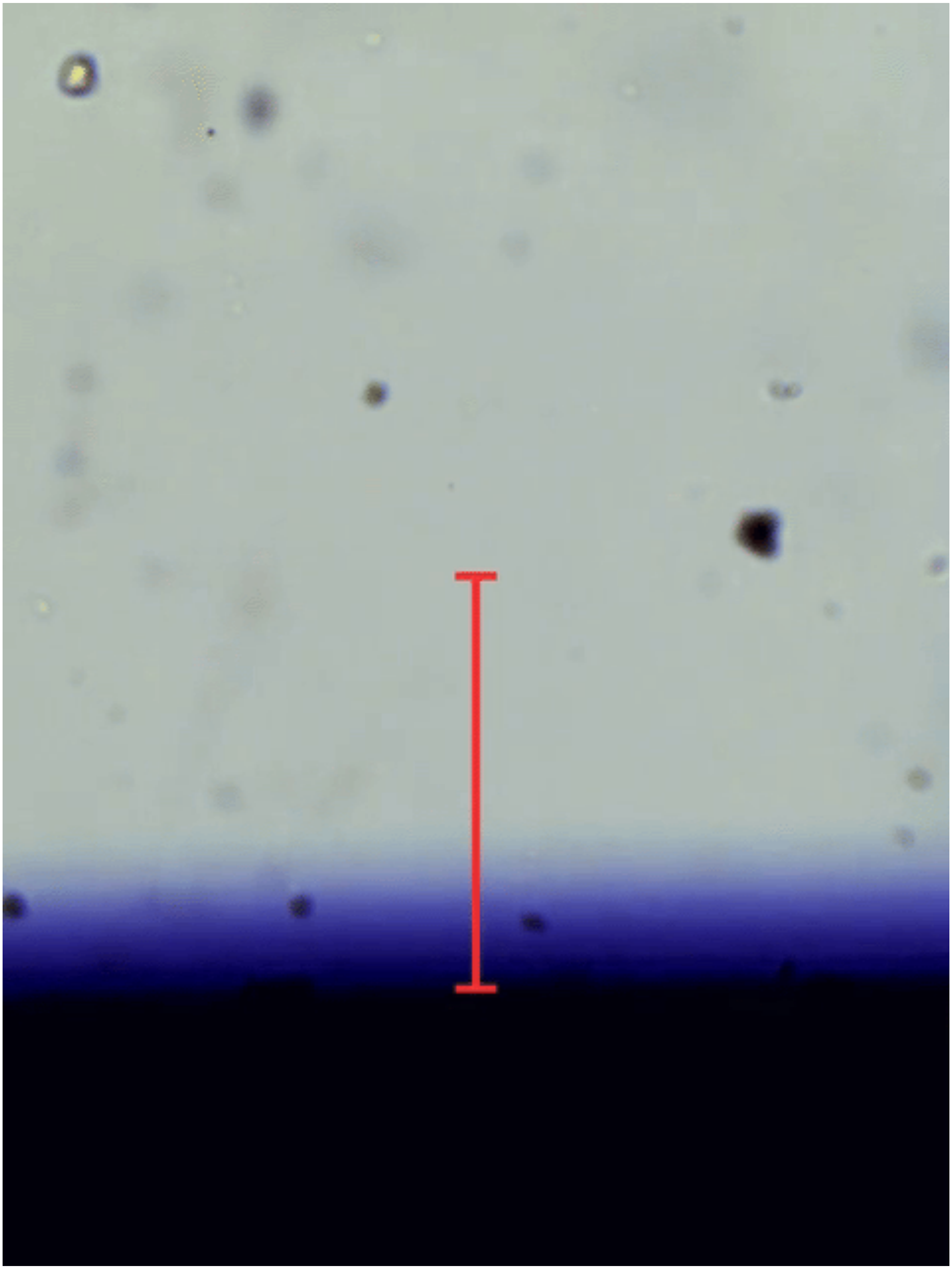

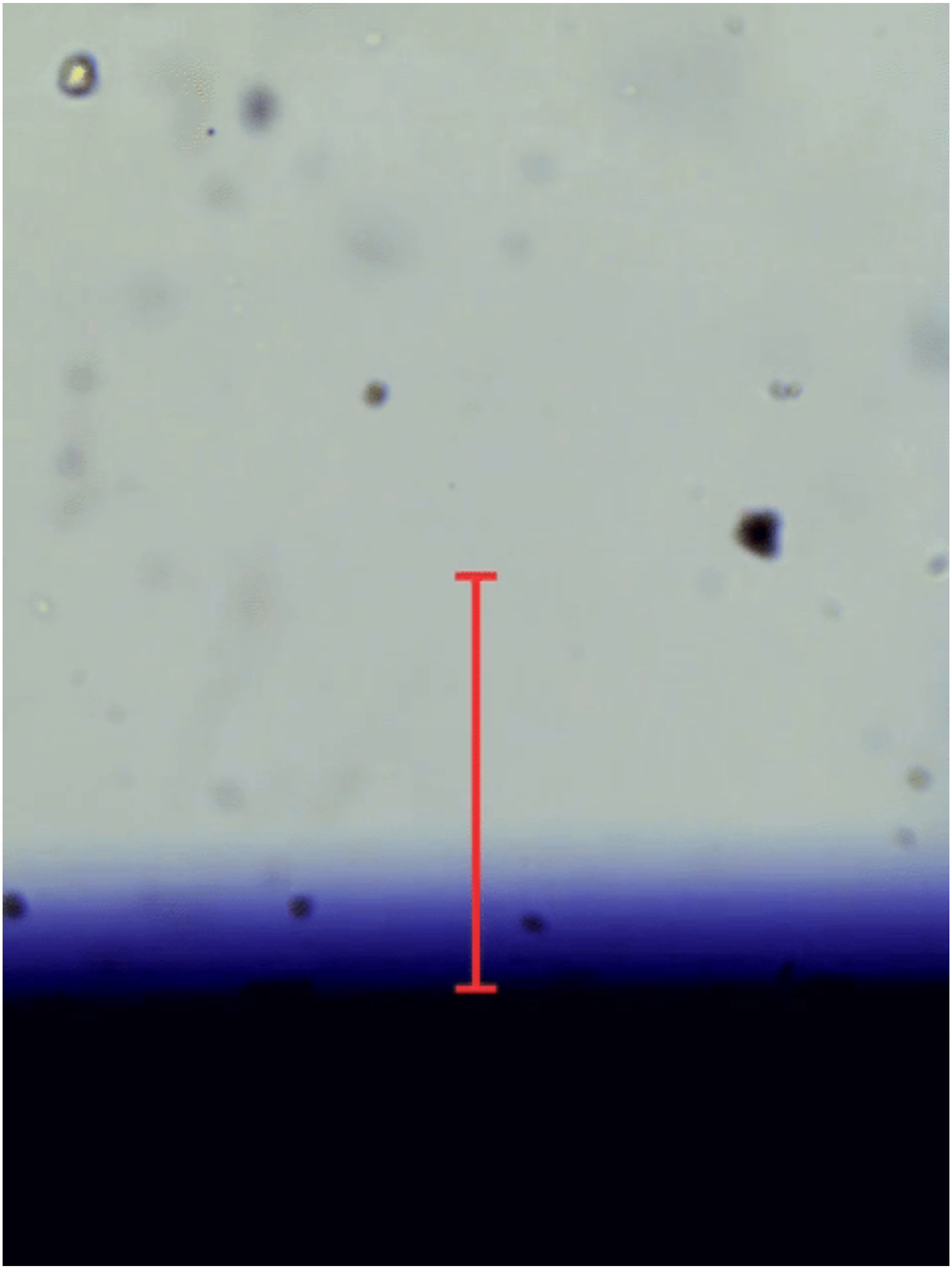

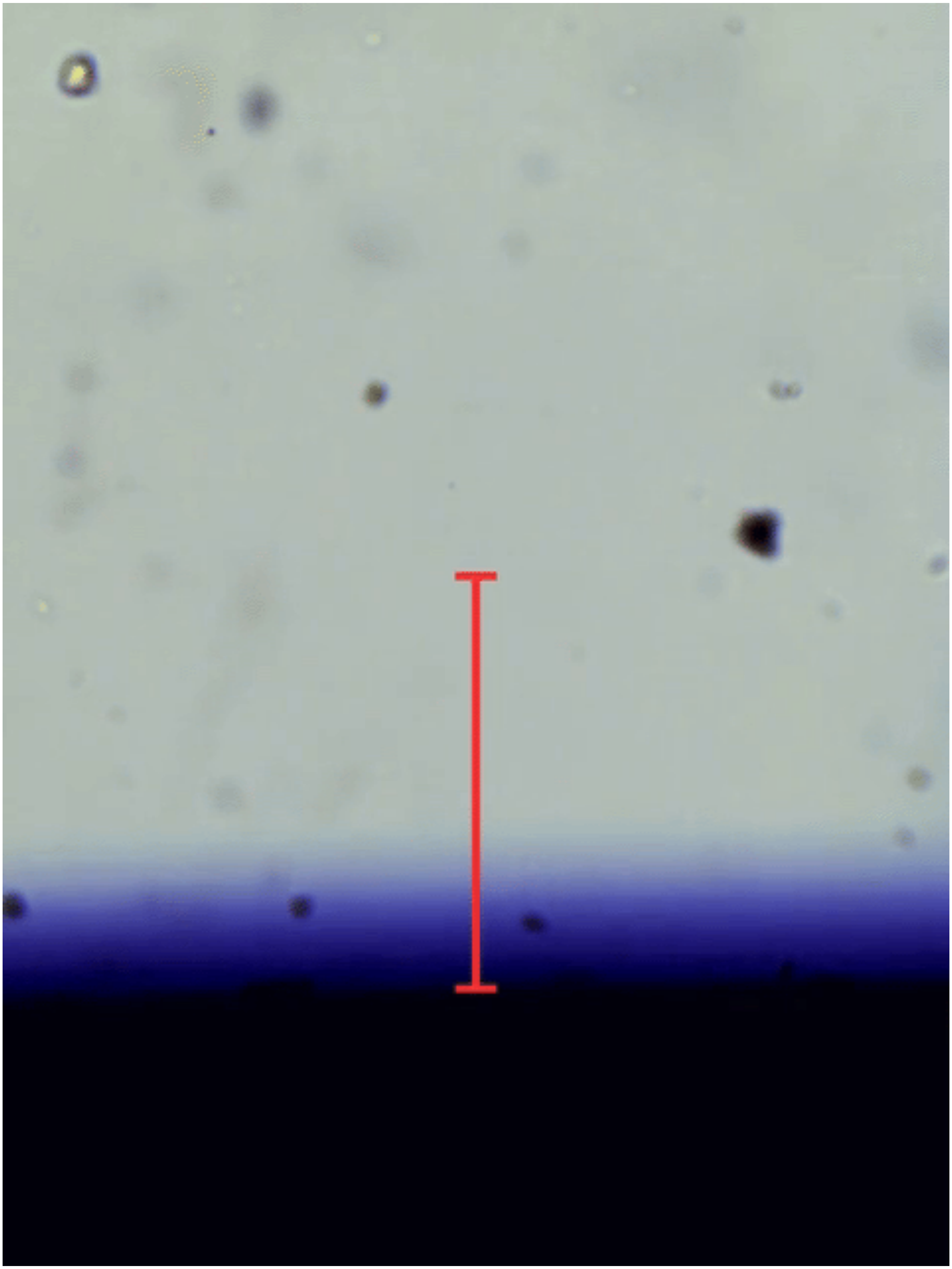

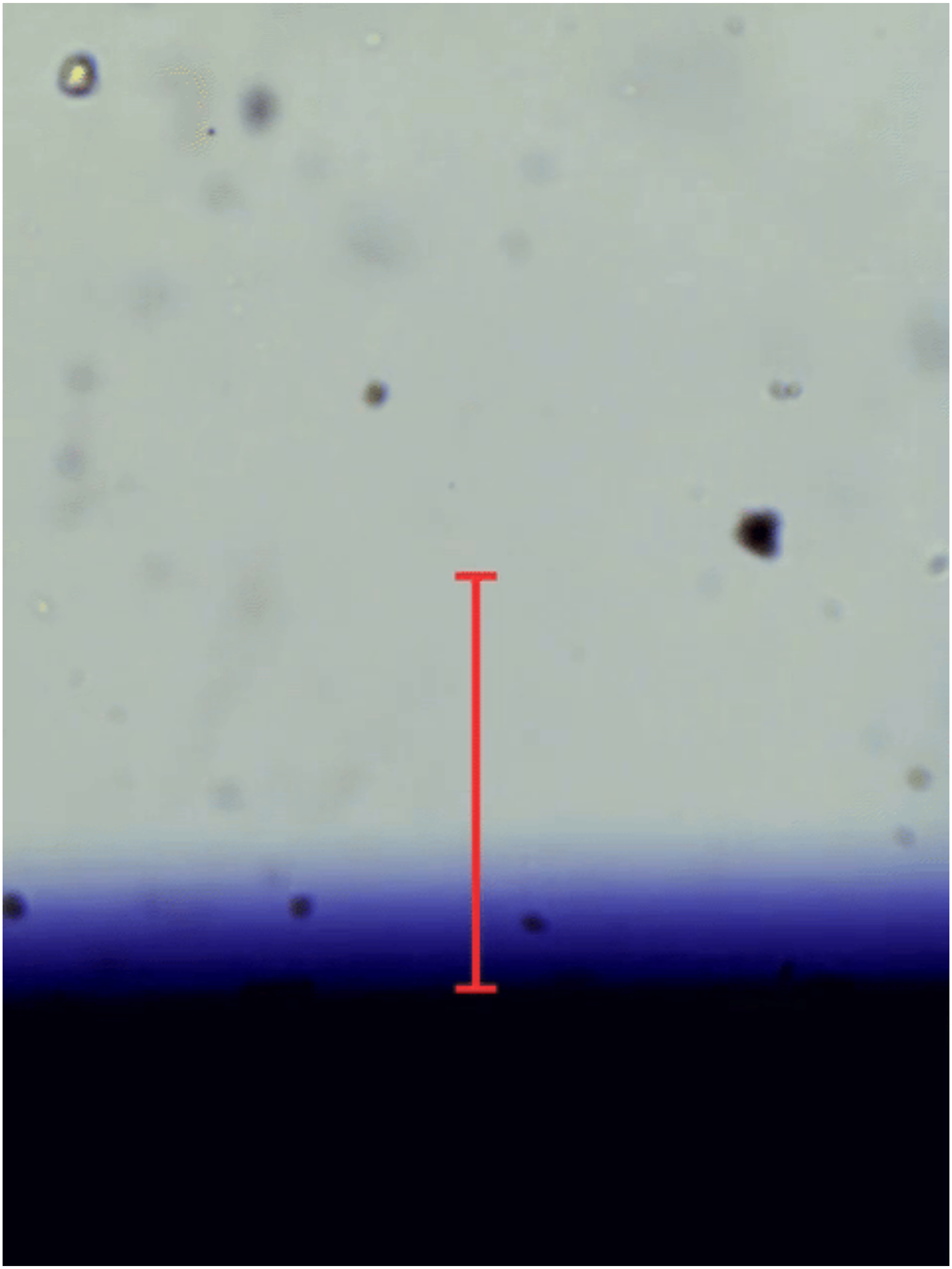

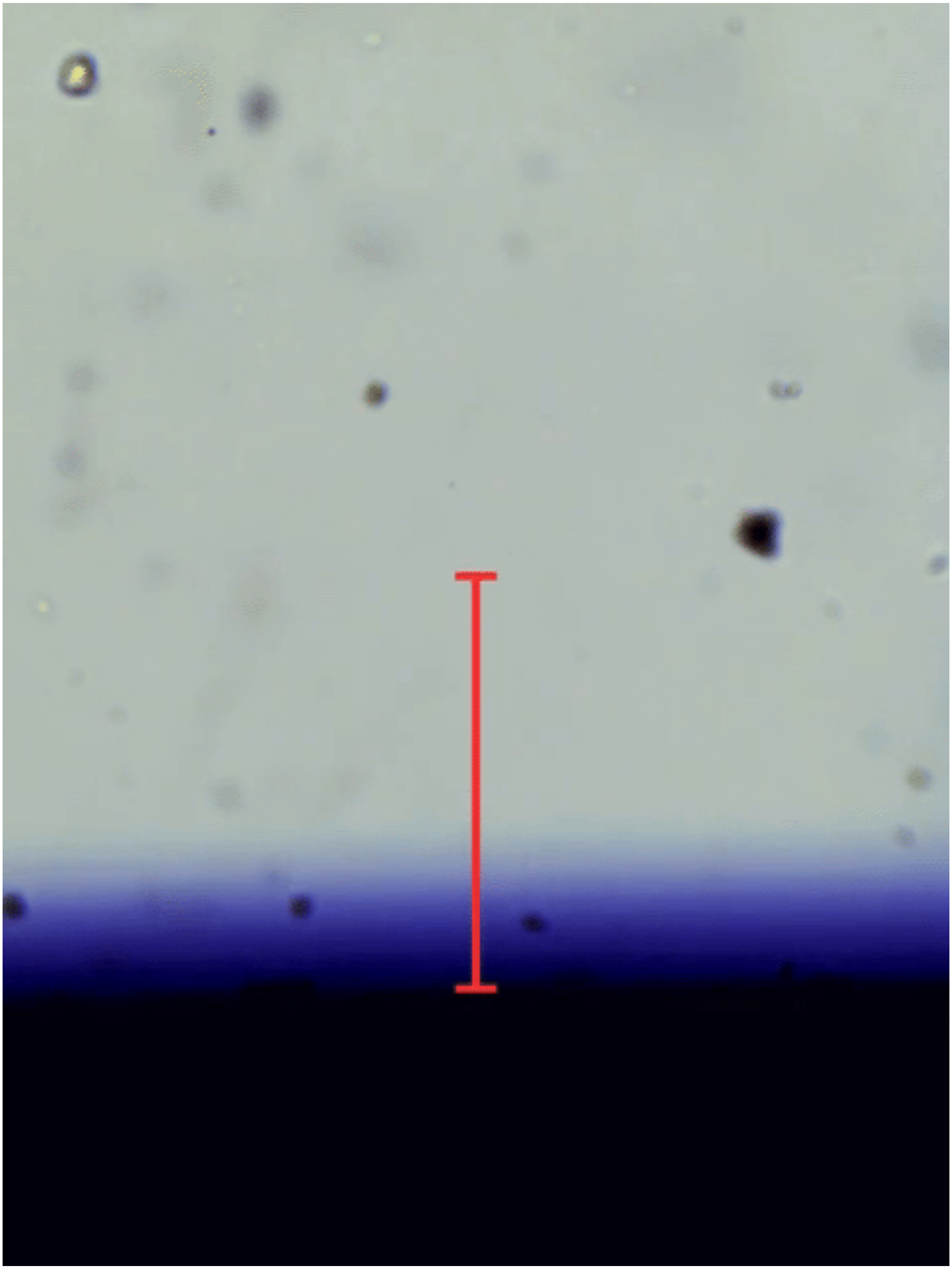

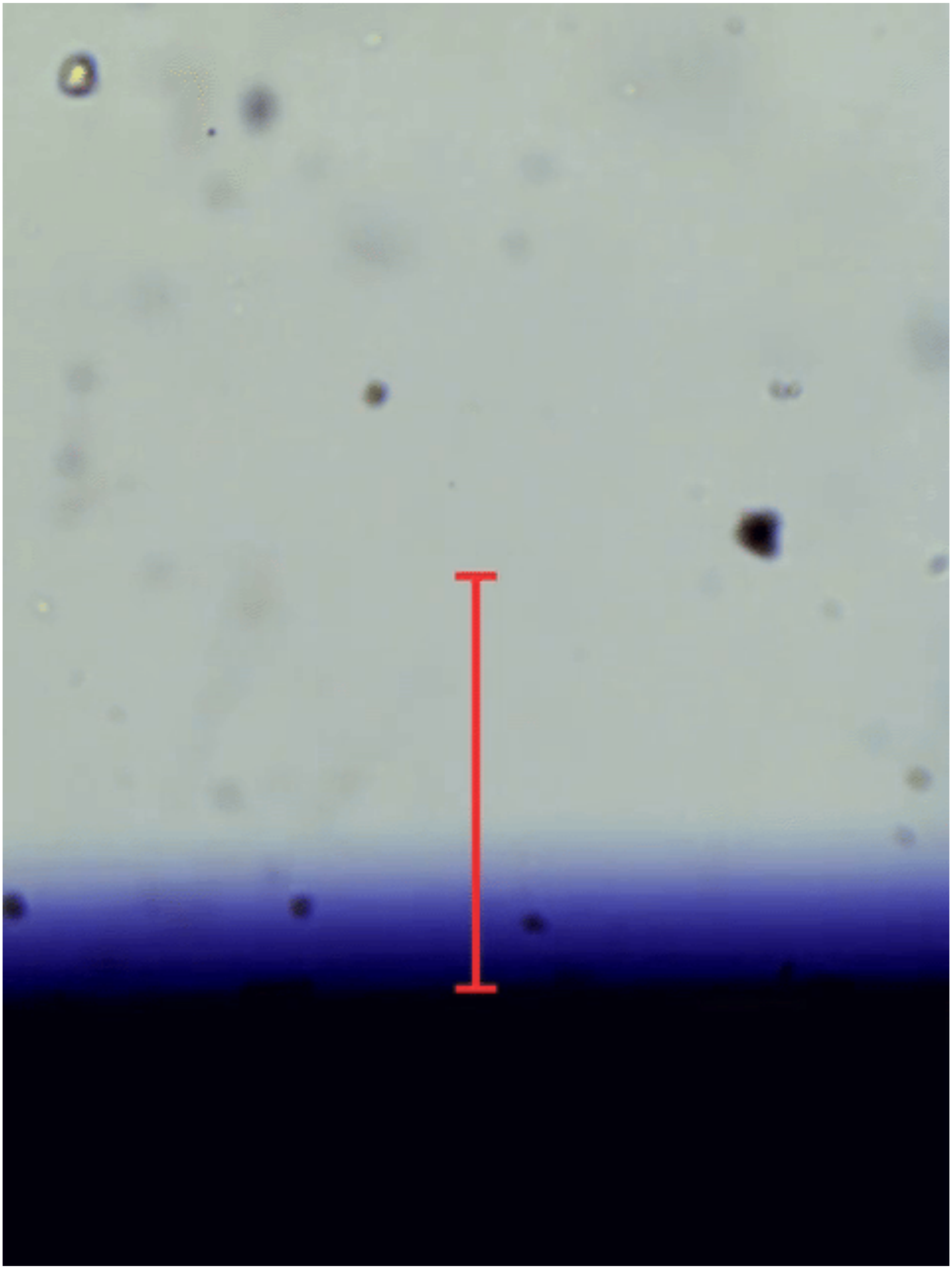

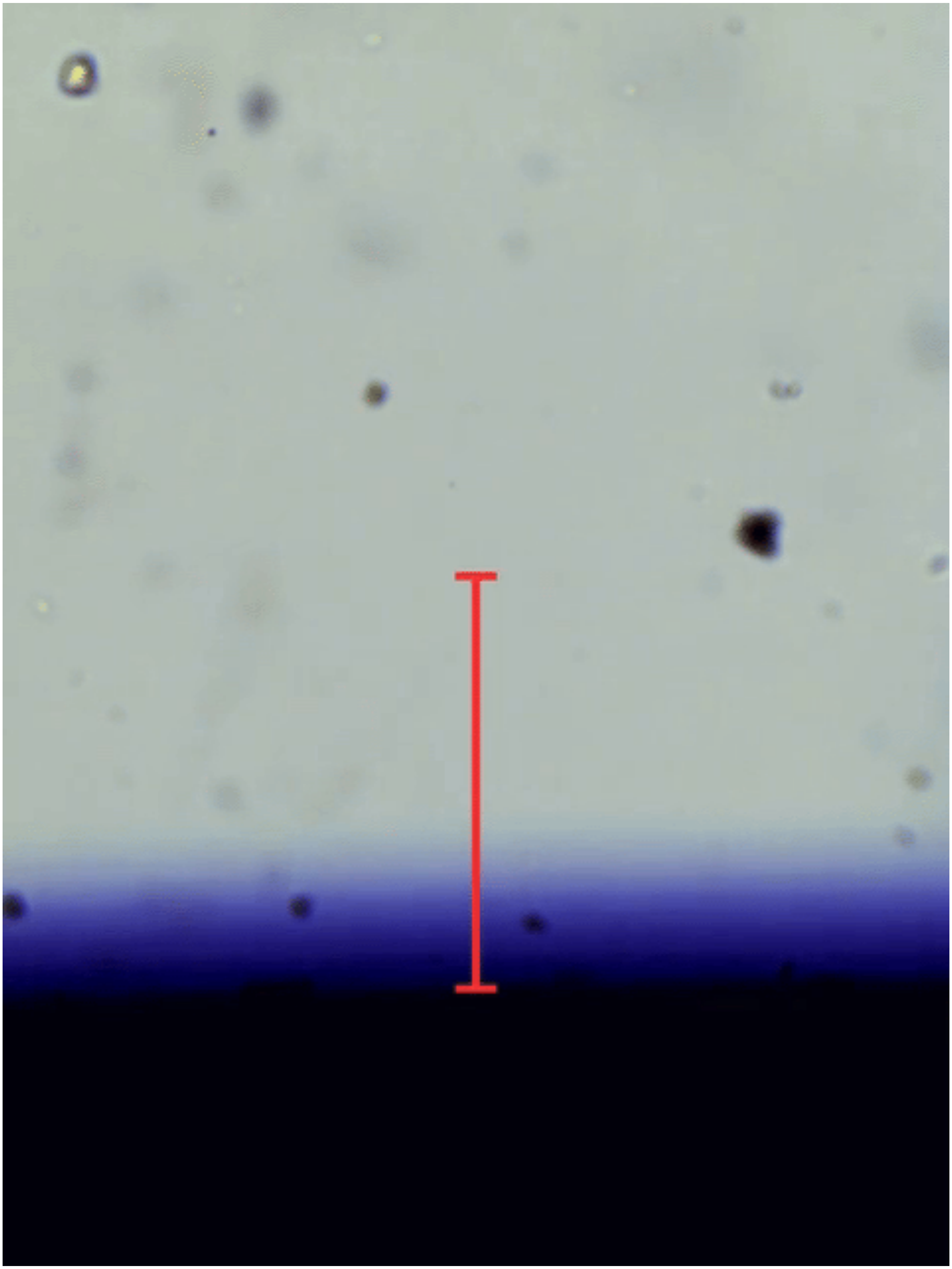

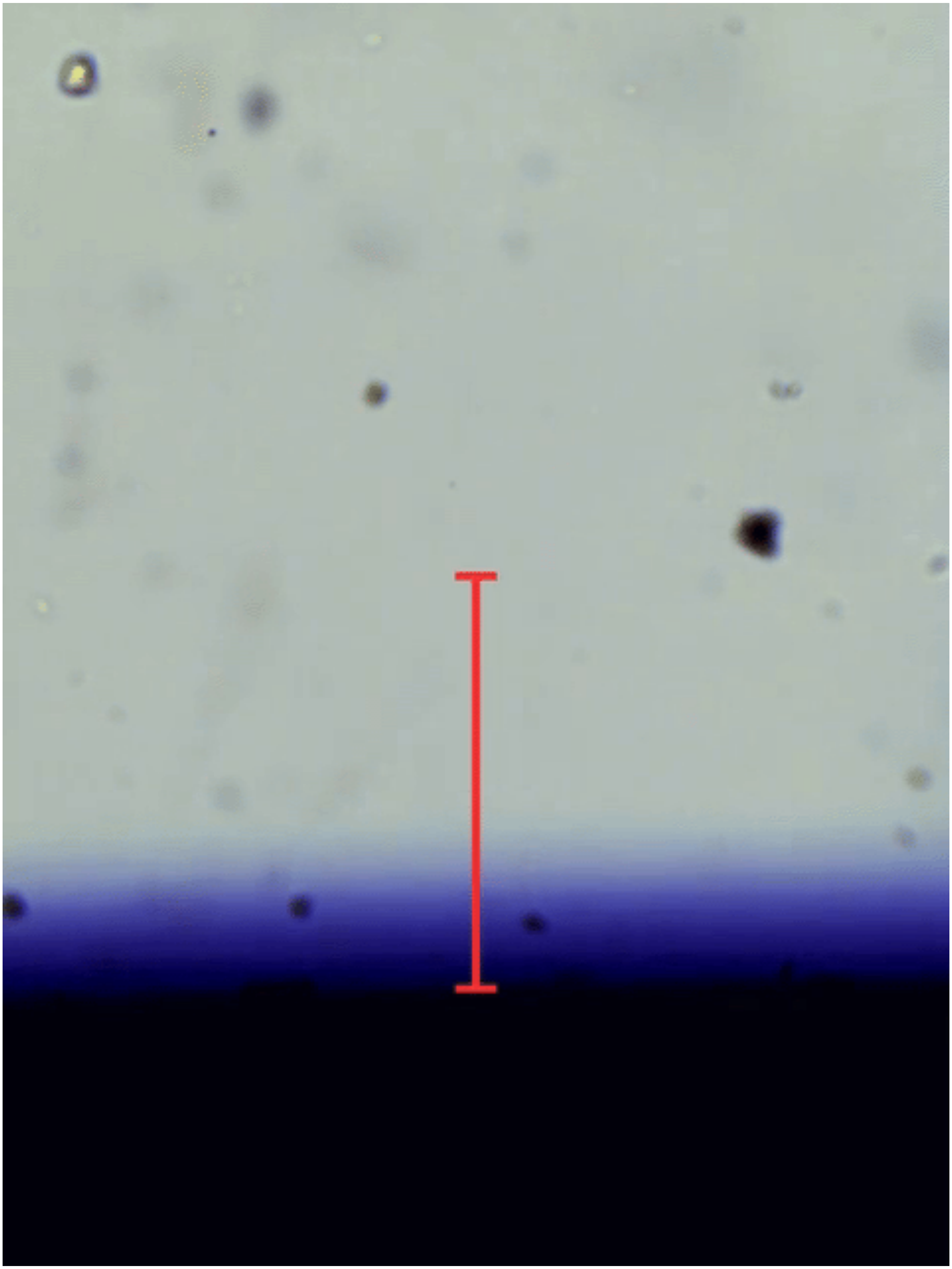

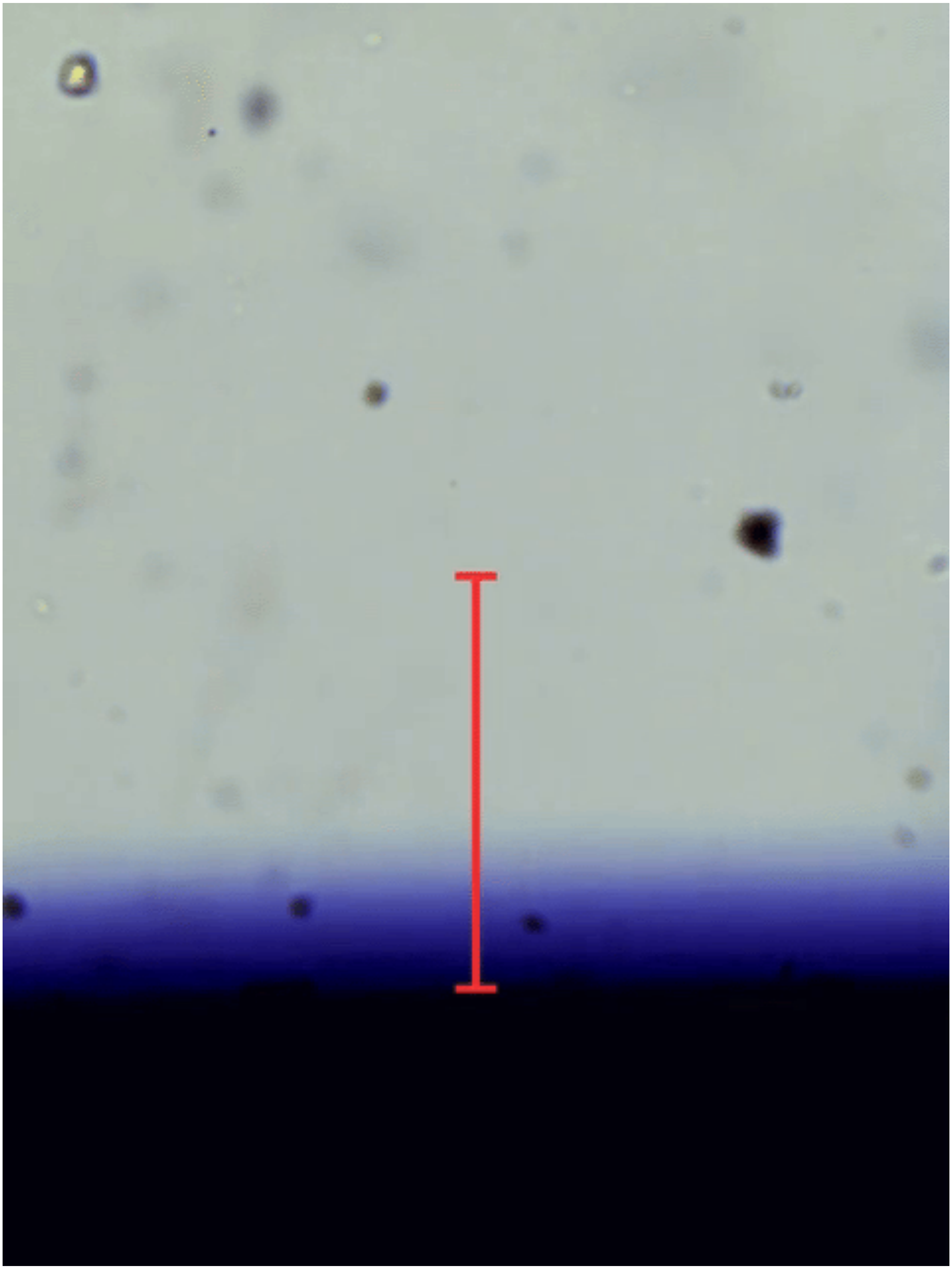

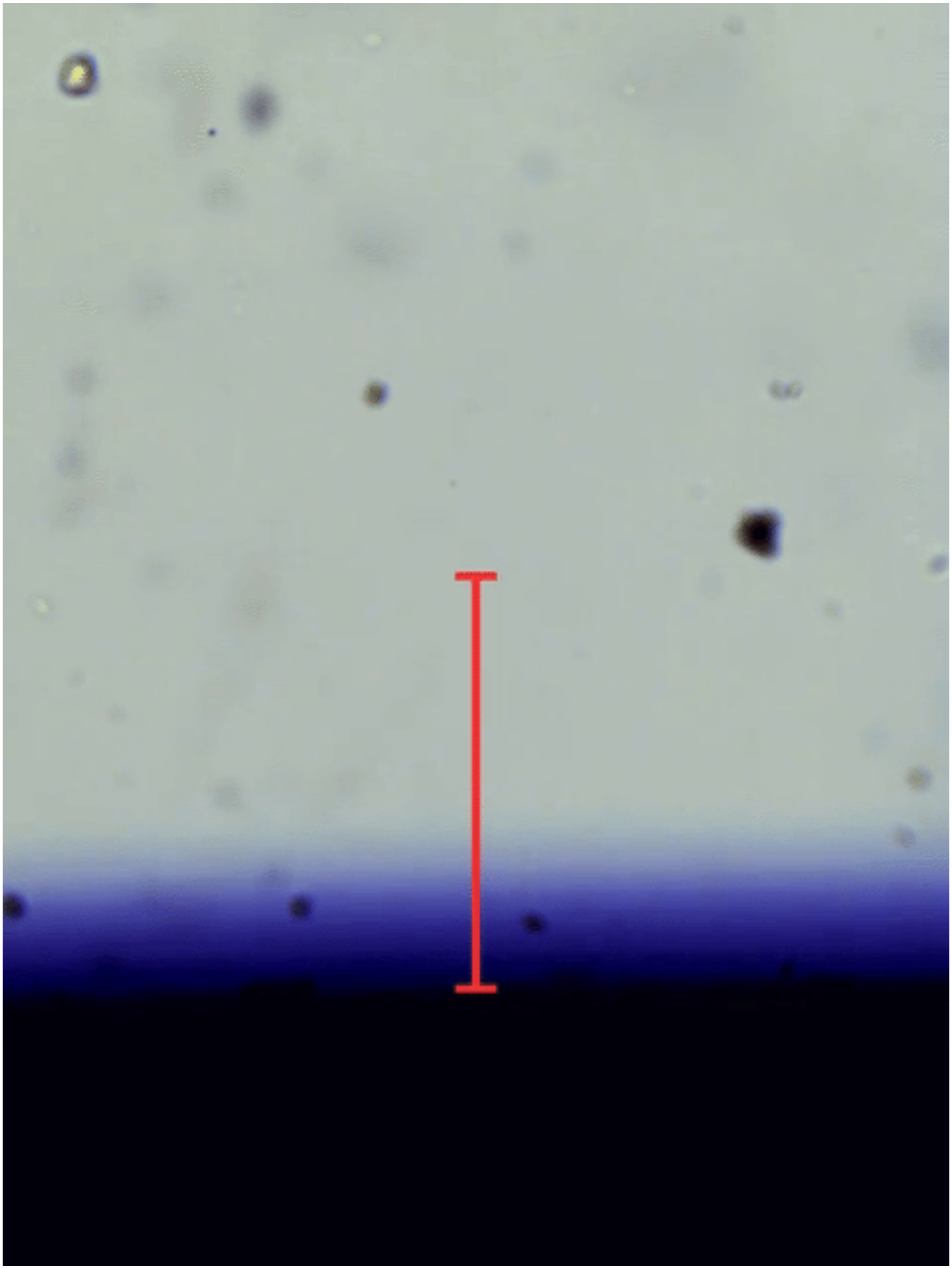

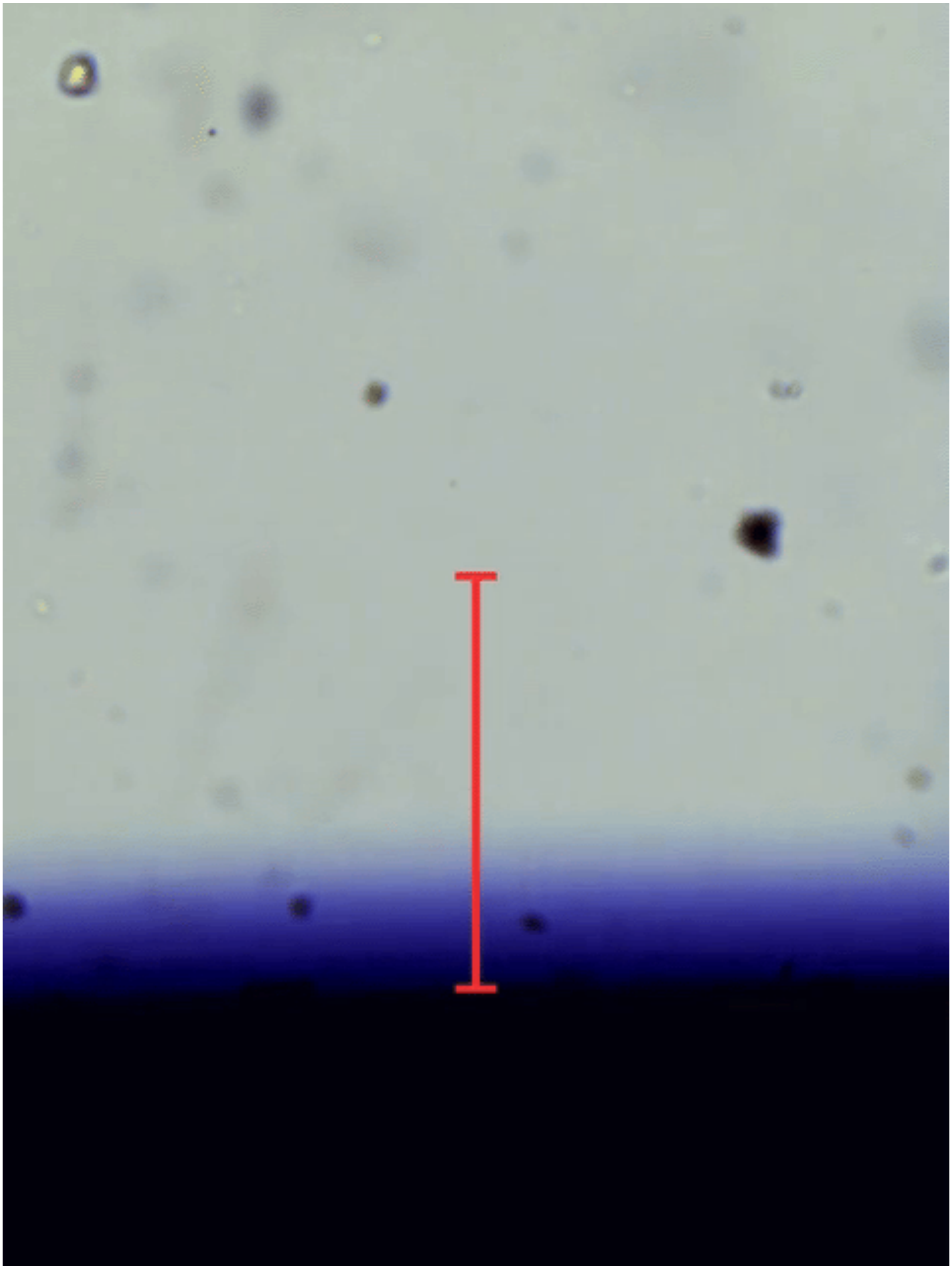

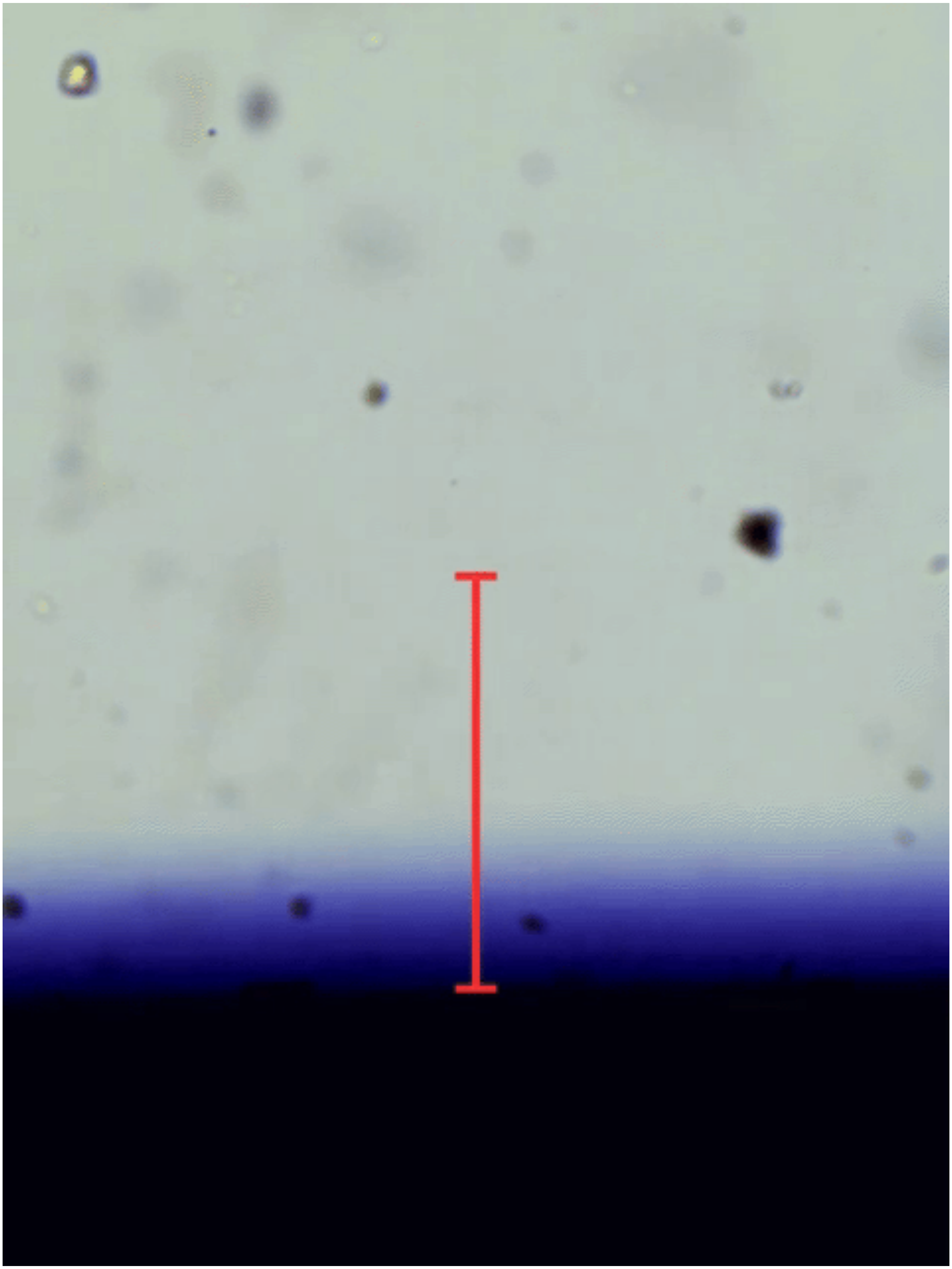

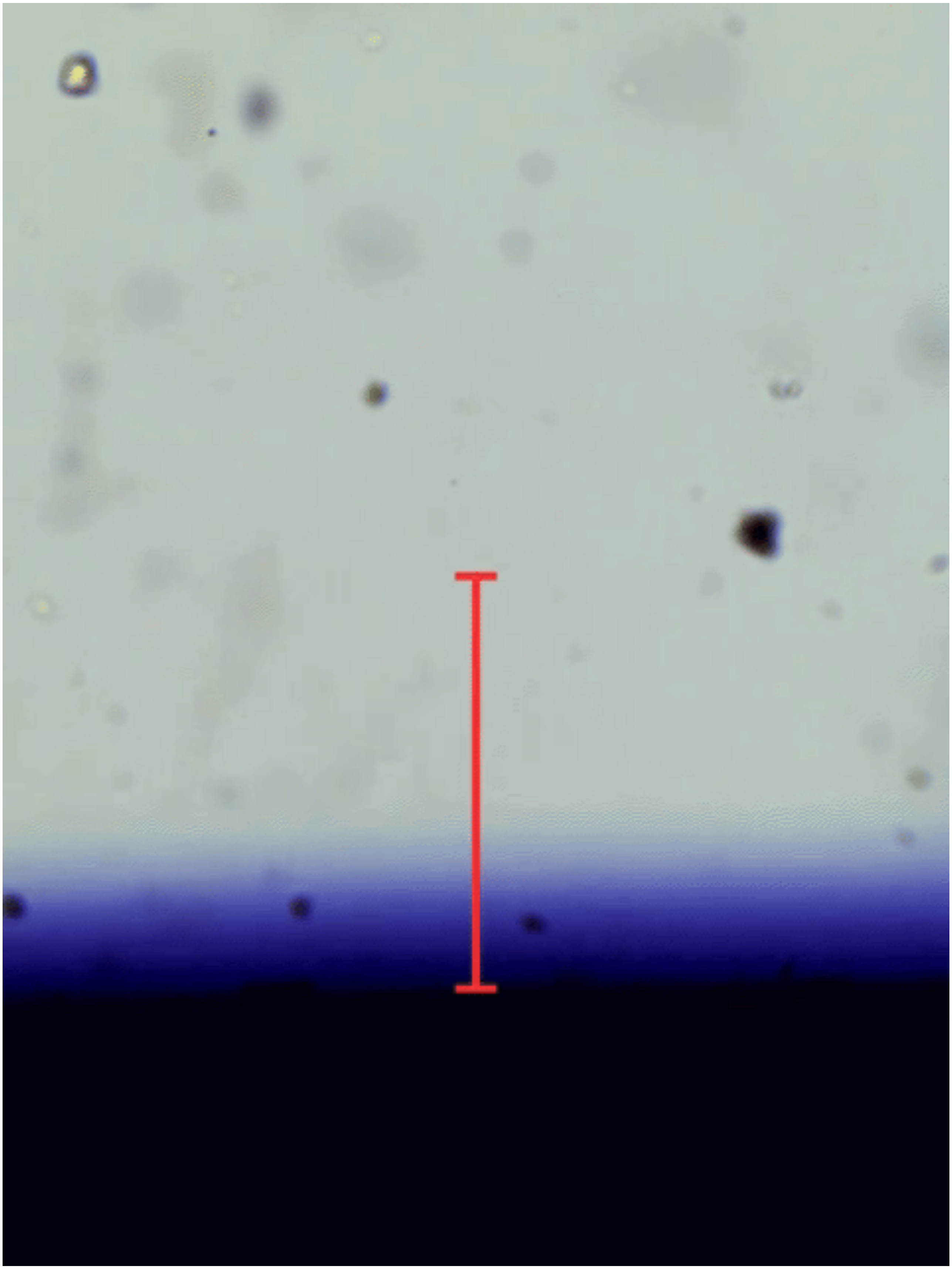

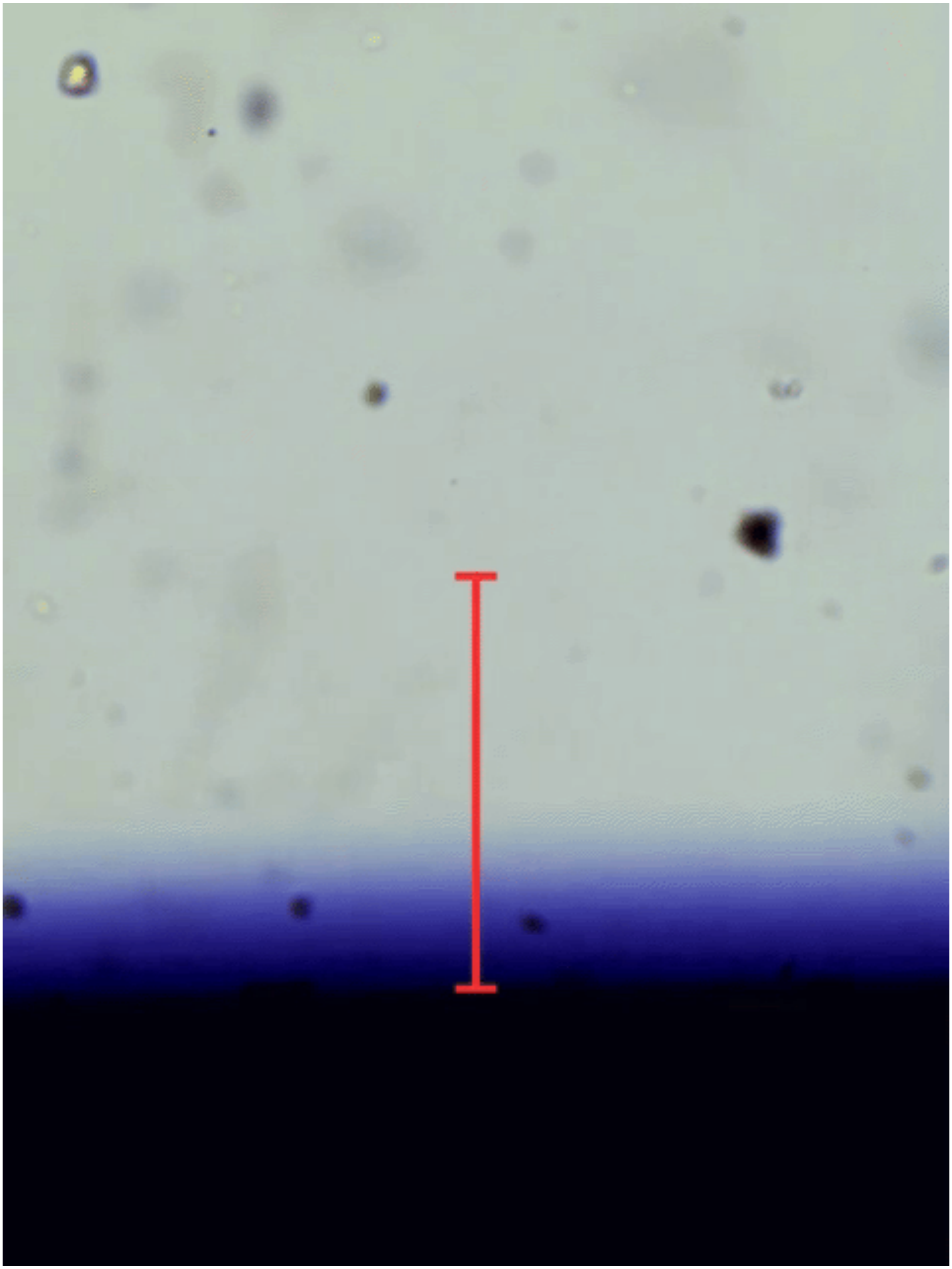

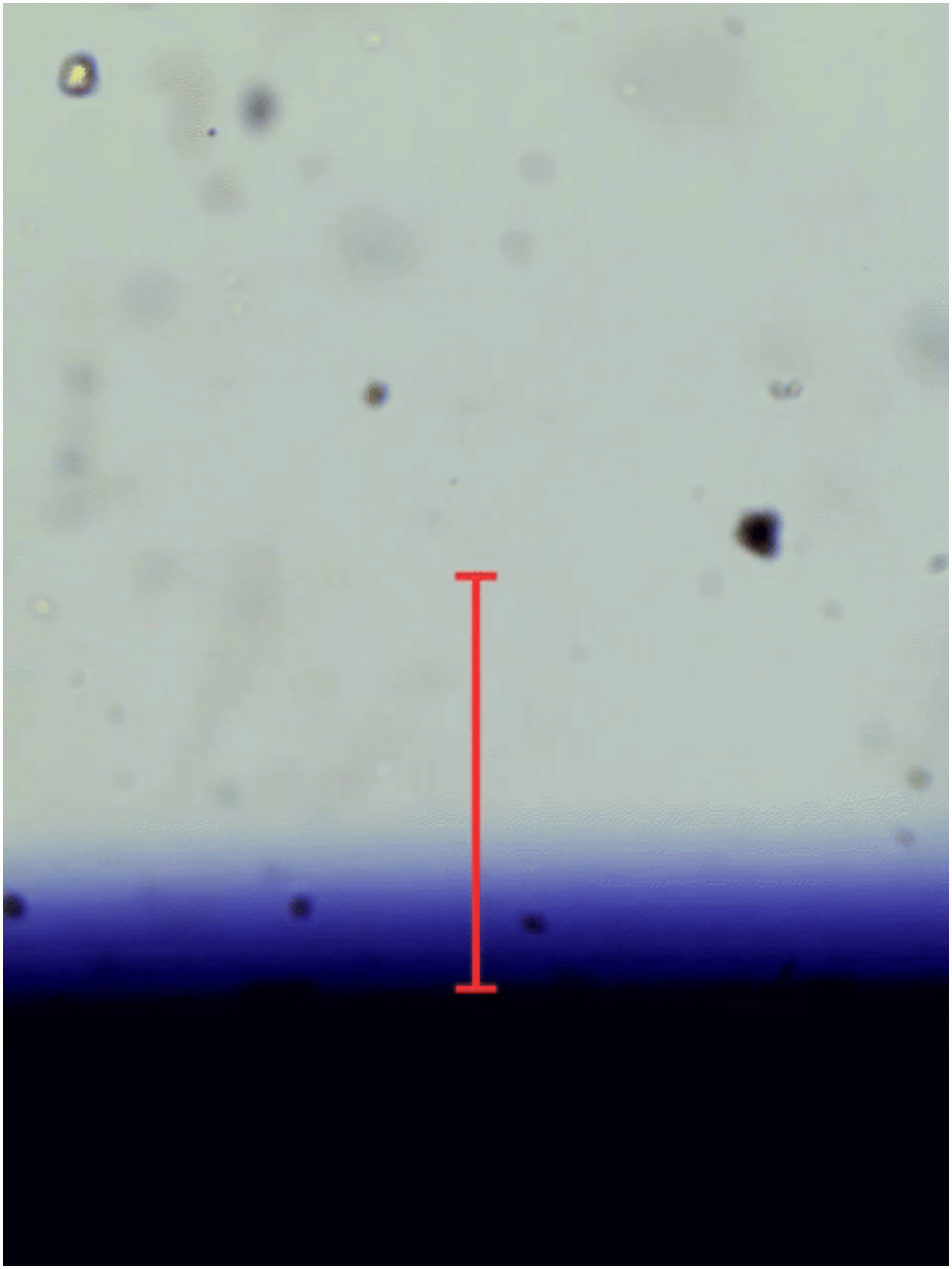

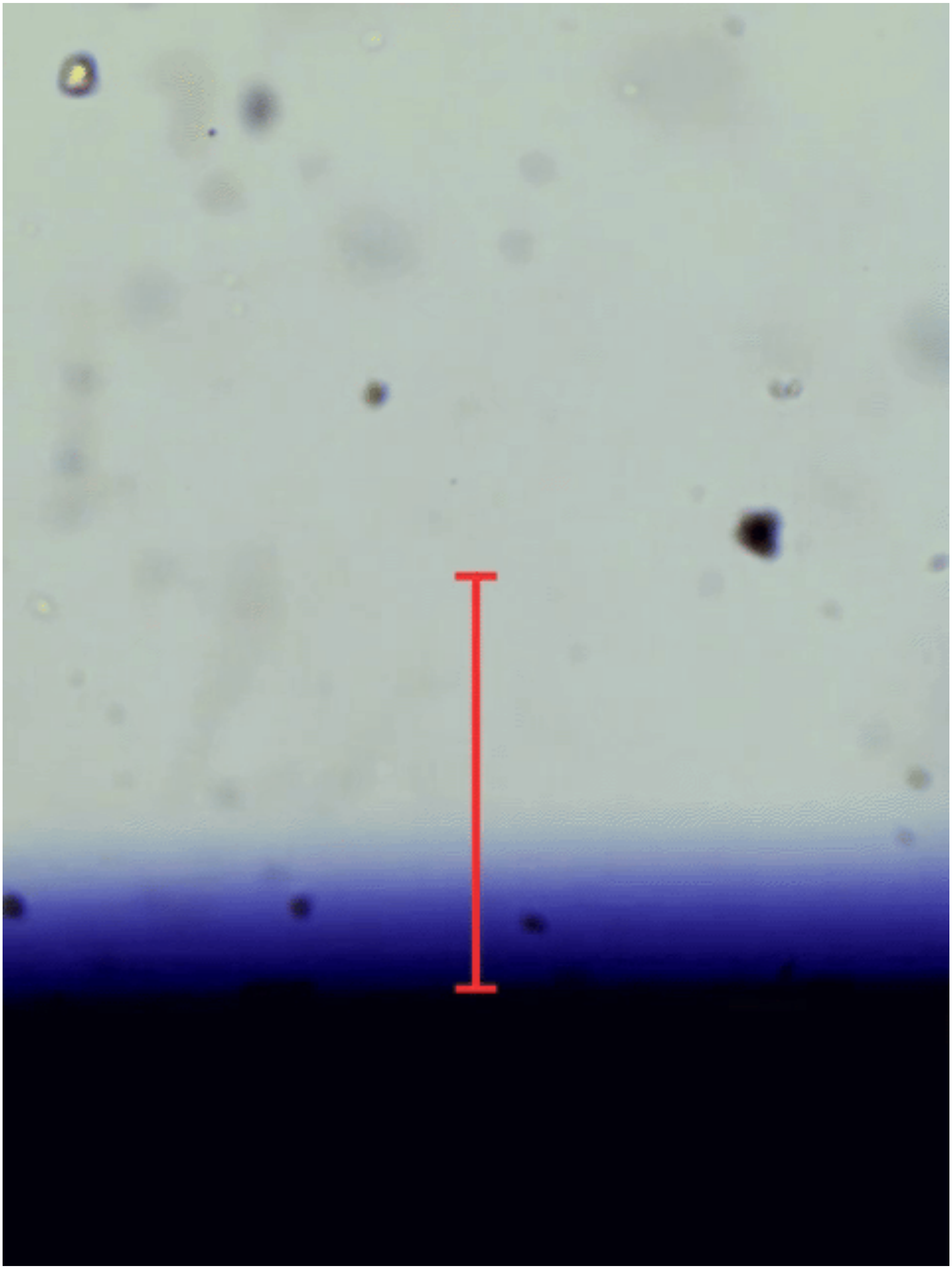

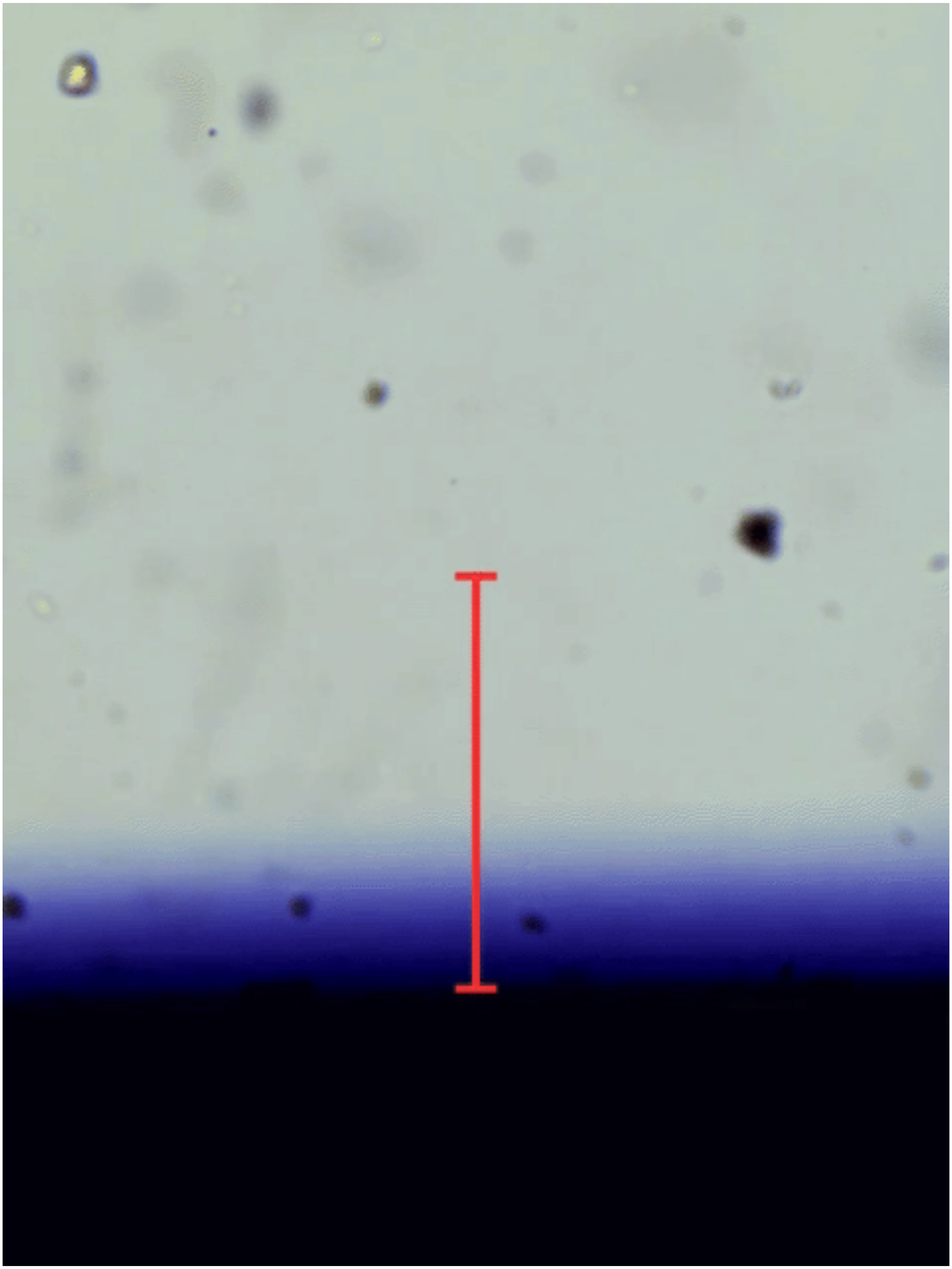

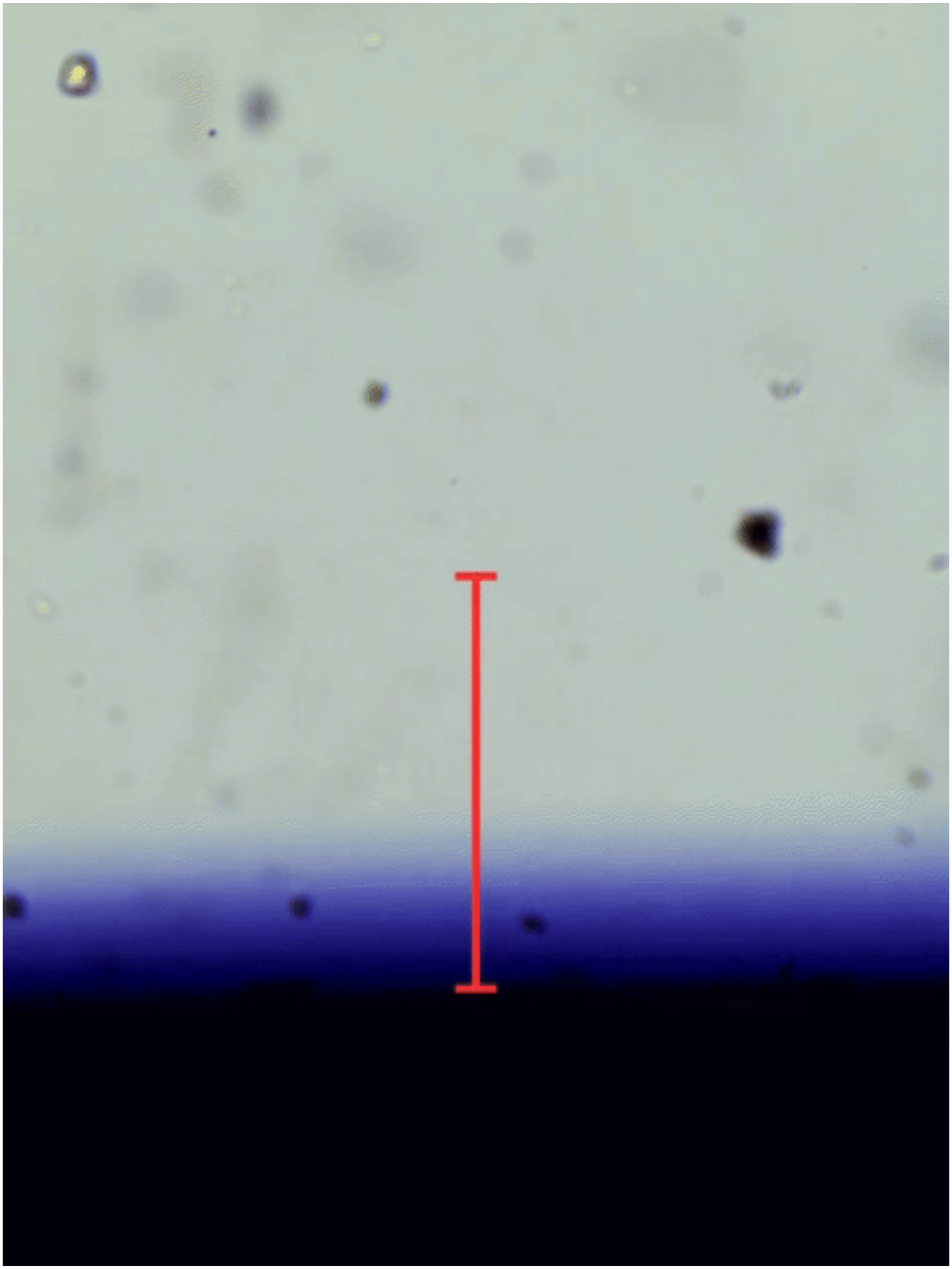

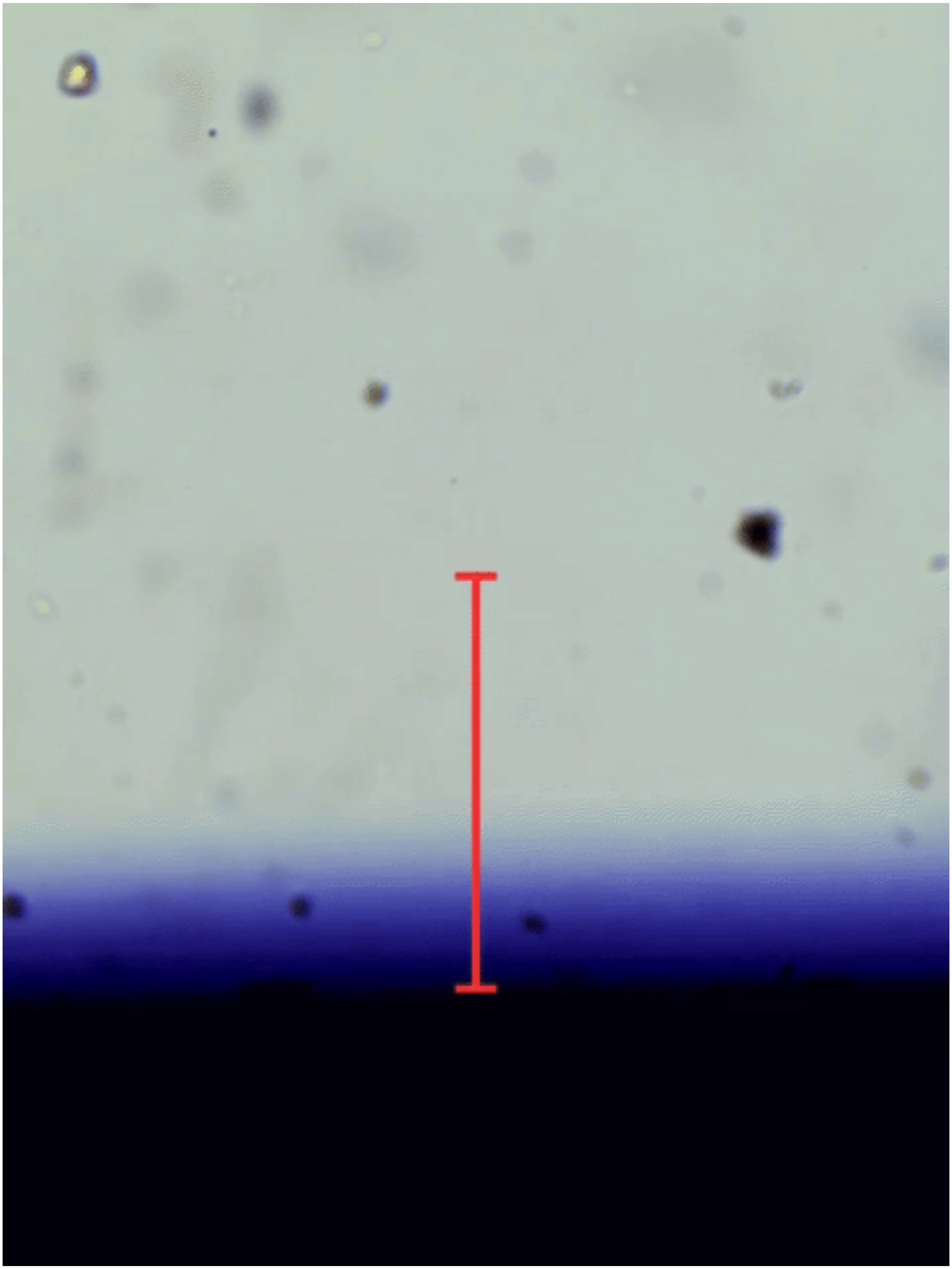

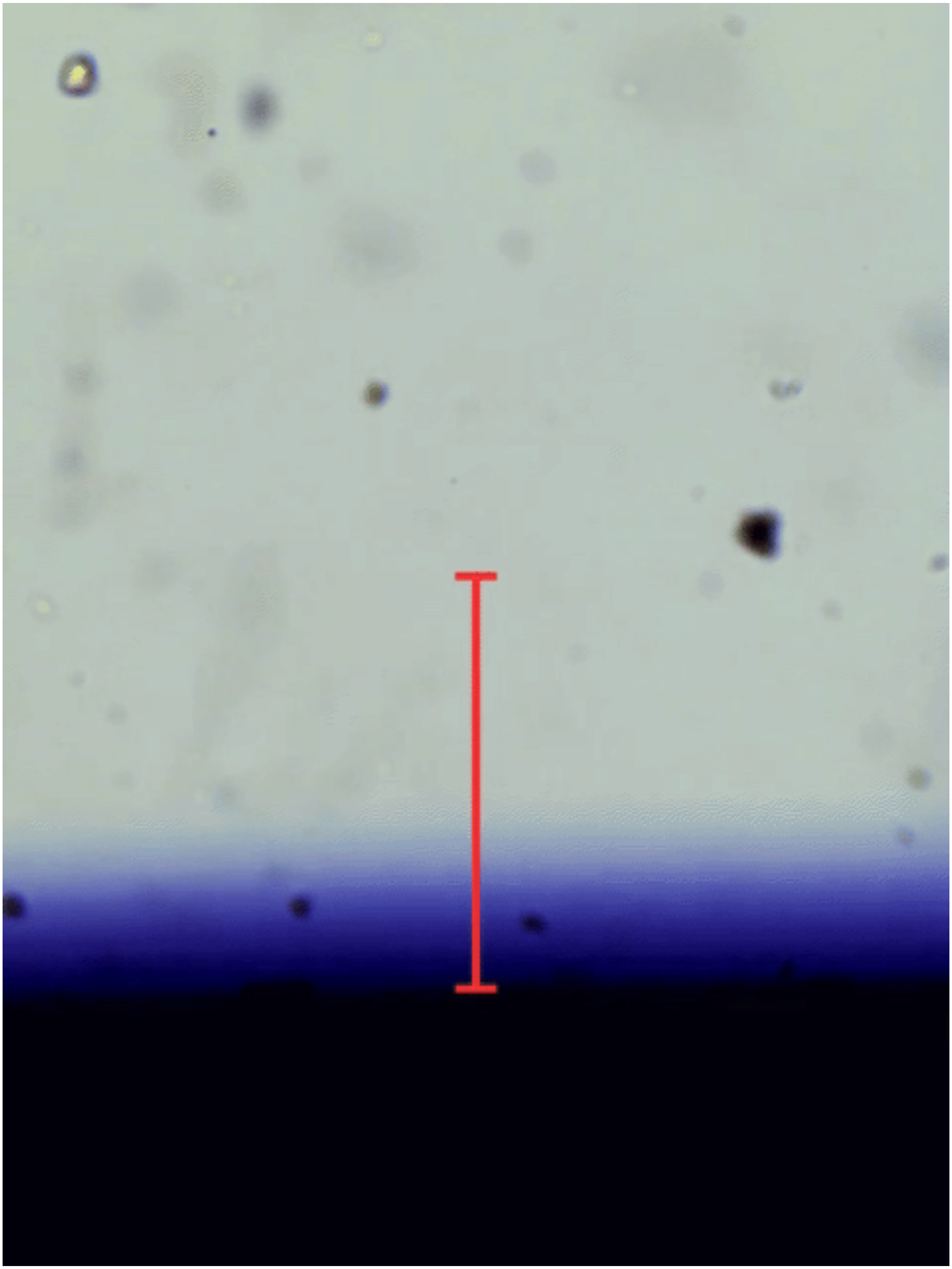

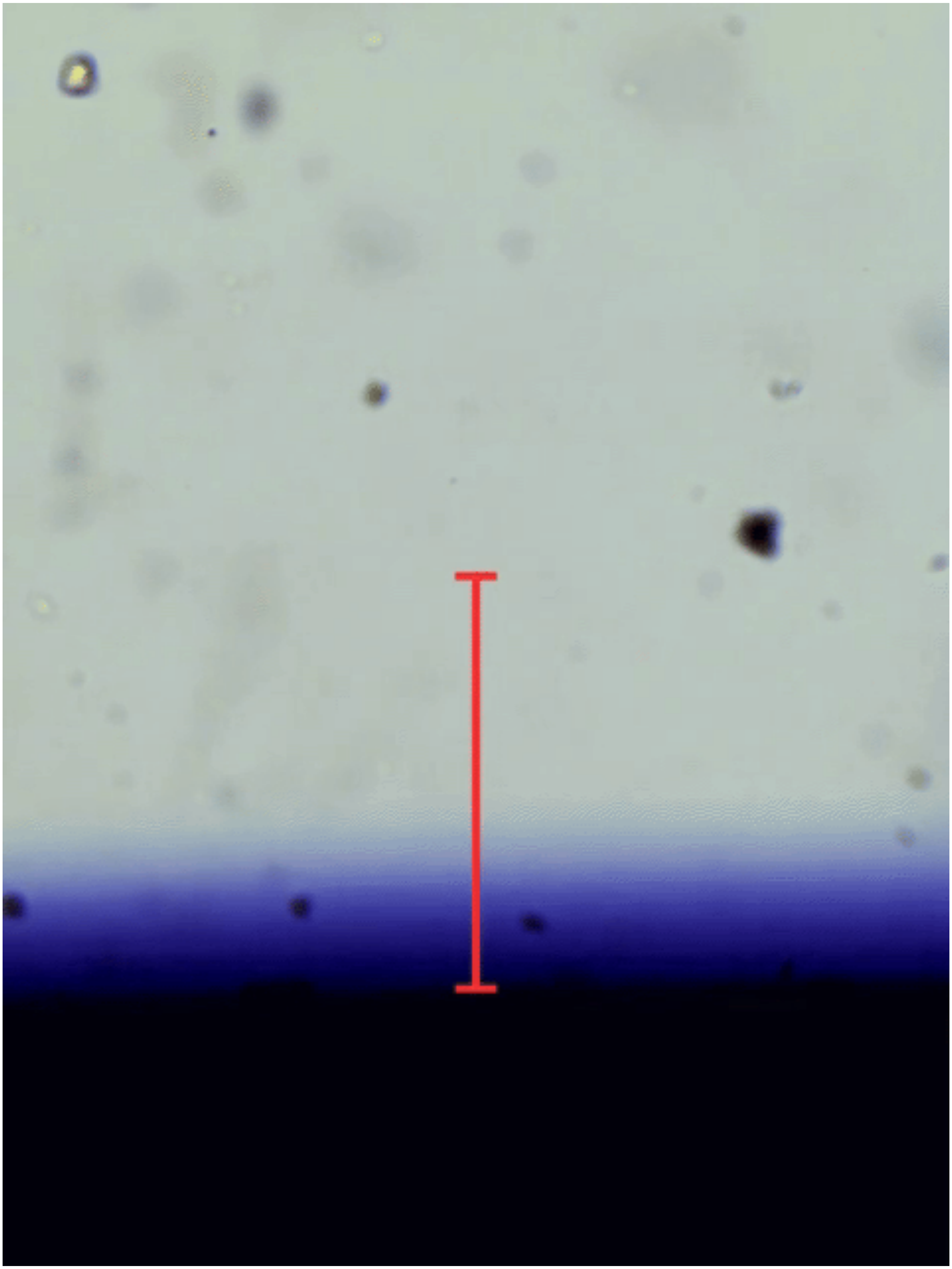

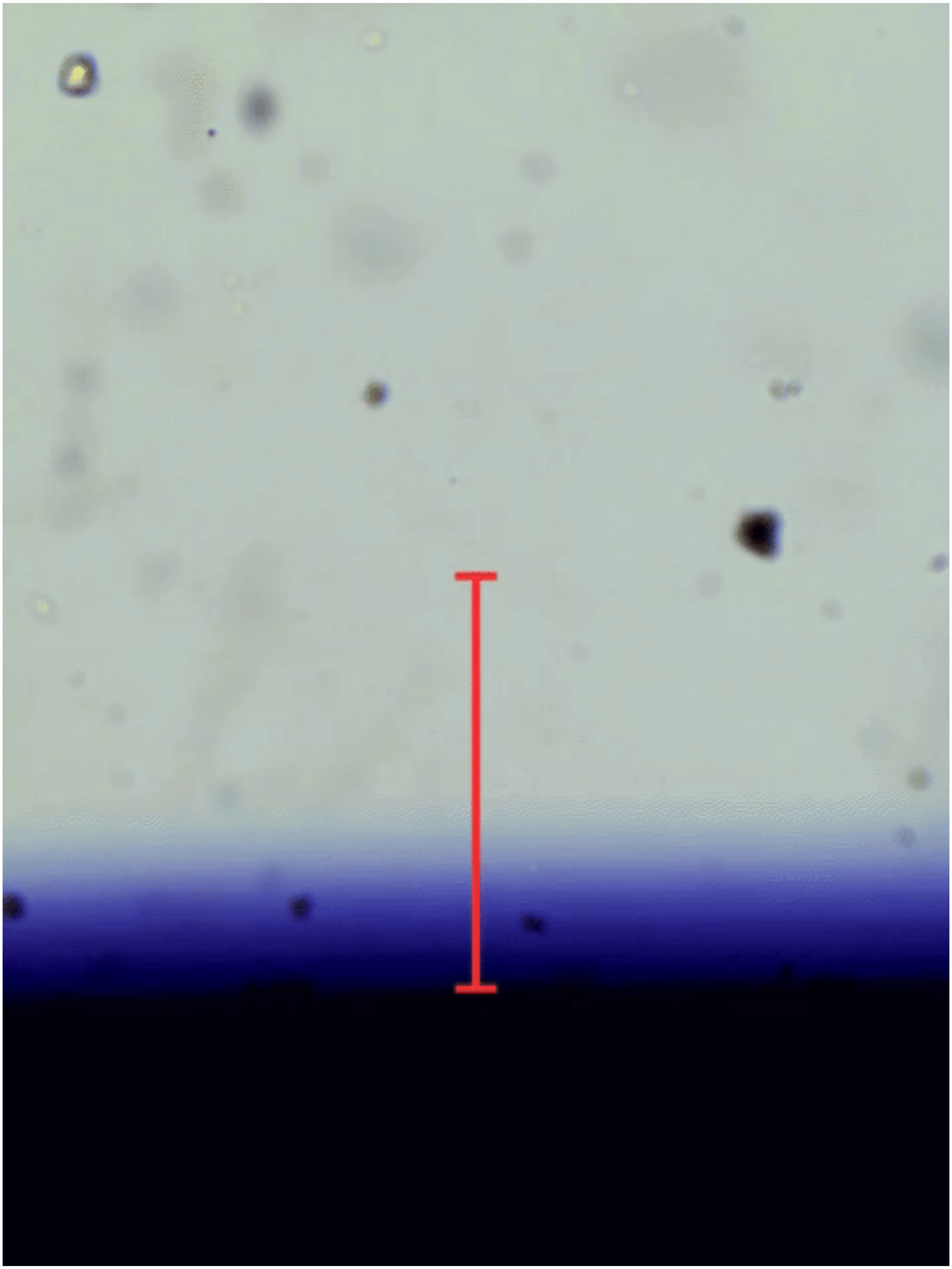

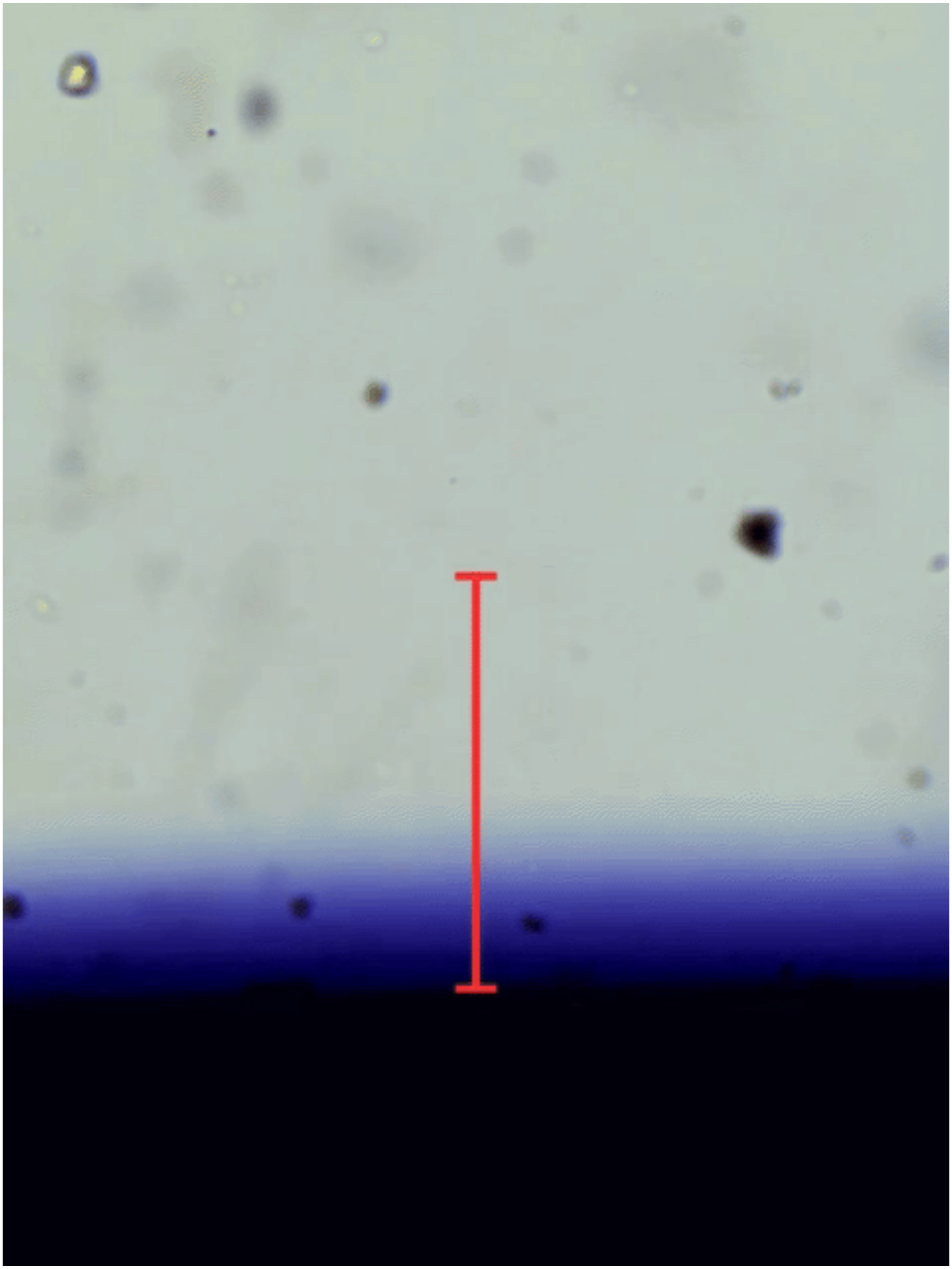

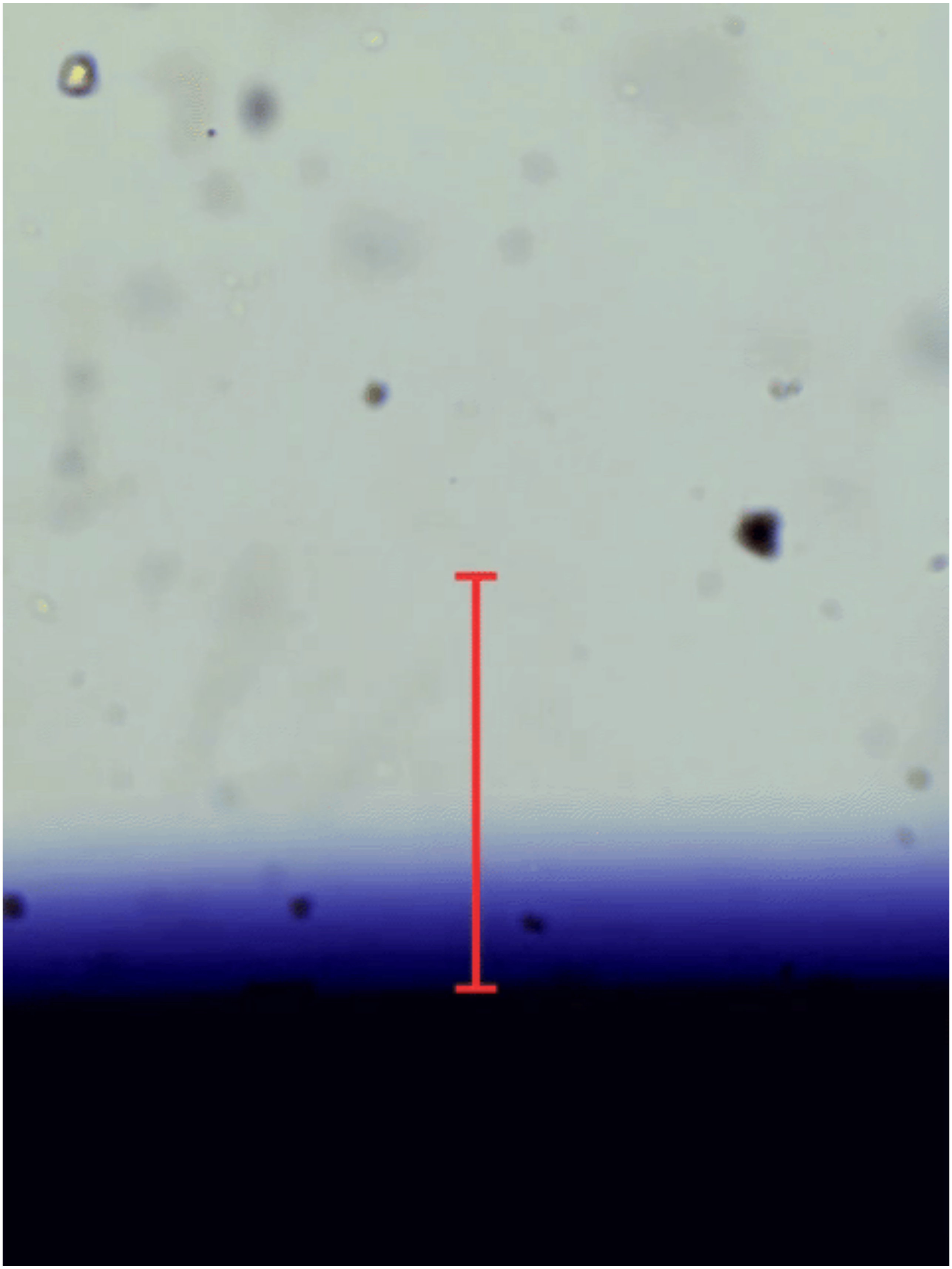

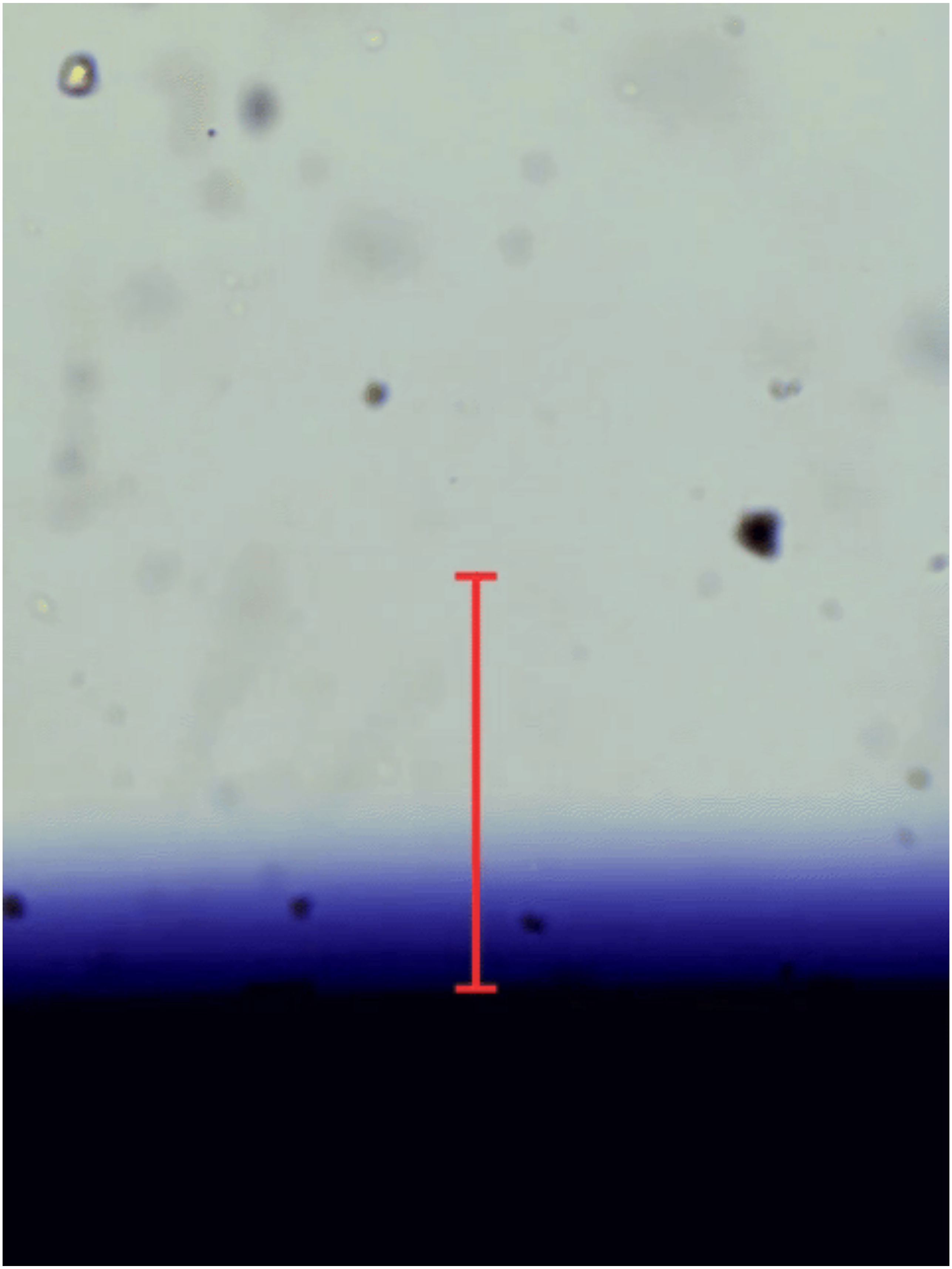

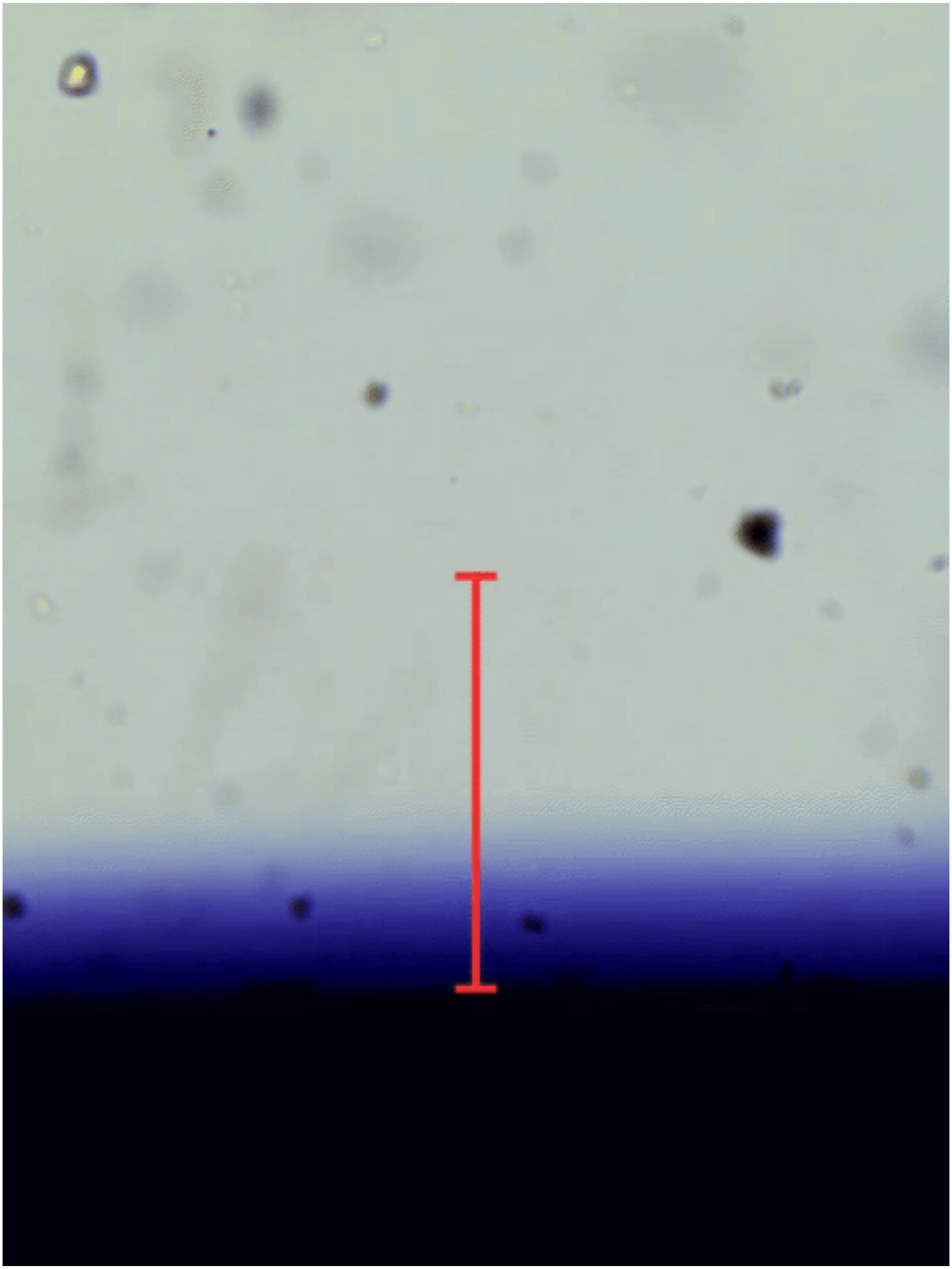

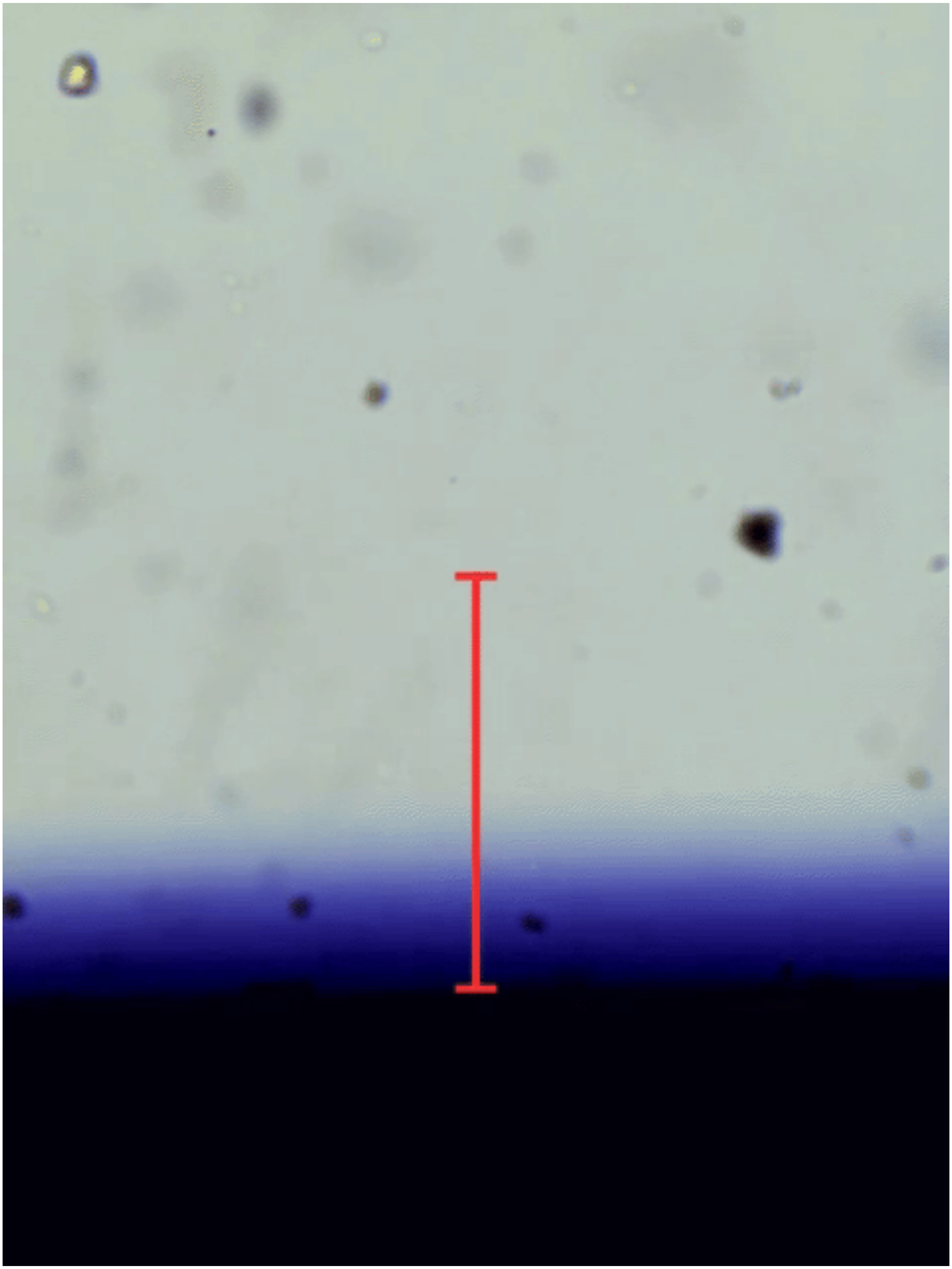

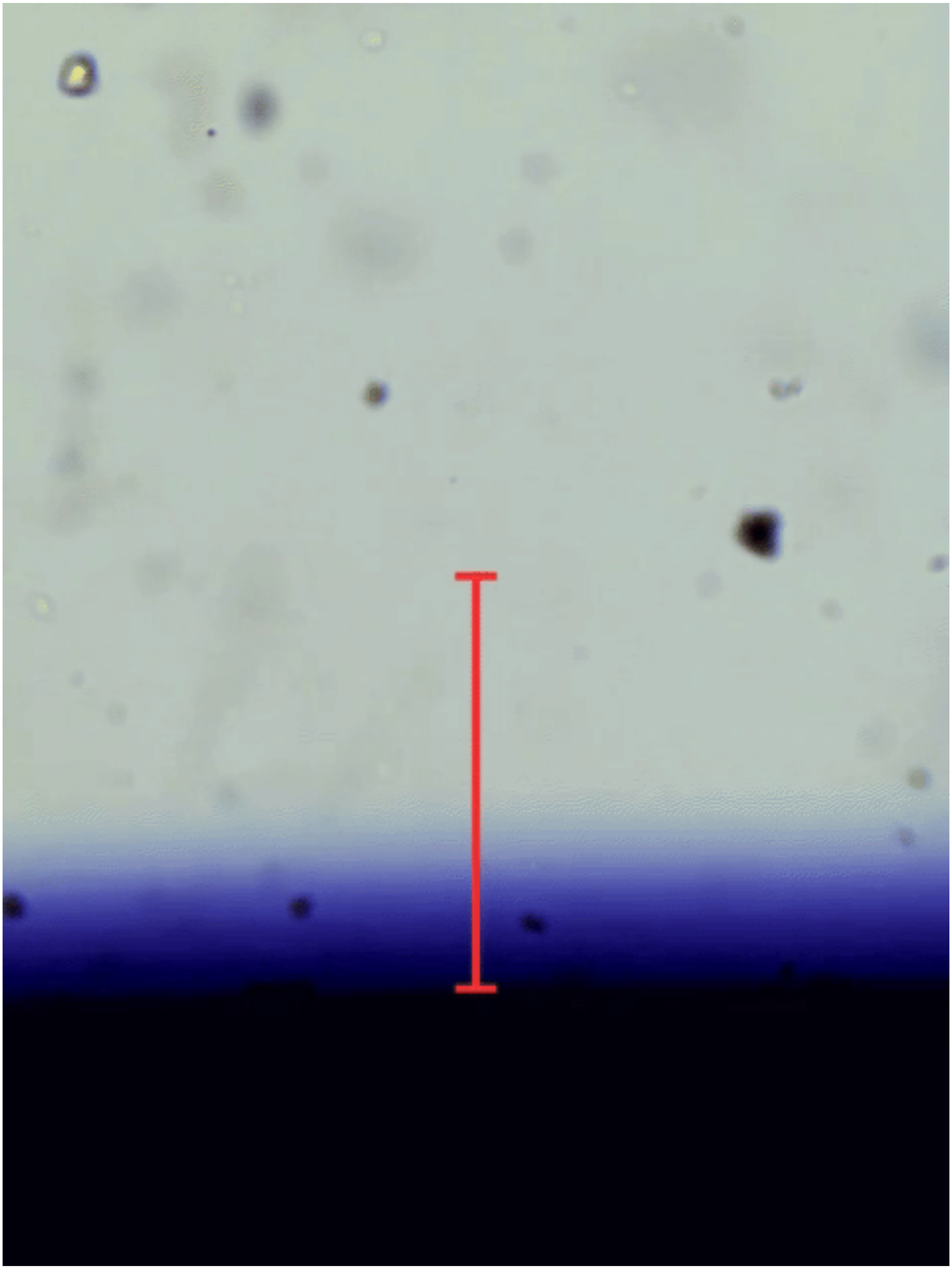

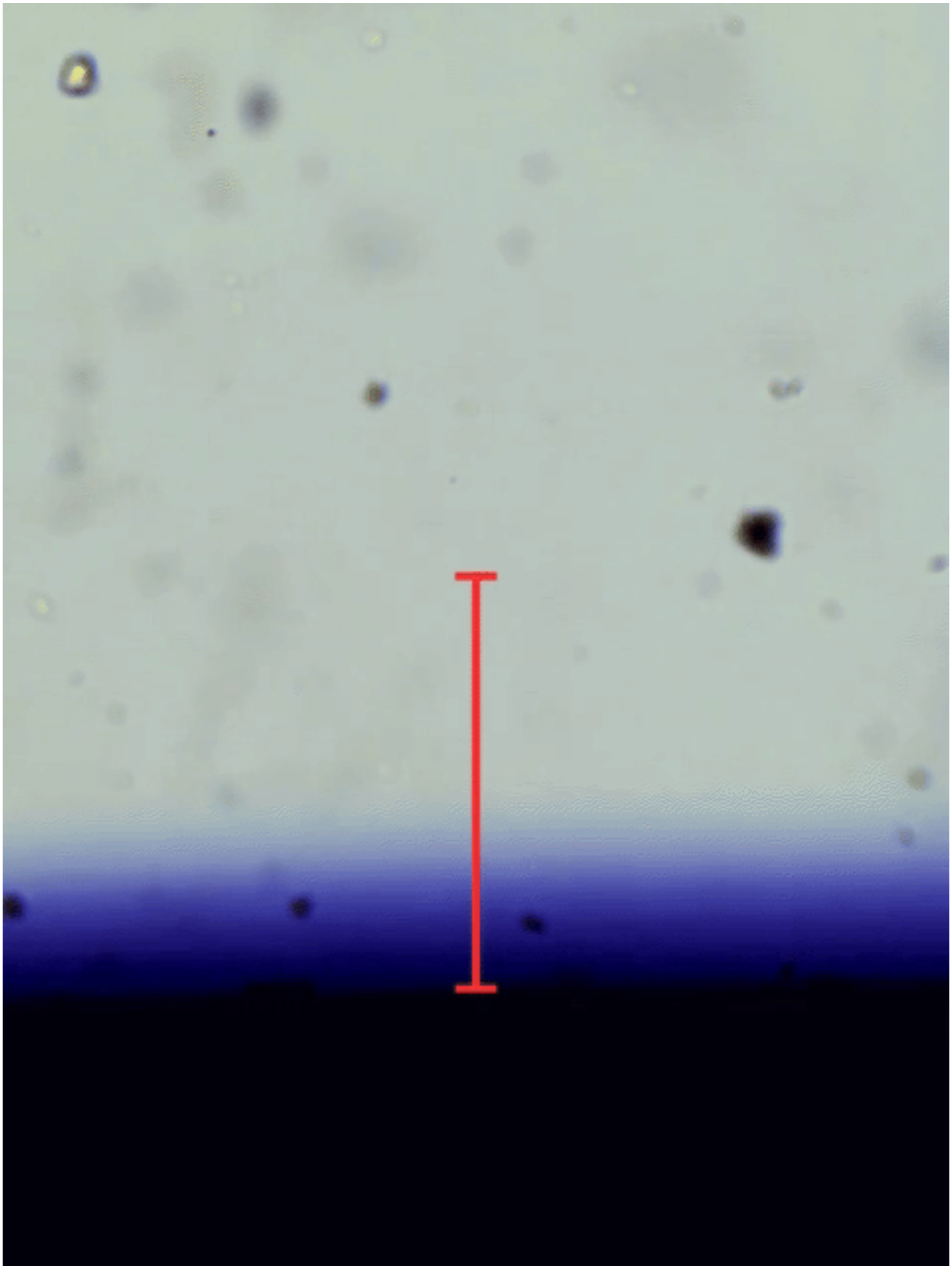

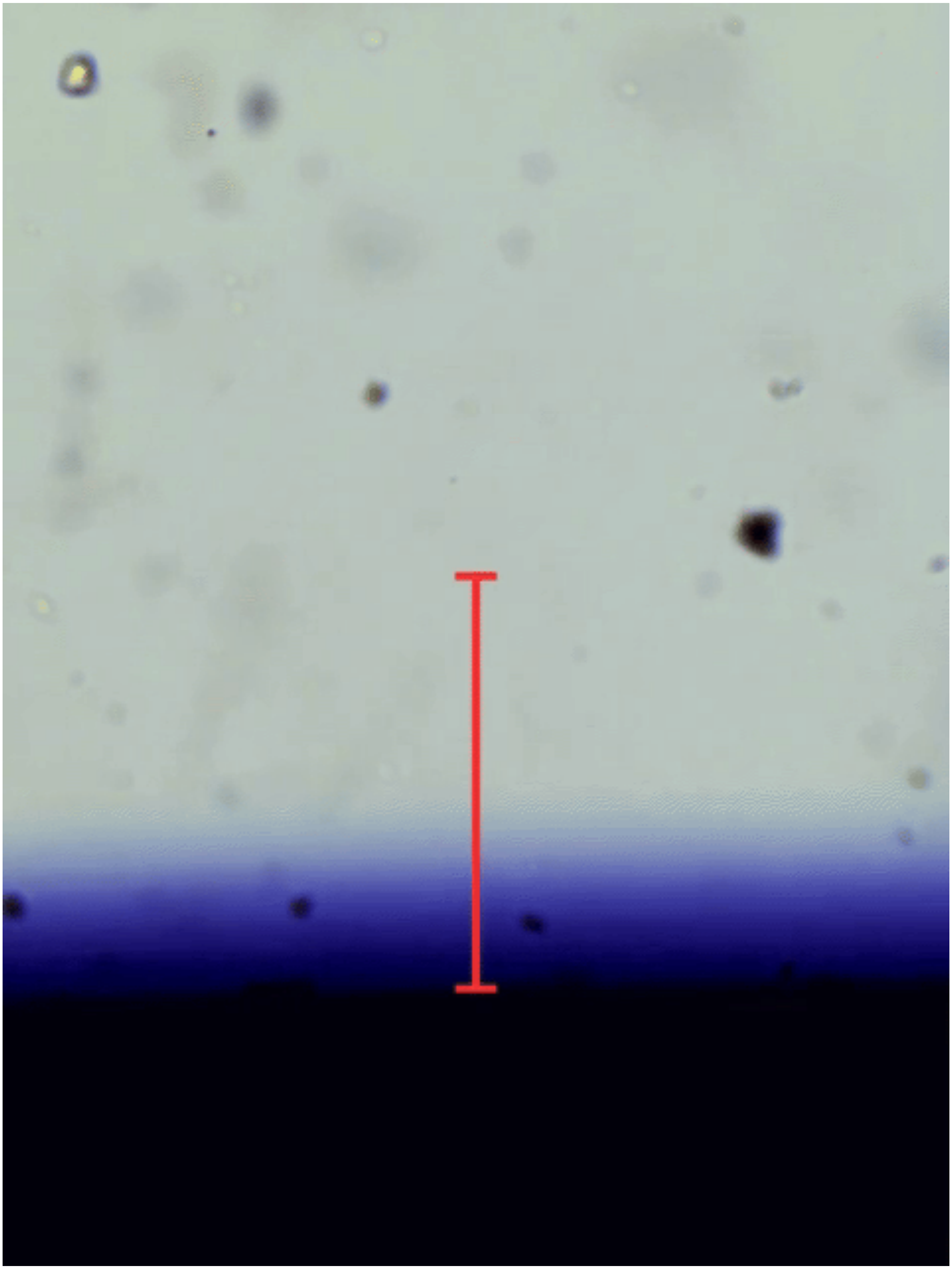

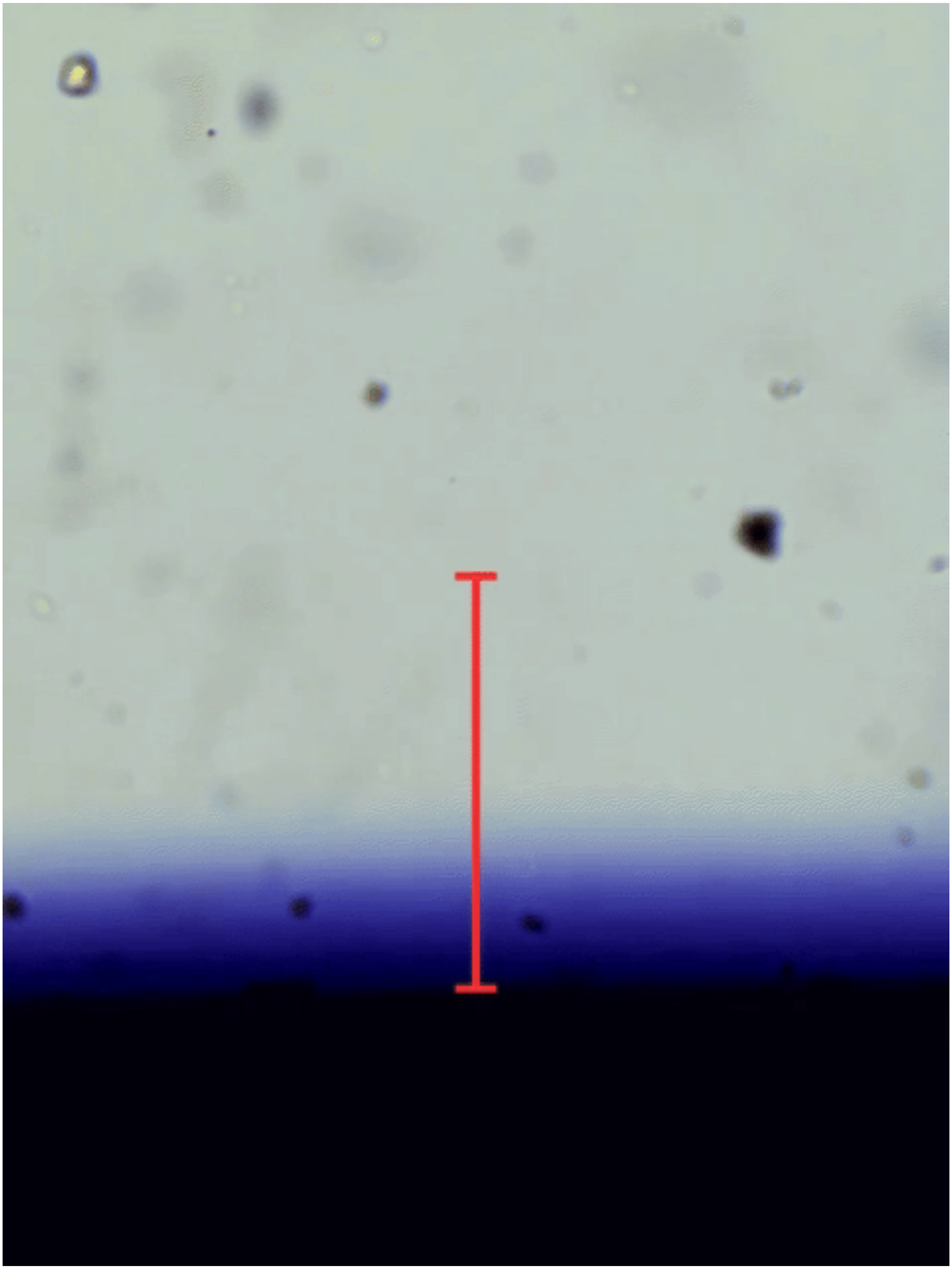

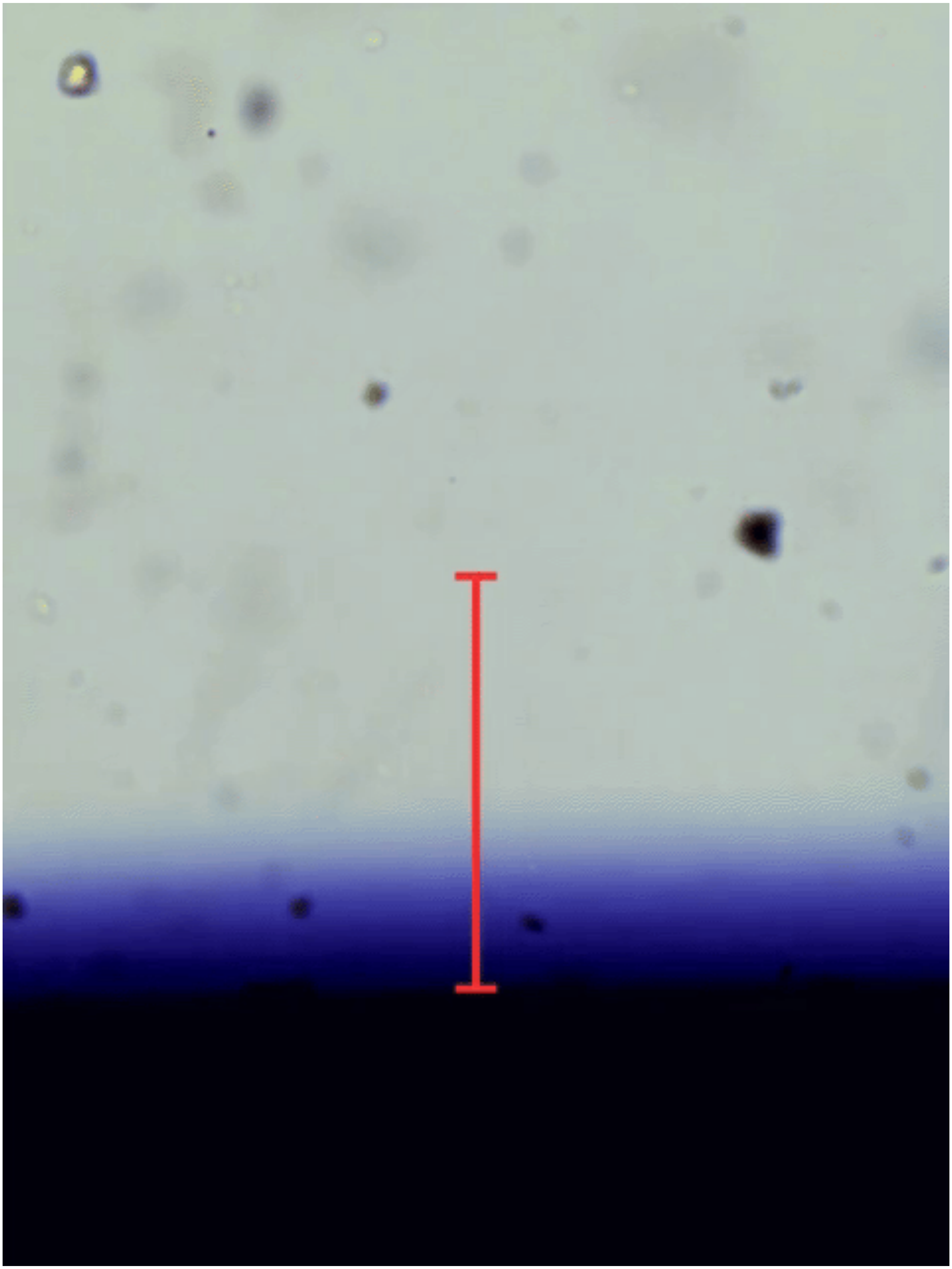

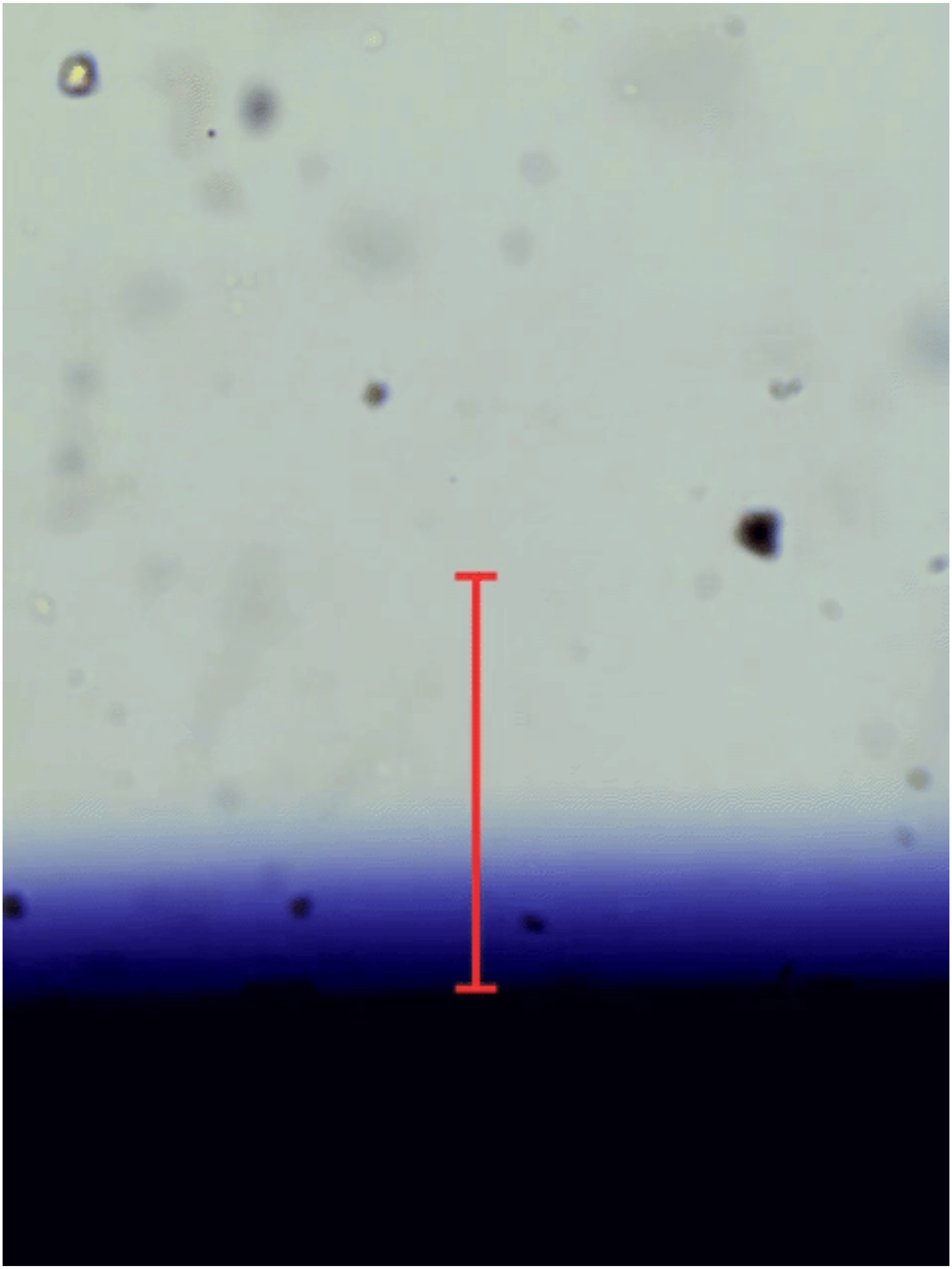

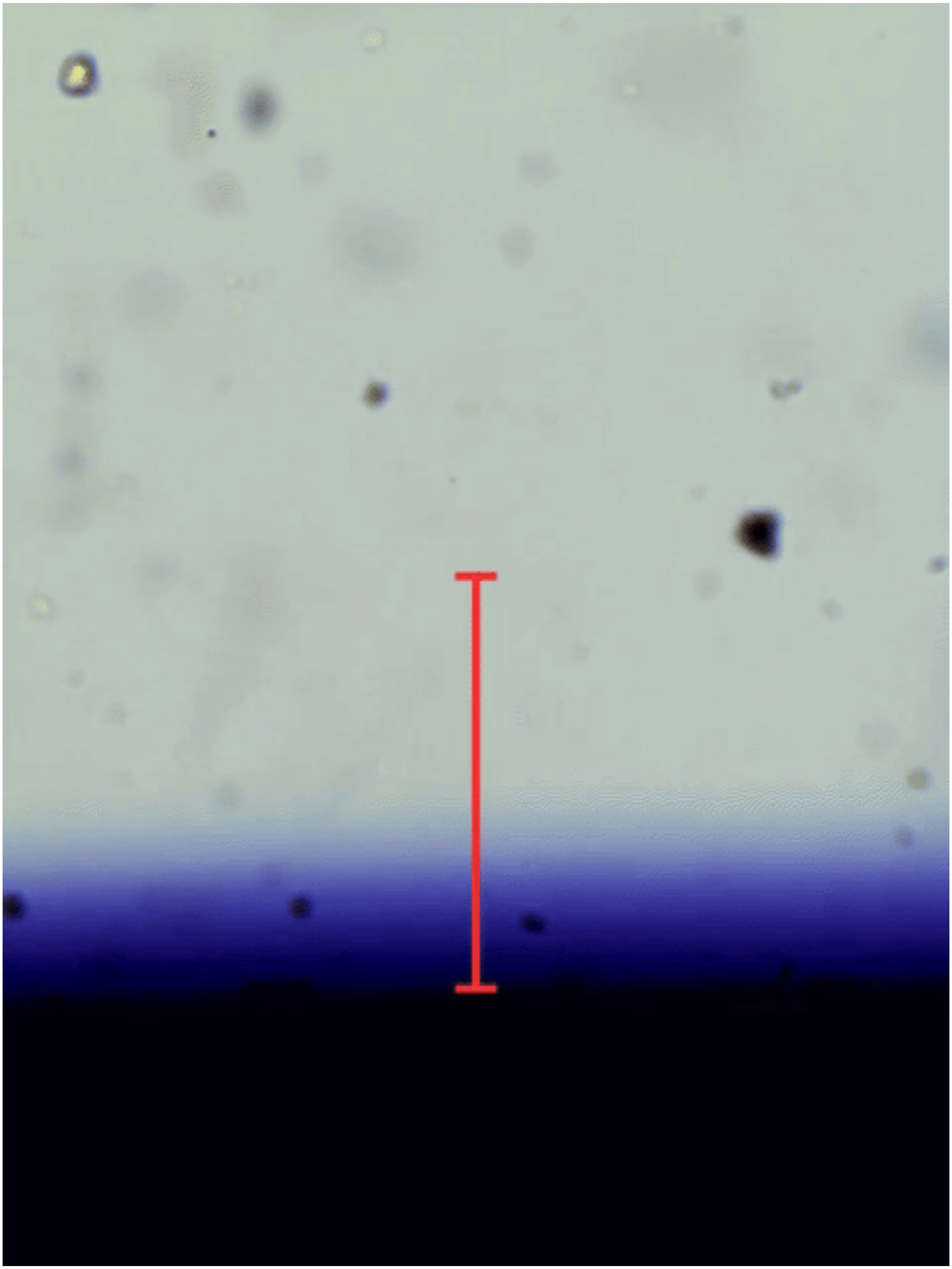

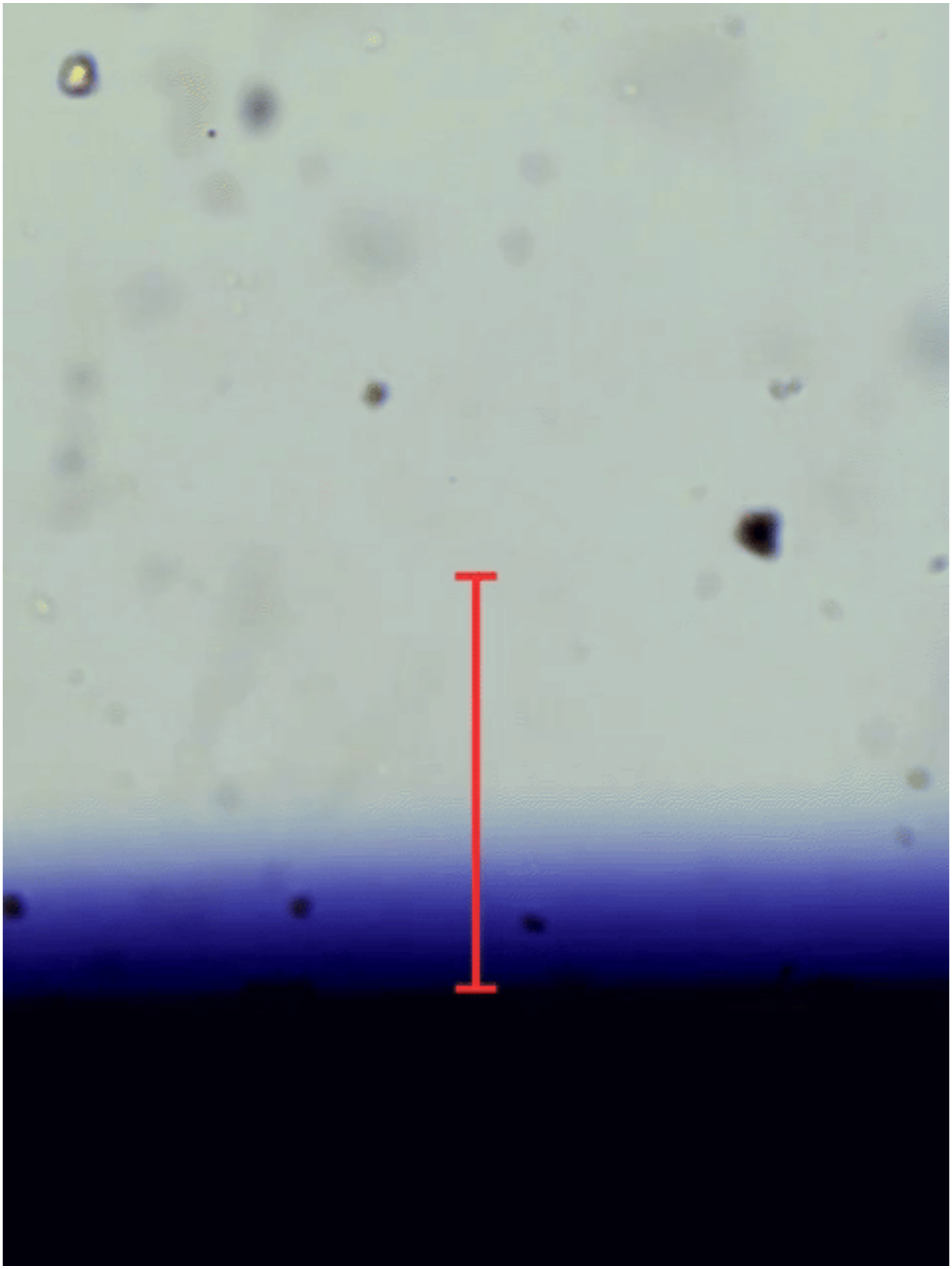

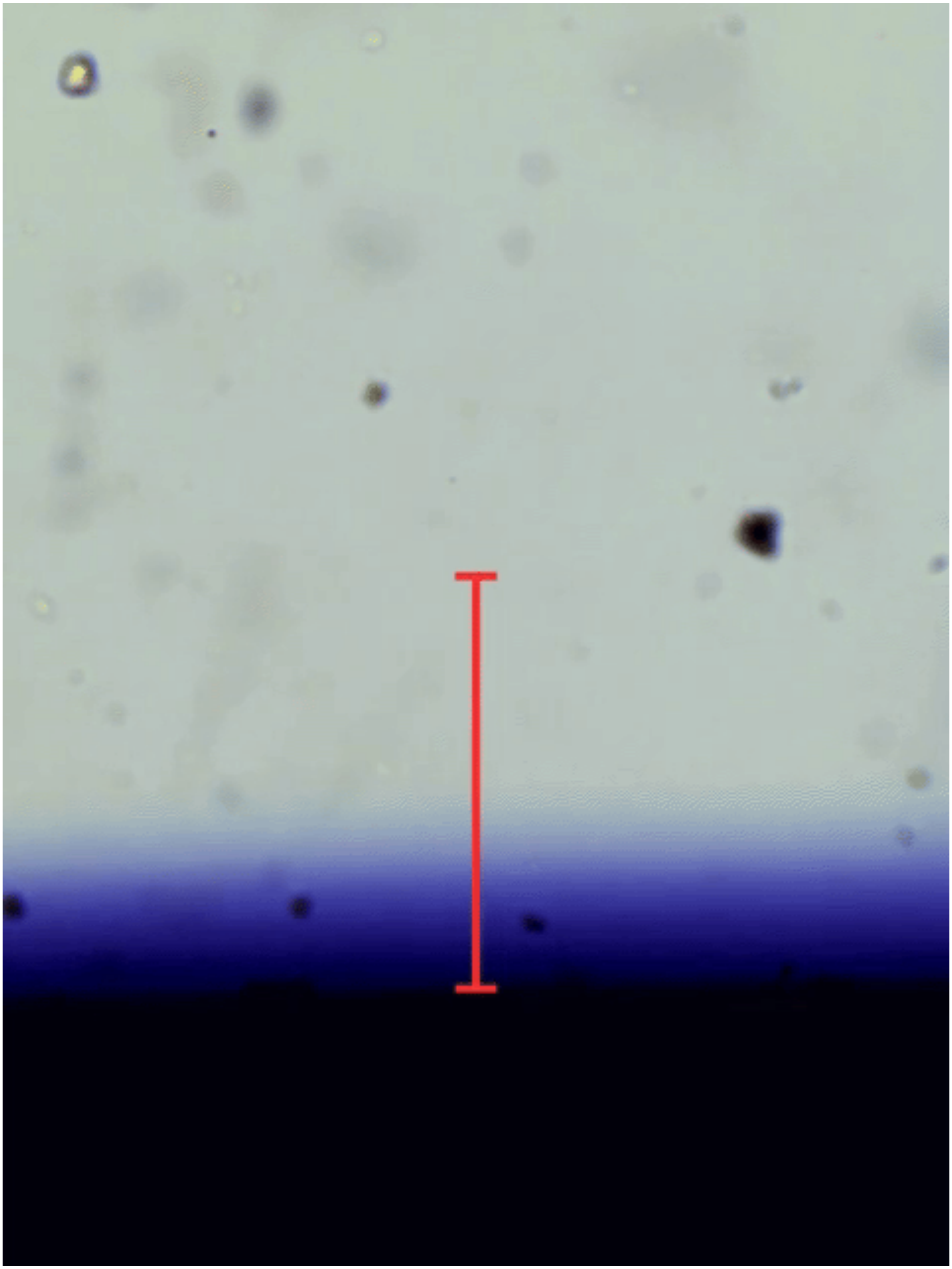

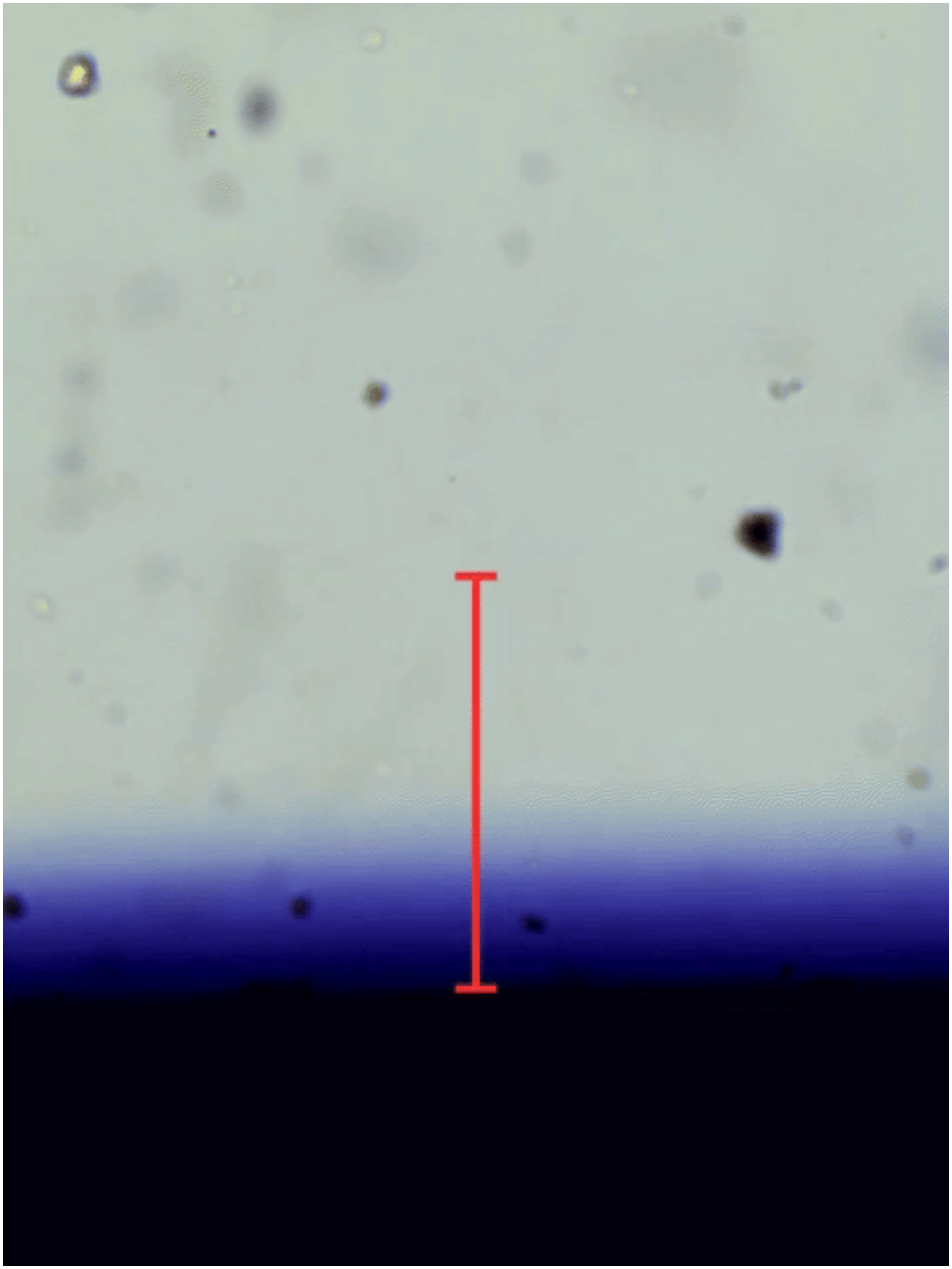

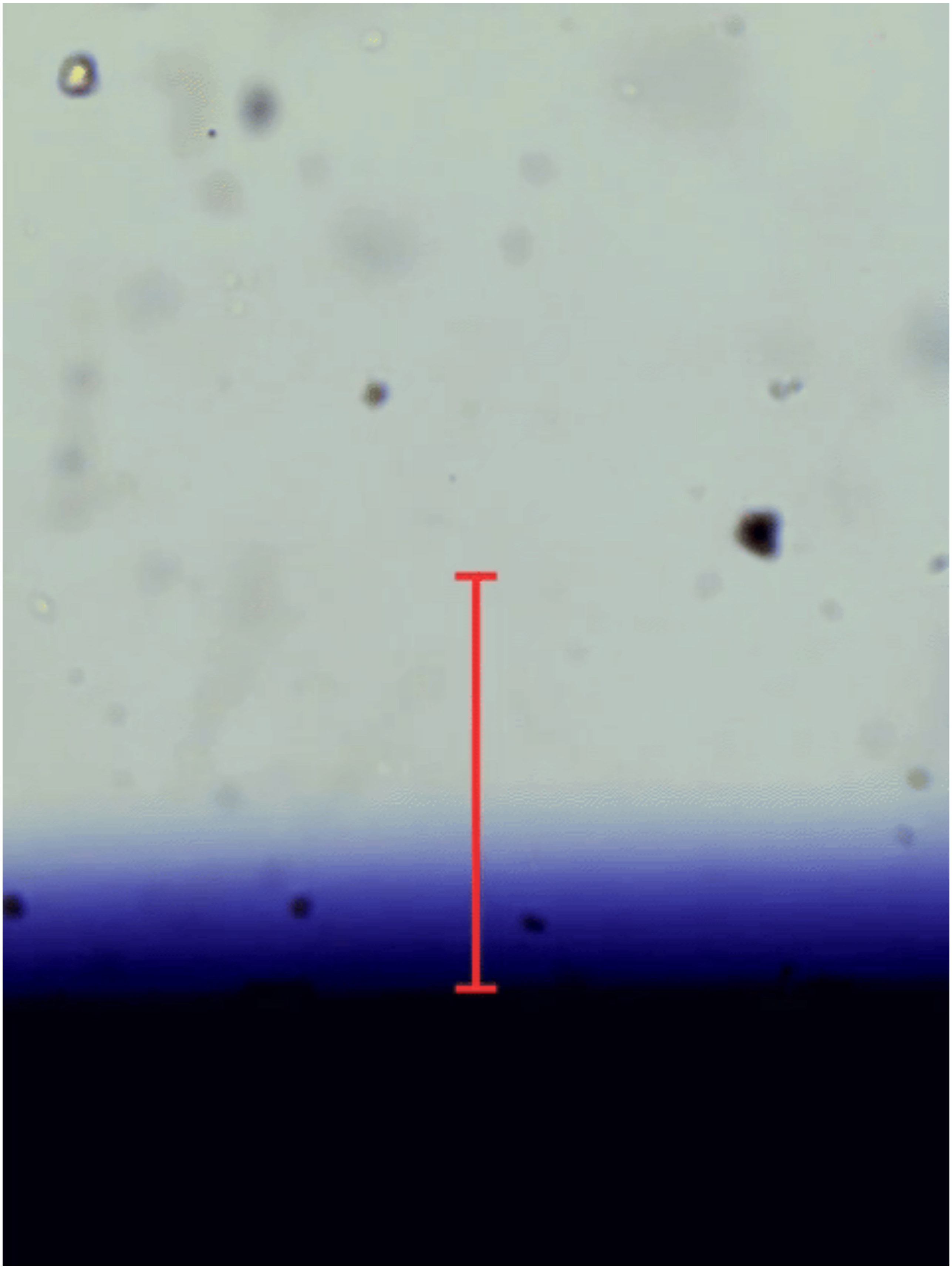

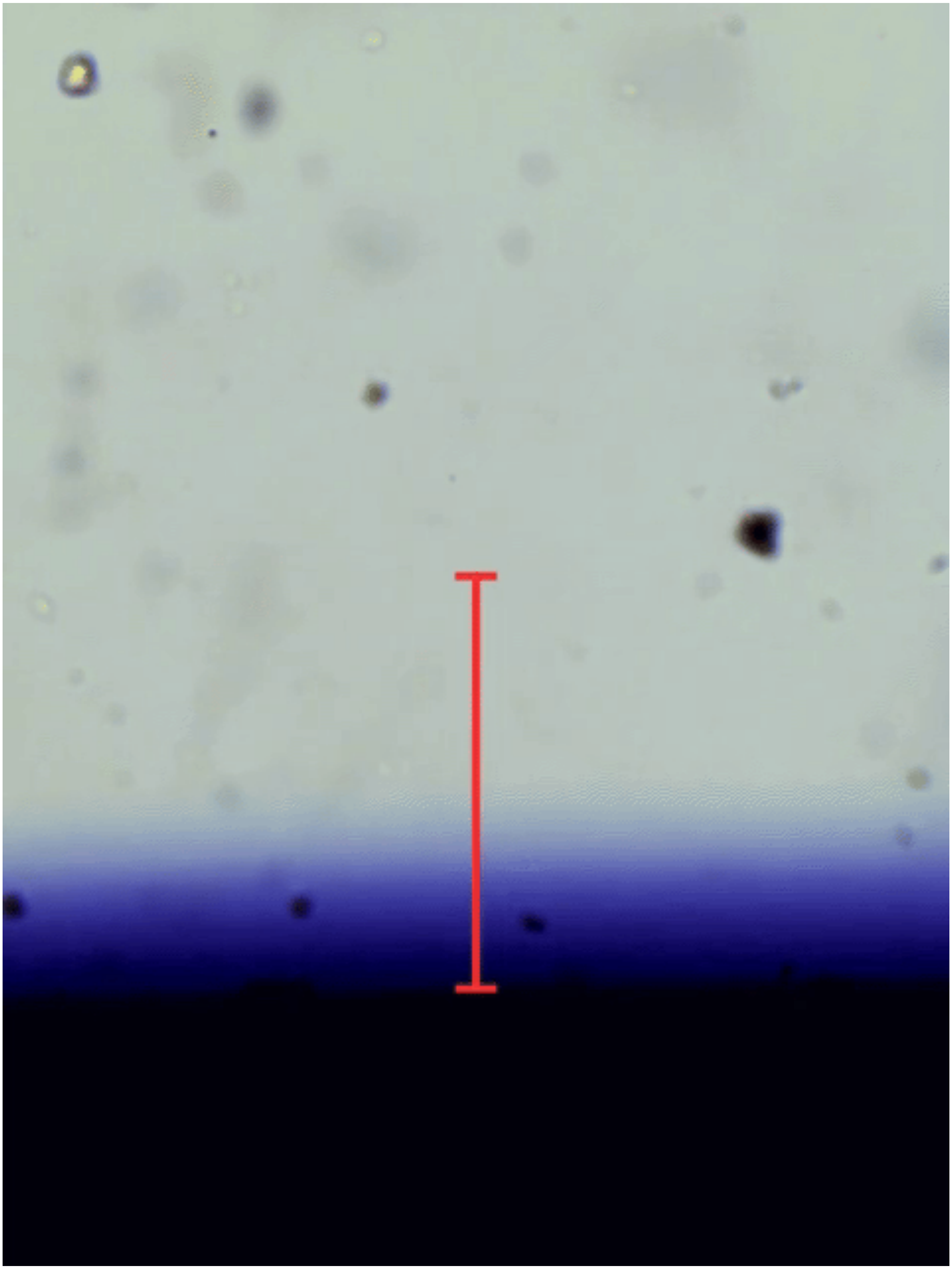

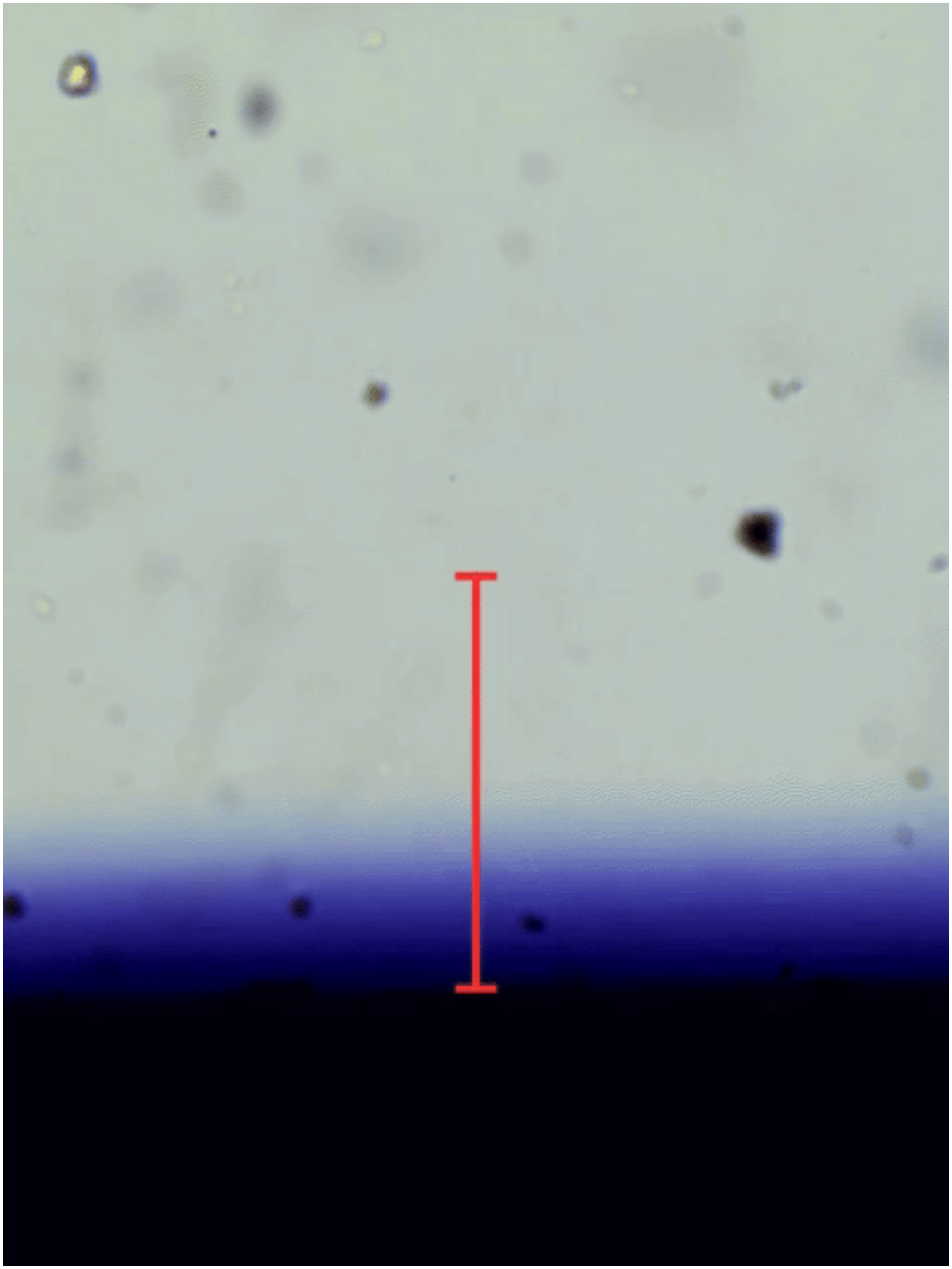

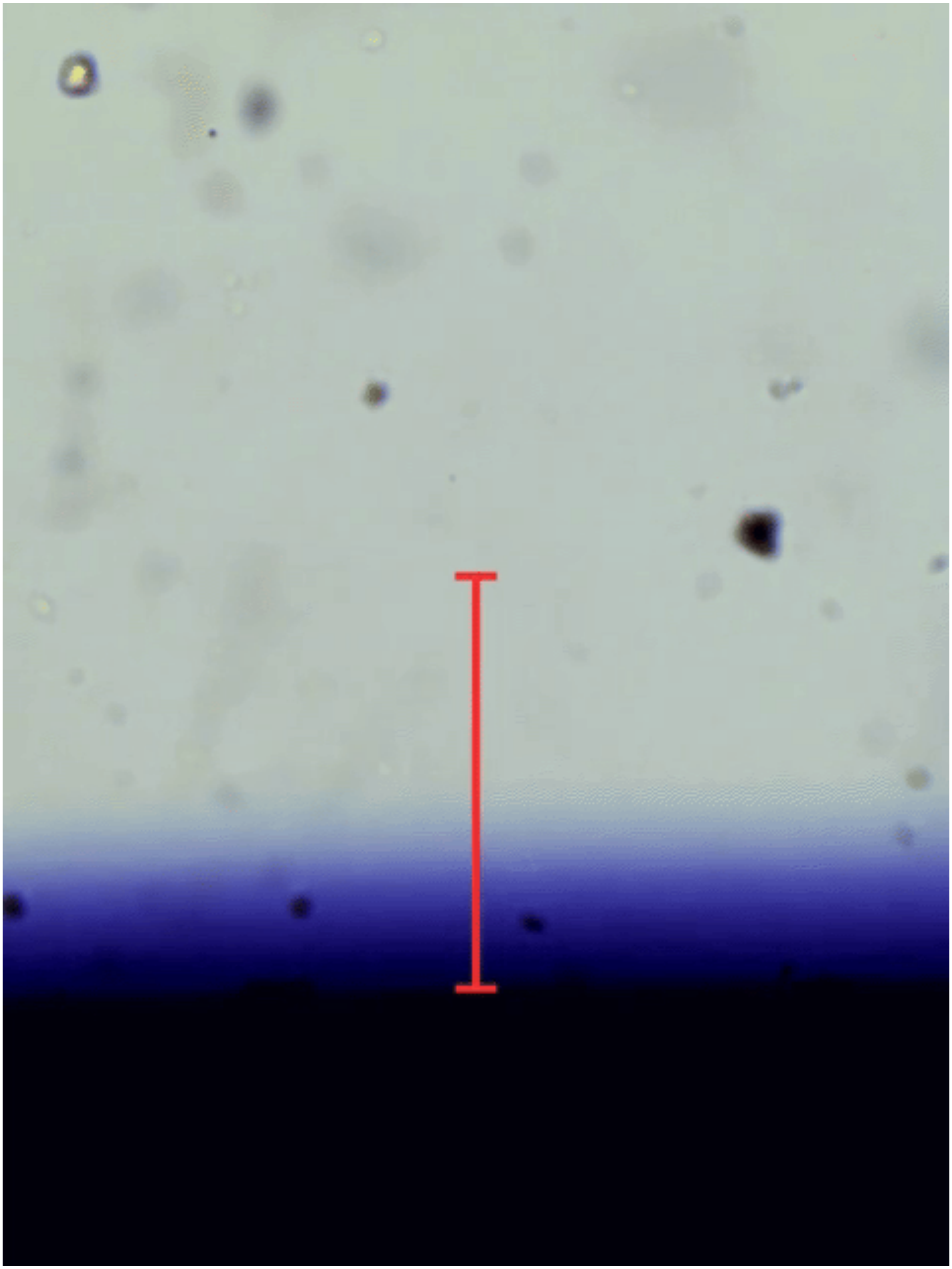

Supplement: Supplementary file 3 [file ed5c01710_si_009.pdf]
