# Supplementary material for: Thin-Layer Electrochemistry: Visualizing the Diffusion Layer During Chronoamperometry and Cyclic Voltammetry
Source: J Chem Educ. 2026 Jul 16;103(8):4704–10. doi: 10.1021/acs.jchemed.5c01710 (PMC13472089; doi:10.1021/acs.jchemed.5c01710)
Supplement: Supplementary file 4 [file ed5c01710_si_010.pdf]

Thin-Layer Electrochemistry: Visualizing the Diffusion Layer During Chronoamperometry and Cyclic Voltammetry

Mohammad Rafiee[a]*, Lida Khalafi[b], Keya Pandey[a], Mary Gipson[a]

[a] Division of Energy, Matter and Systems, School of Science and Engineering, University of Missouri−Kansas City, Kansas City, Missouri 64110, United States

[b] College of Arts and Sciences, Avila University, Kansas City, Missouri 64145, United States

Survey 2: Post-Activity Assessment of Learning and Conceptual Understanding

1. Electrochemical reactions involve both electrode processes and mass transport (e.g., diffusion). Based on your current knowledge, how well do you understand how these factors influence the shape of electrochemical responses (e.g., cyclic voltammograms)?

   ☐ Very well

   ☐ Somewhat well

   ☐ Slightly

   ☐ Not at all

2. In this experiment, methyl viologen (MV^{2+}) is reduced at the electrode to the colored species $MV^{+\bullet}$ during chronoamperometry. Based on this observation, how do you explain the observed decay in current over time? (Select all that apply.)

   ☐ The concentration of MV^{2+} near the electrode surface decreases during the experiment

   ☐ The diffusion layer grows over time, reducing the concentration gradient at the electrode

   ☐ The rate of mass transport of MV^{2+} to the electrode becomes limited by diffusion

   ☐ The bulk solution is rapidly depleted of MV^{2+}

   ☐ I am not sure

3. In cyclic voltammetry, the forward scan produces the colored $MV^{+\bullet}$ species, and the reverse scan causes the color to disappear. What does the reverse (backward) scan represent in this experiment?

   ☐ Oxidation of $MV^{+\bullet}$ back to the colorless MV^{2+} species at the electrode

   ☐ Reversal of the current direction without any chemical change

   ☐ Formation of additional reduced species at the electrode

   ☐ I am not sure

4. How well can you interpret a chronoamperometric plot (current vs time) to explain the relationship between current and diffusion over time?

   ☐ Very well

   ☐ Somewhat well

   ☐ Slightly

   ☐ Not at all

5. How confident are you in interpreting chronoamperometric plots (plots of current vs time)?

   ☐ Very confident

   ☐ Somewhat confident

   ☐ Unsure

   ☐ Not confident

6. How well can you use a cyclic voltammogram (current vs potential) to explain how current changes as the applied potential is swept forward and then reversed over time?

   ☐ Very well

   ☐ Somewhat well

   ☐ Slightly

   ☐ Not at all

7. How well can you imagine what happens at the electrode surface during an electrochemical experiment?

   ☐ Clearly

   ☐ Somewhat

   ☐ Vaguely

   ☐ Not at all

8. Describe one concept that became clearer to you as a result of this demonstration.
