# Supplementary material for: Thin-Layer Electrochemistry: Visualizing the Diffusion Layer During Chronoamperometry and Cyclic Voltammetry
Source: J Chem Educ. 2026 Jul 16;103(8):4704–10. doi: 10.1021/acs.jchemed.5c01710 (PMC13472089; doi:10.1021/acs.jchemed.5c01710)
Supplement: Supplementary file 5 [file ed5c01710_si_012.pdf]

Thin-Layer Electrochemistry: Visualizing the Diffusion Layer During Chronoamperometry and Cyclic Voltammetry

Mohammad Rafiee[a]*, Lida Khalafi[b], Keya Pandey[a], Mary Gipson[a]

[a] Division of Energy, Matter and Systems, School of Science and Engineering, University of Missouri−Kansas City, Kansas City, Missouri 64110, United States

[b] College of Arts and Sciences, Avila University, Kansas City, Missouri 64145, United States

Survey 1: Pre-Activity Assessment of Electrochemical Concepts

1. Electrochemical reactions involve both electrode processes and mass transport (e.g., diffusion). Based on your current knowledge, how well do you understand how these factors influence the shape of electrochemical responses (e.g., cyclic voltammograms)?

   ☐ Very well

   ☐ Somewhat well

   ☐ Slightly

   ☐ Not at all

2. In a chronoamperometry (CA) experiment, the current typically changes over time. What factors are responsible for this behavior? (Select all that apply.)

   ☐ The concentration of reactants near the electrode surface changes during the experiment

   ☐ The diffusion layer grows over time, altering the concentration gradient at the electrode

   ☐ Changes in the electrode surface area during the experiment

   ☐ The bulk solution concentration changes significantly during the experiment

   ☐ I am not sure

3. Cyclic voltammetry involves scanning the potential in a forward direction and then reversing the scan. What is the purpose of the reverse (backward) scan?

   ☐ To observe the reverse redox process of species formed during the forward scan

   ☐ To maintain a constant current throughout the experiment

   ☐ To increase the speed of the experiment

   ☐ I am not sure

4. How well can you interpret a chronoamperometric plot (current vs time) to explain the relationship between current and diffusion over time?

   ☐ Very well

   ☐ Somewhat well

   ☐ Slightly

   ☐ Not at all

5. How confident are you in interpreting chronoamperometric plots (plots of current vs time)?

☐ Very confident

☐ Somewhat confident

☐ Unsure

☐ Not confident

6. How well can you use a cyclic voltammogram (current vs potential) to explain how current changes as the applied potential is swept forward and then reversed over time?

☐ Very well

☐ Somewhat well

☐ Slightly

☐ Not at all

7. How well can you imagine what happens at the electrode surface during an electrochemical experiment?

☐ Clearly

☐ Somewhat

☐ Vaguely

☐ Not at all
